# Innovative PDK1‐Degrading PROTACs Transform Cancer Aerobic Glycolysis and Induce Immunogenic Cell Death in Breast Cancer

**DOI:** 10.1002/EXP.20240031

**Published:** 2025-05-07

**Authors:** Aohua Deng, Renming Fan, Jiakui Gou, Ruoxi Sang, Ruizhuo Lin, Ting Zhao, Junyan Zhuang, Yongrui Hai, Jialin Sun, Gaofei Wei

**Affiliations:** ^1^ Institute of Medical Research Northwestern Polytechnical University Xi'an Shaanxi China; ^2^ Research & Development Institute of Northwestern Polytechnical University in Shenzhen Guangdong China; ^3^ Edinburgh Kidney Research Group Centre for Cardiovascular Science Queen's Medical Research Institute University of Edinburgh Edinburgh UK

**Keywords:** immunogenic cell death, PROTAC, pyruvate dehydrogenase kinase, Warburg effect

## Abstract

Cancer cells are characterized by the Warburg effect, which hijacks glycolysis and hinders OXPHOS. Pyruvate dehydrogenase kinase 1 (PDK1) is a key modulator in the Warburg effect and is highly expressed in tumor cells. We utilize PROTAC technology to design compounds that could achieve long‐lasting degradation on PDK1. After screening anti‐tumor activity in vitro, we selected a top compound **A04**, among 22 chemical candidates in various structures. Compared to a conventional PDK1 inhibitor, **A04** dramatically improves over 1000‐fold proliferation inhibition efficacy. Besides, **A04** reverses Warburg effect and causes tumor apoptosis. In vivo, **A04** achieves potent therapeutic efficacy in tumor‐bearing mice and dramatically prolongs their lifetime after surgery resection. For the mechanism, **A04** induces immunogenic cell death and reverses immunosuppression in the TME to enhance antitumor immunoreactivity. Further, transcriptome analysis verifies the mechanisms and uncovers fluctuation in cancer related pathways. Combination with αPD‐L1 improves therapeutic efficacy and promotes multiple immunocytes infiltration. In conclusion, we first utilize PROTAC technology on modulating aberrant expressed metabolic enzyme PDK1 in cancer cells and achieve a great pharmacological effect, rendering it promising for energy‐aberrant cancer therapy.

## Graphical abstract

In this study, A04, a compound targeting the degradation of metabolic enzyme PDK1, was developed by PROTAC technology, which significantly inhibited tumor proliferation by disrupting the Warburg effect in cancer cells. In the mouse model, A04 could prolong postoperative survival, and also remodel the immunosuppressive microenvironment, which could synergistically enhance the anti‐tumor immune response in combination with αPD‐L1.

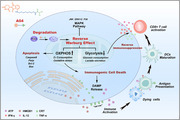

## 1 Introduction

Cancer cells display an aberrant metabolic phenotype in order to meet the demands of rapid proliferation [1, 2]. This metabolic phenomenon, known as aerobic glycolysis or the Warburg effect [3], is characterized by high glucose consumption and substantial lactate secretion, achieved by shifting from oxygen‐dependent oxidative phosphorylation (OXPHOS) to oxygen‐independent glycolysis, even under normoxic conditions [4]. Pyruvate dehydrogenase kinase 1 (PDK1), a key enzyme in the Warburg Effect, is highly expressed in cancer cells and plays a pivotal role in reprogramming tumor metabolism between glycolysis and OXPHOS [5]. PDK1 accomplishes this reprogramming by phosphorylating a serine residue on the E1α subunit of pyruvate dehydrogenase (PDH), thus preventing the flux of pyruvate into the citric cycle and promoting its conversion to lactate [5, 6]. Consequently, inhibiting PDK1 can block glycolysis and activate OXPHOS. Additionally, OXPHOS‐derived reactive oxygen species (ROS) can induce apoptosis in cancer cells. These findings collectively suggest that PDK1 is a promising target for anticancer therapy.

Dichloroacetic acid (DCA) is a classical PDK1 inhibitor that has been incorporated into various drug design strategies [7, 8, 9]. DCA serves as a ligand for PDK and effectively reverses the Warburg Effect, resulting in metabolic modulation and tumor apoptosis [10, 11, 12]. Several early‐phase clinical trials have reported therapeutic effects of DCA in patients with multiple solid tumors [13, 14]. However, the severe toxic effects associated with high doses of DCA remain a challenge. Previous research from our team has demonstrated that DCA reliably targets PDK1 and that inhibiting PDK1 is an effective strategy for inhibiting tumor growth by targeting glycolysis and reshaping the tumor microenvironment [15]. Nevertheless, due to the high abundance and rapid synthesis of PDK1, complete inhibition typically requires high‐dose administration, which raises sustainability concerns. Given the limitations of DCA, we explored whether degrading PDK1 could be a more effective approach for cancer therapy.

Proteolysis targeting chimeras (PROTACs) are an emerging technology that focuses on degrading the entire targeted protein, rather than merely occupying active sites [16, 17, 18]. PROTACs offer advantages over traditional small‐molecule inhibitors, such as reduced systemic exposure and reduced drug resistance [19, 20, 21, 22]. Structurally, PROTACs are hetero‐bifunctional small molecules resembling barbells, with E3 ligase and target ligands connected by linkers [18, 23]. Functionally, PROTACs can simultaneously bind to E3 ligase and the protein of interest (POI), forming a stable ternary complex that results in the ubiquitination of the POI and its constant degradation by recruiting the proteasome [24, 25]. Since PROTACs can be released from the ternary complex after degradation and recycled, even trace amounts of PROTAC can achieve long‐lasting degradation [26, 27]. This confers exceptional advantages in protein degradation [20, 25, 28].

**SCHEME 1:**
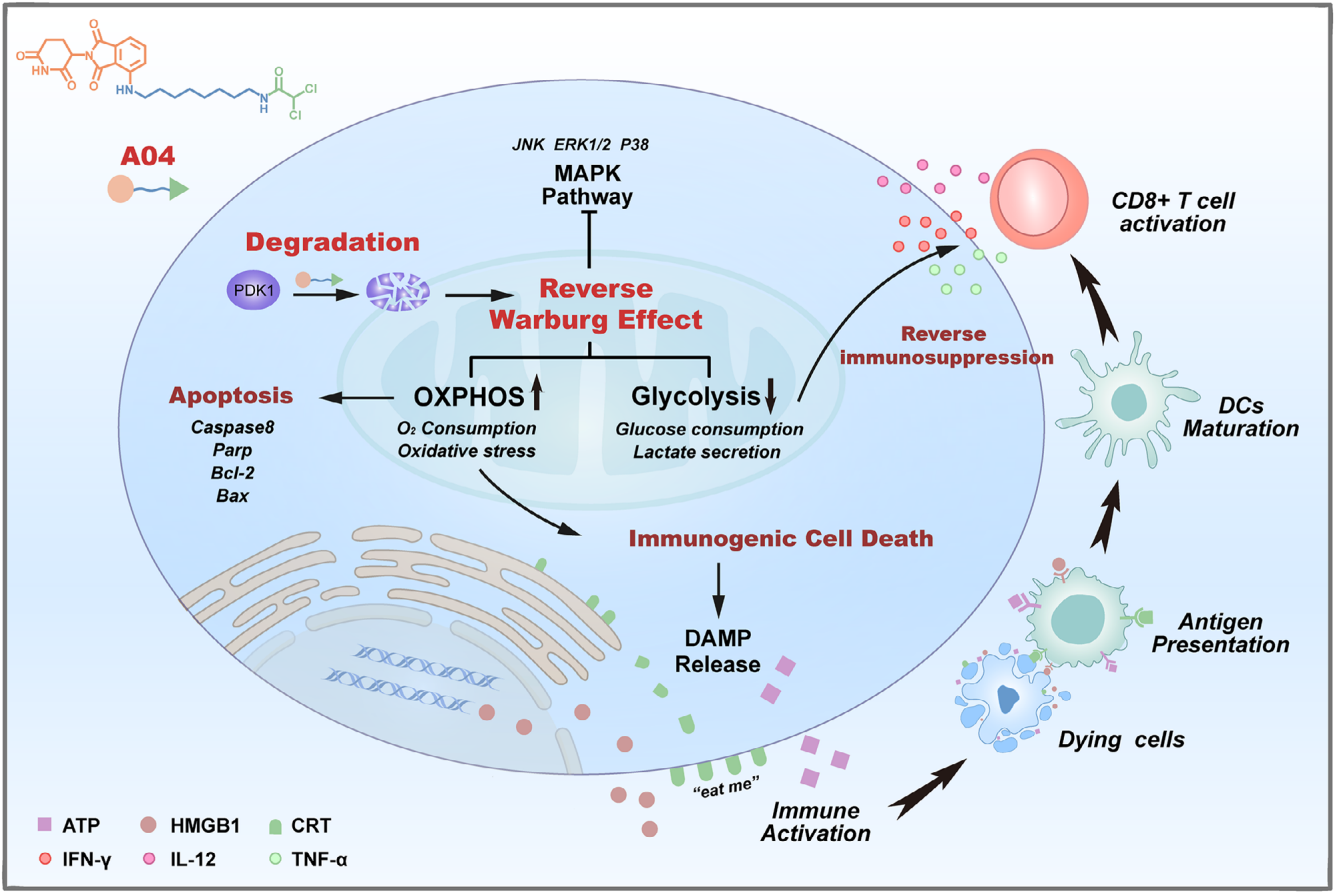
Schematic illustration on mechanism that A04 reverses Warburg effect and enhances immunoreactivity.

In this study, we designed a series of PROTACs to minimize the therapeutic dose and improve the efficacy of DCA in breast cancer treatment. Mechanistically, these PROTACs reduce PDK1 expression and reverse the Warburg Effect in tumor cells, shifting glycolysis to OXPHOS. This has a dual effect: increasing OXPHOS, leading to oxidative stress that induces cell apoptosis and immunogenic cell death (ICD), and reducing extracellular lactate levels, effectively reversing the immunosuppressive characteristics in the tumor microenvironment (Scheme 1). Collectively, we propose a novel approach based on PDK1 degradation for cancer therapy to enhance immunoreactivity.

## 2 Results and Discussion

### 2.1 Design, Synthesis, and Preliminary Biological Evaluation of DCA‐PROTAC

The PROTAC molecule comprises three components: a linker, an E3 ligase ligand, and a POI ligand. To ensure the activity of our designed compounds, we conducted a series of structure‐based analyses. After analyzing the binding mode by examining the co‐crystal structure of DCA and PDK1 (PDB: 2Q8h), we confirmed that the dichloroacetyl group serves as a binding pocket and plays an essential role in interacting with PDK1 (Figure S1A). To maintain targeting ability and ligand affinity, we retained the dichloroacetyl group and constructed a series of PROTACs by introducing linkers at the carboxylic acid position through ester and amide formation (Table 1). Thalidomide is a universal ligand for E3 ligase cereblon (CRBN) and has been successfully applied in multiple PROTACs [29]. Since the imide structure in thalidomide forms hydrogen bonds with CRBN (Figure S1B), linkers can be introduced at the ortho‐ or meta‐position of the aromatic ring in solvent‐exposed regions without disrupting ligand affinity. We fixed the attachment points on both the E3 ligase moiety and the POI recruiting moiety and designed novel compounds based on linker diversity and flexibility.

**TABLE 1:** IC_50_ of compounds.

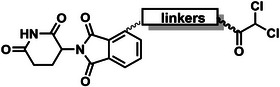									
Compounds	Linkers	IC_50_ Value (µmol/L)^a^				Fold increase^b^			
		4T1	MCF‐7	HGC‐27	786‐O	4T1	MCF‐7	HGC‐27	786‐O
DCA	None	19305.84 ± 196	50208.62 ± 38	14727.55 ± 116	13238.51 ± 44	1	1	1	1
A01	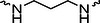	44.30 ± 0.30	104.17 ± 0.78	41.86 ± 1.71	50.56 ± 0.18	436	482	352	262
A02	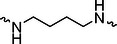	50.21 ± 5.24	70.64 ± 2.93	25.25 ± 0.82	43.30 ± 0.38	385	711	583	306
A03	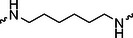	16.80 ± 0.26	94.94 ± 0.23	61.64 ± 2.31	48.69 ± 0.3	1149	529	239	272
A04	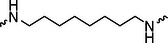	15.11 ± 0.27	57.73 ± 2.47	21.45 ± 0.82	32.86 ± 0.99	1278	870	687	403
A05	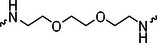	43.82 ± 0.34	92.19 ± 1.92	40.39 ± 2.80	51.18 ± 0.61	441	545	365	259
A06	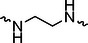	49.29 ± 0.12	165.68 ± 1.36	60.96 ± 1.26	54.77 ± 3.40	392	303	242	242
B01	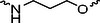	47.86 ± 0.30	102.40 ± 2.18	29.52 ± 2.58	50.61 ± 1.16	403	490	499	262
B02	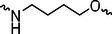	48.95 ± 0.50	71.68 ± 11.45	37.68 ± 0.93	45.20 ± 0.08	394	700	391	293
B03	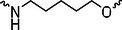	51.09 ± 0.84	110.89 ± 1.56	31.52 ± 2.61	42.75 ± 0.80	378	453	467	310
B04	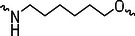	56.62 ± 0.92	83.88 ± 1.67	27.99 ± 1.02	61.38 ± 1.48	341	599	526	216
B05	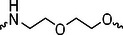	47.09 ± 0.77	82.97 ± 1.99	37.312 ± 1.67	47.49 ± 0.93	410	605	395	279
B06	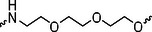	47.03 ± 0.46	60.97 ± 1.69	27.75 ± 1.38	40.81 ± 1.94	411	823	531	324
B07	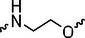	49.38 ± 0.16	206.77 ± 56.99	41.21 ± 0.60	56.05 ± 3.40	391	243	357	236
B08	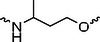	74.12 ± 5.67	65.01 ± 2.71	41.92 ± 1.69	52.23 ± 0.49	260	772	351	253
C01	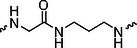	55.89 ± 1.03	52.43 ± 1.41	30.88 ± 1.48	36.83 ± 5.05	345	958	477	359
C02	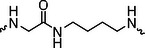	49.99 ± 1.15	45.5 ± 0.42	23.76 ± 3.49	43.79 ± 0.21	386	1103	620	302
C03	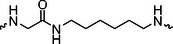	54.55 ± 0.37	48.39 ± 0.94	21.65 ± 1.84	42.88 ± 1.88	354	1038	680	309
D01	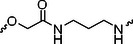	56.46 ± 1.11	72.18 ± 2.78	31.12 ± 0.521	42.23 ± 2.95	342	696	473	313
D02	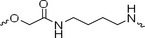	50.80 ± 1.27	82.25 ± 0.08	20.40 ± 0.90	38.39 ± 0.75	380	610	722	345
D03	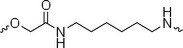	37.91 ± 0.1	126.86 ± 8.18	29.34 ± 0.27	34.75 ± 0.39	509	396	502	381
D04	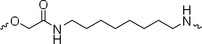	37.04 ± 0.08	44.83 ± 0.72	24.73 ± 0.31	46.40 ± 2.28	521	1120	596	285
D05	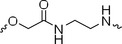	60.94 ± 0.76	50.69 ± 5.95	33.42 ± 0.49	59.03 ± 0.52	317	991	441	224

^a^ IC_50_ values were represented as mean ± standard deviation (SD) of at least three independent assays.

^b^ Fold increase was defined as the ratio of IC_50_ value of DCA / the IC_50_ value of compounds.

We designed and synthesized 22 compounds, characterized their structures using ^1^H‐NMR and ^13^C‐NMR, and confirmed them through mass spectrometry. All compounds achieved purity levels greater than 95%, as verified by high‐performance liquid chromatography. The synthetic routes for these compounds are outlined in Schemes S1–S4.

**FIGURE 1:**
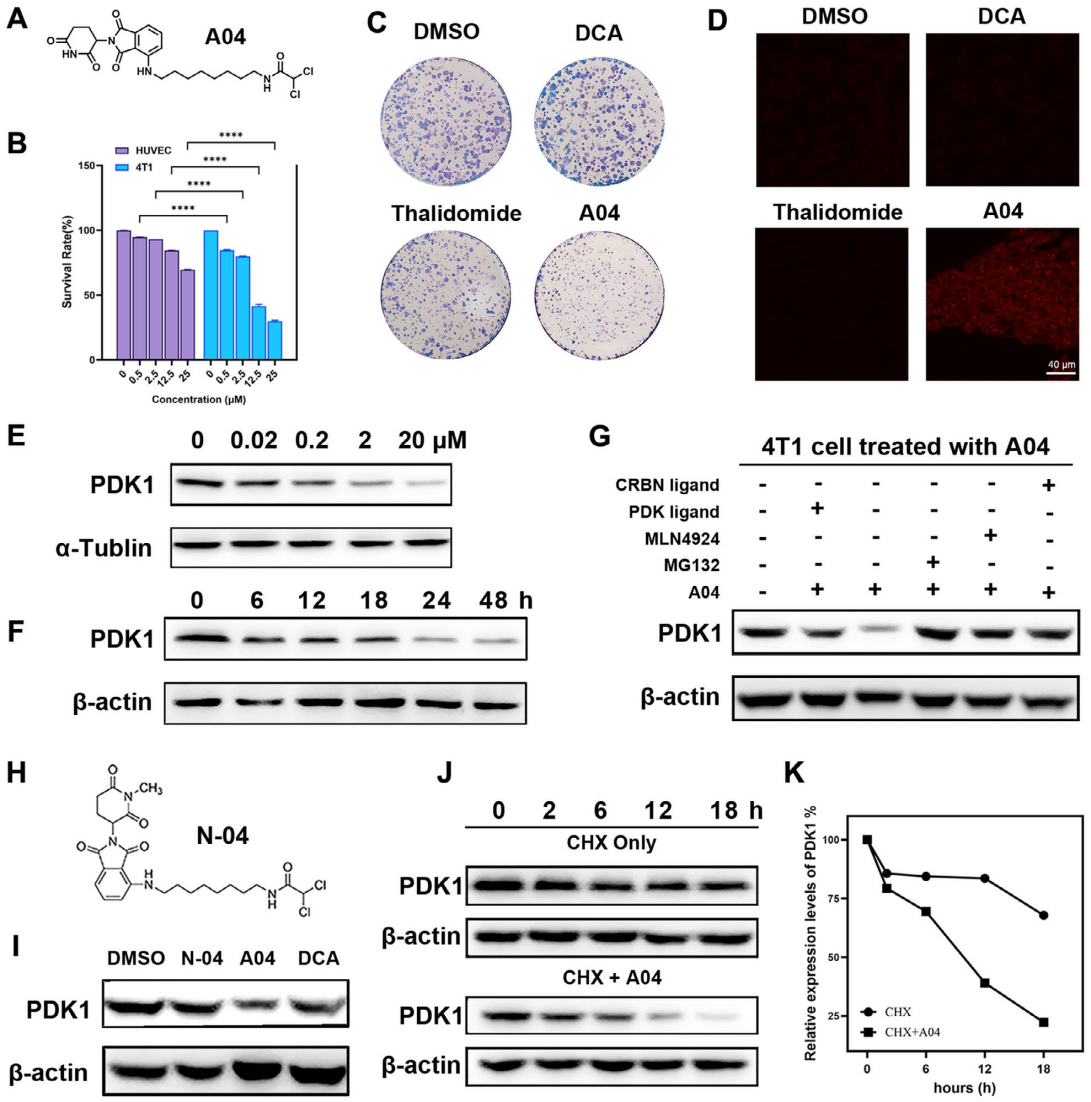
**A04** exhibits excellent cytotoxicity and selectivity on 4T1 cells by degrading PDK1 via UPS. (A) Chemical structure of **A04**. (B) Differential proliferation inhibition between 4T1 and HUVEC response to **A04**. (C) Colony formation in 4T1 cells. (D) CLSM images on dead cells staining by PI. (E) Dose‐dependent degradation on PDK1 of **A04**. (F) Time‐dependent degradation on PDK1 of **A04**. (G) Verification of UPS degradation mechanism. (H) Chemical structure of negative control compound N‐04. (I) Western blot for cells treated with N‐04, **A04**, and DCA. (J,K) CHX chase assay on PDK1.

Next, we evaluated the bioactivity of these compounds in various tumor cell lines through MTT assays, including 4T1, MCF‐7, HGC‐27, and 786‐O (Figure S1C). The fold increase in IC_50_ value between compounds and DCA was calculated. As presented in Table 1, forming amides with DCA (Series‐A) exhibited superior performance compared to esters (Series‐B), possibly due to ester cleavage within cells by enzymatic processes. Among all the compounds we designed, **A04**, featuring an 8‐carbon saturated linker (Figure 1A), performed the best with an IC_50_ value of 15.11 µM, which improved cytotoxicity to 4T1 cells 1277‐fold compared to DCA (IC_50_ = 19.30 mM).

Furthermore, we observed that linkers with longer chains demonstrated more potent antitumor activity than shorter ones, which may be attributed to the need for sufficient spatial distance between the target protein and the E3 ligase. We also evaluated more rigid linkers containing amide groups (Series‐H, E), but no significant improvement was observed. Amphiphilic polyethylene glycol (PEG) linkers were introduced with the expectation of enhanced ductility and flexibility in both hydrophobic and hydrophilic environments. However, compounds with PEG linkers performed worse than those with alkane linkers.

In summary, we synthesized a series of DCA‐PROTACs and conducted preliminary bioactivity assessments. Among these compounds, **A04**, which features an 8‐carbon saturated linker, demonstrated potent antitumor activity, which improved cytotoxicity 1277‐fold compared to DCA.

### 2.2 Selectivity and Cytotoxicity of A04

Due to the heightened sensitivity of the 4T1 mouse breast cancer cell line to **A04** compared to other cancer cell lines, we chose 4T1 as the primary tumor cell model for our subsequent investigations, employing HUVECs as the representative model for normal cells. Because of the high expression of PDK1 in tumor cells (Figure S2A,B), **A04** was able to significantly inhibit tumor cell growth at the same concentration, as shown in Figure 1B.

To corroborate the cytotoxicity of **A04**, a colony formation assay was employed, revealing a substantial reduction in colony numbers when treated with 20 µM of **A04** (Figure 1C). Conversely, no inhibitory effect was observed when 20 µM of either DCA or thalidomide was administered individually. Propidium iodide (PI) is a dye that selectively intercalates into the DNA double strands of dead cells, emitting a bright red fluorescence. PI staining results clearly indicated that **A04** induced significant damage in 4T1 cells at a concentration of 20 µM, while no positive signal was observed when treated with the same dosage of DCA or thalidomide (Figure 1D). Furthermore, we determined the IC_50_ value of thalidomide (THL) and found it to exceed 100 µM in 4T1 cells. To summarize, **A04** exhibited substantial antitumor effects, significantly enhancing the efficacy of DCA, while exerting only minimal effects on normal cells.

### 2.3 A04‐Mediated Degradation of PDK1 Through the Ubiquitin‐Proteasome System (UPS)

Having established the satisfactory cytotoxicity and selectivity of **A04**, we proceeded to assess its degradative capabilities through Western blot analysis. Our findings revealed that **A04** effectively degraded the target protein, PDK1, in a dose‐ and time‐dependent manner (Figure 1E,F and Figure S3A,B). Notably, the half‐maximal degradation concentration (DC_50_) of **A04** was determined to be 1.7 µM, and the maximum degradation ratio (*D*_max_) was achieved at 75% when exposed to a concentration of 20 µM (Figure 1E). PDK1 protein levels exhibited a decline commencing at 2 h post‐**A04** treatment, with maximal degradation occurring at the 24‐h mark (Figure 1F).

Ubiquitination is a pivotal process required to activate the UPS. The tagging of the target protein with ubiquitin is a prerequisite for proteasomes to recognize and initiate the degradation process [30]. To explore the impact of **A04** on ubiquitin‐tagging, we utilized a universal neddylation inhibitor (MLN4924) to counteract the ubiquitin‐tagging effect of **A04**. As depicted in Figure 1G and Figure S3C, the introduction of MLN4924 into the culture medium significantly rescued the protein levels of PDK1 after treatment with **A04**. Notably, the expression of PDK1 remained largely unaltered when cells were pretreated with either the CRBN ligand, thalidomide, or the PDK1 ligand DCA to disrupt ternary complex formation. To further affirm this mechanism, we employed a classical proteasome inhibitor, MG132. As anticipated, the degradation effect of **A04** on 4T1 cells was substantially counteracted by MG132 (Figure 1G).

To provide a negative control for the PROTAC system, we synthesized a compound, N‐04, featuring an N‐methylated group in glutarimide to obstruct hydrogen bond formation between thalidomide and CRBN (Figure 1H and Figure S3D). As anticipated, the treatment of cells with N‐04 resulted in no observable PDK1 degradation, attributed to the failure of ternary complex formation (Figure 1I).

Furthermore, we conducted a cycloheximide (CHX) chase assay to determine the half‐life of PDK1 (Figure 1J and Figure S3E). PDK1 protein levels were monitored via Western blot analysis during the treatment of 4T1 cells with 0.5 µM CHX, both in the presence and absence of **A04**, for 0, 2, 6, 12, and 18 h. As illustrated in Figure 1K, CHX treatment led to a slight reduction (≈25%) in PDK1 levels after 18 h. In contrast, the degradation rate induced by **A04** reached 80%, significantly faster than CHX. These data collectively demonstrate that **A04** reduces the levels of PDK1 through target protein degradation rather than interfering with protein synthesis. Collectively, **A04** induces the time‐ and dose‐dependent degradation of PDK1 via the UPS pathway.

### 2.4 A04 Reverses the Warburg Effect and Induces Apoptosis

**FIGURE 2:**
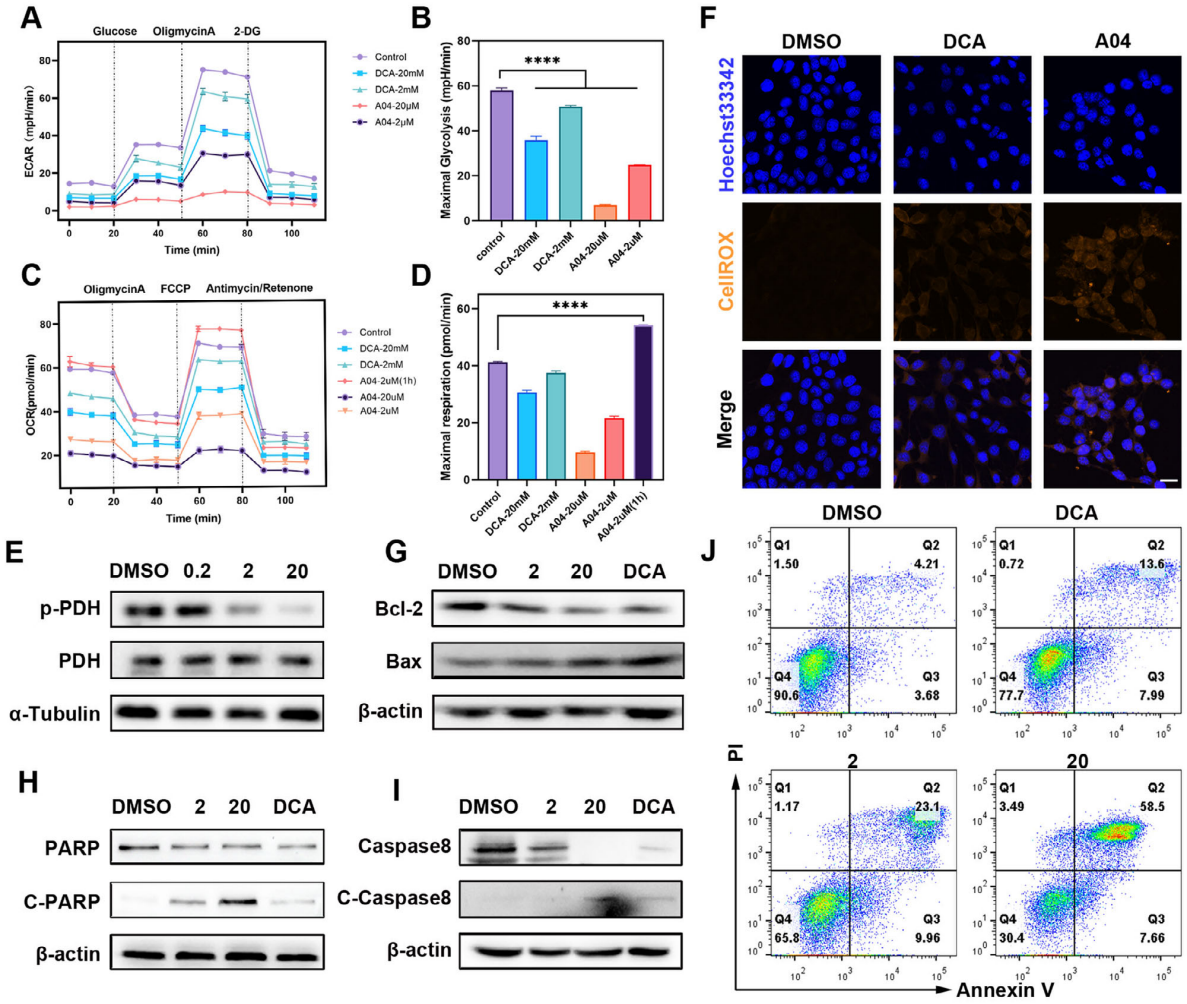
**A04** induces tumor apoptosis by reversing Warburg effect. (A) Determination of ECAR on Seahorse XF24 (*n* = 3). (B) Quantitative analysis of maximal glycolysis. (C) Determination of OCR on Seahorse XF24 (*n* = 3). (D) Quantitative analysis of maximal respiration. (E) Western blot for determination on p‐PDH and PDH expression. (F) ROS detected by CLSM (scale bar: 20 µm). (G) Determinization of proteins with mito‐apoptosis, including Bcl‐2 and Bax. (H,I) Determinization of apoptosis‐related proteins including, PARP, cleaved PARP, caspase 8, and cleaved caspase 8, by Western blot. (J) Flow cytometry analysis of apoptosis in 4T1 cells treated with 20 mM DCA, 2 µM, and 20 µM **A04**.

Having established the effectiveness of **A04** in degrading PDK1, we proceeded to investigate the impact on the energy metabolism phenotype of 4T1 cells. We monitored the extracellular acidification rate (ECAR) using a Seahorse XF24 extracellular flux analyzer (Figure 2A) to assess glycolysis capacity. Cells treated with DCA or **A04** exhibited a reduction in glycolytic features, characterized by lower maximal glycolysis (Figure 2B), basal glycolysis, and decreased spare capacity (Figure S4A,B). Importantly, **A04** at 2 µM demonstrated a glycolysis‐limiting effect comparable to that of DCA at 20 mM, signifying a remarkable 1000‐fold enhancement in glycolysis suppression.

To evaluate the capacity of oxidative phosphorylation (OXPHOS), we conducted an OCR assay. Initially, we applied the same dosing schedule used for ECAR determination, treating cells with the indicated drug concentrations for 4 h. However, cells treated with DCA or **A04** did not exhibit a resurgence of OXPHOS, with decreased basal and maximum respiration (Figure 2C,D). Intriguingly, when we optimized the incubation time and treated 4T1 cells for just 1 h, we observed an increase in maximum respiration (Figure S4C,D). This difference may be attributed to the distinct response dynamics to drug stimuli in 4T1 cells. Furthermore, we assessed the levels of PDH and p‐PDH and found that they were reduced after treatment (Figure 2E and Figure S4E,F), indicating an enhancement of OXPHOS. Intracellular lactate concentrations were also measured and found to be decreased (Figure S4G), further substantiating the restriction of glycolysis.

Given **A04**’s potent reversal of the Warburg effect, we investigated ROS accumulation and mitochondrial apoptosis. We detected ROS levels using confocal laser scanning microscopy (CLSM) and assessed proteins related to mitochondrial apoptosis through Western blot. **A04** significantly elevated ROS levels in 4T1 cells compared to the control group (Figure 2F). Simultaneously, immunofluorescence of JC‐1 demonstrated a significant decrease in mitochondrial membrane potential in 4T1 cells after **A04** treatment (Figure S4H). And we also observed the downregulation of the anti‐apoptotic protein BCL‐2 and the upregulation of the pro‐apoptotic protein BAX (Figure 2G and Figure S4I,J).

To gain further insights into the mechanism of apoptosis, we examined the expression of apoptosis‐related proteins via Western blot. Activation of the caspase family is a crucial indicator of apoptosis. We observed the appearance of cleaved caspase 8 and a decrease in the expression of full‐length caspase 8 (Figure 2I and Figure S4K,L). Furthermore, a significant reduction in full‐length PARP and an increase in cleaved‐PARP demonstrated the robust transduction of apoptotic signals (Figure 2H and Figure S4M,N). To precisely measure the apoptosis rate in 4T1 cells, we applied a gradient dose of **A04** and performed flow cytometry. As a positive control, 4T1 cells treated with 20 mM DCA were included (Figure 2J). The apoptosis rate was 23.1% when treated with 2 µM **A04**, and it dramatically increased to 58.5% with 20 µM (Figure S4O). Collectively, these findings confirm that **A04** induces apoptosis by reversing the Warburg effect.

### 2.5 Transcriptome Analysis of mRNA in 4T1 Cells

**FIGURE 3:**
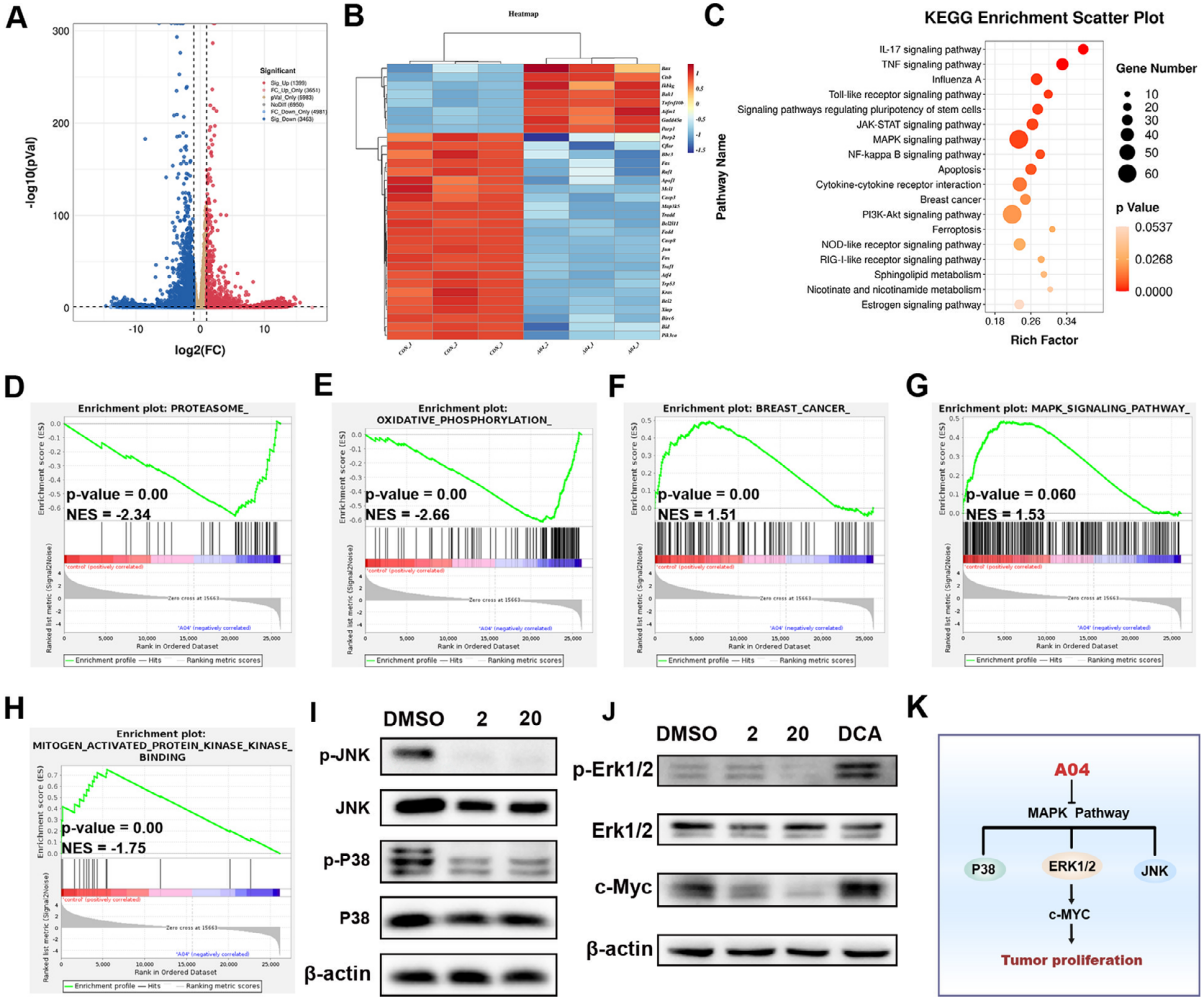
Transcriptome analysis of differentially expressed genes in 4T1 cells after treatment. (A) Volcano plot of differential genes. (B) Heat map of differential genes related with apoptosis. (C) KEGG enrichment plot for differential genes. (D–H) GSEA results of significant pathways including proteasome, oxphos phosphorylation, breast cancer, MAPK, and mitogen activated protein kinase (*n* = 3). (I,J) Verification of mitogen activated protein kinases expression by western blot in 4T1 cells treated with DMSO, DCA (10 mM), 2 and 20 µM **A04**. (K) Schematic illustration of the potential mechanism on transcriptional regulation from **A04**.

Given that **A04** functions as a post‐translational degrader of proteins, we embarked on exploring its potential extra‐transcriptional effects in 4T1 cells. Following transcriptome analysis, we observed a significant alteration in multiple signal pathways within the genes of 4T1 cells. In the treatment group, there were 1399 significantly upregulated genes and 3463 downregulated genes compared to the DMSO control (Figure 3A). Notably, we found a comprehensive change in genes associated with the apoptosis pathway, aligning with previous findings (Figure 3B). Kyoto Encyclopedia of Genes and Genomes (KEGG) enrichment analysis further underscored the substantial modifications in the apoptosis signaling pathway. Additionally, differentially expressed genes were notably enriched in various cancer‐related signaling pathways, including the Toll‐like receptor signaling pathway, JAK‐STAT signaling pathway, and MAPK signaling pathway (Figure 3C).

Gene set enrichment analysis (GSEA), which ranks genes based on the extent of their expression changes, revealed significant enrichment of genes related to the proteasome pathway in the treatment group. This finding further substantiates the ubiquitin‐proteasome system (UPS)‐mediated degradation mechanism of PROTAC (Figure 3D). Genes linked to the oxidative phosphorylation (OXPHOS) signaling pathway were also closely associated with the treatment group (Figure 3E), confirming the metabolic reprogramming that occurs after PDK1 degradation in 4T1 cells. Moreover, multiple gene sets associated with cancer, such as the breast cancer pathway (Figure 3F) and the NF‐kappa B signaling pathway (Figure S5A), were downregulated to varying degrees. This suggests that the treatment may contribute to a non‐cancerous gene expression signature in 4T1 cells.

In line with the results of the KEGG enrichment analysis, GSEA demonstrated a significant alteration in the MAPK signaling pathway (Figure 3G,H). Given the well‐established role of the MAPK pathway in multiple phosphorylation signal transduction in breast cancer [31], we explored its potential relevance to the anti‐tumor effect of **A04**. Western blot analysis was conducted to evaluate the levels of classical mitogen‐activated protein kinases, including P38, ERK1/2, and JNK (Figure 3I,J). It was evident that **A04** significantly downregulated the phosphorylation levels of p‐P38, p‐ERK1/2, and p‐JNK. Additionally, we noted a substantial decrease in the oncogenic transcriptional factor c‐myc in the heatmap (Figure S5B). Given that ERK1/2 can participate in and mediate the transcription and translation of c‐myc, we assessed the protein levels of c‐myc and found them to be downregulated (Figure 3J). C‐myc is a classical marker of malignant tumors and is widely implicated in tumor proliferation and metabolism [32, 33, 34].

Our study on transcriptome analysis revealed significant changes in gene expression, with enrichment in various pathways after treatment with **A04**. Beyond validating known mechanisms involving metabolic reprogramming and the UPS system, which provide robust evidence for our primary strategy, we also uncovered additional transcriptional changes, particularly in the MAPK signaling pathway (Figure 3K). All of these findings inspire us to explore the therapeutic effects of **A04** in vivo.

### 2.6 Evaluation of Anti‐Tumor Effect and Tolerance of A04 In Vivo

**FIGURE 4:**
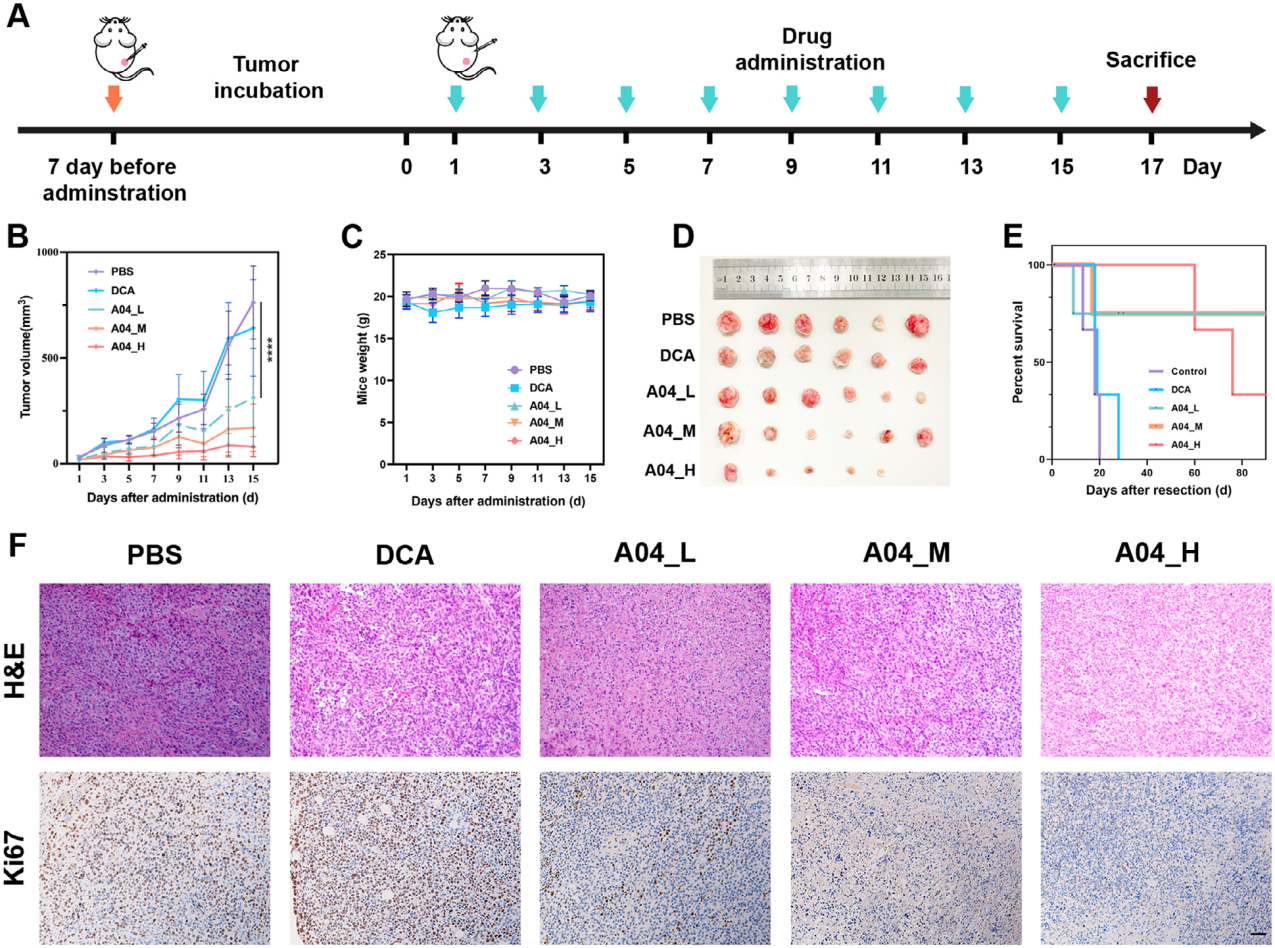
Therapeutic effect of **A04** in 4T1‐bearing Balb/c mice. (A) Administration scheme. (B) Tumor growth curve. (C) Mice weight. (D) Image of tumor, yellow circle indicates a vanished tumor. (E) Survival rate after tumor resection. (F) H&E staining and IHC staining with proliferation marker Ki67 of tumor (Scale bar: 50 µm). Data are shown as mean ± SD (*n* = 6). Data are expressed as the mean ± SD. ****P < 0.0001.

To validate the anti‐tumor effects in vivo, we established a breast cancer model by subcutaneously inoculating 4T1 cancer cells into Balb/c mice. In line with the in vitro results, intraperitoneal administration of **A04** once every alternate day displayed a dose‐dependent anti‐tumor activity (Figure 4A,B). Notably, the treatment with 10 mg/kg **A04** led to a significant anti‐tumor effect, outperforming DCA at 40 mg/kg. And the mice exhibited minimal body weight loss, indicating the excellent tolerance of **A04** (Figure 4C). H&E staining of major organs (Figure S6A) and routine analysis of blood in mice (Figure S6B–J) indicated that the compound **A04** did not produce any toxic side effects during treatment. Treatment with 20 and 40 mg/kg **A04** resulted in remarkable and long‐lasting tumor regression (Figure 4D).

We also investigated the impact on prognosis following treatment. Three mice from each group underwent surgery to remove the in situ carcinoma, and their health was continuously monitored. As shown in Figure 4E, **A04** treatment effectively extended their survival, while mice in the PBS group and the DCA‐treated group succumbed shortly after tumor resection. Autopsy results revealed the presence of tumor metastatic nodules in the lungs (Figure S6K), which may explain the demise of these mice. As for the deaths in the **A04**_H (high dose) group, we speculate that the high‐dose treatment might have caused systemic toxicity in the mice, as these deaths occurred 2 months later.

Furthermore, we verified the pharmaceutical mechanism in vivo by assessing the degradation effect on PDK1 in tumor tissue using Western blot. The results demonstrated a significant reduction in PDK1 protein levels after treatment with **A04** compared to the PBS‐administered group (Figure S6L). Furthermore, we also measured the lactic acid content of the tumor tissues, as shown in Figure S6M, which revealed that the PROTAC **A04** was effective in reducing lactic acid accumulation and inhibiting aerobic glycolysis in mouse tumor tissues. Histopathological evaluation revealed a substantial proportion of damaged cancer cells with characteristic condensed and fragmented nuclei, as indicated by hematoxylin and eosin (H&E) staining in the treatment group. Additionally, there was a remarkable decrease in Ki67, a key marker of cell proliferation, in the tumor tissue, as evidenced by immunohistochemistry (IHC) assays (Figure 4F). The above experiment proved that **A04** is a potent and safe anti‐tumor agent with significant efficacy and minimal side effects in vivo.

### 2.7 A04 Activates Immune Response by Inducing ICD

**FIGURE 5:**
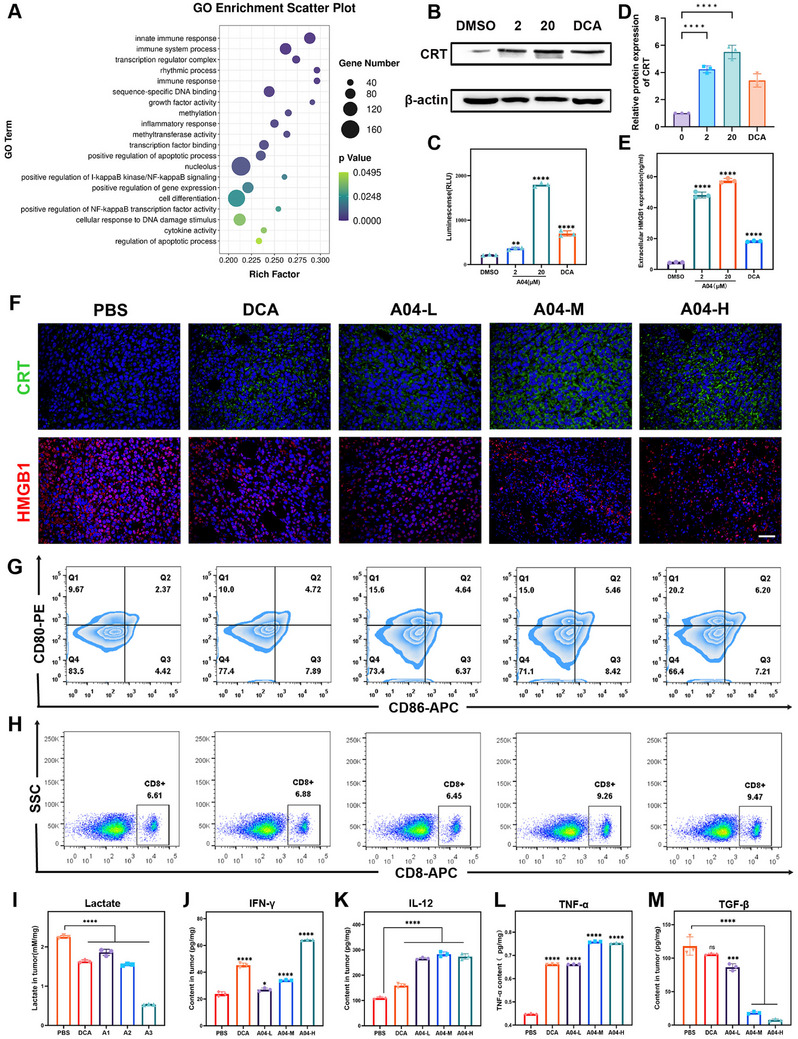
**A04** activates the immune response by ICD and reverses immune suppression in the TME. (A) GO enrichment analysis of differentially expressed genes in 4T1 cells treated with 20 µM **A04**. (B,C) CRT protein expression in 4T1 cells after treatment with DMSO, DCA (20 mM), 2 µM, and 20 µM **A04**. (D) Luminescence assay of ATP content. (E) HMGB1 content determined by ELISA. (F) IF staining of CRT and HMGB1 in tumor after treatment (Scale bar: 50 µm). (G,H) Flow cytometry analysis of DCs and CD8^+^ T cells in spleen. (I–M) Content of lactate, IFN‐γ, IL‐12, TNF‐α, TGF‐β in tumor (*n* = 3). Data are expressed as the mean ± SD. *P < 0.05, **P < 0. 01, ***P < 0.001, ****P < 0.0001.

Based on the results of gene ontology (GO) enrichment analysis, it is evident that treatment with **A04** significantly stimulates a range of immune responses in 4T1 cells (Figure 5A). Notably, GO terms related to innate immune response, immune system processes, and immune responses demonstrated pronounced changes. In the process of ranking *P*‐values and selecting the top 20 genes (Figure S5B), we identified a gene involved in antitumor immunity, calreticulin (CRT), which stood out in the heatmap. Subsequently, we conducted Western blot analysis to determine the expression of CRT in 4T1 cells after treatment with **A04**. As anticipated, the level of CRT was upregulated (Figure 5B,C), emphasizing the significance of the increased CRT expression and prompting further investigations.

Immunogenic cell death (ICD), a distinctive form of apoptosis triggered by oxidative stress, converts non‐immunogenic cells into immunogenic cells by releasing damaged antigen molecular patterns (DAMPs), including ATP and high mobility group box 1 (HMGB1) [35]. Given the strong association of CRT with ICD [36], we assessed changes in DAMPs. We quantified extracellular ATP using bioluminescence and measured secreted HMGB1 with an enzyme‐linked immunosorbent assay (ELISA) (Figure 5D,E). Intriguingly, both ATP and HMGB1 exhibited a significant increase in 4T1 cells, confirming the occurrence of ICD in vitro.

To further validate the induction of ICD in vivo, we evaluated the expression of CRT and HMGB1 through immunofluorescence (IF) staining in tumor slices. The location and fluorescence intensity of CRT and HMGB1 displayed notable differences between the **A04**‐treated group and the PBS group. As shown in Figure 5F, in the **A04**‐treated group, CRT shifted from the cytoplasm to the cell membrane, with much higher fluorescence intensity compared to the PBS group. Similarly, HMGB1 in the PBS group was primarily concentrated in the nuclei, but after **A04** treatment, the fluorescence dimmed, and HMGB1 was mainly distributed in the cytoplasm (Figure 5F). The exposure of CRT signals “eat me,” promoting the recognition of dead‐cell antigens by dendritic cells (DCs) [36]. The release of HMGB1 contributes to antigen presentation to T lymphocytes [35]. The increase in CRT and HMGB1 suggests the initiation of a potent immune response, which led us to investigate immune activation in vivo.

Moreover, we also evaluated dendritic cell (DC) maturation and T lymphocyte activation in the spleen by flow cytometry. As demonstrated in Figure 5G and Figure S7A, the percentage of mature DCs, marked by CD80 and CD86, increased from 2.37% to 6.20%, indicating that **A04** enhances antigen presentation by DCs. CD8^+^ T cells play a crucial role in the infiltration of solid tumors by responding to the antigen‐presented signal from mature DCs. Flow cytometry results showed a marked increase in the percentage of CD8^+^ T cells in the spleen (Figure 5H and Figure S7B), indicating complete immune activation in the periphery. In addition, flow cytometry analysis of tumor tissues demonstrated that compound **A04** not only enhances the immune competence of the lymphatic system but also improves the tumor immunosuppressive microenvironment, with a marked increase in the percentage of both matured dendritic cells (CD80^+^CD86^+^)and CD8^+^T cells in the tumor (Figure S7C–F).

Herein, we concluded that **A04** induces ICD both in vitro and in vivo, characterized by the translocation of CRT from the endoplasmic reticulum to the cell membrane and the release of HMGB1 from the nuclei to the cytoplasm. This process initiates an immune response by promoting DC maturation and the activation of CD8^+^ T lymphocytes.

### 2.8 A04 Reverses Tumor Immunosuppressive Microenvironment

Immunosuppression in the TME is predominantly driven by high lactate concentration [37]. **A04** is anticipated to enhance immunoreactivity in the TME by reversing the Warburg effect [38]. Given that **A04** decreased lactate at the cellular level, we proceeded to assess the lactate content within the tumor, which indeed exhibited a significant reduction (Figure 5I). GSEA analysis also unveiled a significant downregulation of genes enriched in the regulation of pH pathway in the treatment group (*p* = 0.0001, NES = 1.69), providing further evidence of the reversal of an acidic TME following treatment (Figure S8A).

To further validate the impact of **A04** on immune reactivity in the TME, we measured cytokines associated with immunity through ELISA. In comparison to the PBS group, **A04**‐treated mice displayed a substantial increase in IFN‐γ and TNF‐α (Figure 5J,K), indicating an enhancement of CD8^+^ T cell function. Furthermore, tumor tissue with Granzyme B IHC analysis and perforin IF analysis, as shown in Figure S8E, showed that both of the above CD8^+^T cytokines increased significantly after **A04** treatment, which was able to further demonstrate that **A04** has a potent immune‐activating ability. Similarly, IL‐12, a pro‐inflammatory cytokine that contributes to IFN‐γ secretion, was also elevated with **A04** treatment (Figure 5L). Furthermore, the levels of transforming growth factor‐beta (TGF‐β) were significantly reduced (Figure 5M), which is beneficial for reversing immune suppression since TGF‐β can inhibit the proliferation and differentiation of T lymphocytes.

Transcriptome data corroborated the influence of **A04** treatment on immune reactivity. Genes related to TGF‐β (Figure S8B) were significantly downregulated in the treatment group (*p* = 0.0021, NES = 1.56), consistent with the cytokine measurement results. Additionally, GSEA results revealed a significant enrichment of genes associated with antigen processing and cross‐presentation (Figure S8C,D) in the treatment group (FDR; *q* = 0.00049, NES = −2.17).

To conclude, **A04** reverses immunosuppression in the TME by reducing lactate content and modulating multiple immune cytokine secretion.

### 2.9 Combination With αPD‐L1 Enhances Anti‐Tumor Reactivity of A04

Tumors often upregulate the expression of PD‐L1 to interact with PD‐1 on T cells, thereby impairing the function of CD8^+^ T cells and evading anti‐tumor immune responses [39, 40]. Immune checkpoint inhibitors targeting PD‐L1, such as αPD‐L1, have been employed in various cancer therapeutic strategies [41, 42]. In an effort to amplify the immunoreactivity of **A04**, we explored the combination of **A04** with the immune checkpoint inhibitor αPD‐L1 to enhance anti‐tumor therapy.

**FIGURE 6:**
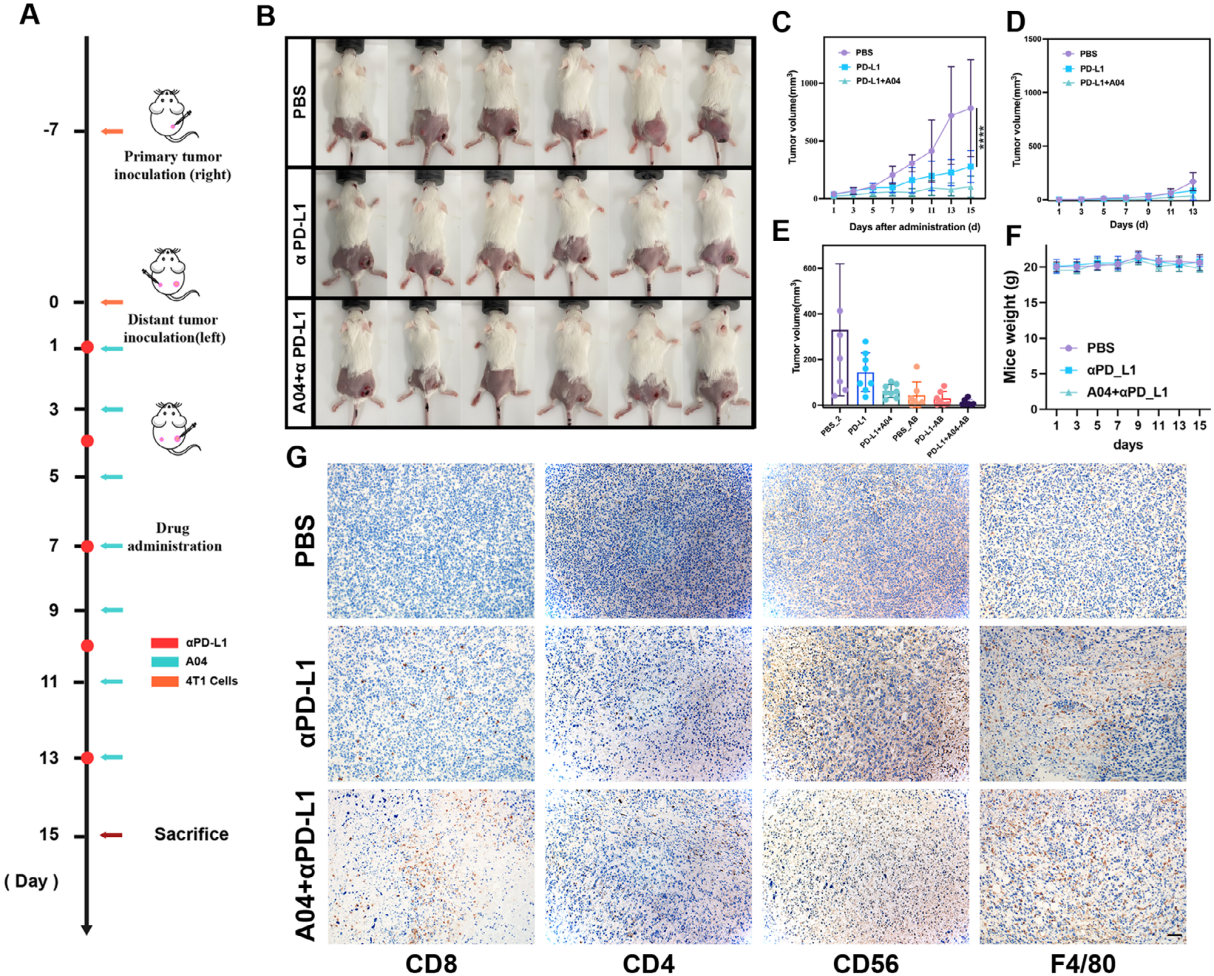
Combination with αPD‐L1 augments immune‐reactivity of **A04** and raises multiple immune infiltration. (A) Administration scheme of combination therapy. (B) Image of mice with bilateral tumor after 15 days of treatment. (C) Volume of tumor in the primary site. (D) Volume of tumor in the distant site. (E) Tumor volume. (F) Mice weight. (G) IHC staining of distant tumor (Scale bar: 50 µm). *n* = 6, ****P < 0.0001.

To assess the systemic immune effects of this combination, we established a bilateral tumor model. Subcutaneous tumors were initiated on both sides of the dorsal flank in female Balb/c mice. Initially, 4T1 cells were inoculated on the right side of the flank, and after 7 days, they were also seeded on the left side (Figure 6A). **A04** was administered peri‐tumorally at a dose of 20 mg/kg twice a day, while αPD‐L1 was given once every 3 days at a dosage of 40 µg per mouse. Comparing the combination therapy with single‐agent administration, the combination of **A04** and αPD‐L1 led to a significant increase in anti‐tumor effects, resulting in remarkable regression of tumor growth at both the primary and distant sites (Figure 6B–E). The weight of the mice in the combination group exhibited only a minor and negligible decrease (Figure 6F).

Furthermore, we assessed the infiltration of immune cells in tumor tissue using immunohistochemistry (IHC). In line with our observation that **A04** can activate an immune response, combination therapy markedly enhanced the infiltration of immune‐activating CD4^+^ T helper cells and CD8^+^ tumor‐killing T cells (Figure 6G). Additionally, a larger presence of positive signals for CD56 and F4/80 indicated that combination therapy also increased the infiltration of natural killer (NK) cells and macrophages in tumor tissue. These findings collectively demonstrate that the combination of **A04** and αPD‐L1 augments anti‐tumor effects by increasing immune cell infiltration within the tumor, leading to an enhanced anti‐tumor immune response.

## 3 Conclusion

Metabolic enzymes are characterized by high abundance and rapid synthesis, making traditional metabolic enzyme inhibitors less viable for clinical use due to the need for large administration doses and the risk of systemic cytotoxicity [13]. PROTAC technology represents an emerging approach that leverages the cell's intrinsic ubiquitin‐proteasome system to degrade oncogenic target proteins, and it has shown great promise in cancer therapy [43, 44, 45, 46, 47, 48].

In this study, we harnessed PROTAC technology to target the aberrantly expressed metabolic enzyme PDK1 in cancer cells, creating degrader compounds based on the classical PDK inhibitor DCA. DCA, as an allosteric inhibitor, binds to a specific pocket to deactivate PDK1, but typically requires millimolar concentrations for efficacy. Our application of PROTAC technology overcomes this challenge.

We designed a total of 22 PDK1 degrader compounds and identified the top‐performing compound, **A04**, which demonstrates long‐lasting PDK1 degradation and potent anti‐tumor activity. Among all the compounds tested, **A04** stands out by enhancing PDK1 degradation and proliferation inhibition efficacy in vitro by a factor of 1000‐fold. Furthermore, **A04** exhibits robust tumor regression in mice bearing 4T1 tumors and extends the lifespans of these mice following tumor resection.

In terms of mechanisms, **A04** reverses the Warburg effect by shifting cellular metabolism from glycolysis to OXPHOS. This shift to overactivated OXPHOS leads to the generation of massive (ROS, inducing tumor apoptosis and ICD within the tumor microenvironment. The release of CRT, HMGB1, and ATP contributes to an immune response, resulting in increased DCs and CD8^+^ T cells in the spleen. Additionally, **A04** limits lactate secretion and regulates multiple immune cytokines, including IL‐12, IFN‐γ, and TNF‐α. These findings confirm that glycolysis inhibition disrupts the immunosuppressive microenvironment within tumors.

Moreover, transcriptome analysis validates these mechanisms at the cellular level and uncovers changes in multiple cancer‐related signaling pathways, with a particular focus on the MAPK pathway. The combination of **A04** with the immune checkpoint inhibitor αPD‐L1 enhances therapy efficacy and promotes the infiltration of various immune cells, including natural killer (NK) cells, macrophages, and CD8^+^ T cells in distant tumors.

Although we have successfully introduced an efficient PDK1‐degrading PROTAC for the first time, challenges remain in the optimization of the binding efficiency between DCA and PDK1. Therefore, future research efforts are expected to concentrate on identifying more potent PDK1 inhibitors and advancing the development of highly effective PDK1 degraders. We believe potent PDK1‐PROTACs could contribute to tumor therapy and immunotherapy by glycolysis inhibition, which drives innovation in cancer therapy and provides promising avenues for future treatments.

In summary, our study has pioneered a novel therapeutic approach centered on PDK1 degradation rather than enzymatic activity inhibition, achieving significant success in cancer immunotherapy. This approach holds great promise for the development of innovative cancer treatments.

## Conflicts of Interest

The authors declare no conflicts of interest.

## Supporting information



Supplementary Materials

## Data Availability

The data that supports the findings of this study are available in the supplementary material of this article.
