# Supplementary material for: Innovative PDK1‐Degrading PROTACs Transform Cancer Aerobic Glycolysis and Induce Immunogenic Cell Death in Breast Cancer
Source: Exploration (Beijing). 2025 May 7;5(4):e20240031. doi: 10.1002/EXP.20240031 (PMC12380065; doi:10.1002/EXP.20240031)
Supplement: Supplementary file 1 — Supplementary Materials [file EXP2-5-e20240031-s001.pdf]

Supplementary Information

Title: Innovative PDK1-Degrading PROTACs Transform Cancer Aerobic Glycolysis and Induce Immunogenic Cell Death in Breast Cancer

Aohua Deng[1, 2], Renming Fan[1, 2], Jiakui Gou[1, 2], Ruoxi Sang[1, 2], Ruizhuo Lin[1, 2], Ting Zhao[1, 2], Junyan Zhuang[1, 2], Yongrui Hai[1, 2], Jialin Sun[3], Gaofei Wei[1, 2*]

[1]Institute of Medical Research, Northwestern Polytechnical University, Xi'an, Shaanxi, 710072, China.

[2]Research & Development Institute of Northwestern Polytechnical University in Shenzhen, Guangdong, 518057, China.

[3]Edinburgh Kidney Research Group, Centre for Cardiovascular Science, Queen's Medical Research Institute, University of Edinburgh, Edinburgh EH16 4TJ, United Kingdom.

Aohua Deng, Renming Fan, Jiakui Gou, and Ruoxi Sang contributed equally to this work.

***Corresponding authors:**

Prof. Gaofei Wei

Xi'an Key Laboratory of Stem Cell and Regenerative Medicine, Institute of Medical Research, Northwestern Polytechnical University

Xi'an, Shaanxi, 710072, China.

E-mail: weigf0605@163.com

Material and methods

Synthesis material and equipment

All commercial reagents obtained from Energy-Chemical, Adamas-Beta, or Topbiochem were used without purification unless otherwise specified. Flash-column chromatography was realized with 200−300 mesh silica gel (Qingdao Haiyang Chemical, China). HPLC chromatograms were obtained on a Waters Alliance e2695 with a reverse-phase column (C18, Agilent) (150 mm × 4.6 mm) using a mixture of solvent acetonitrile /water (70:30) at the flow rate of 1.0 ml/min and peak detection at 254 nm under UV, injection volume of 10 μL, and injection time of 10 min. ^{1}H and ^{13}C NMR spectra were recorded on a Bruker ARX 600 MHz spectrometer: chemical shifts and coupling constants (J) were showed in parts per million and in hertz, respectively. HRMS were measured on a Bruker microTOF_Q spectrometer. Supporting Information described the synthetic routes and characterization data.

Cell culture and viability assay

4T1 and MCF-7 cells were purchased from the Laboratory Animal Center of Sun Yat-sen University (Guangzhou, China). 786-O, HGC-27 and Human Umbilical Vein Endothelial Cells (HUVECs) cells were purchased from Cell Bank of Chinese Academy of Sciences, China. The cells were cultivated in RPMI-1640 or DMEM supplemented with 1% (w/v) penicillin (100 U/ml) /streptomycin (100 μg/ml) and 10% (v/v) FBS having 5% CO_2 at 37 °C. Cell viability was assayed by MTT method (Beyotime Biotechnology, ST316). Briefly, cells were seeded into 96-well plates with 5000 cells per well and cultured in a 37 ºC, 5% CO_2 incubator (Thermo Fisher, Forma 371). Overnight, gradient concentration of compounds in cultural medium was incubated for 72 h. Subsequently, 200 μL fresh medium containing MTT solutions with final concentration at 5 mg/ml was added to cells and incubated for 4 h. Absorbance at 490 nm was measured using a microplate reader (Tecan Spark).

Colony formation assay.

Cells were seeded in a six-well plate with 10^5 cells per well and cultured in a 37ºC, 5% CO_2 incubator. After 24 h treatment of 20 μM **A04**, thalidomide， DCA, and DMSO, cells were incubated with fresh medium and cultured for one week. When the colony formed, cells were fixed with methanol and stained with Giemsa solution (Beyotime Biotechnology, C0133). Images were photographed by digital camera.

PI dead cell staining and ROS detection.

Cell death was determined by PI staining method (ThermoFischer scientific, P1304MP). ROS was stained by CellROX Deep Red (ThermoFischer scientific, C10448). 4T1 cells were seeded into 12-well plate and cultured in the condition with 37ºC, 5% CO_2. After 24 h treatment, cells in plate were washed and stained with 1 μM PI or 10 μM ROS in the PBS buffer for 20min at 37 ºC. Cell nuclei was stained with Hoechst 33342(Beyotime, C1025). The staining cells were fixed with 4% Paraformaldehyde (PFA) and observed by CLSM (OLYMPUS FV3000). Images captured by CLSM were disposed with FV31S-SW Viewer.

Protein extraction and western blot.

Cells were lysed in RIPA lysis buffer (Beyotime Biotechnology, P0013) containing protease inhibitor (Beyotime Biotechnology, ST505) and phosphatase inhibitors (Beyotime Biotechnology, P1081) on ice for 10 min. After centrifugation at 14,000g for 12 min at 4°C, the supernatants were gently collected and quantified using a BCA assay Kit (Beyotime Biotechnology, P0012). The sample was denatured at 95°C with loading buffer containing DTT and SDS. The protein samples were subjected to 8%-12% sodium dodecyl sulfate- polyacrylamide gel electrophoresis (SDS-PAGE) and were transferred to PVDF membranes in Bio-Rad system. After blocking by TBST containing 5% skim milk for 1 h, the membrane was incubated overnight at 4°C with multiple primary antibodies (1:1000-1:5000). After incubation with HRP-conjugated secondary antibodies (1:5000) for 1 h at room temperature, the signals were captured and visualized by Chemiluminescence Imaging System (VILBER-VILBER, FUSION FX6.EDGE) and analyzed using the Evolution Capt Edge (Vilber).

OCR/ECAR measurement.

Glycolytic capacity and mitochondrial OXPHOS level of 4T1 cells after treatment were determined by using Seahorse analyzer referring to manufacturer's instructions. In brief, 5×10^4 cells were seeded in Seahorse plates and treated with **A04** and DCA at indicated concentrations for 1 h or 4 h, then washed in Seahorse buffer. To measure the OCR, Seahorse buffer containing each of 1 μM oligomycin, 1 μM FCCP and 1 μM rotenone was added at certain moment. To determine the ECAR, 10 mM glucose, 1 μM oligomycin and 100 mM 2-DG in Seahorse buffer were consequently injected into the analyzer.

Lactate and ATP assay.

Lactate production was measured by colorimetry using a Lactate Assay Kit (Nanjing jiancheng bioengineering institute, A019-2-1). For intracellular lactate determination, cells (2×10^6) were lysed and centrifuged at 15000 g for 10 min. Next, 100 μL of the supernatants was mixed with 100 μL of the reaction mix. For extracellular lactate determination, 100 μL serum-free medium was added instantly to100 μL reaction mix. The reaction was incubated at 37 °C for 10 min and terminated by stop solution. Lactate levels were measured at 530 nm using a microplate reader. For measurement of ATP levels, extracellular ATP in medium was determined by ATP assay kit (S0027, Beyotime) according to standard protocol. The final luminescence level was determined by a microplate reader (Tecan Spark)

Apoptosis rate determination.

Annexin V/ FITC Apoptosis Detection Kit (DOJINDO LABORATORISE) was used to analyze tumor cell apoptosis rate after treating with **A04**. In brief, 4T1 cells were seeded in six-well plates and treated with different concentration of **A04** and DCA for indicated time. Then, cells were collected and stained with AnnexinV-FITC and PI for 10 minutes and determined by flow cytometry. Positive control was set for adjusting parameter and compensation.

mRNA Sequencing and Analysis.

mRNA Sequencing and Analysis: In this study, the 4T1 cell samples ($>10^7$ cells) were collected for mRNA sequencing and analysis after 24 h-treated with PBS and 20μM **A04**. The RNA libraries were sequenced on the illumina Novaseq™ 6000 platform by LC Bio Technology CO.,Ltd (Hangzhou, China). Bioinformatic analysis was performed using the OmicStudio tools at https://www.omicstudio.cn/tool.

Enzyme-linked immunosorbent Assay (ELISA).

ELISA assay kits were purchased from Thermo Fischer scientific. According to protocol, 100 μL cell culture medium was added into pro-coated microplate. Then, multiple antibody was added for detection, and the plate was incubated in 4°C overnight. Plate was washed with buffer before adding HRP-conjugated streptavidin and TMB substrate. Plate were read at OD 450 nm. Protein concentration was determined by BCA assay for correction.

***In vivo* antitumor efficacy.**

Balb/c mice (6–8 weeks old) were purchased from SPF (Beijing) Biotechnology Co., Ltd. All

experiments involving animals were conducted according to the ethical policies and procedures approved by the ethics committee of the Northwestern Polytechnical University. (Ethical Number: NWPU202201057) All animals were bred in the pathogen-free facility with a 12 h light/dark cycle and relative humidity (40–70%) at 21 ± 2 °C. All the mice had access to food and water ad libitum. The tumor-bearing mouse models were established by inoculating 4T1 cells (100 µL, concentration of 1×10^6) in PBS into the right flank of the Balb/c mice. Once the tumor volume reached about 50 mm^3, five groups of the tumor-bearing mice were intravenously injected with saline,40 mg/kg DCA, and **A04** at 10mg/kg, 20mg/ kg and 40mg/kg (n = 6). The tumor volumes and body weights of mice were measured every 2 days. The tumor volume (V) was calculated by using the equation: V=L*S*S/2, where L is the longest dimension of tumor, and S is the shortest dimension of tumor.

Quantification of DCs and lymphocytes in spleen.

4T1 cell-bearing mice were intravenously injected with same formulation twice a day. After 17 days, mice were euthanatized and spleens were harvest and grind into mono-dispersed cell suspension. After using red blood cell lysis buffer, DCs and lymphocytes were collected for staining. Anti-CD11c-FITC, anti-CD80-PE and anti-CD86-APC were used to mark matured DCs ($CD11c^+$ $CD80^+$ $CD86^+$), 7AAD was employed to exclude dead cells. Anti-CD3-PE and anti-CD8-APC were applied in distinguishing $CD8^+$ cytotoxic T cells ($CD3^+CD8^+$). Samples were analyzed by Flow cytometry and graph by FlowJo_V10.

H&E, IHC, and IF studies.

All specimens used for staining were fixed in 4% PFA and embedded in paraffin. The paraffin mass was cut into 4-µm sections that were mounted on slides. For the H&E assay, the slides were stained with hematoxylin for nuclei and eosin for cytoplasm. For IHC staining, slides were blocked by 5% bovine serum albumin and incubated with Ki67, CD4, CD8, CD56, and F4/80 antibody. Afterwards The slides were then washed and incubated with HRP-polymer-conjugated secondary antibody and counterstained with hematoxylin. Sides for IF were blocked and incubated with CRT and HMGB1 antibody, then stained with FITC or Cy3 conjugated Goat Anti-mouse IgG. H&E, IHC, and IF slides were imaged by using a microscope (BX53, Olympus) with CellSens Entry software.

Antibody and dilution.

The supplier and dilution of antibodies used in this manuscript are listed in Supporting Information

Table S1.

Statistical analysis.

The statistical analysis was performed using the GraphPad Prism 8 by the Student's t-test and one-way analysis of variance (ANOVA). The data were expressed as means ± standard deviation (SD).

Supplementary scheme

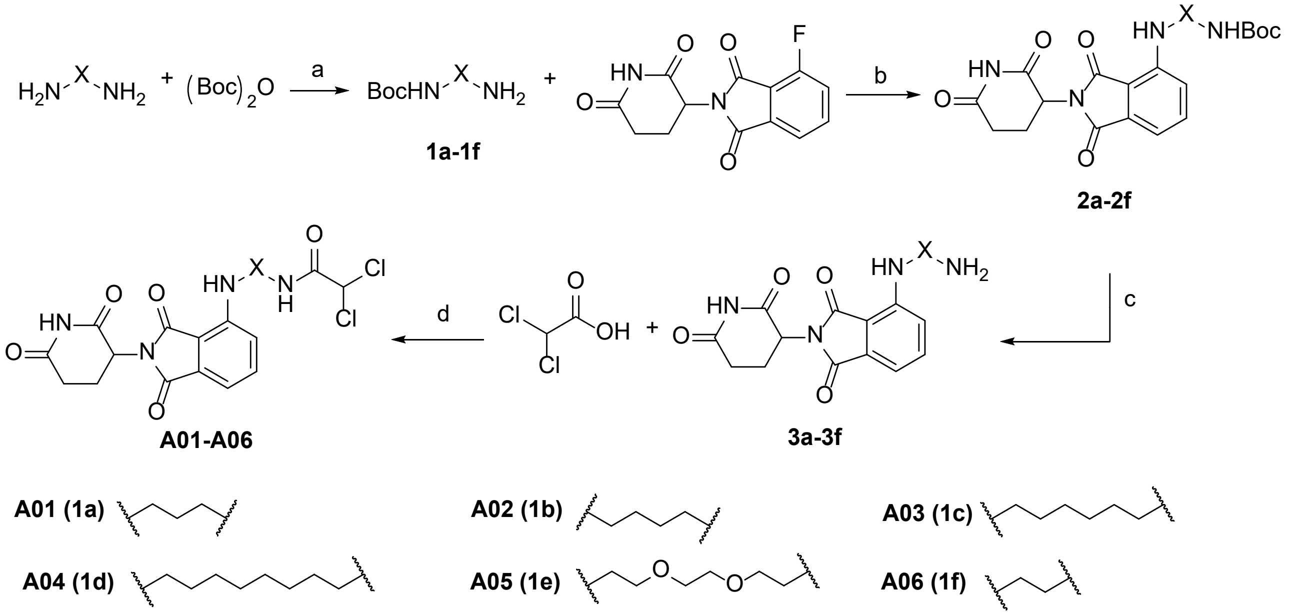

Scheme S1. Synthesis of Series A derivatives. (a) TEA, DCM; (b) DIPEA, 80 °C; (c) TFA, DCM; (d) DCA, DIPEA, HATU, DMF.

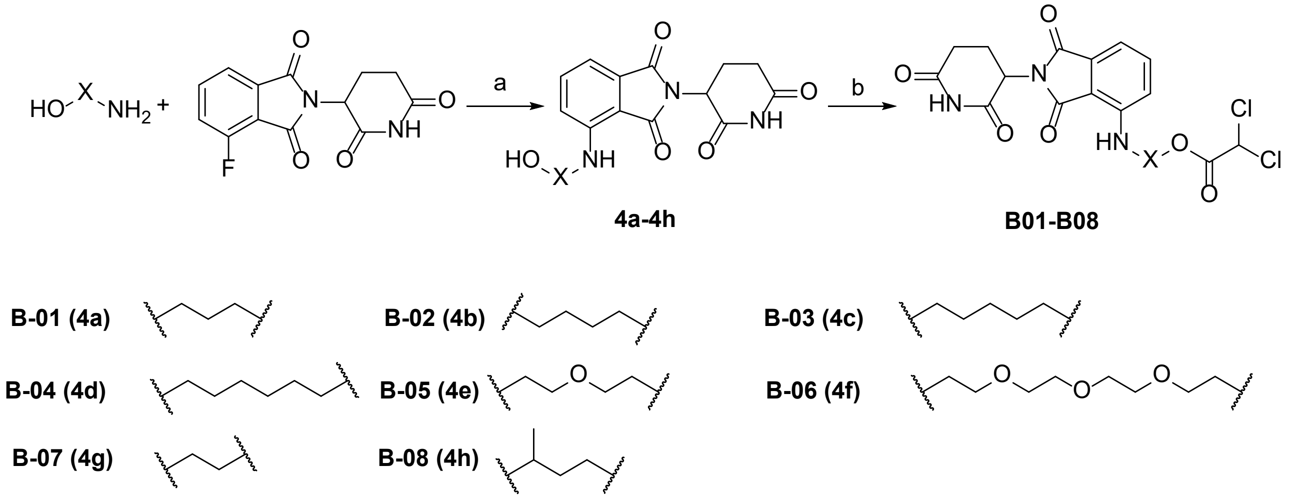

Scheme S2. Synthesis of Series B derivatives. (a) DIPEA, EA, 80 °C; (b) DCA, Dichloroacetyl chloride, TEA, DCM.

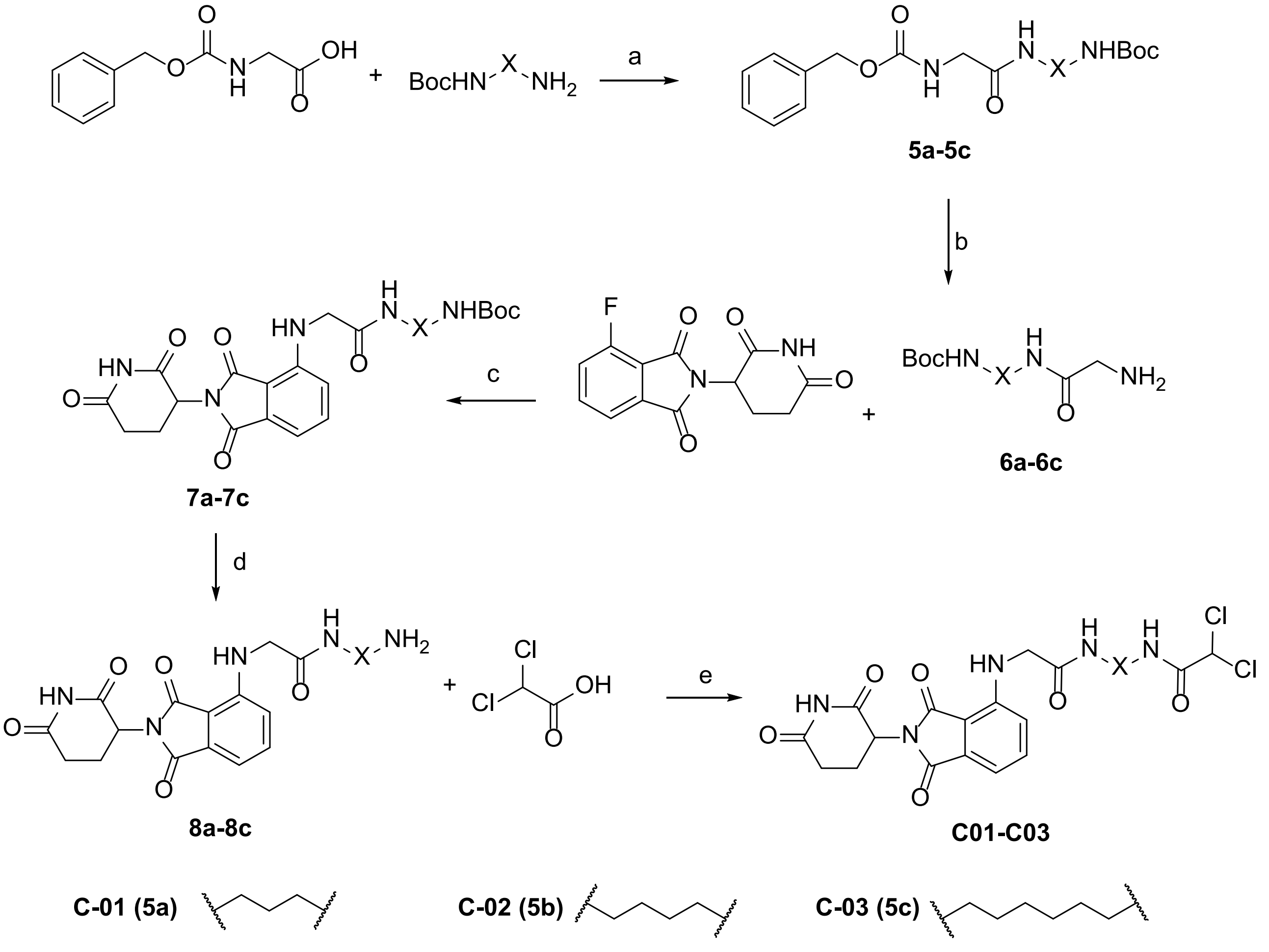

Scheme S3. Synthesis of Series C derivatives. (a) DIPEA, HATU, DMF; (b) Pd/C, H_2, CH3OH; (c) DIPEA, 80 °C, EA; (d) TFA, DCM; (e) DCA, DIPEA, HATU, DMF.

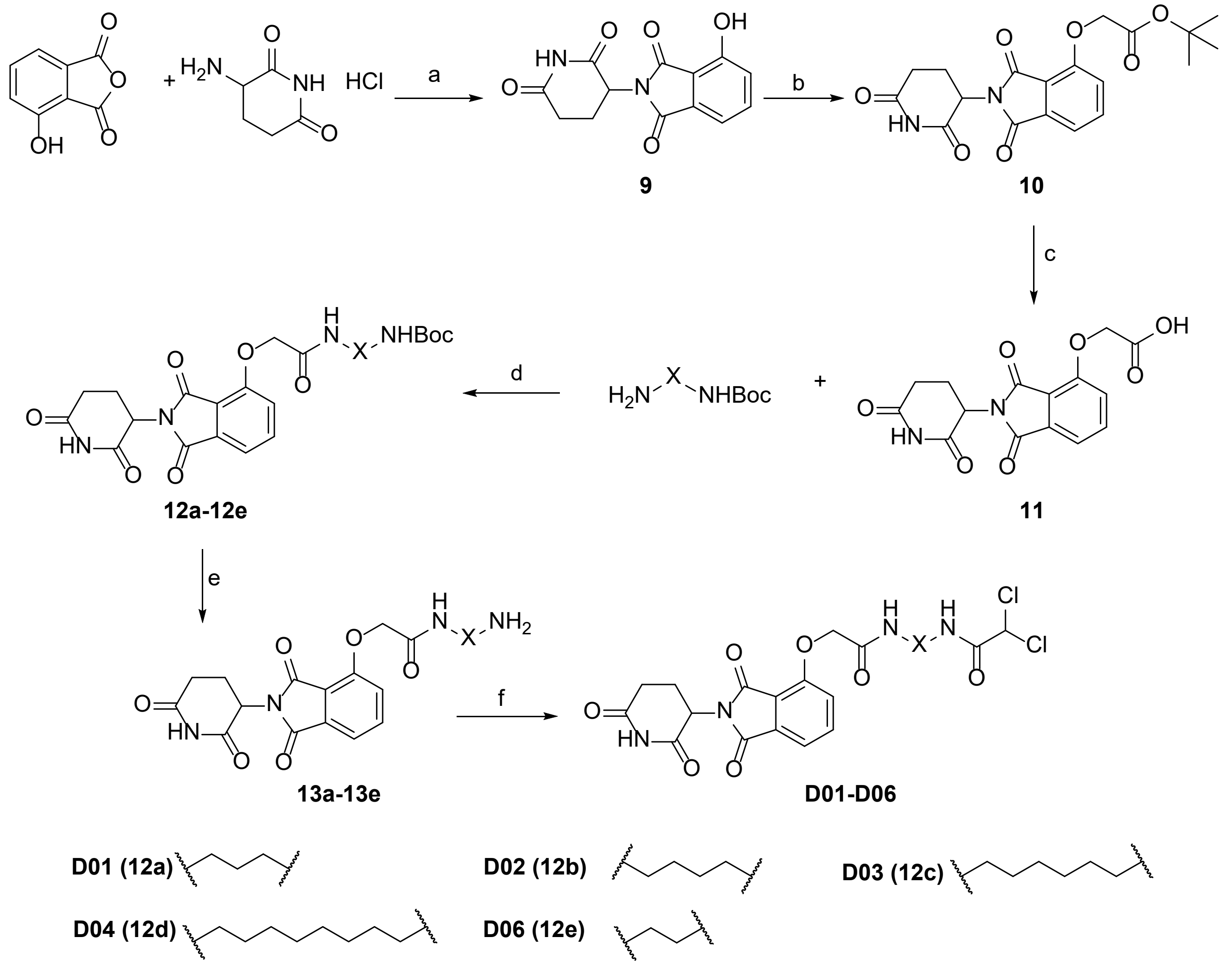

Scheme S4. Synthesis of Series D derivatives. (a) DMAP, DCC, THF, 60 °C; (b) tert-butyl 2-bromoacetate, $KHCO_3$, DMF, 60 °C; (c) TFA, DCM; (d) DIPEA, HATU, DMF; (e) TFA, DCM; (f) DCA, DIPEA, HATU, DMF.

Supplementary figures

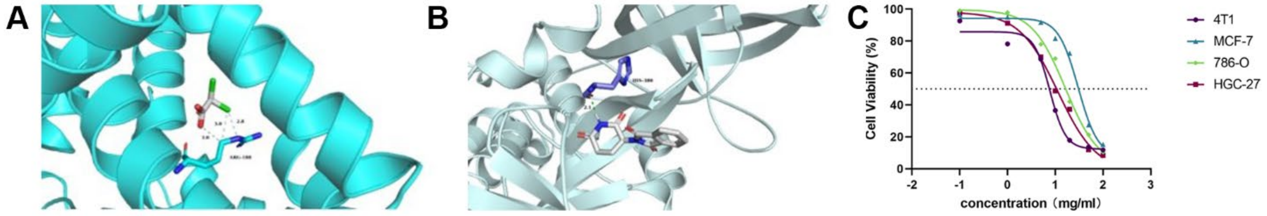

Figure S1. Design, Synthesis, DCA-PROTAC. A, Binding mode between DCA and PDK1 (pdb:2Q8h). B, Binding mode between thalidomide and CRBN (pdb:4ci3). C, The IC_{50} curve of compound A04 in 4T1, MCF-7, HGC-27 and 786-O.

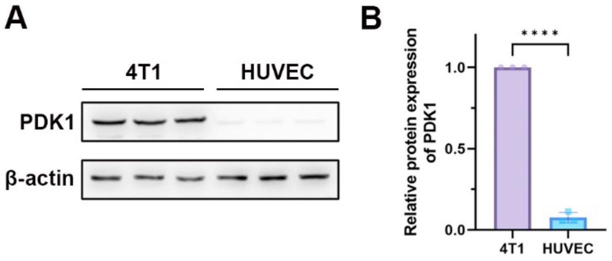

Figure S2. PDK1 protein expression in 4T1 and HUVEC. A, Western blot for PDK1 in 4T1 and HUVEC. B, Statistical analysis for PDK1 in 4T1 and HUVEC. (n=3).

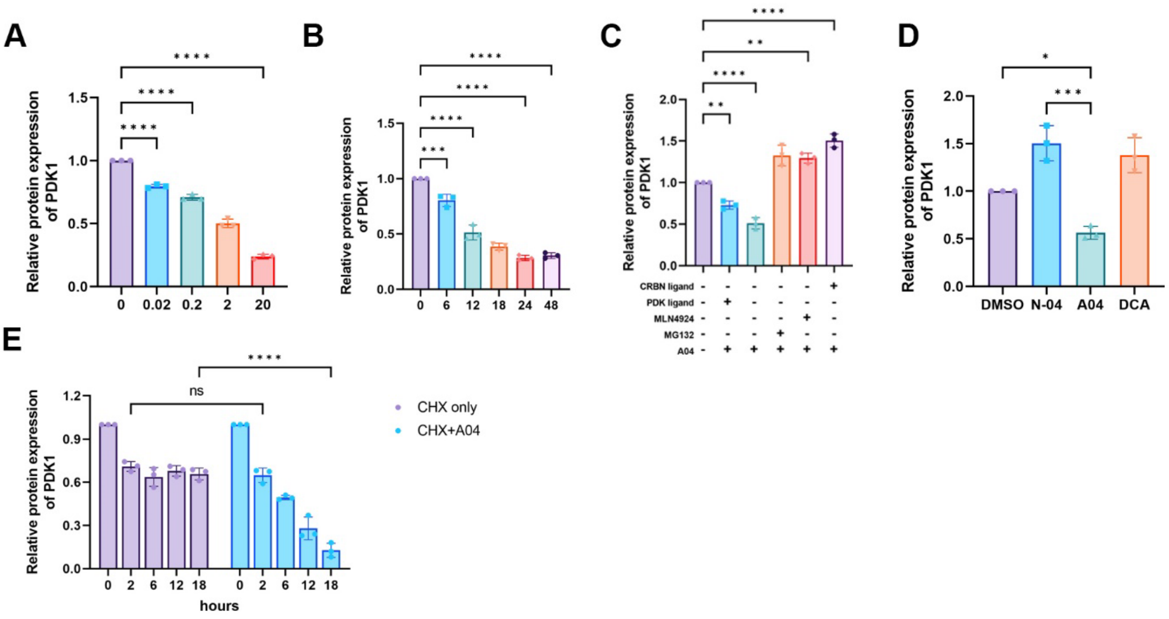

Figure S3. A04 exhibits excellent cytotoxicity and selectivity on 4T1 cells by degrading PDK1 via UPS. A, Statistical analysis for dose-dependent degradation on PDK1. (n=3). B, Statistical analysis for time-dependent degradation on PDK1. (n=3). C, Statistical analysis for verification of UPS degradation mechanism. (n=3). D, Statistical analysis for cells treated with N-04, A04 and DCA.

(n=3). E, Statistical analysis for CHX chase assay on PDK1. (n=3).

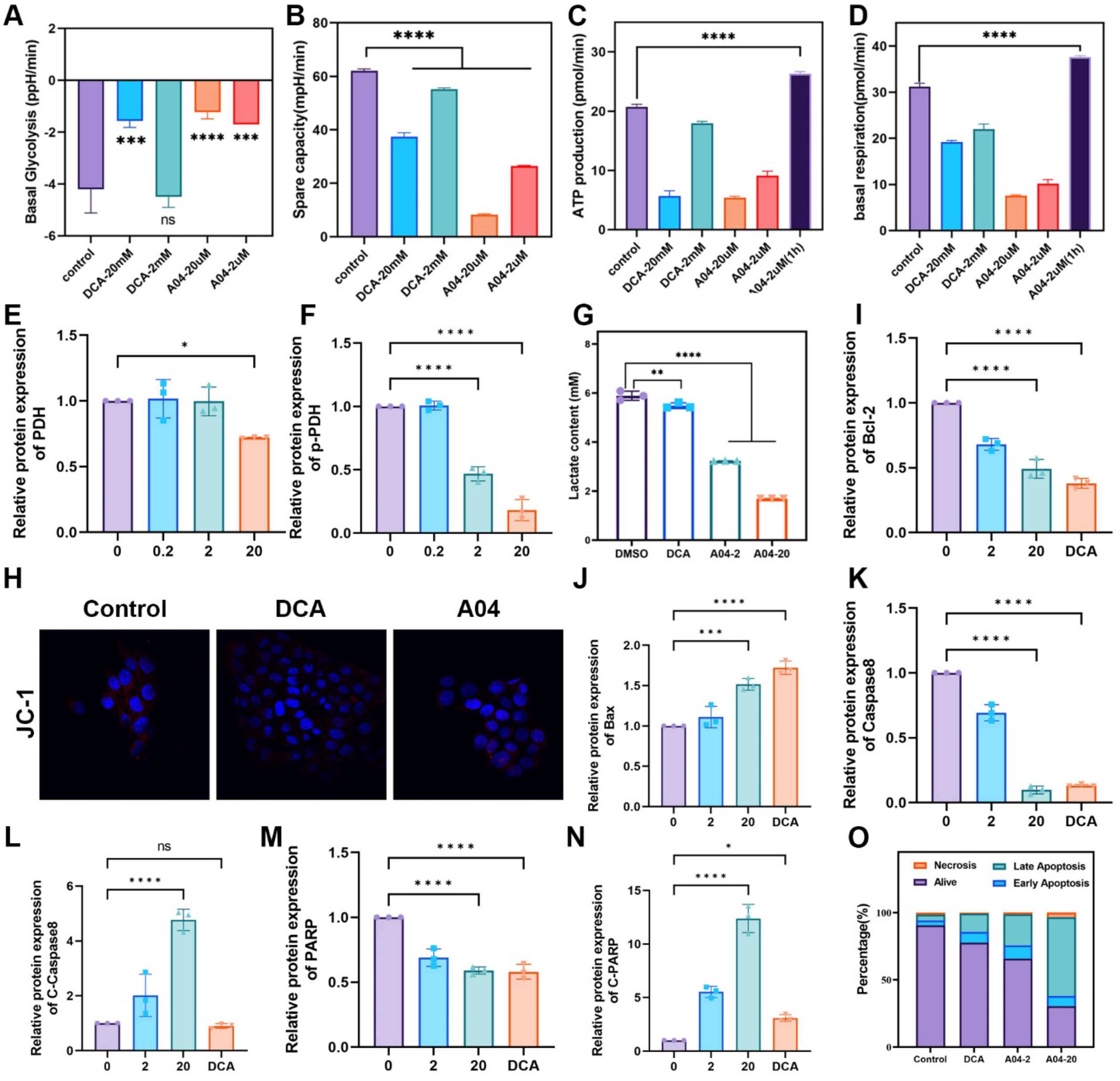

Figure S4. A04 Reverses the Warburg Effect and Induces Apoptosis. A-B, Basal glycolysis and spare capacity of cells after treatment with DMSO, 2 mM and 20 mM DCA, 2 μM and 20 μM A04. C-D, ATP and basal respiration of cells after treatment with DMSO, 2 mM and 20 mM DCA, 2 μM and 20 μM A04. E-F, Statistical analysis for PDH and p-PDH. (n=3). G, Lactate content in 4T1 cells after treatment with 2 and 20 μM A04 and 20 mM DCA. H, Confocal images of mitochondrial membrane potential detected by JC-1 probe. I-J, Statistical analysis for Bcl-2 and Bax. (n=3). K-L, Statistical analysis for Caspase8 and Cleaved-Caspase8. (n=3). M-N, Statistical analysis for PARP and Cleaved-PARP. (n=3). O, Statistical analysis of apoptosis experiments.

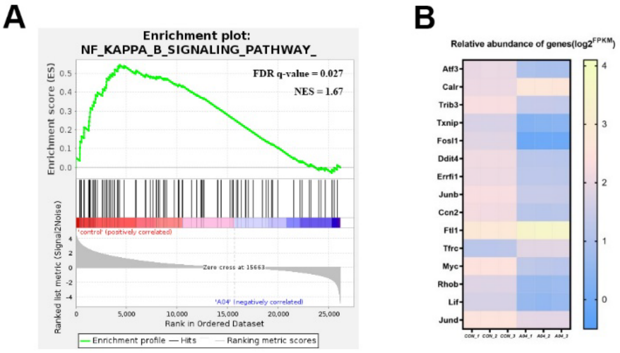

Figure S5. Transcriptome Analysis of mRNA in 4T1 Cells. A, GSEA on NF_KAPPA_B_SIGNAL PATHWAY (control VS A04). B, Relative abundance of genes in top 15 ranking by P value (decrease from top to bottom).

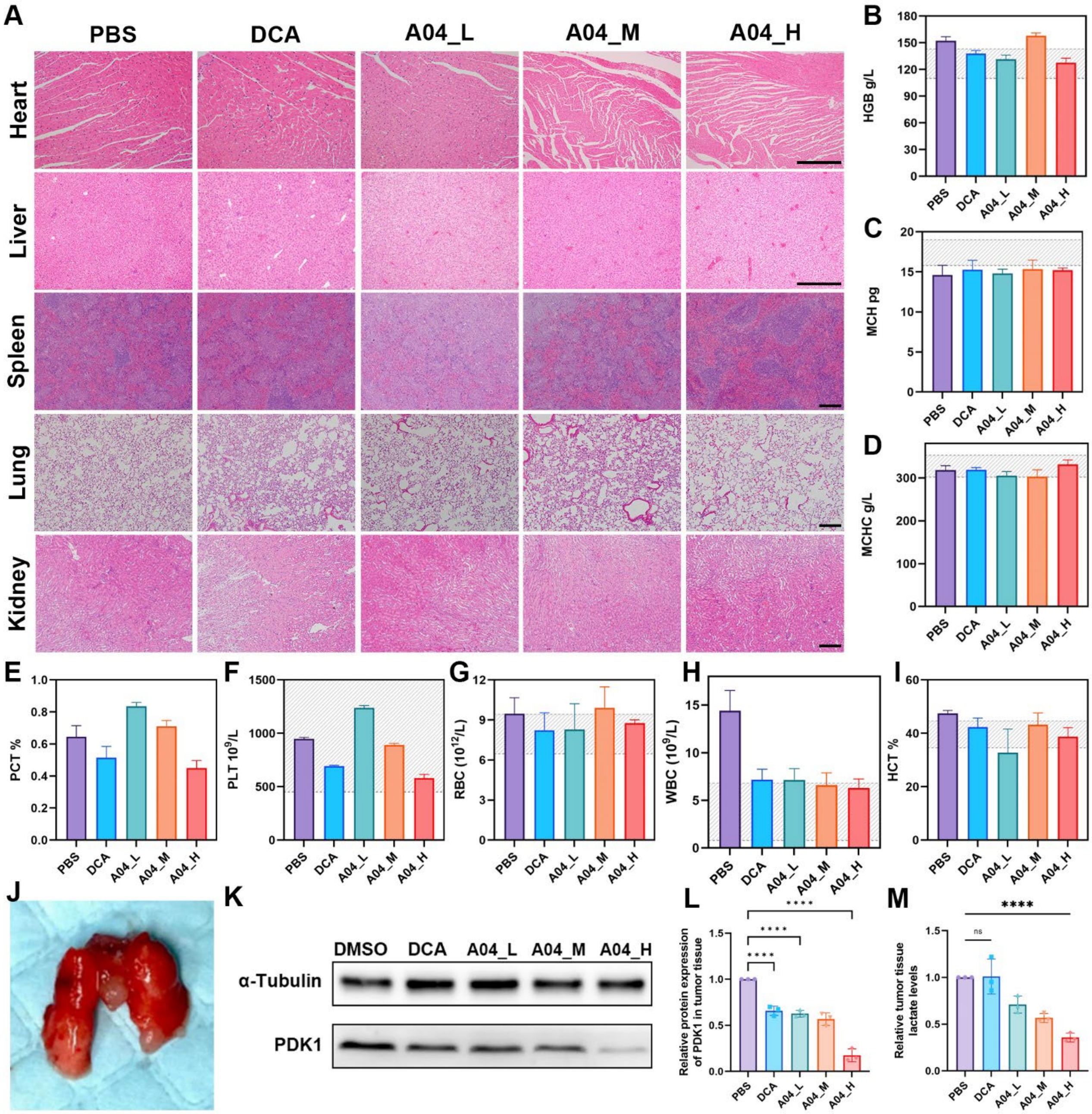

Figure S6. Evaluation of Anti-Tumor Effect and Tolerance of A04 In Vivo. A, H&E staining images of major organs. Scale bar = 100 μm. B-I, Histogram of statistical analyses of routine analysis of blood in mice (n=3). J, Lung nodes in 4T1-bearing mouse. K-L, Western blot and statistical analysis of PDK1 in tumor. (n=3). M, Statistical analysis of the lactate content of tumor tissues. (n=3).

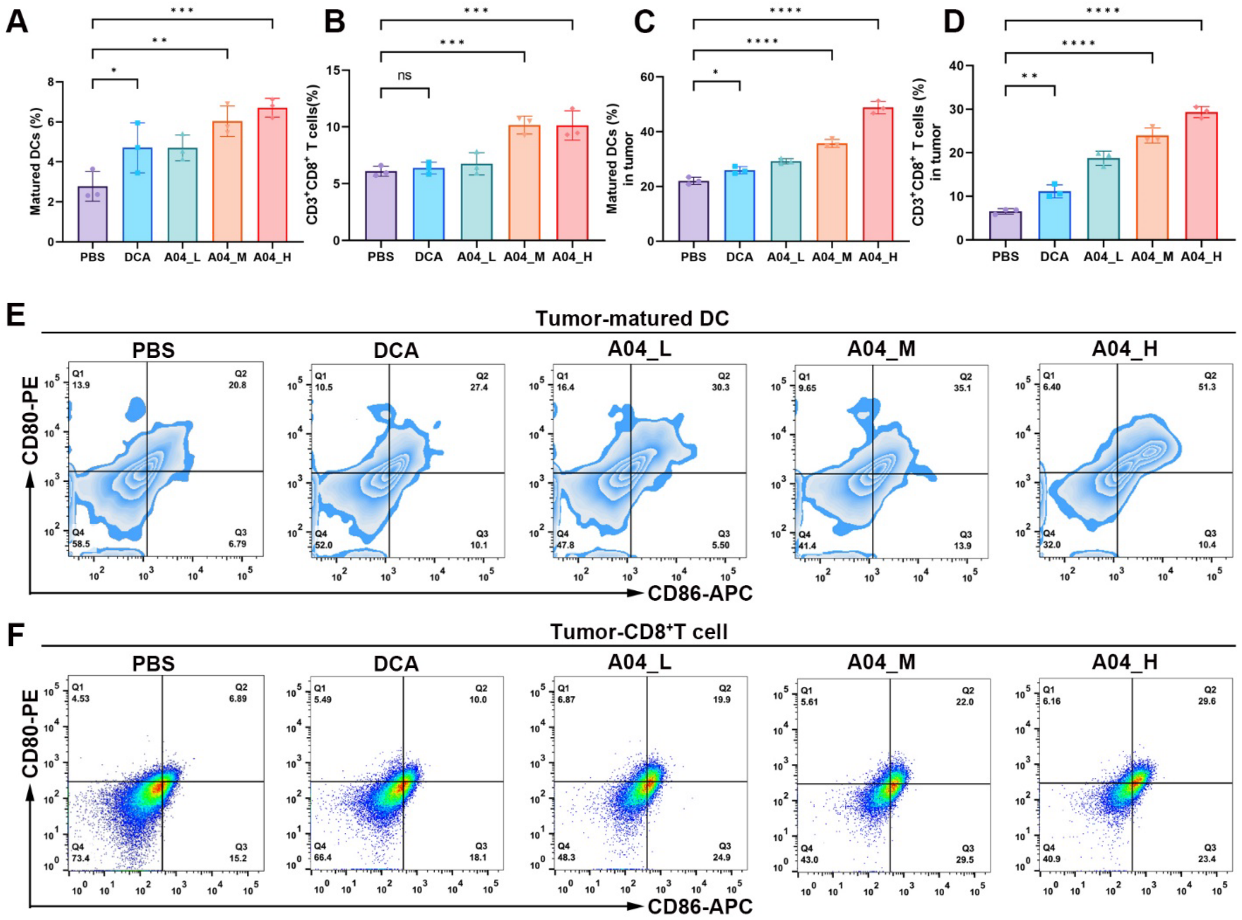

Figure S7. A04 Activates Immune Response by Inducing ICD. A, Statistical analysis of matured DC cells in spleen. (n=3). B, Statistical analysis of $CD8^{+}$T cells in spleen. (n=3). C, Statistical analysis of matured DC cells in tumor. (n=3). D, Statistical analysis of $CD8^{+}$T cells in tumor. (n=3). E, Flow cytometry analysis of DCs in tumor. F, Flow cytometry analysis of $CD8^{+}$ T cells in tumor. G, Immunohistochemistry of Granzyme B and IF staining of perforin in tumor after treatment.

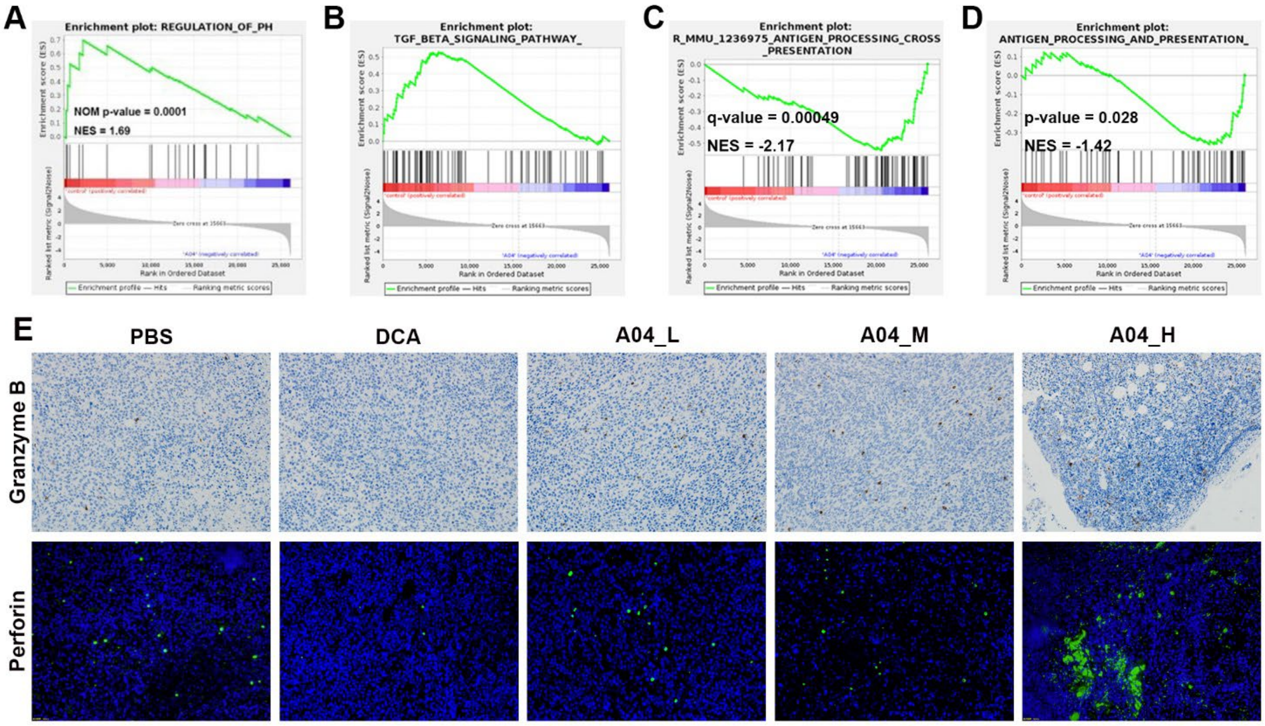

Figure S8. A04 Reverses Tumor Immunosuppressive Microenvironment. A, GSEA on REGULATION_OF_PH PATHWAY. B, GSEA on TGF-β after treatment with **A04** in 4T1 cells. C-D, GSEA on antigen-process related pathway after treatment with **A04** in 4T1 cells. E, Immunohistochemistry of Granzyme B and IF staining of perforin in tumor after treatment.

Supplementary table

Table S1 Dilution and supplier of antibodies.

Antibody	dilution	Supplier	Cat. Num.
Anti-PDK1 antibody	1:2000	ABbcam	ab202468
Anti-PDHA1 (phosphor-S293) antibody	1:1000	ABbcam	ab92696
Anti-alpha Tubulin antibody	1:2000	ABbcam	ab7291
1:1000; Anti-Bax antibody	1:2000	ABbcam	ab182733
Anti-Bcl-2 antibody	1:1000	ABbcam	ab182858
Anti-Calreticulin antibody	1:1000	ABbcam	ab92516
Anti-HMGB1 antibody	1:1000	ABbcam	ab79823
Anti-beta Actin	1:5000	CST	ab8226
44/42 MAPK (Erk1/2) Rabbit mAb	1:1000	CST	4695
Phospho-p44/42 MAPK (Erk1/2) (Thr202/Tyr204) Rabbit mAb	1:2000	CST	4370
SAPK/JNK Antibody	1:1000	CST	9252
Phospho-SAPK/JNK (Thr183/Tyr185) Rabbit mAb	1:1000	CST	4668
p38 MAPK Rabbit mAb	1:1000	CST	8690
Phospho-p38 MAPK (Thr180/Tyr182) (28B10) Mouse mAb	1:2000	CST	9216
Pyruvate Dehydrogenase (C54G1) Rabbit mAb	1:1000	CST	3205

Cleaved Caspase-8 (Asp387) (D5B2) XP® Rabbit mAb (Mouse Specific)	1:1000	CST	8592
Caspase-8 Antibody (Mouse Specific)	1:1000	CST	4927
CD11c (3.9) Mouse mAb (FITC Conjugate)	1:20	CST	69627
PE Anti-Mouse CD80	1:100	Proteintech	PE-65076
APC Anti-Mouse CD86	1:50	Proteintech	APC-65068
CD8α (RPA-T8) Mouse mAb (APC Conjugate)	1:20	CST	64915
CD3 (UCHT1) Mouse mAb (PE Conjugate)	1:20	CST	46233
Anti -CD4 Mouse mAb	1:100	ServiceBio	GB13064-1
Anti -CD8 alpha Mouse mAb	1:1000	ServiceBio	GB12068
Anti -NCAM1 Mouse mAb	1:600	ServiceBio	GB12041
Anti -F4/80 Rabbit pAb	1:500	ServiceBio	GB11027
FITC conjugated Goat Anti-Mouse IgG	1:100	ServiceBio	GB22301
Cy3 conjugated Goat Anti-mouse IgG	1:100	ServiceBio	GB21301
Recombinant Anti-beta Actin antibody (Mouse mAb)	1:1000	ServiceBio	GB15001
Recombinant Anti-beta Actin antibody (Rabbit mAb)	1:1000	ServiceBio	GB15003

Chemical synthesis and characterization

Preparation for intermediate A-A_{1-6}

To a stirred and cooled solution (0 °C), 1,3-propanediamine (500.00 mg, 6.75 mmol) in 30 ml dichloromethane (DCM) was added slowly add di-tert-butyldecarbonate (378.47 mg, 1.69 mmol) dropwise at room temperature overnight. After that, the reaction mix was extracted by DCM and organic layer was washed by brine then dried by anhydrous Na_2SO_4. colorless oil compound (A-A_1) was harvested by silica gel column chromatography (20：1, DCM：MeOH).

A-A_1 (Colorless oil, 61.23%). ^{1}H NMR (500 MHz, $CDCl_3$) δ 4.96 (s, 1H), 3.21 (q, J = 6.51 Hz, 2H), 2.77 (t, J = 6.72 Hz, 2H), 1.67 (s, 2H), 1.62 (p, J = 6.81 Hz, 2H), 1.44 (s, 9H).^{13}C NMR (126 MHz, $CDCl_3$) δ 156.2, 78.9, 39.3, 38.1, 33.2, 28.3.

A-A_2 (Colorless oil, 73.43%). ^{1}H NMR (500 MHz, $CDCl_3$) δ 4.75 (s, 1H), 3.08 (d, J = 6.56 Hz, 2H), 2.67 (s, 2H), 1.40 (s, 13H).^{13}C NMR (126 MHz, $CDCl_3$) δ 156.0, 78.9, 41.7, 40.3, 30.7, 28.4, 27.4.

A-A_3 (Colorless oil, 56.45%). ^{1}H NMR (500 MHz, $CDCl_3$) δ 4.60 (s, 1H), 3.12 (s, 2H), 2.68 (s, 2H), 1.44 (s, 15H), 1.33 (s, 2H).^{13}C NMR (126 MHz, $CDCl_3$) δ 156.0, 78.9, 41.9, 40.4, 33.4, 30.0, 28.4, 26.5, 26.5.

A-A_4 (Colorless oil, 31.26%). ^{1}H NMR (500 MHz, $CDCl_3$) δ 4.60 (s, 1H), 3.11 (s, 1H), 2.69 (s, 1H), 1.61 (s, 2H), 1.44 (s, 14H), 1.30 (s, 9H).^{13}C NMR (126 MHz, $CDCl_3$) δ 156.0, 79.0, 42.1, 40.6, 33.7, 30.0, 29.4, 29.2, 28.4, 26.8, 26.7.

A-A_5 (Colorless oil, 37.35%). ^{1}H NMR (500 MHz, $CDCl_3$) δ 5.26 (s, 1H), 3.64 (s, 4H), 3.60 (t, J = 5.18 Hz, 2H), 3.52 – 3.24 (m, 6H), 2.98 (t, J = 5.34 Hz, 2H), 1.45 (s, 9H).^{13}C NMR (126 MHz, $CDCl_3$) δ 156.1, 79.2, 72.3, 70.2, 70.2, 70.1, 41.3, 40.3, 28.4.

A-A_6 (Colorless oil, 65.43%),^{1}H NMR (500 MHz, DMSO-d_6) δ 6.74 (t, J = 5.58 Hz, 1H), 2.90 (q, J = 6.25 Hz, 2H), 2.52 (t, J = 6.58 Hz, 2H), 1.37 (s, 9H).^{13}C NMR (126 MHz, DMSO-d_6) δ =156.1, 77.8, 44.1, 42.0, 28.7.

Preparation for intermediate A-B_{1-6}

To a solution of 4-fluoro-thalidomide (150.00 mg,0.57 mmol) dissolved in 3 ml ethyl acetate (EA) was added tert-butyl-3- (aminopropyl) carbamate (150.00 mg,0.86 mmol) and di-isopropylethylamine (DIPEA) (209.37 mg,1.72 mmol) at 75 °C. Then, the mixture was heated to 80 °C for reflux overnight. After completion, yellow mixture was quenched by water and extracted

by EA for three times. Organic layer was washed by saturated brine and dried over anhydrous Na_2SO_4. Chartreuse product (156.52 mg) was purified by silica gel column chromatography (50:1, DCM: MeOH).

A-B$_1$ (chartreuse solid, 43.23%). ^{1}H NMR (500 MHz, DMSO-*d$_6$*) δ 11.10 (s, 1H), 7.58 (dd, *J* = 8.62 Hz, 7.05, 1H), 7.09 (d, *J* = 8.56 Hz, 1H), 7.03 (d, *J* = 6.96 Hz, 1H), 6.92 (t, *J* = 5.78 Hz, 1H), 6.67 (t, *J* = 6.14 Hz, 1H), 5.06 (dd, *J* = 12.70, 5.46 Hz, 1H), 3.34 – 3.26 (m, 2H), 3.01 (q, *J* = 6.41 Hz, 2H), 2.89 (ddd, *J* = 16.84, 13.77, 5.38 Hz, 1H), 2.64 – 2.52 (m, 2H), 2.08 – 1.99 (m, 1H), 1.66 (p, *J* = 6.58 Hz, 2H), 1.38 (s, 9H).^{13}C NMR (126 MHz, DMSO-*d$_6$*) δ 173.3, 170.6, 169.3, 167.8, 156.2, 146.8, 136.7, 132.7, 117.6, 110.8, 109.6, 78.0, 55.4, 49.0, 37.7, 31.5, 29.4, 28.7, 22.6.

A-B$_2$ (chartreuse solid, 45.28%). ^{1}H NMR (500 MHz, DMSO-*d$_6$*) δ 11.09 (s, 1H), 7.58 (dd, *J* = 8.54, 7.06 Hz, 1H), 7.11 (d, *J* = 8.56 Hz, 1H), 7.02 (d, *J* = 7.01 Hz, 1H), 6.83 (t, *J* = 5.82 Hz, 1H), 6.55 (t, *J* = 6.01 Hz, 1H), 5.05 (dd, *J* = 12.77, 5.49 Hz, 1H), 3.30 (q, *J* = 6.76 Hz, 2H), 2.95 (q, *J* = 6.55 Hz, 2H), 2.90 – 2.82 (m, 1H), 2.64 – 2.53 (m, 2H), 2.03 (dtd, *J* = 13.04, 5.28, 2.26 Hz, 1H), 1.55 (dd, *J* = 9.16, 5.98 Hz, 2H), 1.45 (p, *J* = 7.06 Hz, 2H), 1.37 (s, 9H).^{13}C NMR (126 MHz, DMSO-*d$_6$*) δ 173.3, 170.6, 169.4, 167.8, 156.1, 146.9, 136.7, 132.7, 117.7, 110.8, 109.5, 77.9, 55.4, 49.0, 42.0, 31.4, 28.7, 27.4, 26.6, 22.6.

A-B$_3$ (chartreuse solid, 46.38%). ^{1}H NMR (500 MHz, DMSO-*d$_6$*) δ 11.09 (s, 1H), 7.63 (dd, *J* = 8.54, 7.06 Hz, 1H), 7.14 (d, *J* = 8.64 Hz, 1H), 7.07 (d, *J* = 6.96 Hz, 1H), 6.71 (t, *J* = 5.71 Hz, 1H), 6.58 (t, *J* = 5.95 Hz, 1H), 5.10 (dd, *J* = 12.78, 5.47 Hz, 1H), 3.34 (q, *J* = 6.78 Hz, 2H), 2.94(ddd, *J* = 16.38, 13.47, 5.89 Hz, 3H), 2.69 – 2.56 (m, 2H), 2.08 (dtd, *J* = 12.97, 5.20, 2.17 Hz, 1H), 1.62 (p, 7.19 Hz, 2H), 1.42 (m, 15H).^{13}C NMR (126 MHz, $CDCl_3$) δ 171.1, 169.5, 168.4, 167.6, 156.0, 147.0, 136.1, 132.5, 116.6, 111.4, 109.9, 79.1, 48.9, 42.6, 40.5, 31.4, 30.0, 29.2, 28.4, 26.6, 26.54, 22.8.

A-B$_4$ (chartreuse solid, 26.38%). ^{1}H NMR (400 MHz, $CDCl_3$) δ 8.26 (s, 1H), 7.49 (ddd, *J* = 8.82, 7.20, 2.15 Hz, 1H), 7.09 (dd, *J* = 7.12, 2.38 Hz, 1H), 6.88 (dd, *J* = 8.60, 1.98 Hz, 1H), 6.23 (t, *J* = 5.58 Hz, 1H), 4.92 (dd, *J* = 12.05, 5.30 Hz, 1H), 4.54 (s, 2H), 3.26 (q, *J* = 6.59 Hz, 2H), 3.10 (q, *J* = 6.84 Hz, 2H), 2.98 – 2.83 (m, 1H), 2.81 – 2.70 (m, 2H), 2.20 – 2.03 (m, 1H), 1.78 – 1.56 (m, 2H), 1.44 (s, 13H), 1.36 – 1.25 (m, 6H).^{13}C NMR (101 MHz, $CDCl_3$) δ 171.0, 169.5, 168.4, 167.6, 156.0, 147.0, 136.1, 132.5, 116.7, 111.4, 109.9, 79.1, 77.2, 48.9, 42.6, 40.6, 31.4, 30.0, 29.2, 29.1, 28.4, 26.8, 26.7, 22.8.

A-B_5 (chartreuse solid, 31.57%). ^{1}H NMR (500 MHz, DMSO-d_6) δ 11.09 (s, 1H), 7.59 (dd, *J* = 8.53, 7.09 Hz, 1H), 7.15 (d, *J* = 8.61, 1H), 7.04 (d, *J* = 7.01 Hz, 1H), 6.73 (t, *J* = 5.78 Hz, 1H), 6.61 (t, *J* = 5.83 Hz, 1H), 5.06 (dd, *J* = 12.78, 5.41 Hz, 1H), 3.62 (t, *J* = 5.47 Hz, 2H), 3.58 – 3.54 (m, 2H), 3.52 (dd, *J* = 4.43, 1.77 Hz, 2H), 3.47 (q, *J* = 5.57 Hz, 2H), 3.38 (d, *J* = 6.13 Hz, 2H), 3.06 (t, *J* = 5.97 Hz, 2H), 2.94 – 2.82 (m, 1H), 2.65 – 2.52 (m, 2H), 2.07 – 1.93 (m, 1H), 1.36 (s, 9H).^{13}C NMR (126 MHz, $CDCl_3$) δ = 171.2, 169.4, 168.5, 167.6, 156.1, 146.8, 136.1, 132.6, 116.7, 111.7, 110.4, 77.2, 70.8, 70.4, 70.2, 69.4, 48.9, 42.3, 40.4, 31.4, 28.4, 22.9.

A-B_6 (chartreuse solid, 41.73%). ^{1}H NMR (500 MHz, DMSO-d_6) δ 11.09 (s, 1H), 7.58 (dd, *J* = 8.58, 7.05 Hz, 1H), 7.15 (d, *J* = 8.61 Hz, 1H), 7.02 (dd, *J* = 13.05, 6.42, 1H), 6.71 (t, *J* = 6.21 Hz, 1H), 5.05 (dd, *J* = 12.77, 5.45 Hz, 1H), 3.37 (d, *J* = 6.33 Hz, 2H), 3.13 (q, *J* = 6.09 Hz, 2H), 2.89 (ddd, *J* = 16.87, 13.80, 5.44 Hz, 1H), 2.71 – 2.56 (m, 2H), 2.11 – 1.85 (m, 1H), 1.37 (s, 9H).^{13}C NMR (126 MHz, DMSO-d_6) δ 173.3, 170.5, 169.2, 167.8, 156.4, 146.9, 136.6, 132.7, 117.5, 111.0, 109.7, 78.3, 49.0, 42.1, 31.4, 28.7, 22.6.

General synthesis for intermediate A-C_{1-6}

Trifluoroacetic acid (1 ml) was applied for de-protection by reacting with intermediate (A-B_{1-6}) in DCM for 1 h, and yellow solid was collected for next step after solvent evaporation and without purification.

Preparation for compound A01~A06. To a stirred and cooled solution (0 °C), **A-C_1** (25.31 mg, 0.07 mmol) in N, N-Dimethylformamide (DMF) was reacted with DCA (8.91 mg, 0.07 mmol) accompanied with DIPEA (59.36 mg, 0.42 mmol) for 0.5 h. Then, 2-(7-Azabenzotriazol-1-yl)-*N, N, N', N'*-tetramethyluronium hexafluorophosphate (HATU) (29.16 mg, 0.08 mmol) was added to react overnight at room temperature. After completion, the reaction mix was extracted by DCM for three times and washed by saturated brine. After drying by anhydrous Na_2SO_4, yellow solid (14.01 mg) was puryfied from silica gel column chromatography (DCM：MeOH=30：1).

A01 (yellow solid, 45.47%). ^{1}H NMR (400 MHz, DMSO-d_6) δ 11.10 (s, 1H), 8.70 (s, 1H), 7.59 (dd, *J* = 8.56, 7.02 Hz, 1H), 7.10 (d, *J* = 8.61 Hz, 1H), 7.04 (d, *J* = 7.00 Hz, 1H), 6.72 (t, *J* = 6.29 Hz, 1H), 6.46 (d, *J* = 1.16 Hz, 1H), 5.06 (dd, *J* = 12.82, 5.38 Hz, 1H), 3.48 (s, 2H), 3.23 (t, *J* = 6.30 Hz, 2H), 2.89 (ddd, *J* = 17.78, 13.90, 5.39 Hz, 1H), 2.66 – 2.53 (m, 2H), 2.04 (dd, *J* = 9.55 Hz, 4.44, 1H), 1.74 (p, *J* = 6.85 Hz, 2H).^{13}C NMR (126 MHz, DMSO-d_6) δ 173.3, 170.6, 169.3, 167.8, 164.2, 146.7,

136.7, 132.8, 117.6, 111.0, 109.7, 67.5, 49.0, 38.7, 37.4, 31.4, 28.6, 22.6. HRMS (ESI): Calcd for $[M+H]^+$ $C_{18}H_{19}Cl_2N_4O_5$, 441.0733; found, 441.0751. Purity >95%.

A02 (yellow solid, 35.47%). ^{1}H NMR (400 MHz, $CDCl_3$) δ 8.03 (s, 1H), 7.50 (dd, *J* = 8.44 Hz, 7.02, 1H), 7.11 (d, *J* = 7.10 Hz, 1H), 6.89 (d, *J* = 8.54 Hz, 1H), 6.59 (s, 1H), 5.93 (s, 1H), 4.92 (dd, *J* = 12.12, 5.34 Hz, 1H), 3.40 (d, *J* = 5.98 Hz, 2H), 3.33 (s, 2H), 2.94 – 2.86 (m, 1H), 2.77 (td, *J* = 15.65, 8.55 Hz, 2H), 2.18 – 2.08 (m, 1H), 1.82 (s, 2H), 1.51 – 1.39 (m, 2H).^{13}C NMR (126 MHz, DMSO-d_6) δ 173.3, 170.6, 167.8, 164.0, 162.8, 146.8, 136.7, 132.7, 117.7, 110.9, 109.5, 67.4, 49.0, 41.9, 38.7, 36.3, 31.4, 29.5, 26.4. HRMS (ESI): Calcd for $[M+H]^+$ $C_{19}H_{21}Cl_2N_4O_5$, 455.0889, found, 455.0911. Purity >95%.

A03 (yellow solid, 34.98%). ^{1}H NMR (400 MHz, $CDCl_3$) δ 8.13 (s, 1H), 7.49 (ddd, *J* = 8.37, 7.04 Hz, 1.10, 1H), 7.09 (d, *J* = 7.08 Hz, 1H), 6.88 (d, *J* = 8.52 Hz, 1H), 6.54 (s, 1H), 6.23 (t, *J* = 5.70 Hz, 1H),5.92 (d, *J* = 1.10 Hz, 1H), 4.92 (dd, *J* = 12.03, 5.33 Hz, 1H), 3.35 (t, *J* = 6.76 Hz, 2H), 3.29 – 3.23 (m, 2H), 2.96 – 2.86 (m, 1H), 2.82 – 2.62 (m, 2H), 2.13 (dt, *J* = 10.25, 3.88 Hz, 1H), 1.69 (q, *J* = 7.09 Hz, 2H), 1.60 (d, *J* = 7.49 Hz, 2H), 1.48 – 1.42 (m, 4H).^{13}C NMR (126 MHz, DMSO-d_6) δ 173.3, 170.6, 169.4, 167.8, 162.8, 146.9, 136.8, 132.7, 117.7, 109.5, 67.4, 49.0, 42.2, 38.7, 36.3, 31.4, 31.2, 29.1, 28.9, 26.4, 22.6. HRMS (ESI): Calcd for $[M+H]^+$ $C_{21}H_{25}Cl_2N_4O_5$, 483.1202, found, 483.1221. Purity >95%.

A04 (yellow solid, 25.56%).^{1}H NMR (400 MHz, $CDCl_3$) δ 8.19 (s, 1H), 7.49 (dd, *J* = 8.54, 7.09 Hz, 1H), 7.08 (d, *J* = 7.09 Hz, 1H), 6.88 (d, *J* = 8.51 Hz, 1H), 6.53 (s, 1H), 6.22 (t, *J* = 5.58 Hz, 1H), 5.92 (s, 1H), 4.99 – 4.87 (m, 1H), 3.43 – 3.16 (m, 4H), 2.99 – 2.83 (m, 1H), 2.82 – 2.61 (m, 2H), 2.13 (ddd, *J* = 12.27, 5.71, 3.26 Hz, 1H), 1.78 – 1.53 (m, 6H),1.36 – 1.25 (m, 6H). ^{13}C NMR (101 MHz, $CDCl_3$) δ 171.0, 169.5, 168.4, 167.7, 164.1, 147.0, 116.7, 111.4, 109.9, 66.5, 48.9, 42.6, 40.4, 31.4, 29.1, 29.1, 29.0, 26.8, 26.5, 22.8. HRMS (ESI): Calcd for $[M+H]^+$ $C_{23}H_{29}Cl_2N_4O_5$, 511.1515, found, 511.1531. Purity >95%.

A05 (yellow solid, 38.54%). ^{1}H NMR (400 MHz, $CDCl_3$) δ 8.37 (s, 1H), 7.50 (dd, *J* = 8.58, 7.17 Hz, 1H), 7.12 (d, *J* = 7.10 Hz, 2H), 6.91 (d, *J* = 8.53 Hz, 1H), 6.52 (t, *J* = 5.60 Hz, 1H), 5.94 (s, 1H), 4.97 – 4.88 (m, 1H), 3.74 (t, *J* = 5.23 Hz, 2H), 3.67 (d, *J* = 12.52 Hz, 6H), 3.60 – 3.51 (m, 2H), 3.49 (dd, *J* = 10.34, 5.03 Hz, 2H), 2.91 – 2.80 (m, 1H), 2.83 – 2.66 (m, 2H), 2.13 (ddd, *J* = 8.17, 6.40, 3.90 Hz, 1H).^{13}C NMR (101 MHz, $CDCl_3$) δ 171.1, 169.5, 168.5, 167.6, 164.2, 146.8, 136.1, 132.5,

116.7, 111.8, 110.4, 70.7, 70.3, 69.3, 69.1, 66.5, 48.9, 42.3, 40.2, 31.4, 22.9. HRMS (ESI): Calcd for $[M+H]^+$, $C_{21}H_{25}Cl_2N_4O_7$, 515.1100, found, 515.1123. Purity >95%.

A06 (yellow solid, 42.47%). ^{1}H NMR (500 MHz, DMSO-*d6*) δ 11.10 (s, 1H), 8.80 (t, *J* = 5.62 Hz, 1H), 7.60 (dd, *J* = 8.58, 7.09 Hz, 1H), 7.18 (d, *J* = 8.65 Hz, 1H), 7.05 (d, *J* = 7.01 Hz, 1H), 6.76 (t, *J* = 6.32 Hz, 1H), 6.45 (s, 1H), 5.06 (dd, *J* = 12.76, 5.44 Hz, 1H), 3.55 – 3.35 (m, 2H), 3.35 (s, 2H), 2.89 (ddd, *J* = 16.94, 13.84, 5.43 Hz, 1H), 2.63 – 2.55 (m, 2H), 2.03 (dtd, *J* = 12.95, 5.29, 2.28 Hz, 1H). ^{13}C NMR (126 MHz, DMSO-*d6*) δ 173.3, 170.6, 169.2, 167.8, 164.6, 146.7, 136.7, 132.7, 117.6, 111.2, 109.9, 67.3, 49.0, 41.3, 38.7, 31.5, 22.6. HRMS (ESI): Calcd for $[M+H]^+$, $C_{17}H_{17}Cl_2N_4O_5$, 427.0576, found, 427.0589. Purity >95%.

Synthesis of series-B compounds

General synthesis for intermediate 4a-4h

To a solution of 3-aminopropanol (58.96 mg, 0.54 mmol) in EA (15 ml), DIPEA (209.37 mg, 1.72 mmol) was added. The mixture was heated to 75 °C, then 4-fluoro-thalidomide (100.00 mg, 0.36 mmol) dissolved in EA was added and heated to 80 °C under reflux. Overnight, yellow mixture was quenched by water and extracted by EA for three times. Organic layer was washed by saturated brine and dried over anhydrous Na_2SO_4. Chartreuse product (54.17 mg) was purified by silica gel column chromatography (30:1, DCM: MeOH).

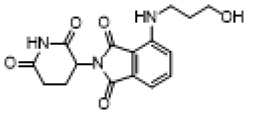

4a (chartreuse solid, 45.45%). ^{1}H NMR (500 MHz, DMSO-*d6*) δ 11.09 (s, 1H), 7.59 (dd, *J* = 8.54, 7.08 Hz, 1H), 7.10 (d, *J* = 8.63 Hz, 1H), 7.03 (d, *J* = 7.00 Hz, 1H), 6.71 (t, *J* = 5.87 Hz, 1H), 5.06 (dd, *J* = 12.74, 5.42 Hz, 1H), 4.65 (t, *J* = 4.97 Hz, 1H), 3.52 (q, *J* = 5.59 Hz, 2H), 3.37 (q, *J* = 6.49 Hz, 2H), 2.89 (ddd, *J* = 16.86, 13.77, 5.41 Hz, 1H), 2.64 – 2.52 (m, 2H), 2.08 – 1.99 (m, 1H), 1.73 (p, *J* = 6.38 Hz, 2H). ^{13}C NMR (126 MHz, DMSO-*d6*) δ 173.3, 170.6, 169.3, 167.8, 146.9, 136.7, 132.7, 117.6, 110.8, 109.5, 59.0, 55.4, 49.0, 32.1, 31.5, 22.6.

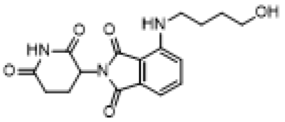

4b (chartreuse solid, 48.45%).^{1}H NMR (500 MHz, $CDCl_3$) δ 8.24 (s, 1H), 7.51 (dd, *J* = 8.45, 7.14 Hz, 1H), 7.11 (d, *J* = 7.07 Hz, 1H), 6.92 (d, *J* = 8.54 Hz, 1H), 4.94 (dd, *J* = 12.31, 5.32 Hz, 1H), 3.73 (t, *J* = 6.16 Hz, 2H), 3.35 (t, *J* = 6.84 Hz, 2H), 2.97 – 2.52 (m, 3H), 2.15 (ddd, *J* = 12.04, 6.11, 3.53 Hz, 1H), 2.11 – 1.75 (m, 2H), 1.75 – 1.57 (m, 2H).^{13}C NMR (126 MHz, DMSO-d_6) δ 173.3, 170.6, 169.4, 167.8, 146.9, 136.7, 132.7, 117.7, 110.8, 109.5, 60.9, 49.0, 42.2, 31.5, 30.2, 25.9, 22.6.

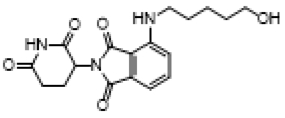

4c (chartreuse solid, 43.46%). ^{1}H NMR (500 MHz, $CDCl_3$) δ 8.22 (s, 1H), 7.51 (dd, *J* = 8.51, 7.11 Hz, 1H), 7.11 (d, *J* = 7.09 Hz, 1H), 6.90 (d, *J* = 8.55 Hz, 1H), 5.12 – 4.78 (m, 1H), 3.70 (t, *J* = 6.41 Hz, 2H), 3.31 (t, *J* = 7.02 Hz, 2H), 3.04 – 2.61 (m, 3H), 2.15 (ddd, *J* = 12.24, 6.16, 3.57 Hz, 1H), 1.73 (p, *J* = 7.21 Hz, 2H), 1.69 – 1.60 (m, 2H), 1.53 (qd, *J* = 7.42, 4.17 Hz, 2H).^{13}C NMR (126 MHz, $CDCl_3$) δ 171.3, 169.6, 168.5, 167.7, 147.0, 136.2, 132.5, 116.7, 111.4, 109.9, 62.6, 48.9, 42.6, 32.3, 31.4, 29.0, 23.2, 22.8

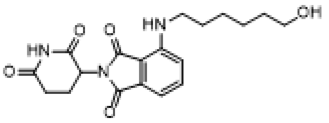

4d (chartreuse solid, 42.56%) ^{1}H NMR (400 MHz, $CDCl_3$) δ 8.24 (s, 1H), 7.49 (dd, *J* = 8.58, 7.11 Hz, 1H), 6.24 (s, 1H), 3.66 (t, *J* = 6.47 Hz, 2H), 3.27 (q, *J* = 6.46 Hz, 2H), 2.94 – 2.84 (m, 1H), 2.83 – 2.64 (m, 2H), 2.18 – 2.08 (m, 1H), 1.76 – 1.64 (m, 2H), 1.59 (dd, *J* = 13.71, 7.06 Hz, 2H), 1.45 (tt, *J* = 5.50, 2.57 Hz, 4H).^{13}C NMR (126 MHz, $CDCl_3$) δ 171.8, 169.6, 168.9, 167.7, 147.0, 136.1, 132.5, 116.7, 111.3, 109.8, 62.6, 48.9, 42.5, 32.5, 31.4, 29.1, 26.7, 25.5, 22.8.

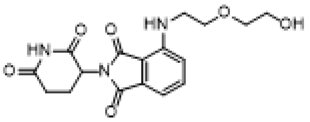

4e (chartreuse solid, 35.76%).^{1}H NMR (500 MHz, $CDCl_3$) δ 8.27 (s, 1H), 7.70 – 7.41 (m, 1H), 7.14 (d, *J* = 7.11 Hz, 1H), 6.94 (d, *J* = 8.51 Hz, 1H), 4.95 (dd, *J* = 12.28, 5.26 Hz, 1H), 3.87 – 3.71 (m, 4H), 3.72 – 3.58 (m, 2H), 3.51 (t, *J* = 5.33 Hz, 2H), 3.14 – 2.64 (m, 3H), 2.25 – 2.08 (m, 1H).^{13}C NMR (126 MHz, $CDCl_3$) δ 171.5, 169.5, 168.7, 167.6, 146.9, 136.1, 132.5, 116.8, 111.8, 110.4, 72.5, 69.3, 61.8, 48.9, 42.3, 31.4, 22.7.

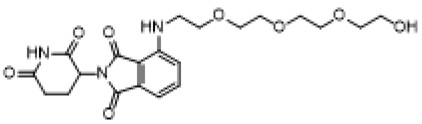

4f (chartreuse solid, 38.54%) ^{1}H NMR (500 MHz, $CDCl_3$) δ 8.30 (s, 1H), 7.50 (dd, *J* = 8.43, 7.17 Hz, 1H), 7.11 (d, *J* = 7.11 Hz, 1H), 6.93 (d, *J* = 8.49 Hz, 1H), 4.96 – 4.89 (m, 1H), 3.72 (t, *J* = 4.82 Hz, 4H), 3.68 (s, 8H), 3.64 – 3.58 (m, 2H), 3.48 (t, *J* = 5.39 Hz, 2H), 2.91 – 2.84 (m, 1H), 2.86 – 2.67 (m, 2H), 2.17 – 2.09 (m, 1H).^{13}C NMR (126 MHz, $CDCl_3$) δ 171.5, 169.3, 168.7, 167.7, 146.8, 136.1, 132.5, 116.8, 111.7, 110.3, 72.5, 70.7, 70.7, 70.5, 70.3, 69.5, 61.7, 48.9, 42.4, 31.4, 22.8.

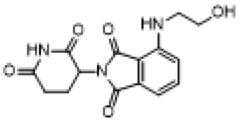

4g (chartreuse solid, 54.21%).^{1}H NMR (500 MHz, DMSO-d_6) δ 11.10 (s, 1H), 7.59 (dd, *J* = 8.55, 7.08 Hz, 1H), 7.13 (d, *J* = 8.59 Hz, 1H), 6.65 (t, *J* = 5.80 Hz, 1H), 5.06 (dd, *J* = 12.67, 5.44 Hz, 1H), 4.91 (t, *J* = 5.15 Hz, 1H), 3.60 (q, *J* = 5.41 Hz, 2H), 3.37 (q, *J* = 5.68 Hz, 2H), 2.89 (ddd, *J* = 16.80, 13.70, 5.39 Hz, 1H), 2.66 – 2.55 (m, 2H), 2.03 (ddt, *J* = 12.88, 5.67, 3.00 Hz, 1H). ^{13}C NMR (126 MHz, DMSO-d_6) δ 173.3, 170.6, 169.4, 167.8, 147.1, 136.7, 132.6, 117.9, 111.0, 109.6, 59.8, 49.0, 44.8, 31.5, 22.6.

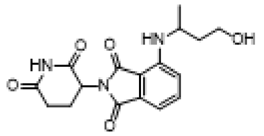

4h (chartreuse solid, 53.87%). ^{1}H NMR (500 MHz, DMSO-d_6) δ 11.05 (s, 1H), 7.59 (dd, J = 8.56, 7.04 Hz, 1H), 7.14 (d, J = 8.64 Hz, 1H), 7.02 (d, J = 7.02 Hz, 1H), 6.40 (dd, J = 8.51, 2.25 Hz, 1H), 5.05 (dd, J = 12.79, 5.44 Hz, 1H), 4.63 (td, J = 4.80, 1.28 Hz, 1H), 3.90 (dq, J = 8.30, 6.28 Hz, 1H), 3.53 (ddd, J = 8.95, 6.40, 4.42 Hz, 2H), 2.88 (ddd, J = 16.93, 13.85, 5.44 Hz, 1H), 2.64 – 2.51 (m, 2H), 2.03 (dtd, J = 12.85, 5.21, 2.23 Hz, 1H), 1.71 (tdd, J = 15.04, 10.89, 7.11 Hz, 2H), 1.21 (dd, J = 6.41, 1.12 Hz, 3H). ^{13}C NMR (126 MHz, $CDCl_3$) δ 171.5, 169.6, 168.7, 167.7, 146.4, 136.2, 132.6, 117.2, 111.4, 109.8, 59.4, 48.9, 45.3, 39.3, 31.4, 22.8, 20.7.

General synthesis for compound B01~B06

To a stirred condition, **4a** (30.12 mg,0.08 mmol) was reacted with dichloroacetyl chloride (11.61 mg,0.08 mmol) dropwise at room temperature for 3 h. After quenching with water, the reaction mix was extracted by DCM for three times and washed by saturated brine. After drying by Na_2SO_4, yellow solid (16.04 mg) was harvested by silica gel column chromatography (30:1, DCM: MeOH).

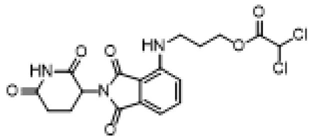

B01 (yellow solid, 45.47%). ^{1}H NMR (400 MHz, $CDCl_3$) δ 8.08 (s, 1H), 7.52 (dd, J = 8.52, 7.10 Hz, 1H), 7.13 (d, J = 7.11 Hz, 1H), 6.91 (d, J = 8.53 Hz, 1H), 6.37 (t, J = 6.06 Hz, 1H), 6.05 (d, J = 0.80 Hz, 1H), 4.92 (dd, J = 12.07, 5.34 Hz, 1H), 4.42 (t, J = 5.88 Hz, 2H), 3.45 (q, J = 6.42 Hz, 2H), 2.90 (dd, J = 15.51, 3.61 Hz, 1H), 2.85 – 2.68 (m, 2H), 2.19 – 2.00 (m, 3H). ^{13}C NMR (101 MHz, $CDCl_3$) δ 170.9, 169.5, 168.2, 167.5, 164.5, 146.6, 136.3, 132.6, 116.4, 112.0, 110.4, 65.1, 64.2, 48.9, 39.4, 31.4, 28.2, 22.8. HRMS (ESI): Calcd for $[M+H]^+$, $C_{18}H_{18}Cl_2N_3O_6$, 442.0573, found, 442.0582.

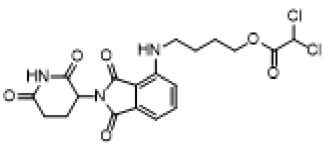

B02 (yellow solid, 35.37%).^{1}H NMR (400 MHz, $CDCl_3$) δ 8.15 (s, 1H), 7.42 (dd, J = 8.54, 7.12 Hz, 1H), 7.03 (d, J = 7.11 Hz, 1H), 6.81 (d, J = 8.48 Hz, 1H), 6.19 (t, J = 5.78 Hz, 1H), 5.89 (s, 1H), 4.84 (dd, J = 12.04, 5.33 Hz, 1H), 4.25 (t, J = 6.11 Hz, 2H), 3.27 (q, J = 6.43 Hz, 2H), 2.93 – 2.77 (m, 1H), 2.74 – 2.57 (m, 2H), 2.16 – 1.93 (m, 1H), 1.87 – 1.64 (m, 4H).^{13}CNMR (101 MHz, $CDCl_3$) δ 171.0, 169.5, 168.4, 167.5, 164.5, 146.7, 136.2,132.5, 116.5, 111.7, 110.2,67.0, 64.3, 48.9, 42.1, 31.4, 25.8, 25.6, 22.8. HRMS (ESI): Calcd for $[M+H]^+$, $C_{19}H_{20}Cl_2N_3O_6$, 456.0729, found, 456.0743.

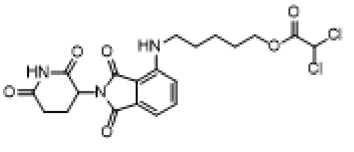

B03 (yellow solid, 47.46%). ^{1}H NMR (400 MHz, $CDCl_3$) δ 8.12 (s, 1H), 7.50 (dd, J = 8.54, 7.13 Hz, 1H), 7.10 (d, J = 7.05 Hz, 1H), 6.88 (d, J = 8.53 Hz, 1H), 6.24 (t, J = 5.79 Hz, 1H), 5.96 (s, 1H), 4.92 (dd, J = 12.10, 5.34 Hz, 1H), 4.30 (t, J = 6.46 Hz, 2H), 3.30 (q, J = 6.60 Hz, 2H), 2.99 – 2.86 (m, 1H), 2.82 – 2.73 (m, 1H), 2.17 – 2.11 (m, 1H), 1.85 – 1.67 (m, 4H), 1.67 – 1.45 (m, 2H).^{13}C NMR (101 MHz, $CDCl_3$) δ 170.9, 169.5, 168.3, 167.6, 164.6, 146.9, 136.2, 132.5, 116.6, 111.6, 110.0, 67.2, 64.3, 48.9, 42.4, 31.4, 28.8, 28.0, 23.1, 22.8. HRMS (ESI): Calcd for $[M+H]^+$, $C_{20}H_{22}Cl_2N_3O_6$, 470.0886, found, 470.0899.

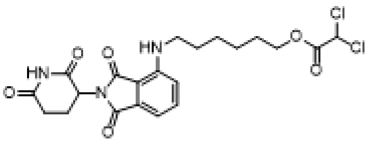

B04 (yellow solid, 55.36%) ^{1}H NMR (400 MHz, $CDCl_3$) δ 8.16 (s, 1H), 7.50 (dd, J = 8.56 Hz, 7.11, 1H), 7.09 (d, J = 7.11 Hz, 1H), 6.88 (d, J = 8.56 Hz, 1H), 6.24 (s, 1H), 5.95 (s, 1H), 4.92 (dd, J = 12.05, 5.33 Hz, 1H), 4.28 (t, J = 6.53 Hz, 2H), 3.32 – 3.23 (m, 2H), 2.97 – 2.85 (m, 1H), 2.77 (td, J = 15.68, 8.80 Hz, 2H), 2.13 (ddd, J = 12.29, 5.67, 3.06, 1H), 1.81 – 1.63 (m, 4H), 1.54 – 1.39 (m, 4H).^{13}C NMR (101 MHz, $CDCl_3$) δ 171.0, 169.5, 168.3, 167.6, 164.6, 146.9,

136.2, 132.5, 116.6, 111.5, 110.0, 67.4, 64.3, 48.9, 42.5, 31.4, 29.1, 28.2, 26.5, 25.4, 22.8. HRMS (ESI): Calcd for $[M+H]^+$, $C_{21}H_{24}Cl_2N_3O_6$, 484.1042, found, 484.1059.

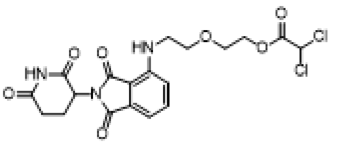

B05(yellow solid, 48.37%). ^{1}H NMR (400 MHz, $CDCl_3$) δ 8.21 (d, *J* = 10.27 Hz, 1H), 7.50 (ddd, *J* = 9.30, 7.06, 2.01 Hz, 1H), 7.12 (dd, *J* = 7.10, 2.04, 1H), 6.92 (d, *J* = 8.46 Hz, 1H), 6.51 (s, 1H), 6.44 (s, 1H), 4.93 (dd, *J* = 12.25, 5.34 Hz, 1H), 4.43 (dq, *J* = 5.03, 2.20 Hz, 2H), 3.76 (dtd, *J* = 15.40, 4.94, 2.15 Hz, 4H), 3.47 (q, *J* = 5.40 Hz, 2H), 2.95 – 2.86 (m, 1H), 2.81 – 2.70 (m, 2H), 2.12 (dd, *J* = 10.99, 5.51 Hz, 1H).^{13}C NMR (101 MHz, $CDCl_3$) δ 171.0, 169.5, 168.3, 167.6, 164.6, 146.8, 136.1, 132.5, 116.8, 111.9, 110.4, 69.6, 68.4, 66.3, 64.3, 48.9, 42.3, 31.4, 22.8. HRMS (ESI): Calcd for $[M+H]^+$, $C_{19}H_{20}Cl_2N_3O_7$, 472.0678, found, 472.0691.

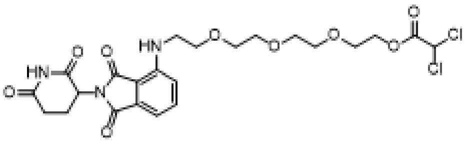

B06 (yellow solid, 46.82%). ^{1}H NMR (400 MHz, $CDCl_3$) δ 8.36 (s, 1H), 7.49 (t, *J* = 7.80 Hz, 1H), 7.10 (d, *J* = 7.11 Hz, 1H), 6.93 (d, *J* = 8.56 Hz, 1H), 6.49 (s, 1H), 6.00 (s, 1H), 4.93 (dd, *J* = 11.76, 5.35 Hz, 1H), 4.53 – 4.37 (m, 2H), 3.74 (dt, *J* = 15.85, 5.07 Hz, 4H), 3.67 (d, *J* = 4.04 Hz, 8H), 3.48 (d, *J* = 5.97 Hz, 2H), 2.95 – 2.83 (m, 1H), 2.77 (qd, *J* = 12.48, 4.11 Hz, 2H), 2.17 – 2.07 (m, 1H).^{13}C NMR (101 MHz, $CDCl_3$) δ 171.2, 169.3, 168.5, 167.6, 164.6, 146.9, 136.1, 132.5, 116.8, 111.7, 110.3, 70.8, 70.7, 70.7, 69.5, 68.5, 66.6, 64.2, 48.9, 42.4, 31.4, 22.8. HRMS (ESI): Calcd for $[M+H]^+$, $C_{23}H_{28}Cl_2N_3O_9$, 560.1203, found, 560.1229.

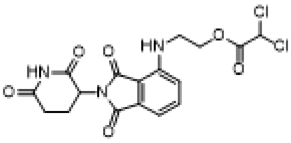

B07 (yellow solid, 44.21%).^{1}H NMR (500 MHz, DMSO-d_6) δ 11.10 (s, 1H), 7.61 (dd, *J* = 8.55, 7.06 Hz, 1H), 7.21 (d, *J* = 8.57 Hz, 1H), 7.07 (d, *J* = 7.02 Hz, 1H), 6.87 (s, 1H), 6.75 (t, *J* = 6.34 Hz, 1H), 5.06 (dd, *J* = 12.79, 5.44 Hz, 1H), 4.43 (t, *J* = 5.42 Hz, 2H), 3.72 – 3.65

(m, 2H), 2.94 – 2.84 (m, 1H), 2.61 – 2.52 (m, 2H), 2.08 – 1.98 (m, 1H). ^{13}C NMR (126 MHz, DMSO-*d*$_6$) δ 173.3, 170.5, 169.2, 167.7, 165.1, 146.6, 136.8, 132.6, 117.9, 111.5, 110.1, 66.5, 65.4, 49.0, 41.0, 31.4, 22.6. HRMS (ESI): Calcd for $[M+H]^+$, $C_{17}H_{16}Cl_2N_3O_6$, 428.0416, found, 428.0432.

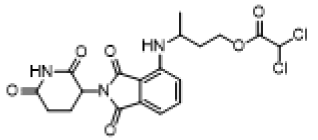

B08 (yellow solid, 56.39%). ^{1}H NMR (400 MHz, $CDCl_3$) δ 8.15 (s, 1H), 7.50 (dd, *J* = 8.54, 7.11 Hz, 1H), 7.11 (d, *J* = 7.08 Hz, 1H), 6.93 (d, *J* = 8.56 Hz, 1H), 6.16 (dd, *J* = 8.94, 4.51 Hz, 1H), 6.01 (d, *J* = 1.84 Hz, 1H), 4.92 (ddd, *J* = 12.19, 5.48, 2.18 Hz, 1H), 4.47 – 4.31 (m, 2H), 3.94 – 3.80 (m, 1H), 2.96 – 2.86 (m, 1H), 2.83 – 2.68 (m, 2H), 2.19 – 2.09 (m, 1H), 1.33 (dd, *J* = 6.42, 1.41 Hz, 3H). ^{13}C NMR (101 MHz, $CDCl_3$) δ 170.9, 169.6, 168.3, 167.4, 164.4, 146.1, 136.3, 132.6, 116.7, 111.8, 110.2, 64.5, 64.2, 48.9, 45.2, 35.4, 31.4, 22.8, 20.9. HRMS (ESI): Calcd for $[M+H]^+$, $C_{19}H_{20}Cl_2N_3O_6$, 456.0729, found, 456.0745.

Synthesis for series-C compounds

General synthesis for intermediate 5a-5c

To a stirring condition on ice (0°C), ((benzyloxy)carbonyl) glycine in DMF was added with DIPEA (493.23 mg, 3.82 mmol) and N-boc-1,3-propanediamine (183.15 mg,1.06 mmol). Half an hour later, HATU (400.12 mg,1.06 mmol) was added and reacted overnight. Then, reaction mixture was extracted by 30 ml DCM and washed with saturated brine. Anhydrous Na_2SO_4 was applied for drying the product. White solid (292.68 mg) was harvested by silica gel column chromatography (50:1, DCM: MeOH).

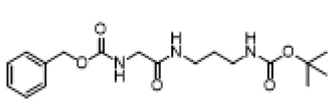

5a (white solid, 83.53%). ^{1}H NMR (400 MHz, $CDCl_3$) δ 7.36 (s, 1H), 7.35 (s, 3H), 7.32 (dd, *J* = 8.24, 3.61 Hz, 1H), 6.78 (s, 1H), 5.48 (s, 1H), 5.13 (s, 2H), 4.85 (s, 1H), 3.87 (d, *J* = 5.76 Hz, 2H), 3.32 (t, *J* = 6.32 Hz, 2H), 3.18 – 3.11 (m, 2H), 1.64 – 1.57 (m, 2H), 1.43

(s, 9H).^{13}C NMR (101 MHz, $CDCl_3$) δ 169.1, 156.7, 156.6, 136.2, 128.5, 128.2, 128.1, 79.5, 67.2, 44.6, 36.9, 35.8, 30.0, 28.4.

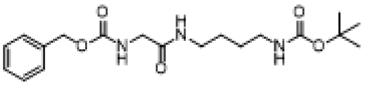

5b (white solid, 87.57%).^{1}H NMR (400 MHz, $CDCl_3$) δ 7.38 – 7.29 (m, 5H), 6.35 (s, 1H), 5.57 (s, 1H), 5.13 (s, 2H), 4.64 (s, 1H), 3.85 (d, J = 5.73 Hz, 2H), 3.28 (q, J = 6.26 Hz, 2H), 3.11 (q, J = 6.44 Hz, 2H), 1.50 (s, 4H), 1.43 (s, 9H). ^{13}C NMR (101 MHz, $CDCl_3$) δ 168.9, 156.2, 136.2, 128.6, 128.3, 128.1, 79.3, 67.2, 44.7, 40.0, 39.2, 28.4, 27.6, 26.3.

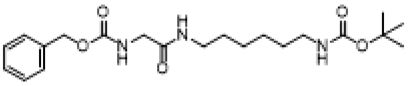

5c (white solid, 86.39%). ^{1}H NMR (400 MHz, $CDCl_3$) δ 7.39 – 7.29 (m, 5H), 6.21 (s, 1H), 5.58 (s, 1H), 5.13 (s, 2H), 4.57 (s, 1H), 3.85 (d, J = 5.74 Hz, 2H), 3.24 (q, J = 6.66 Hz, 2H), 3.09 (q, J = 6.77 Hz, 2H), 1.43 (s, 15H), 1.31 (q, J = 3.56 Hz, 2H).^{13}C NMR (101 MHz, $CDCl_3$) δ 168.8, 156.7, 156.1, 136.2, 128.6, 128.3, 128.1, 79.1, 67.2, 44.7, 40.1, 39.2, 29.9, 29.2, 28.4, 26.0, 25.9.

General synthesis for intermediate 6a-6c

In a solution with **5a** in methanol, hydrogen was pumped into liquid accompanied with 5% palladium carbon and reacted for 12 h. After completion, the mixture was condensed for the next step.

General synthesis for intermediate 7a-7c

To a solution of 4-fluoro-thalidomide (167.78 mg,0.61 mmol) dissolved in ethyl acetate (EA), **6a** (210.12 mg,0.96 mmol) dissolved in 15 ml EA and DIPEA (725.13 mg,3.66 mmol) were added at 70 °C. Then, the mixture was reacted at 80 °C under reflux overnight. After completion, yellow mixture was quenched by water and extracted by EA for three times. Organic layer was washed by saturated brine and dried over anhydrous Na_2SO_4 (5h). Yellow product (137.92 mg) was purified by silica gel column chromatography (30:1, DCM: MeOH).

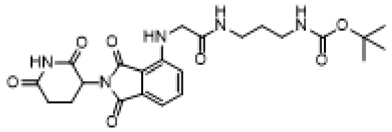

7a (yellow solid, 46.43%).^{1}H NMR (400 MHz, $CDCl_3$) δ 8.62 (s, 1H), 7.52 (t, *J* = 7.80 Hz, 1H), 7.18 (d, *J* = 7.13 Hz, 1H), 6.89 – 6.74 (m, 2H), 6.74 (d, *J* = 12.13 Hz, 1H), 4.99 – 4.86 (m, 2H), 3.96 (d, *J* = 6.02 Hz, 2H), 3.28 (q, *J* = 6.28 Hz, 2H), 3.07 (q, *J* = 6.36 Hz, 2H), 2.89 (d, *J* = 13.19 Hz, 1H), 2.83 – 2.59 (m, 2H), 2.21 – 2.07 (m, 1H), 1.58 (d, *J* = 6.34 Hz, 2H), 1.36 (s, 9H).^{13}C NMR (101 MHz, $CDCl_3$) δ 171.3, 169.3, 169.2, 168.6, 167.4, 156.6, 145.9, 136.4, 132.4, 116.9, 113.0, 111.5, 79.4, 49.0, 46.9, 37.0, 35.7, 31.4, 30.0, 28.3, 22.7.

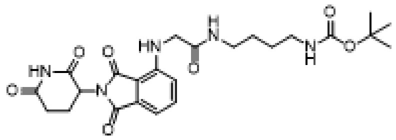

7b (yellow solid, 50.21%).^{1}H NMR (400 MHz, $CDCl_3$) δ 8.27 (s, 1H), 7.51 (dd, *J* = 8.48, 7.21 Hz, 1H), 7.18 (d, *J* = 7.18 Hz, 1H), 6.77 (d, *J* = 8.46 Hz, 1H), 6.62 (s, 2H), 4.90 (dd, *J* = 12.04, 5.33 Hz, 1H), 4.55 (s, 1H), 3.91 (d, *J* = 6.03 Hz, 2H), 3.25 – 3.17 (m, 2H), 3.04 (d, *J* = 6.69 Hz, 2H), 2.91 – 2.81 (m, 1H), 2.80 – 2.63 (m, 2H), 2.16 – 2.06 (m, 1H), 1.58 (s, 2H), 1.38 (s, 11H).^{13}C NMR (101 MHz, $CDCl_3$) δ 169.9, 168.3, 167.9, 167.4, 166.2, 155.1, 144.9, 135.6, 131.4, 116.1, 112.4, 110.7, 78.3, 48.1, 46.2, 39.0, 38.1, 30.4, 27.4, 26.5, 25.6, 21.7.

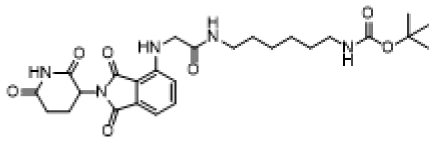

7c (yellow solid, 48.17%).^{1}H NMR (400 MHz, $CDCl_3$) δ 8.31 (s, 1H), 7.56 (dd, *J* = 8.46, 7.18 Hz, 1H), 6.82 (d, *J* = 8.43 Hz, 1H), 6.68 (t, *J* = 6.07 Hz, 1H), 6.60 (t, *J* = 5.95 Hz, 1H), 4.95 (dd, *J* = 12.11, 5.30 Hz, 1H), 3.96 (d, *J* = 6.02 Hz, 2H), 3.24 (d, *J* = 6.92 Hz, 2H), 3.06 (d, *J* = 6.90 Hz, 2H), 2.91 (dd, *J* = 15.27, 3.40 Hz, 1H), 2.89 – 2.69 (m, 2H), 2.19 – 2.11 (m, 1H), 1.61 (s, 2H), 1.43 (s, 9H), 1.31 – 1.22 (m, 6H).^{13}C NMR (101 MHz, $CDCl_3$) δ 170.9, 168.8, 168.4, 167.9, 167.3, 155.1, 145.9, 136.6, 132.4, 117.1, 113.4, 111.8, 78.1, 49.1, 47.3, 40.2, 39.1, 31.5, 29.9, 29.2, 28.4, 26.2, 26.0, 22.7.

General synthesis for intermediate 8a-8c

In a solution of **7a** dissolved in 4 ml DCM, TFA was added under stirring and reacted for 1 h at room temperature. Intermediate (white solid) was condensed for next step.

General synthesis for intermediate C01-C03

To a stirring condition on ice (0 °C), intermediate **8a** in DMF was added with DIPEA (138.62 mg,0.56 mmol) and DCA (8.62 mg,0.07 mmol). Half an hour later, HATU (24.49 mg,0.08 mmol) was added and reacted overnight. Then, reaction mixture was extracted by 30 ml DCM and washed with saturated brine. Anhydrous Na_2SO_4 was applied for drying the product. Yellow solid (14.37 mg) was harvested by silica gel column chromatography (30:1, DCM: MeOH).

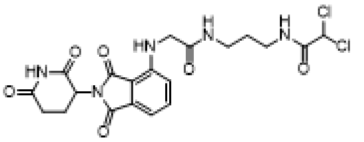

C01 (yellow solid, 41.24%). ^{1}H NMR (400 MHz, DMSO-d_6) δ 11.17 (s, 1H), 8.61 (t, J = 5.66 Hz, 1H), 8.19 (t, J = 5.70 Hz, 1H), 7.66 (dd, J = 8.50, 7.11 Hz, 1H), 7.14 (d, J = 7.06 Hz, 1H), 7.02 (t, J = 5.67 Hz, 1H), 6.94 (d, J = 8.52 Hz, 1H), 6.51 (s, 1H), 5.14 (dd, J = 12.89, 5.37 Hz, 1H), 4.00 (d, J = 5.65 Hz, 2H), 3.18 (qd, J = 6.94, 2.39 Hz, 4H), 2.96 (ddd, J = 17.92, 13.88, 5.35 Hz, 1H), 2.71 – 2.59 (m, 2H), 2.18 – 2.03 (m, 1H), 1.66 (p, J = 6.95 Hz, 2H). ^{13}C NMR (101 MHz, DMSO-d_6) δ 173.3, 170.5, 169.2, 169.1, 167.8, 164.0, 146.3, 136.7, 132.5, 117.9, 111.5, 110.4, 67.4, 49.0, 45.7, 37.6, 36.7, 31.5, 29.1, 22.6. HRMS (ESI): Calcd for $[M+H]^+$, $C_{20}H_{22}Cl_2N_5O_6$, 498.0947, found, 498.0962.

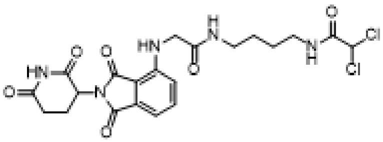

C02 (yellow solid, 47.37%). ^{1}H NMR (400 MHz, $CDCl_3$) δ 8.13 (s, 1H), 7.50 (dd, J = 8.44, 7.19 Hz, 1H), 7.17 (d, J = 7.19 Hz, 1H), 6.75 (d, J = 8.46 Hz, 1H), 6.61 (s, 2H), 5.86 (s, 1H), 4.88 (dd, J = 12.16, 5.29 Hz, 1H), 3.90 (d, J = 5.37 Hz, 2H), 3.24 (s, 4H), 2.90 –

2.79 (m, 1H), 2.78 – 2.65 (m, 2H), 2.13 – 2.04 (m, 1H), 1.51 – 1.45 (m, 4H).^{13}C NMR (101 MHz, $CDCl_3$) δ 170.8, 169.3, 168.9, 168.5, 167.2, 164.4, 145.8, 136.6, 132.4, 117.0, 113.6, 111.7 66.5, 49.1, 47.2, 39.8, 38.9, 31.4, 26.6, 26.3, 22.7. HRMS (ESI): Calcd for $[M+H]^+$, $C_{21}H_{24}Cl_2N_5O_6$, 512.1104, found, 512.1131.

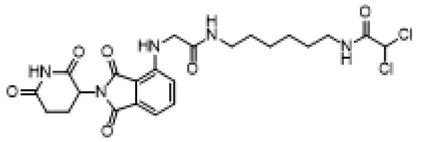

C03 (yellow solid, 35.58%).^{1}H NMR (400 MHz, $CDCl_3$)δ 8.29 (s, 1H), 7.55 (q, J = 7.84 Hz, 1H), 7.24 (d, J = 7.28 Hz, 1H), 6.82 (d, J = 8.46 Hz, 1H), 6.70 (t, J = 6.05 Hz, 1H), 6.62 (d, J = 6.02 Hz, 2H), 5.95 (s, 1H), 4.95 (dd, J = 12.07, 5.35 Hz, 1H), 4.48 (s, 1H), 3.97 (d, J = 6.04 Hz, 2H), 3.26 (p, J = 6.91 Hz, 4H), 2.98 – 2.84 (m, 1H), 2.83 – 2.67 (m, 2H), 2.22 – 2.06 (m, 1H), 1.61 (s, 4H), 1.56 – 1.30 (m, 4H).^{13}C NMR (101 MHz, $CDCl_3$) δ 170.9, 169.3, 168.9, 168.5, 167.3, 164.3, 145.9, 136.6, 132.4, 117.1, 113.4, 111.7, 66.6, 49.1, 47.3, 39.8, 38.9, 31.4, 29.2, 28.9, 25.8, 25.7, 22.7. HRMS (ESI): Calcd for $[M+H]^+$, $C_{23}H_{28}Cl_2N_5O_6$, 540.1417, found, 540.1435.

Synthesis for series-D compounds

Synthesis for intermediate 9

Under a stirring condition, triethylamine (406.89 mg, 4.02 mmol) was added in 2,6-dioxopiperidine-3-ammonium chloride (243.12 mg,1.48 mmol) in tetrahydrofuran (THF). After 0.5 h, 2-hydroxyphthalic anhydride (220.34 mg,1.34 mmol), 4-dimethylaminopyridine (16.15 mg,0.13 mmol) and N, N-dicyclohexylcarbodiimide (332.45 mg,1.61 mmol) were added and reacted at 90 °C under reflux for 10 h. After completion, cool down the reaction system and filter the residue. After solvent evaporation, off-white powder (70.25 mg) was collected by silica gel column chromatography (40:1, DCM: MeOH).

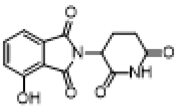

9 (off-white powder, 46.37%). ^{1}H NMR (400 MHz, DMSO-d_6) δ 11.18 (s, 1H), 11.08 (s, 1H), 7.65 (dd, J = 8.39, 7.18 Hz, 1H), 7.32 (d, J = 7.13 Hz, 1H), 7.25 (d, J = 8.37 Hz, 1H), 5.07 (dd, J = 12.90, 5.34 Hz, 1H), 2.89 (ddd, J = 17.26, 14.06, 5.40 Hz, 1H), 2.61 (t, J = 3.48 Hz, 1H), 2.55 (d, J = 9.94 Hz, 1H), 2.17 – 1.92 (m, 1H). ^{13}C NMR (101 MHz, DMSO-d_6) δ 173.3, 170.5, 167.5, 166.3, 156.0, 136.9, 133.6, 124.1, 114.8, 114.7, 49.1, 31.4, 22.5.

Synthesis for intermediate 10

9 (50.11 mg,0.20 mmol), tert-butyl bromoacetate (42.67 mg,0.24 mmol), KI (3.02 mg,0.02 mmol) and $KHCO_3$ (37.80 mg,0.30 mmol) were dissolved in DMF and heated to 60 °C over a period of 5 h. Then, residue was extracted by 30 ml DCM, washed with saturated brine and dried over anhydrous Na_2SO_4. White solid (43.91 mg) was harvested by silica gel column chromatography (30:1, DCM: MeOH).

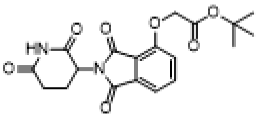

10 (white solid, 56.59%) ^{1}H NMR (400 MHz, $CDCl_3$)δ 8.01 (s, 1H), 7.60 (dd, J = 8.48, 7.29 Hz, 1H), 7.44 (d, J = 7.25 Hz, 1H), 7.04 (d, J = 8.48 Hz, 1H), 4.95 – 4.86 (m, 1H), 4.72 (s, 2H), 2.88 – 2.79 (m, 1H), 2.78 – 2.65 (m, 2H), 2.13 – 2.01 (m, 1H), 1.41 (s, 9H). ^{13}C NMR (101 MHz, $CDCl_3$)δ 169.9, 166.9, 165.8, 165.8,164.4, 154.5, 135.3, 132.9, 118.8, 116.6, 115.9, 82.1, 65.5, 48.2, 30.4, 27.0, 21.6.

Synthesis for intermediate 11

TFA (1ml) was added to H-B dissolved in DCA and reacted for 6 h. After solvent evaporation, white solid was collected for next step.

General synthesis for 12a-12e

To a stirring solution on ice (0 °C) containing with H-C_1 (200.00 mg,0.62 mmol) in DMF, DIPEA and tert-butyl (3-aminopropyl) carbamate (129.80 mg,0.72 mmol) were added. Half an hour later, HATU (259.71 mg,0.70 mmol) was added and reacted overnight. Then, reaction mixture was extracted by 30 ml DCM and washed with saturated brine. Anhydrous Na_2SO_4 was applied for drying the product. White solid (201.26 mg) was collected by silica gel column chromatography (30:1, DCM: MeOH).

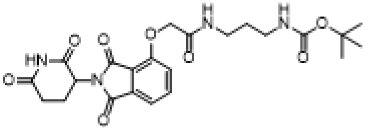

12a (white solid, 66.52%).^{1}H NMR (400 MHz, $CDCl_3$) δ 8.32 (s, 1H), 7.76 (t, *J* = 7.88 Hz, 1H), 7.66 (s, 1H), 7.57 (d, *J* = 7.36 Hz, 1H), 7.22 (d, *J* = 8.40 Hz, 1H), 5.02 – 4.93 (m, 1H), 4.74 – 4.60 (m, 2H), 3.43 (tt, *J* = 13.06, 6.84 Hz, 2H), 2.93 (d, *J* = 14.01 Hz, 1H), 2.81 – 2.69 (m, 2H), 2.23 – 2.14 (m, 1H), 1.75 (p, *J* = 6.67 Hz, 2H), 1.43 (s, 9H).^{13}C NMR (101 MHz, $CDCl_3$) δ 170.7, 167.1, 166.5, 154.8, 137.1, 133.5, 120.3, 117.8, 79.4, 68.7, 49.4, 37.6, 36.3, 31.4, 29.8, 28.4, 22.7.

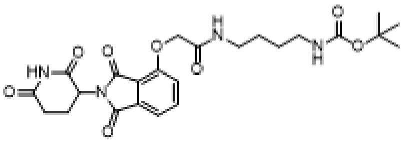

12b (white solid, 68.42%).^{1}H NMR (400 MHz, DMSO-d_6) δ 11.12 (s, 1H), 7.96 (t, *J* = 5.70 Hz, 1H), 7.86 – 7.77 (m, 1H), 7.50 (d, *J* = 7.26 Hz, 1H), 7.39 (d, *J* = 8.52 Hz, 1H), 6.78 (t, *J* = 5.78 Hz, 1H), 5.13 (dd, *J* = 12.96, 5.38 Hz, 1H), 4.77 (s, 2H), 3.14 (q, *J* = 6.29 Hz, 2H), 2.90 (p, *J* = 7.16 Hz, 3H), 2.67 – 2.54 (m, 2H), 2.10 – 1.97 (m, 1H), 1.37 (s, 13H).^{13}C NMR (101 MHz, DMSO-d_6) δ 173.2, 170.3, 167.2, 167.1, 166.0, 156.1, 155.6, 137.4, 133.5, 120.9, 117.3, 116.5, 77.8, 68.1, 55.4, 49.3, 38.6, 31.4, 28.7, 27.4, 26.9, 22.5.

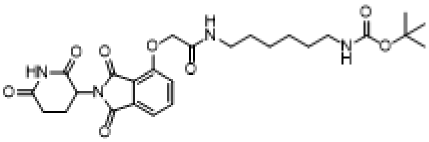

12c (white solid, 72.52%).^{1}H NMR (400 MHz, $CDCl_3$) δ 8.86 (s, 1H), 7.75 (t, *J* = 7.85 Hz, 1H), 7.55 (d, *J* = 7.32 Hz, 1H), 7.49 – 7.42 (m, 1H), 7.20 (d, *J* = 8.38 Hz,

1H), 5.03 – 4.94 (m, 1H), 4.64 (s, 2H), 3.49 – 3.26 (m, 2H), 3.23 – 2.98 (m, 2H), 2.98 – 2.71 (m, 3H), 2.21 – 2.11 (m, 1H), 1.68 (s, 2H), 1.64 – 1.58 (m, 2H), 1.44 (s, 13H).^{13}C NMR (101 MHz, $CDCl_3$) δ 171.1, 168.1, 166.6, 166.1, 154.5, 137.1, 133.6, 119.6, 118.2, 117.4, 68.1, 49.3, 40.5, 39.1, 31.5, 30.0, 29.1, 28.4, 26.5, 22.7.

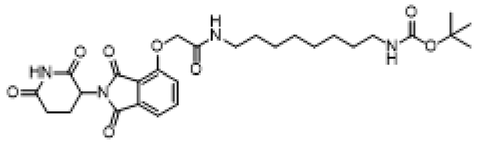

12d (white solid, 3.55%). NMR (400 MHz, $CDCl_3$) δ 8.49 (s, 1H), 7.68 (dd, *J* = 8.40, 7.33 Hz, 1H), 7.48 (d, *J* = 7.30 Hz, 1H), 7.32 (t, *J* = 5.60 Hz, 1H), 7.13 (d, *J* = 8.38 Hz, 1H), 4.95 – 4.85 (m, 1H), 4.57 (d, *J* = 1.48 Hz, 2H), 4.53 (s, 1H), 3.30 (q, *J* = 6.69 Hz, 2H), 3.02 (q, *J* = 6.87 Hz, 2H), 2.91 – 2.81 (m, 1H), 2.82 – 2.64 (m, 2H), 2.09 (ddd, *J* = 10.00, 4.43, 2.36 Hz, 1H), 1.52 (p, *J* = 6.98 Hz, 2H), 1.37 (s, 13H), 1.32 – 1.22 (m, 6H).^{13}C NMR (101 MHz, $CDCl_3$) δ 169.9, 167.0, 165.6, 165.6, 164.9, 155.0, 153.5, 136.0, 132.6, 118.5, 117.1, 116.3, 78.1, 67.0, 48.3, 39.5, 38.2, 30.5, 29.0, 28.2, 28.1, 27.4, 25.7, 21.6.

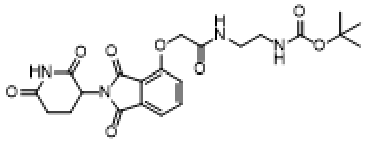

12e (white solid, 76.67%).^{1}H NMR (400 MHz, $CDCl_3$) δ 8.18 (s, 1H), 7.68 (dd, *J* = 8.38, 7.38 Hz, 1H), 7.65 (s, 1H), 7.50 (d, *J* = 7.26 Hz, 1H), 7.15 (d, *J* = 8.37 Hz, 1H), 4.91 (dt, *J* = 12.11, 6.16 Hz, 1H), 4.60 (d, *J* = 3.73 Hz, 2H), 3.43 (s, 3H), 3.26 (d, *J* = 6.20 Hz, 2H), 2.95 – 2.81 (m, 1H), 2.70 (dd, *J* = 15.29, 4.17 Hz, 1H), 2.33 – 1.99 (m, 1H), 1.34 (s, 9H).^{13}C NMR (101 MHz, $CDCl_3$) δ 170.7, 167.9, 167.5, 166.5, 156.1, 154.8, 137.1, 133.5, 120.3, 118.5, 117.7, 79.5, 68.7, 49.4, 40.4, 39.5, 31.4, 28.4, 22.6.

General synthesis for intermediate 13a-13e

In a solution of H-D_1 dissolved in 4 ml DCM, TFA was added under stirring and reacted for 1 h at room temperature. Intermediate (white solid) was condensed for next step.

General synthesis for D01-D05

To a stirring condition on ice (4 °C), **13a** (23.31 mg,0.06 mmol) in DMF was added in DIPEA (46.52 mg,0.36 mmol) and DCA (7.72 mg,0.06 mmol). Half an hour later, HATU (400.12 mg,1.06 mmol) was added and reacted overnight. Then, residue was extracted by 30 ml DCM and washed with saturated brine. Anhydrous Na_2SO_4 was applied for drying the product. White solid 10.31 mg) was harvested by silica gel column chromatography (30:1, DCM: MeOH).

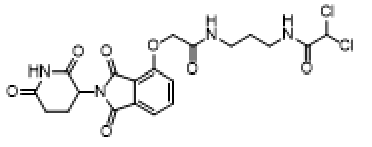

D01 (white solid, 34.52%) ^{1}H NMR (400 MHz, DMSO-*d6*) δ 11.17 (s, 1H), 8.63 (t, *J* = 5.66 Hz, 1H), 8.09 (t, *J* = 5.80 Hz, 1H), 7.88 (dd, *J* = 8.51, 7.26 Hz, 1H), 7.56 (d, *J* = 7.28 Hz, 1H), 7.46 (d, *J* = 8.52 Hz, 1H), 6.51 (s, 1H), 5.18 (dd, *J* = 12.96, 5.35 Hz, 1H), 4.85 (s, 2H), 2.96 (ddd, *J* = 17.33, 14.01, 5.37 Hz, 1H), 2.64 (td, *J* = 13.72, 4.15 Hz, 2H), 2.14 – 2.06 (m, 1H), 1.69 (q, *J* = 6.99 Hz, 2H).^{13}C NMR (101 MHz, DMSO-*d6*) δ 173.2, 170.4, 167.4, 167.2, 165.9, 164.1, 155.6, 137.4, 133.5, 120.9, 117.3, 116.5, 68.1, 67.4, 49.3, 37.6, 36.5, 31.4, 29.1, 22.5. HRMS (ESI): Calcd for $[M+H]^+$, $C_{20}H_{21}Cl_2N_4O_7$, 499.0787, found, 499.0798.

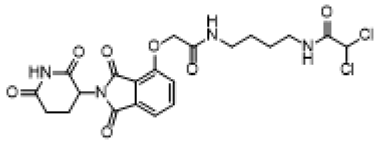

D02(white solid, 35.57%)^{1}H NMR (400 MHz, $CDCl_3$) δ 8.28 (s, 1H), 7.75 (dd, *J* = 8.39, 7.38 Hz, 1H), 7.56 (d, *J* = 7.26 Hz, 1H), 7.40 (s, 1H), 7.20 (d, *J* = 8.40 Hz, 1H), 6.52 (s, 1H), 5.94 (s, 1H), 4.98 (dd, *J* = 12.12, 5.36 Hz, 1H), 4.64 (s, 2H), 3.42 – 3.27 (m, 4H), 3.01 – 2.89 (m, 1H), 2.81 (td, *J* = 15.87, 8.57 Hz, 2H), 2.16 (dd, *J* = 10.42, 5.42 Hz, 1H), 1.66 – 1.47 (m, 2H), 1.34 (s, 2H).^{13}C NMR (101 MHz, $CDCl_3$) δ 170.8, 167.9, 166.6, 166.0, 164.2, 154.5, 137.1, 133.6, 119.5, 118.1, 117.4, 68.0, 66.5, 49.3, 40.3, 39.2, 31.5, 29.2, 29.0, 22.6. HRMS (ESI): Calcd for $[M+H]^+$, $C_{21}H_{23}Cl_2N_4O_7$, 513.0944, found, 513.0963.

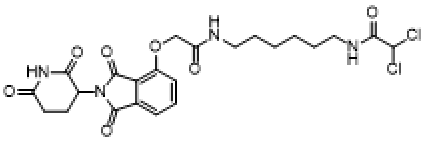

D03 (white solid, 33.34%)^{1}H NMR (400 MHz, DMSO-d_6) δ 11.11 (s, 1H), 8.53 (s, 1H), 7.93 (t, *J* = 5.70 Hz, 1H), 7.81 (dd, *J* = 8.51, 7.32 Hz, 1H), 7.50 (d, *J* = 7.21 Hz, 1H), 7.39 (d, *J* = 8.48 Hz, 1H), 6.42 (s, 1H), 5.12 (dd, *J* = 12.84, 5.42 Hz, 1H), 4.77 (s, 2H), 3.12 (dq, *J* = 13.08, 6.66 Hz, 4H), 2.90 (ddd, *J* = 16.96, 13.81, 5.28 Hz, 1H), 2.58 (td, *J* = 14.31, 3.92 Hz, 2H), 2.08 – 1.99 (m, 1H), 1.42 (d, *J* = 7.99 Hz, 4H), 1.30 – 1.22 (m, 4H).^{13}C NMR (101 MHz, DMSO-d_6) δ 173.3, 170.4, 167.2, 167.1, 166.0, 163.9, 155.5, 137.4, 133.5, 120.9, 117.3, 116.5, 68.1, 67.4, 49.3, 39.7, 38.7, 31.4, 29.4, 28.9, 26.4, 26.3, 22.5. HRMS (ESI): Calcd for $[M+H]^+$, $C_{23}H_{27}Cl_2N_4O_7$, 541.1257, found, 541.1273.

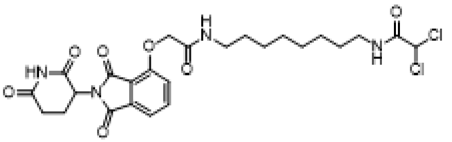

D04 (white solid, 38.54%) ^{1}H NMR (400 MHz, DMSO-d_6) δ 11.18 (s, 1H), 8.60 (t, *J* = 5.63 Hz, 1H), 7.99 (t, *J* = 5.76 Hz, 1H), 7.87 (dd, *J* = 8.51, 7.31 Hz, 1H), 7.56 (d, *J* = 7.20 Hz, 1H), 7.45 (d, *J* = 8.56 Hz, 1H), 6.48 (s, 1H), 5.18 (dd, *J* = 12.93, 5.35 Hz, 1H), 4.83 (s, 2H), 3.26 – 3.16 (m, 4H), 2.96 (ddd, *J* = 17.20, 13.95, 5.42 Hz, 1H), 2.71 – 2.58 (m, 2H), 2.10 (dtd, *J* = 12.99, 5.47, 2.50 Hz, 1H), 1.49 (t, *J* = 6.93 Hz, 4H), 1.31 (d, *J* = 6.83 Hz, 8H).^{13}C NMR (101 MHz, DMSO-d_6) δ 173.2, 170.3, 167.2, 167.1, 166.0, 163.9, 155.5, 137.4, 133.5, 120.9, 117.3, 116.5, 68.1, 67.4, 49.3, 41.6, 38.8, 31.4, 29.4, 29.1, 29.0, 28.9, 26.7, 26.6,22.5. HRMS (ESI): Calcd for $[M+H]^+$, $C_{25}H_{31}Cl_2N_4O_7$, 569.1570, found, 569.1594.

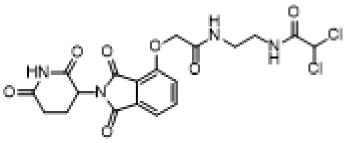

D05 (white solid, 44.85%) ^{1}H NMR (400 MHz, DMSO-d_6) δ 11.18 (s, 1H), 8.70 (s, 1H), 8.16 (d, *J* = 5.97 Hz, 1H), 7.88 (dd, *J* = 8.55, 7.31 Hz, 1H), 7.57 (d, *J* = 7.26 Hz, 1H), 7.45 (d, *J* = 8.52 Hz, 1H), 6.48 (s, 1H), 5.18 (dd, *J* = 12.92, 5.43 Hz, 1H), 4.84 (s, 2H), 3.36 –

3.27 (m, 4H), 3.03 – 2.89 (m, 1H), 2.77 – 2.61 (m, 2H), 2.14 – 2.03 (m, 1H). ^{13}C NMR (101 MHz, DMSO-d_6) δ 173.3, 170.3, 167.7, 167.2, 165.9, 164.4, 155.6, 137.5, 133.5, 121.0, 117.3, 116.6, 68.1, 67.3, 49.3, 38.1, 31.4, 22.5. HRMS (ESI): Calcd for $[M+H]^+$, $C_{19}H_{19}Cl_2N_4O_7$, 485.0631, found, 485.0645.

NMR Spectrum

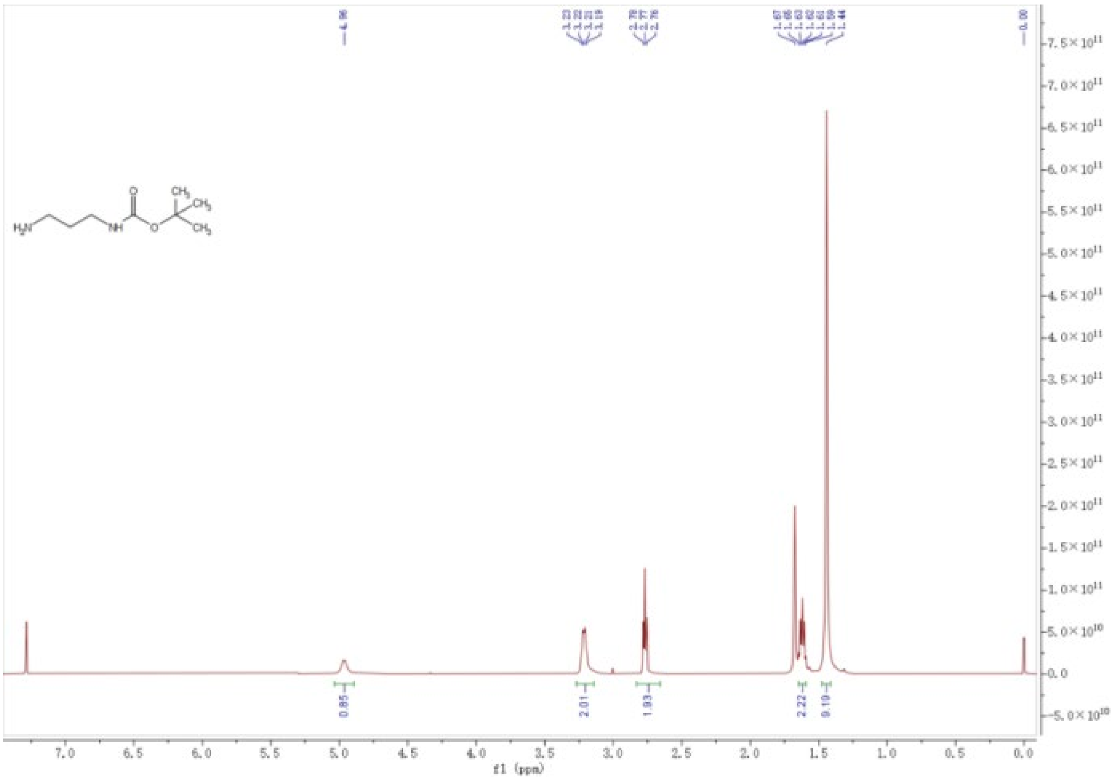

Figure S9 ^{1}H-NMR spectrum of 1a

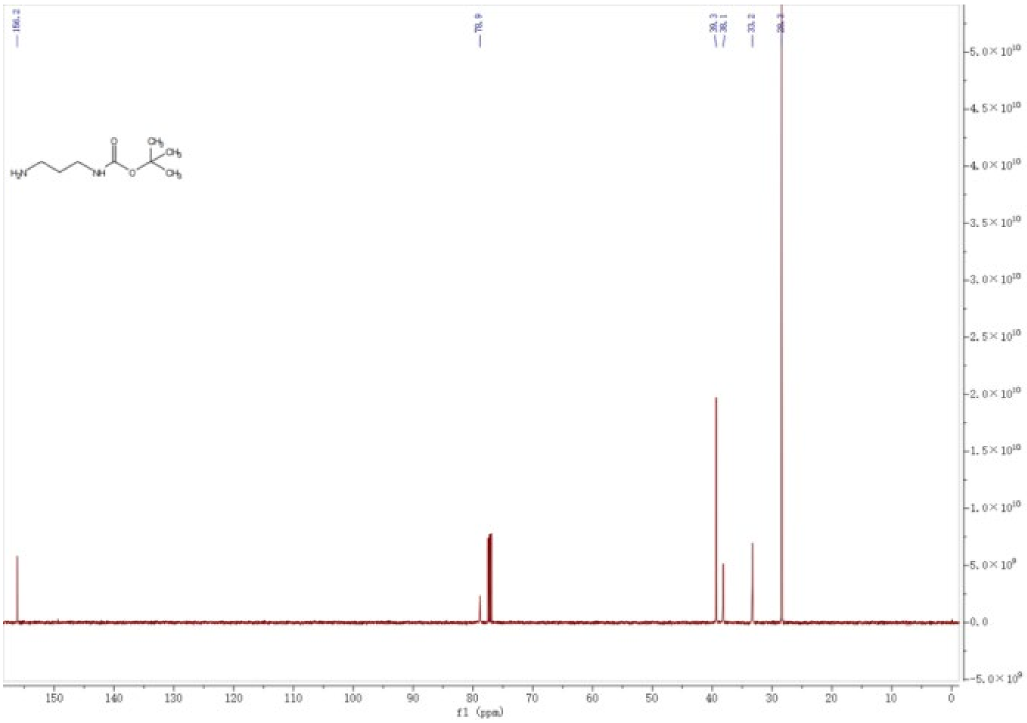

Figure S10 ^{13}C NMR spectrum of 1a

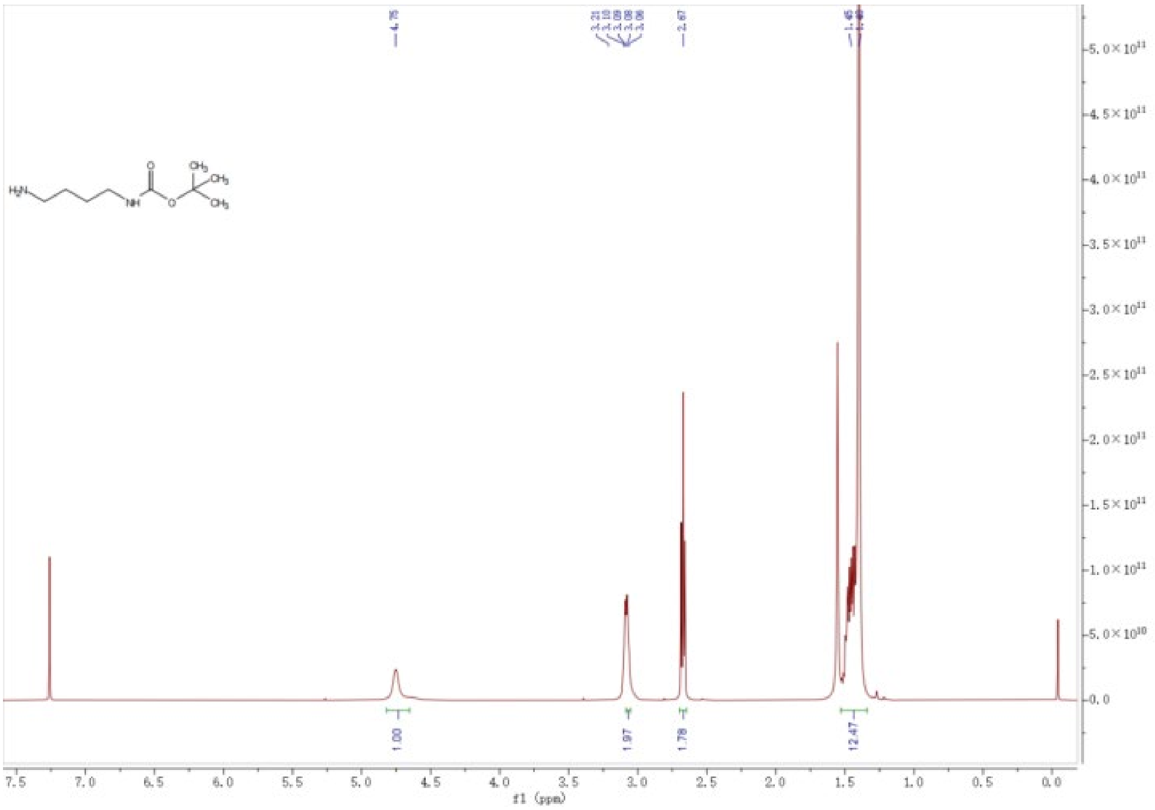

Figure S11 ^{1}H-NMR spectrum of 1b

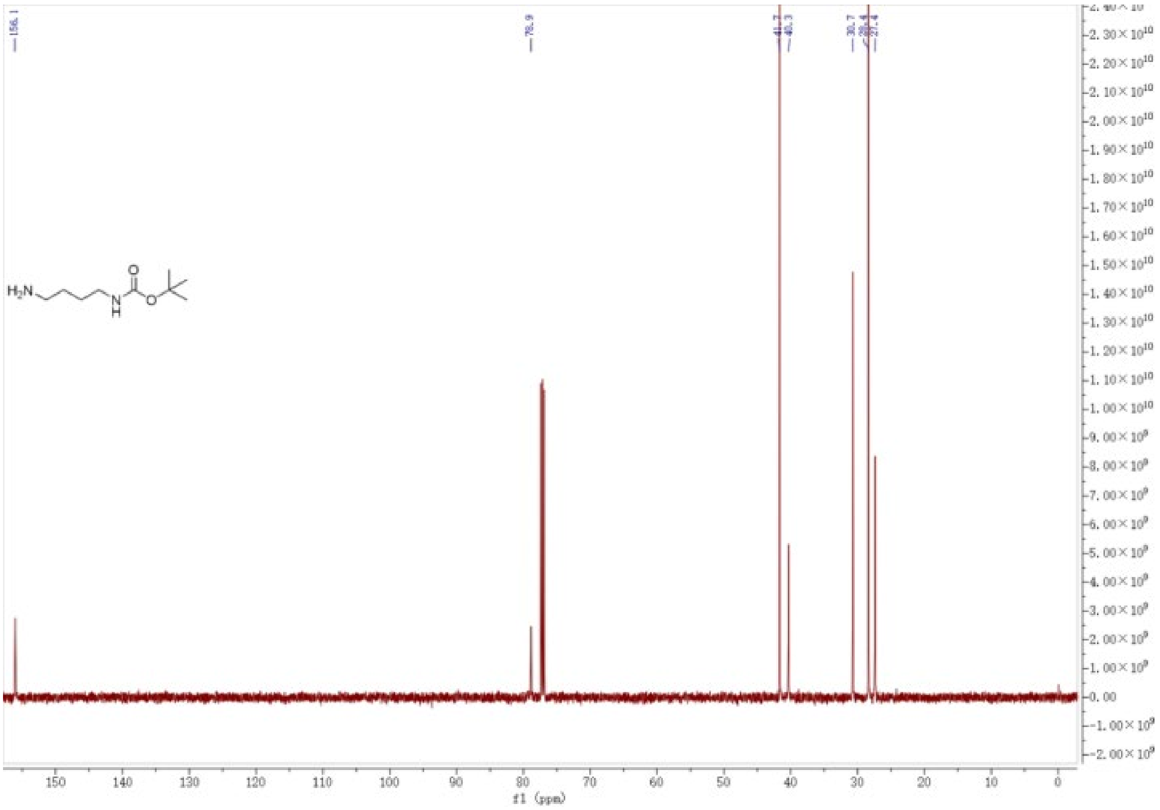

Figure S12 ^{13}C NMR spectrum of 1b

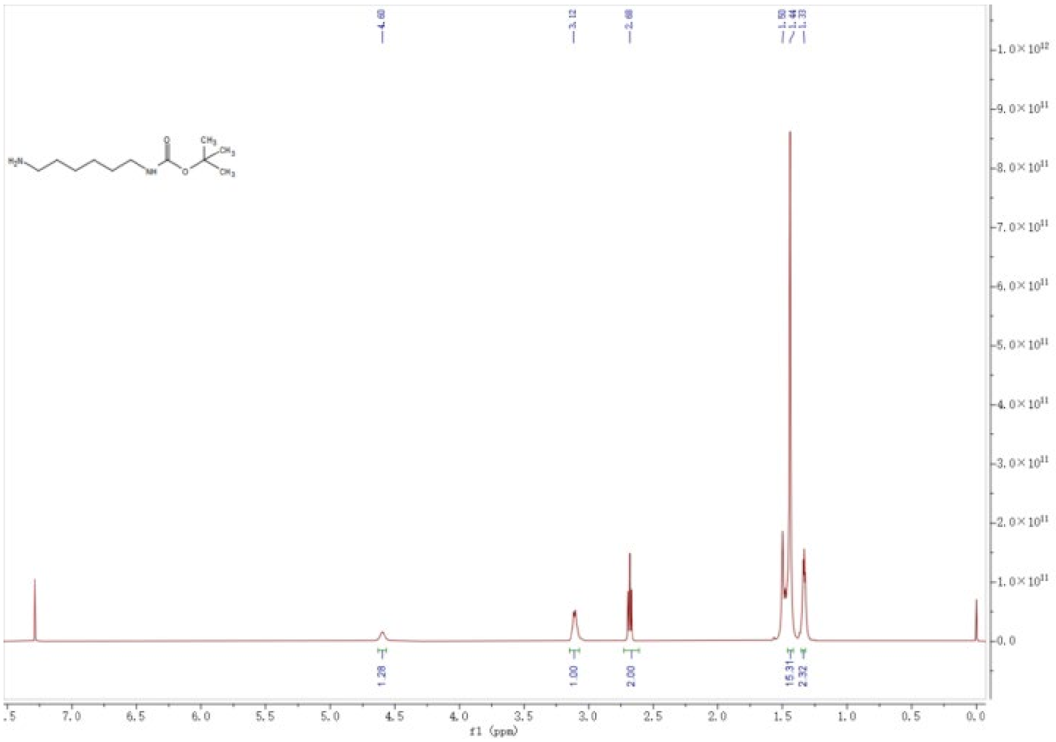

Figure S13 ^{1}H-NMR spectrum of 1c

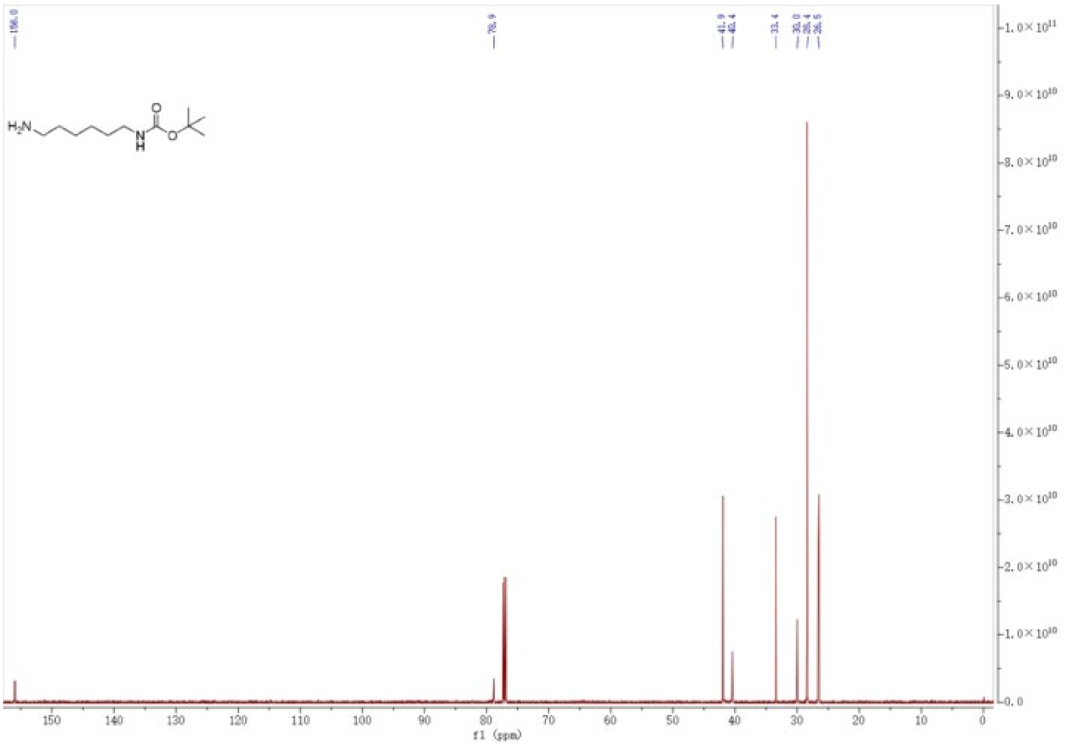

Figure S13 ^{13}C NMR spectrum of 1c

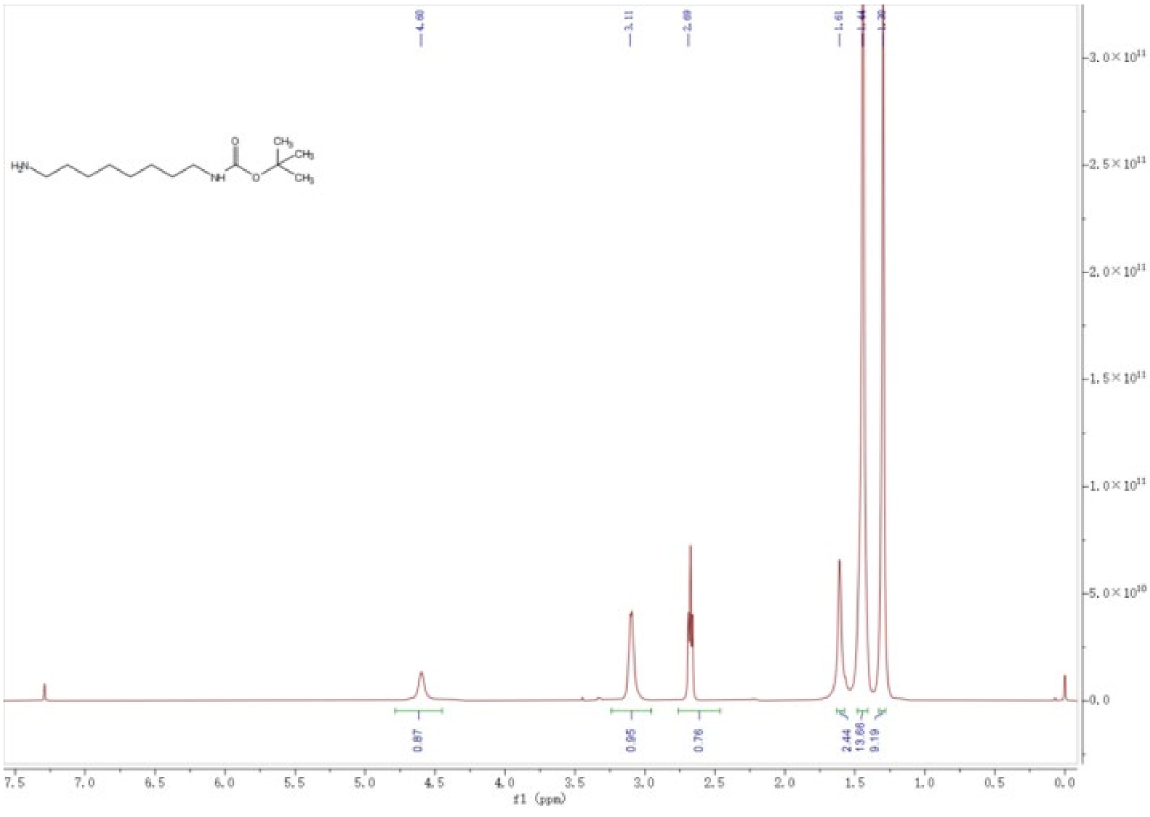

Figure S14 ^{1}H-NMR spectrum of 1d

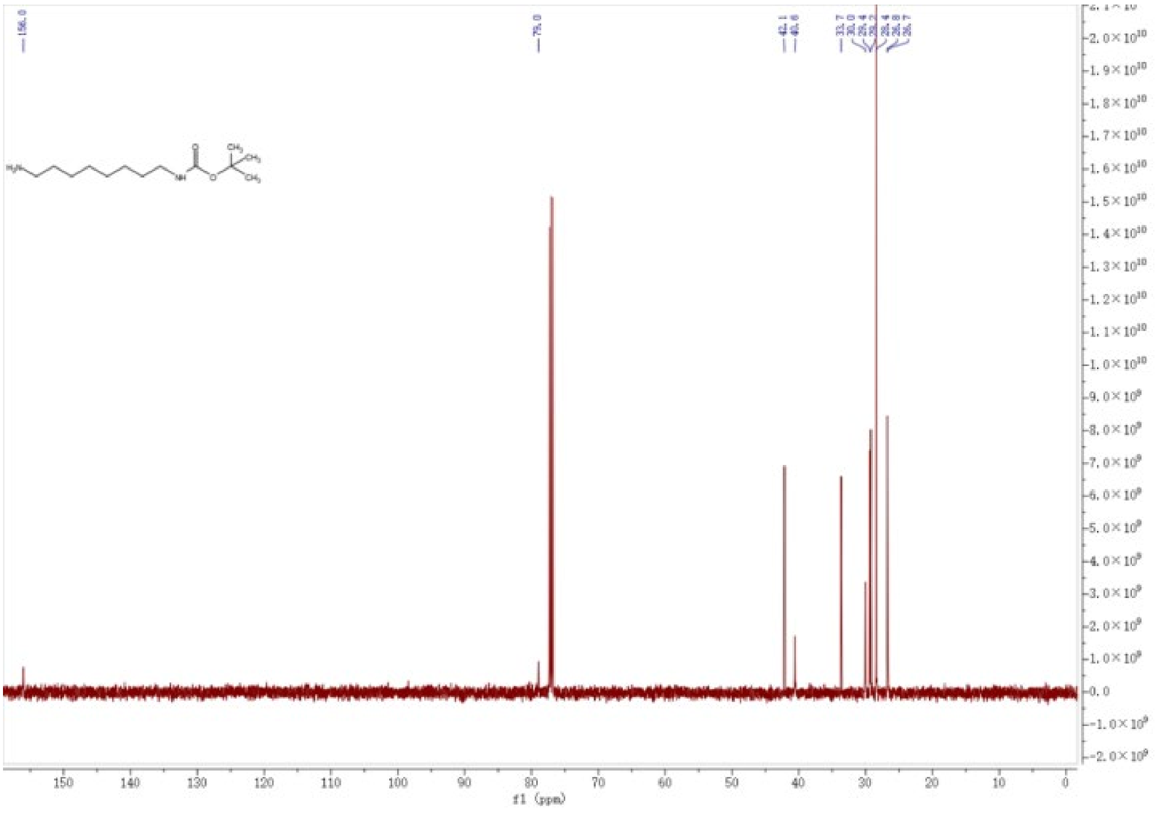

Figure S15 ^{13}C NMR spectrum of 1d

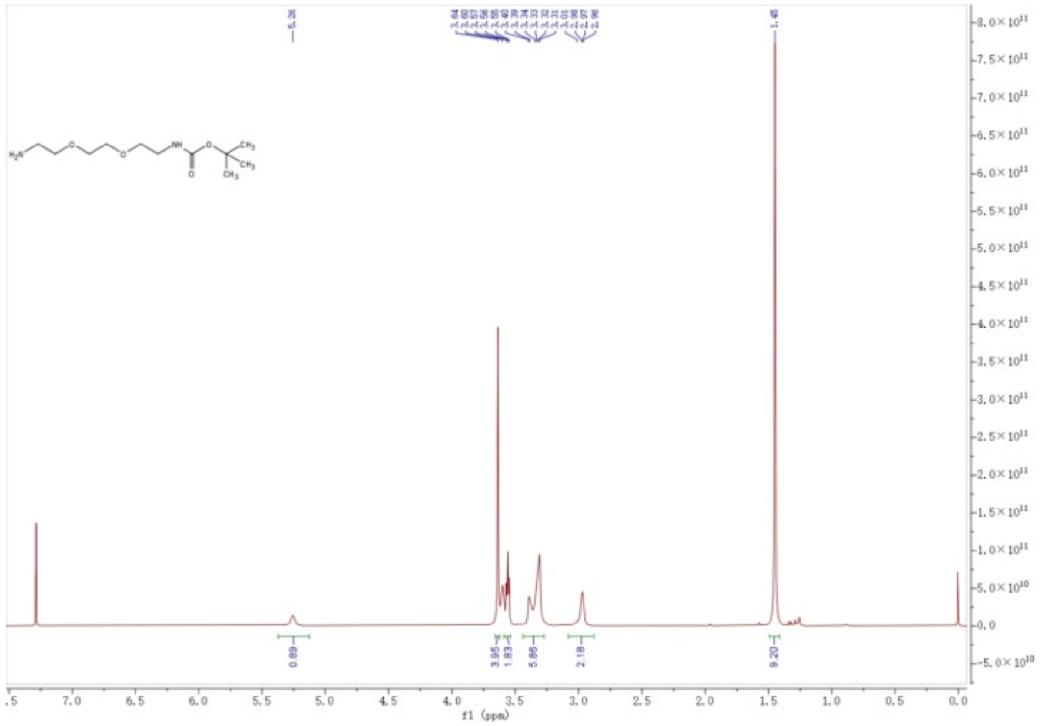

Figure S16 ^{1}H-NMR spectrum of 1e

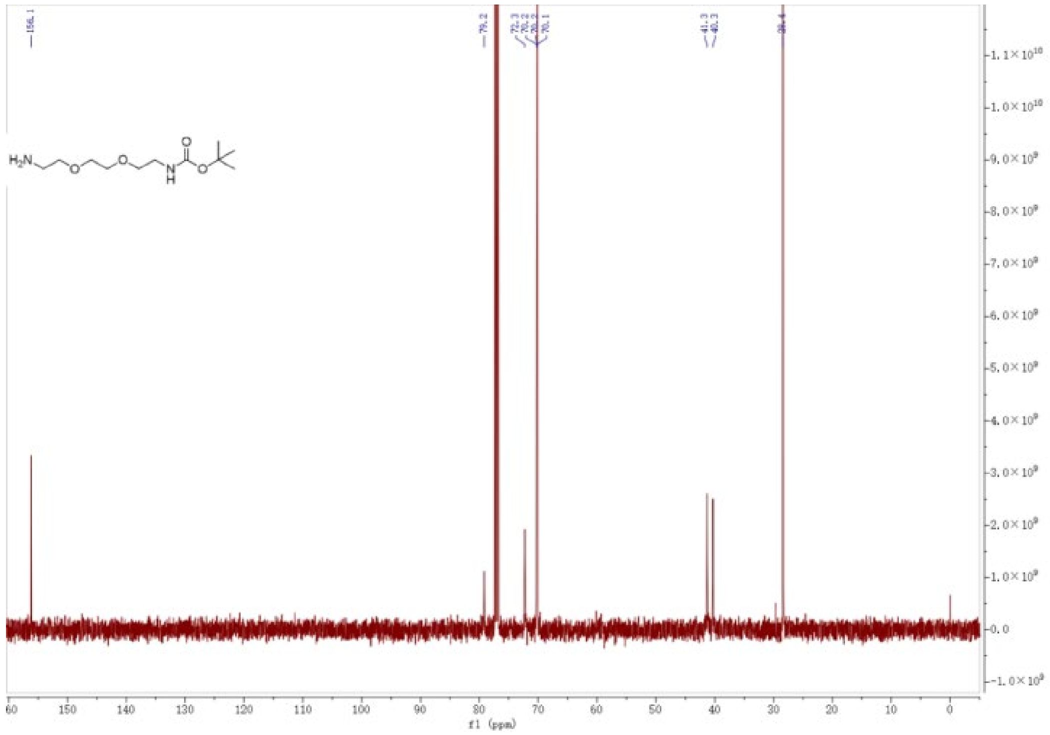

Figure S17 ^{13}C NMR spectrum of 1e

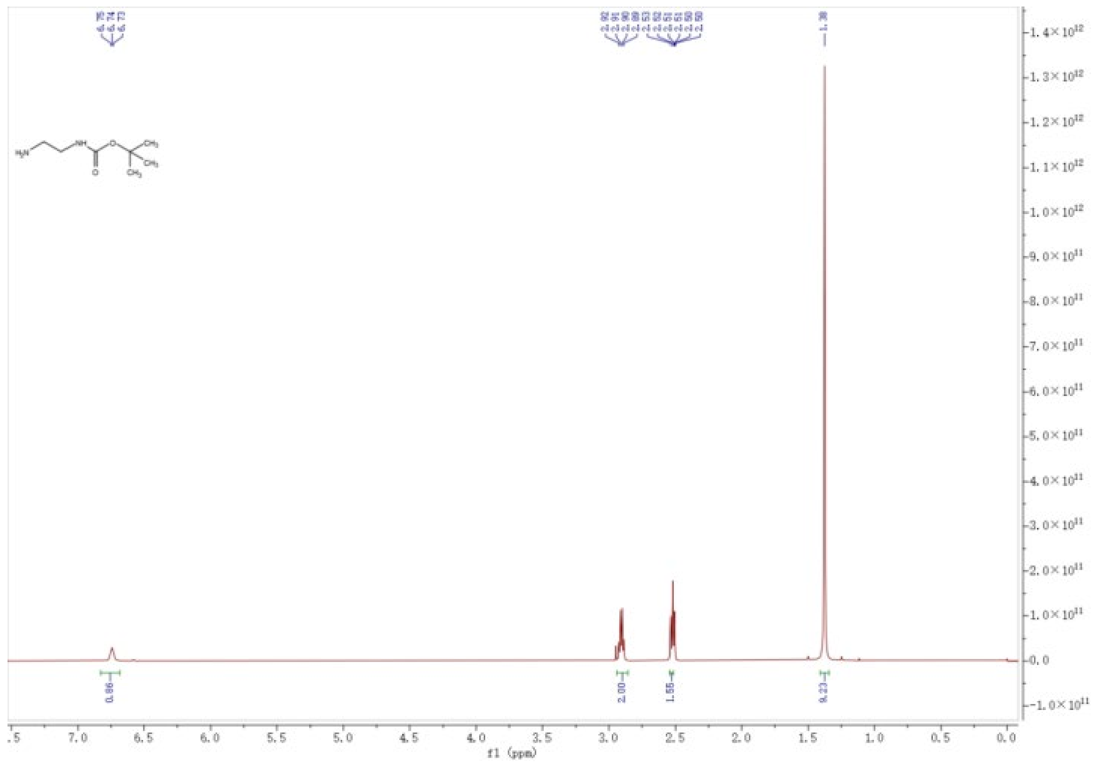

Figure S18 ^{1}H-NMR spectrum of 1f

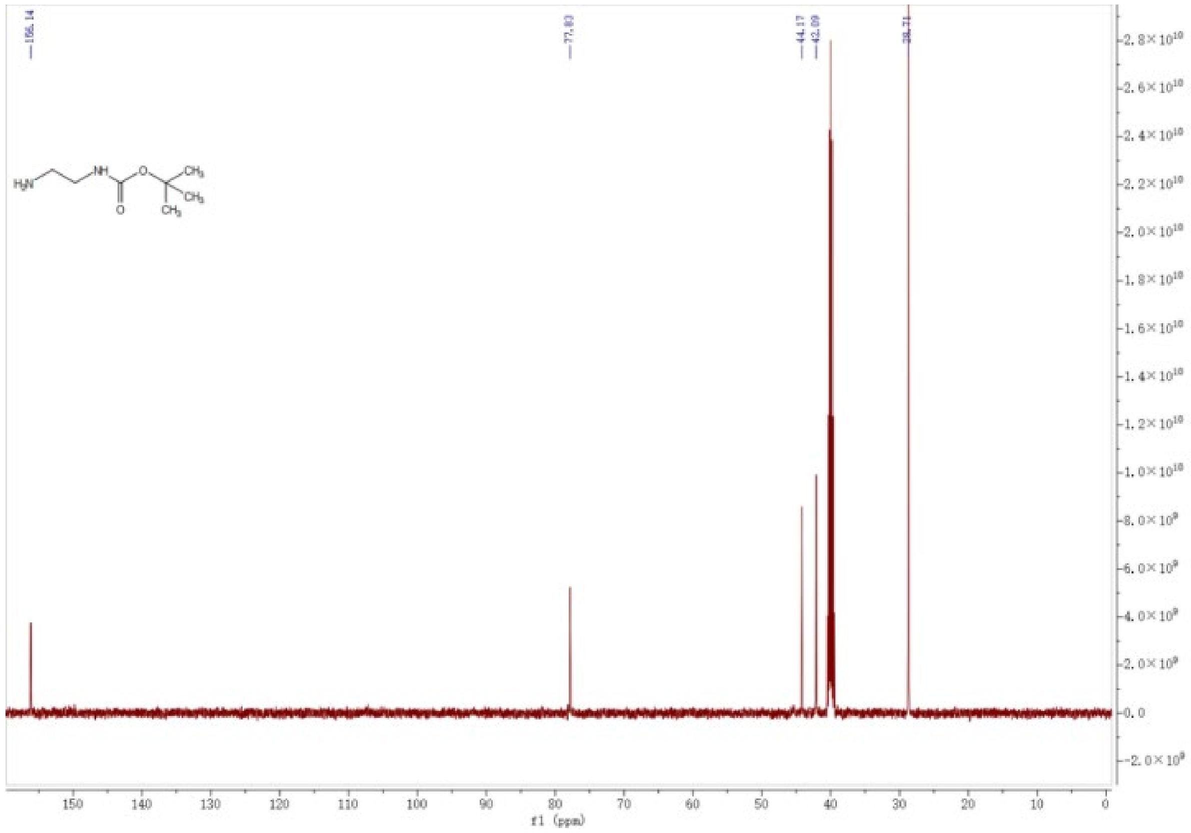

Figure S19 ^{13}C NMR spectrum of 1f

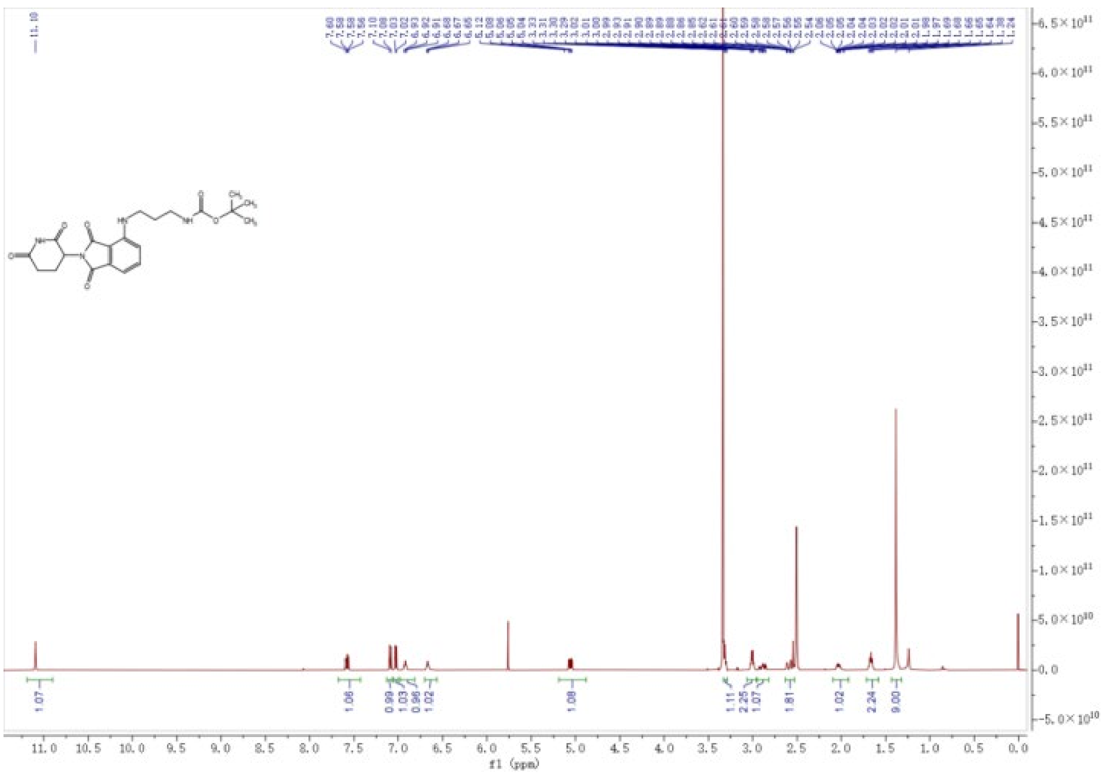

Figure S20 ^{1}H-NMR spectrum of 2a

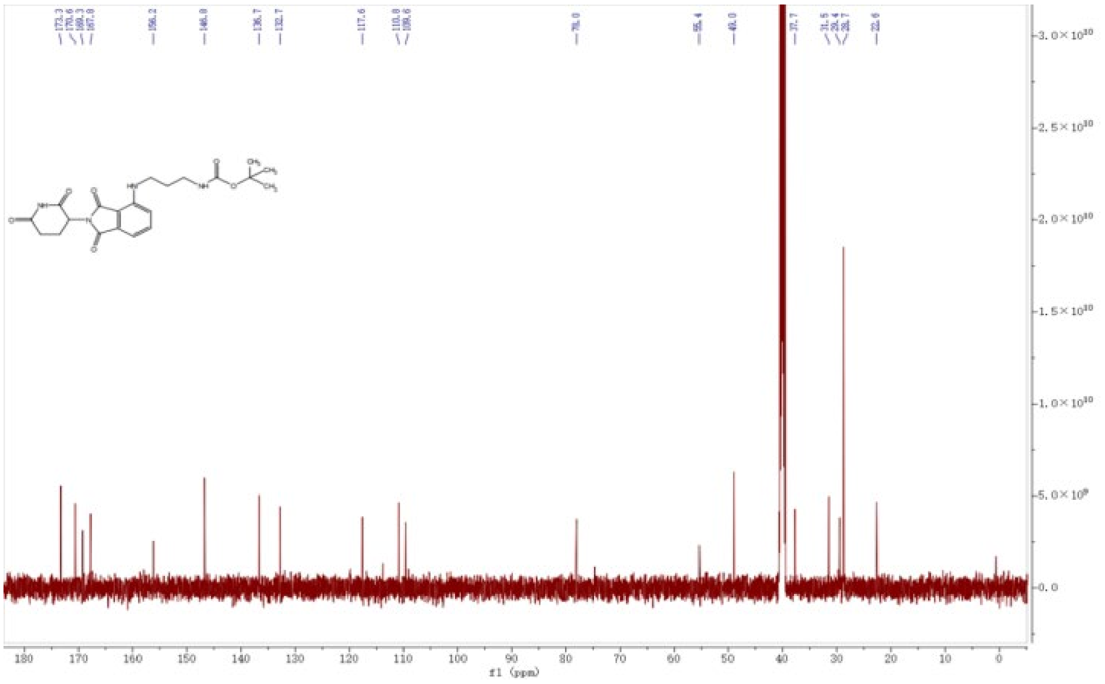

Figure S21 ^{13}C NMR spectrum of 2a

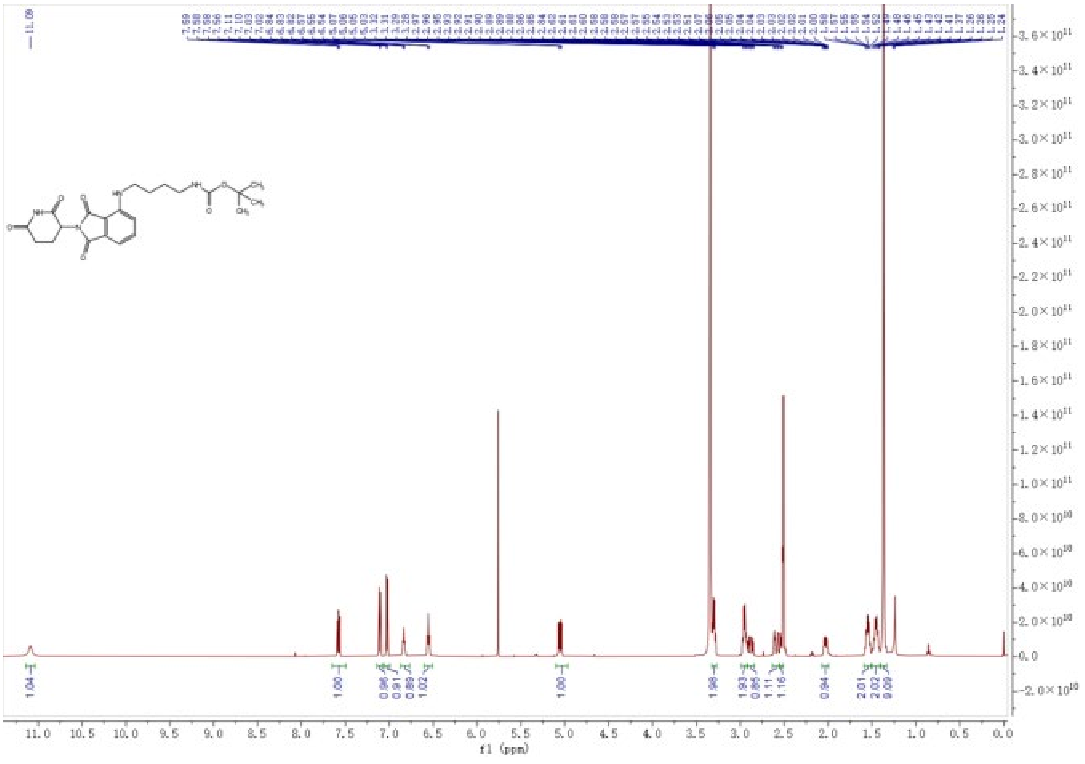

Figure S22 ^{1}H-NMR spectrum of 2b

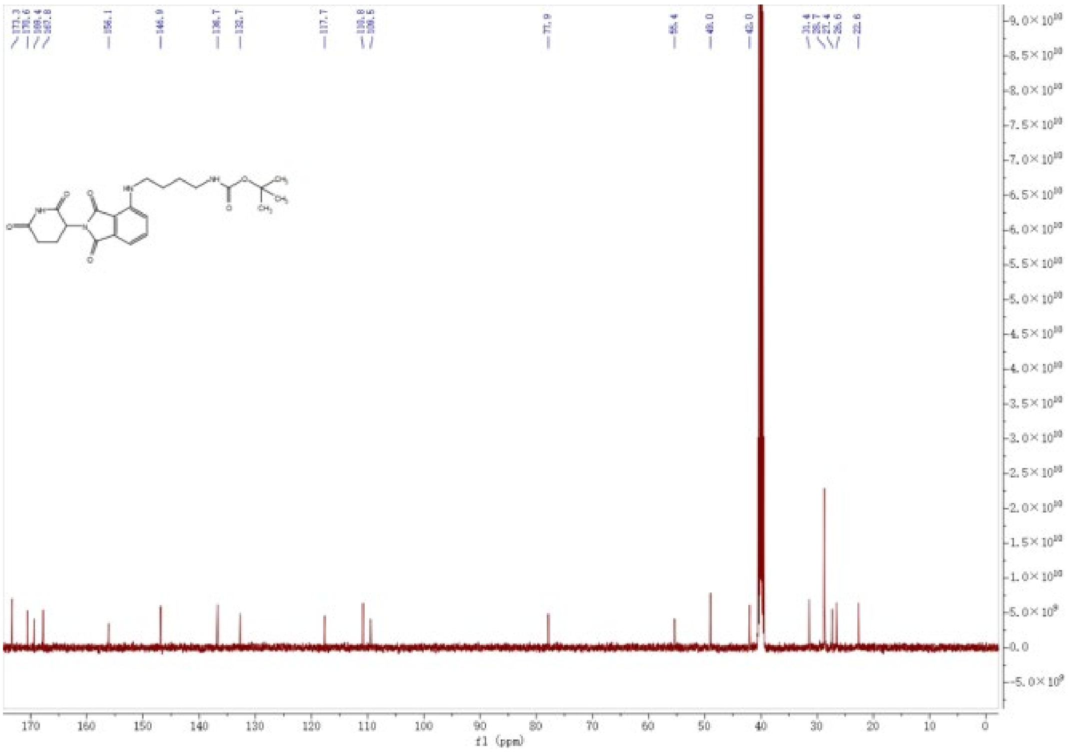

Figure S23 ^{13}C NMR spectrum of 2b

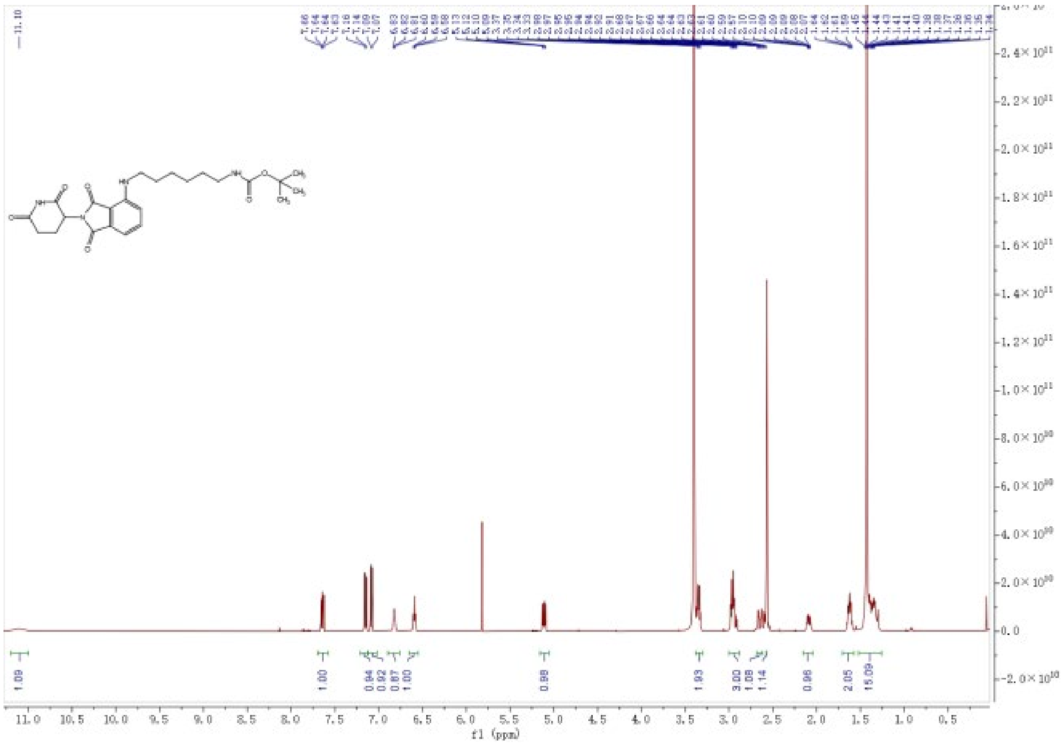

Figure S24 ^{1}H-NMR spectrum of 2c

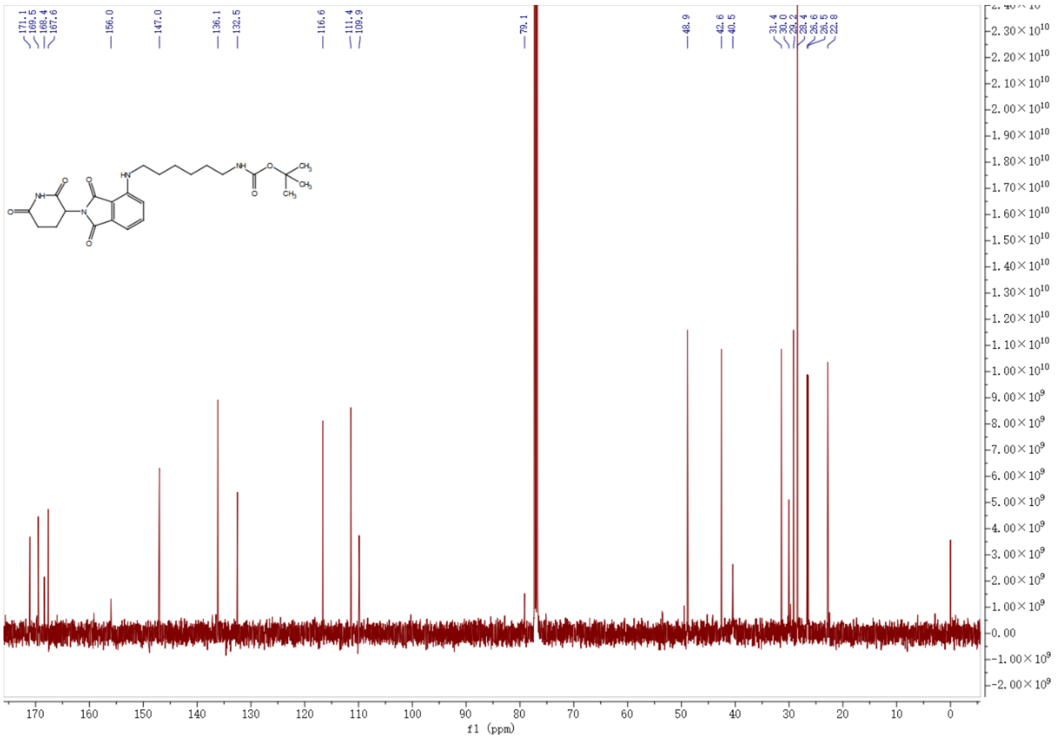

Figure S25 ^{13}C NMR spectrum of 2c

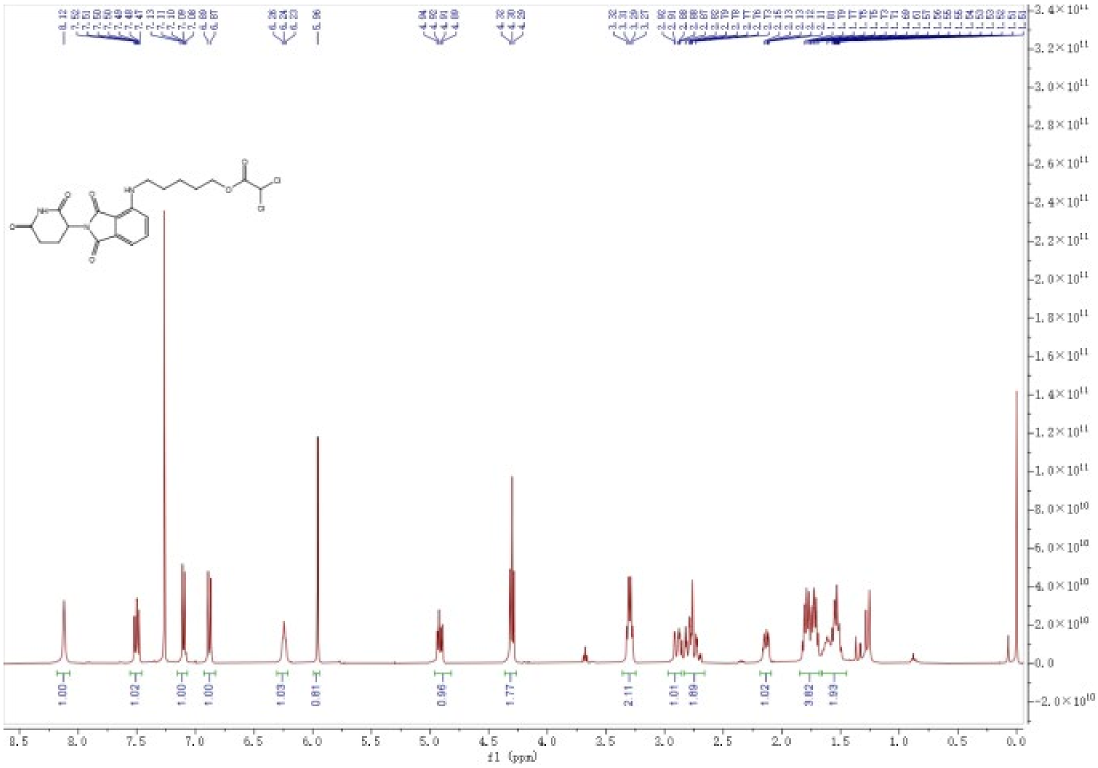

Figure S26 ^{1}H-NMR spectrum of 2d

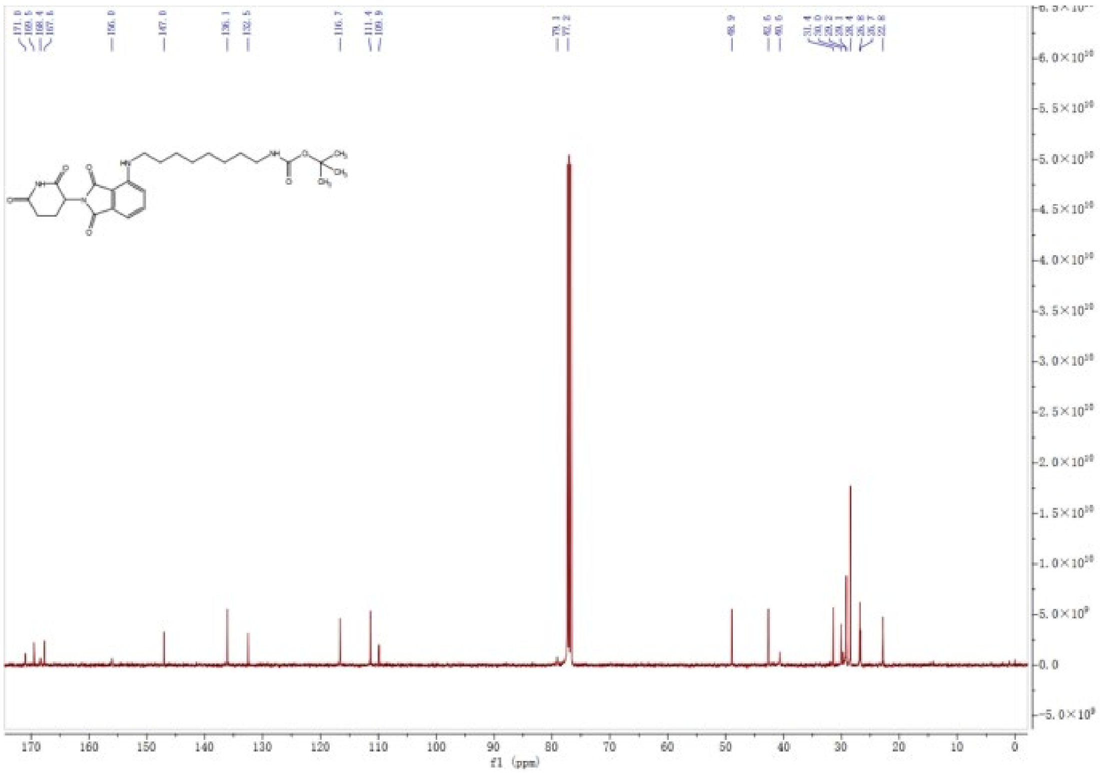

Figure S27 ^{13}C NMR spectrum of 2d

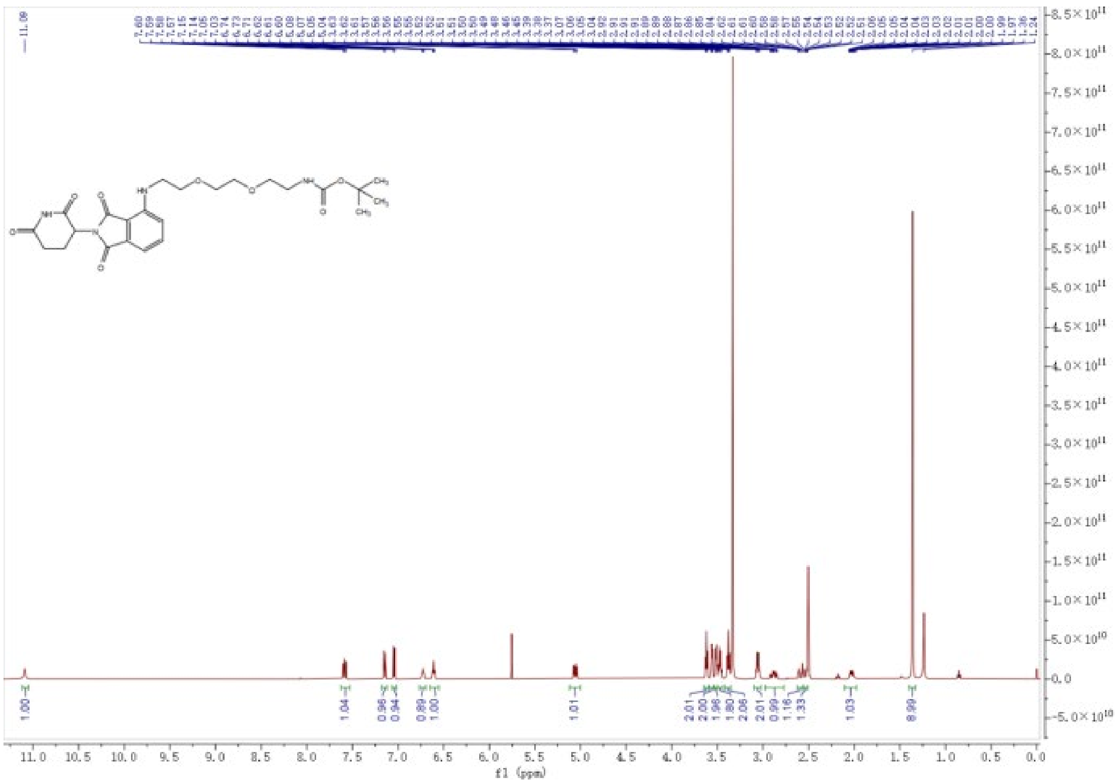

Figure S28 ^{1}H-NMR spectrum of 2e

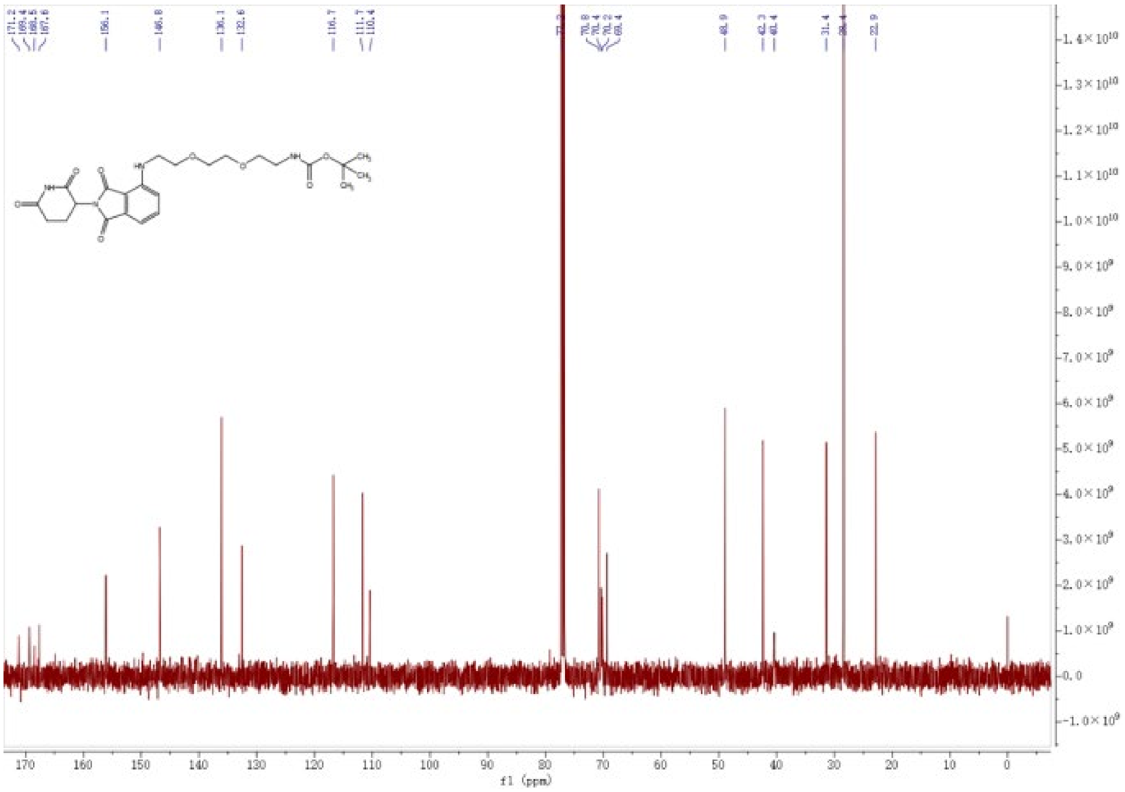

Figure S29 ^{13}C NMR spectrum of 2e

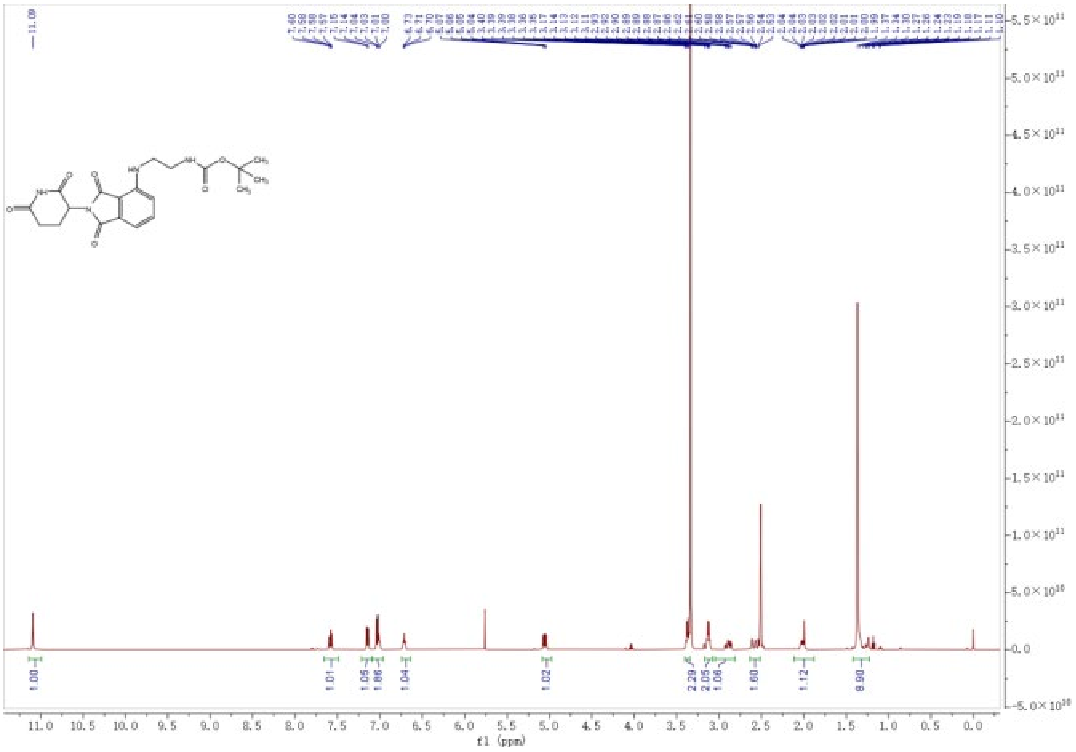

Figure S30 ^{1}H-NMR spectrum of 2f

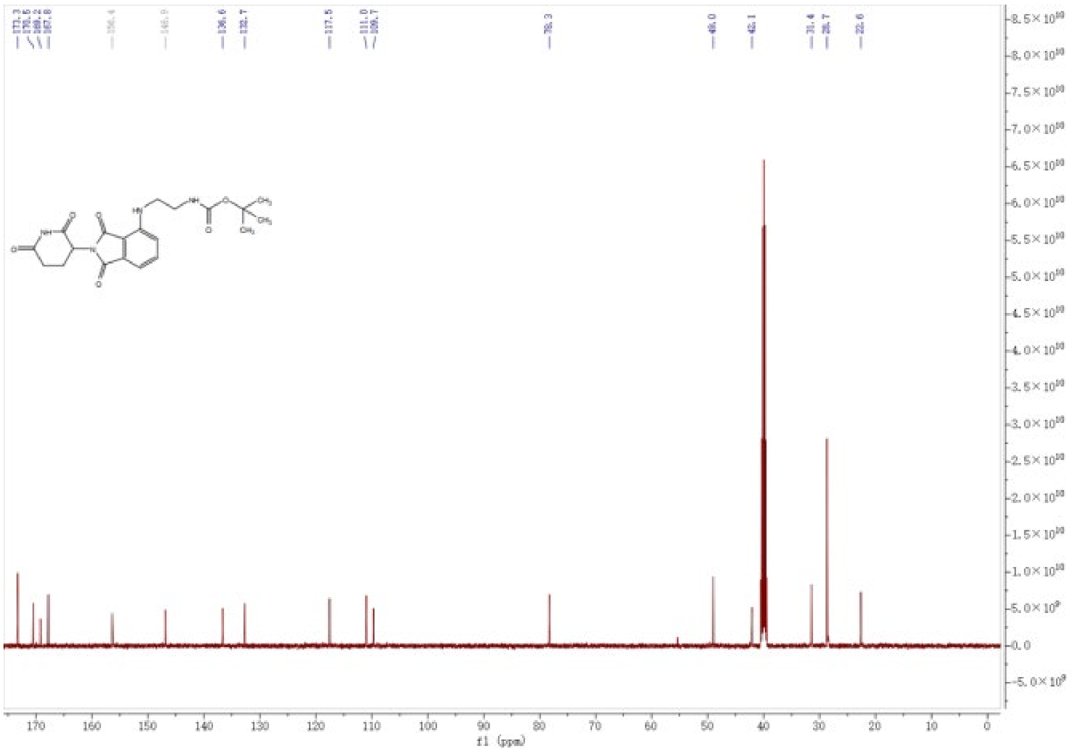

Figure S31 ^{13}C NMR spectrum of 2f

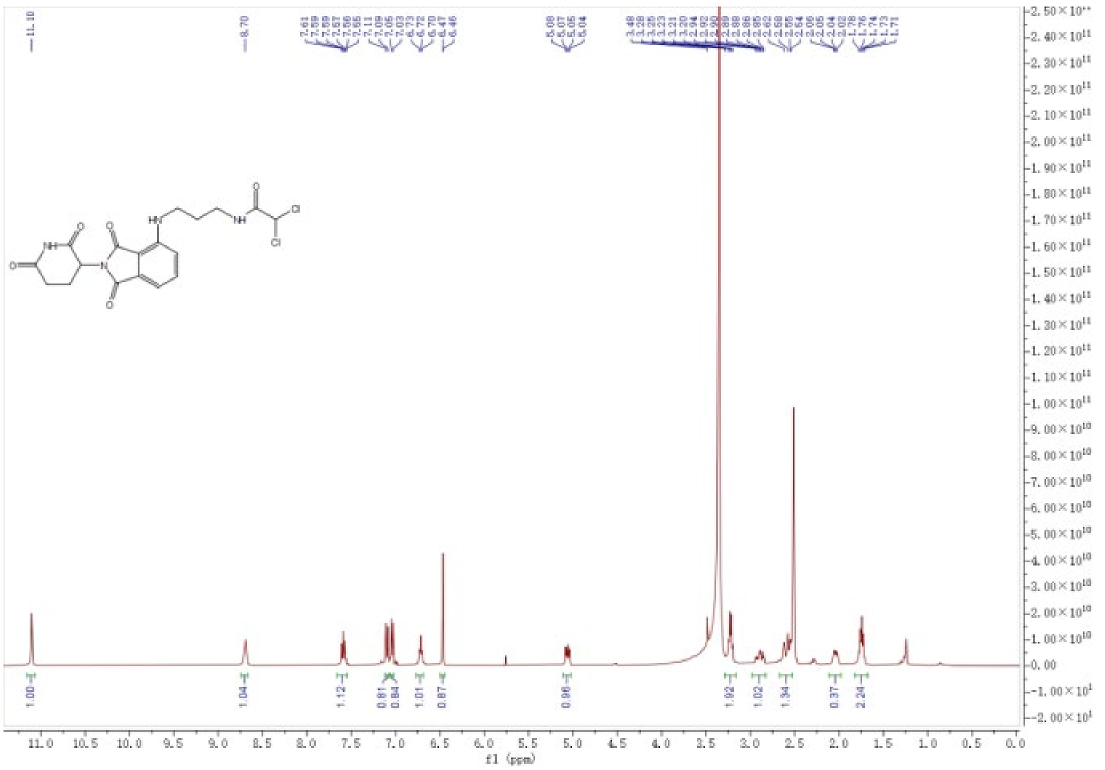

Figure S32 ^{1}H-NMR spectrum of A01

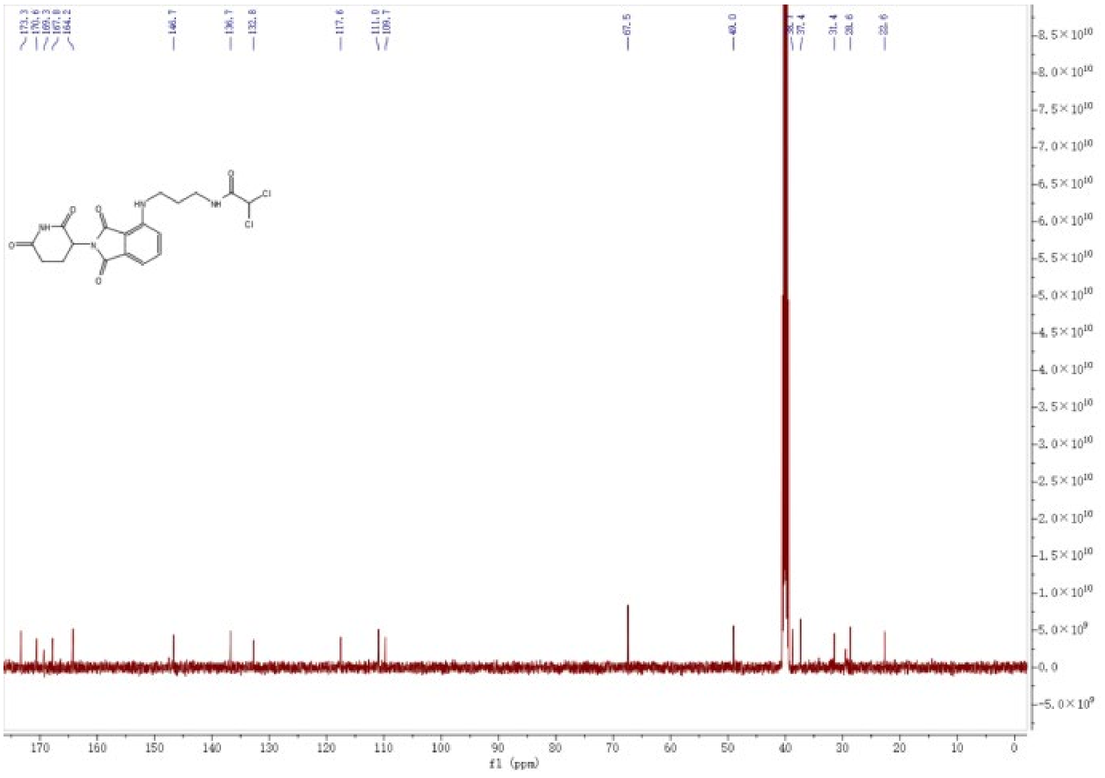

Figure S33 ^{13}C NMR spectrum of A01

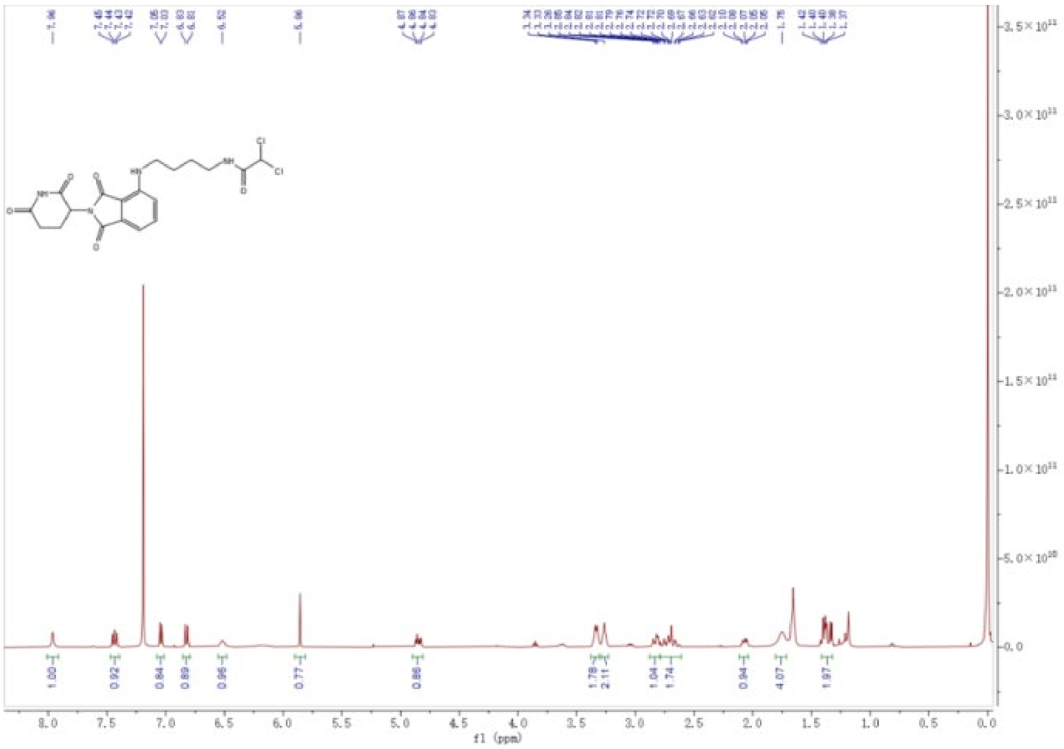

Figure S34 ^{1}H-NMR spectrum of A02

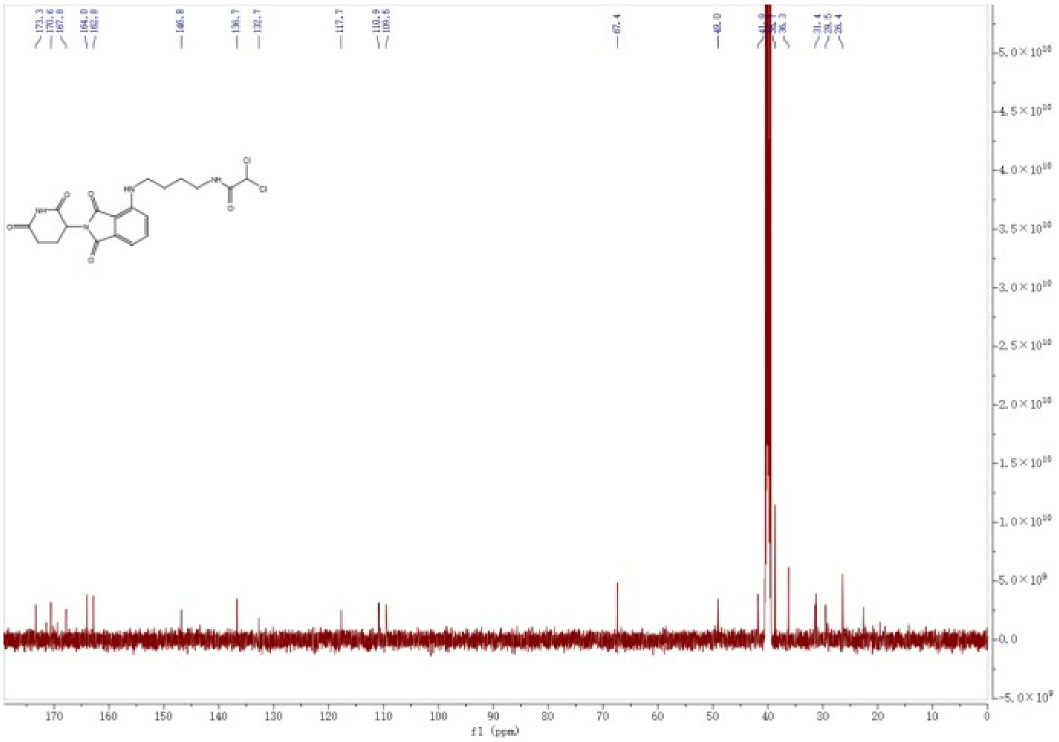

Figure S35 ^{13}C NMR spectrum of A02

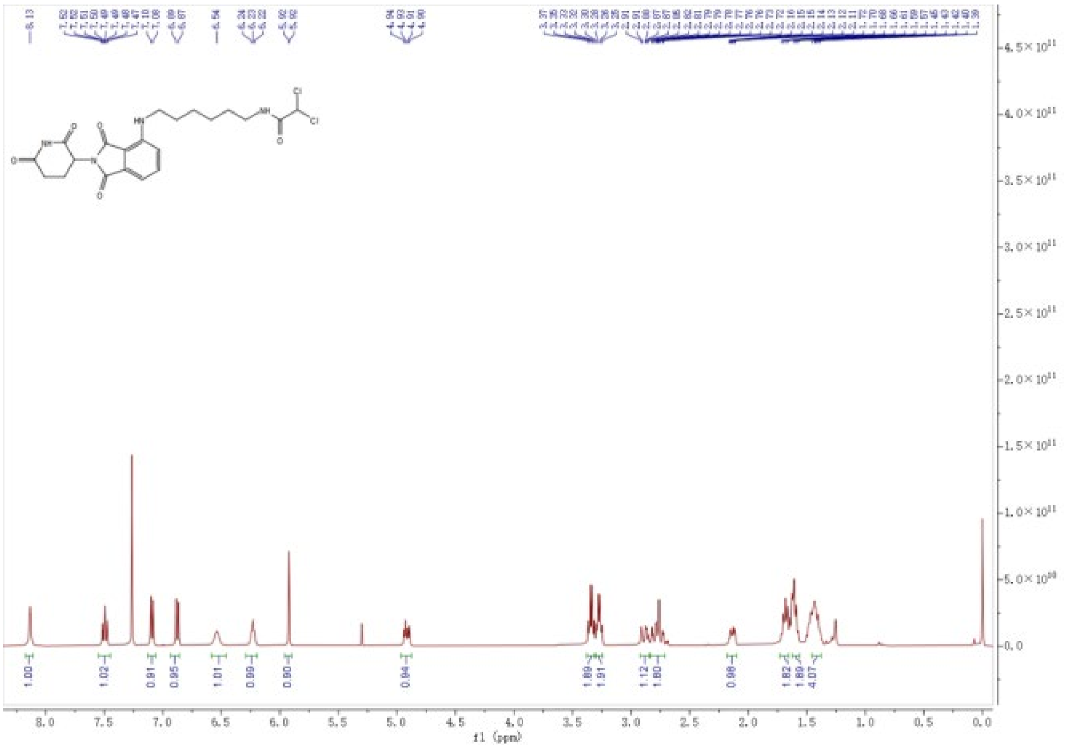

Figure S36 ^{1}H-NMR spectrum of A03

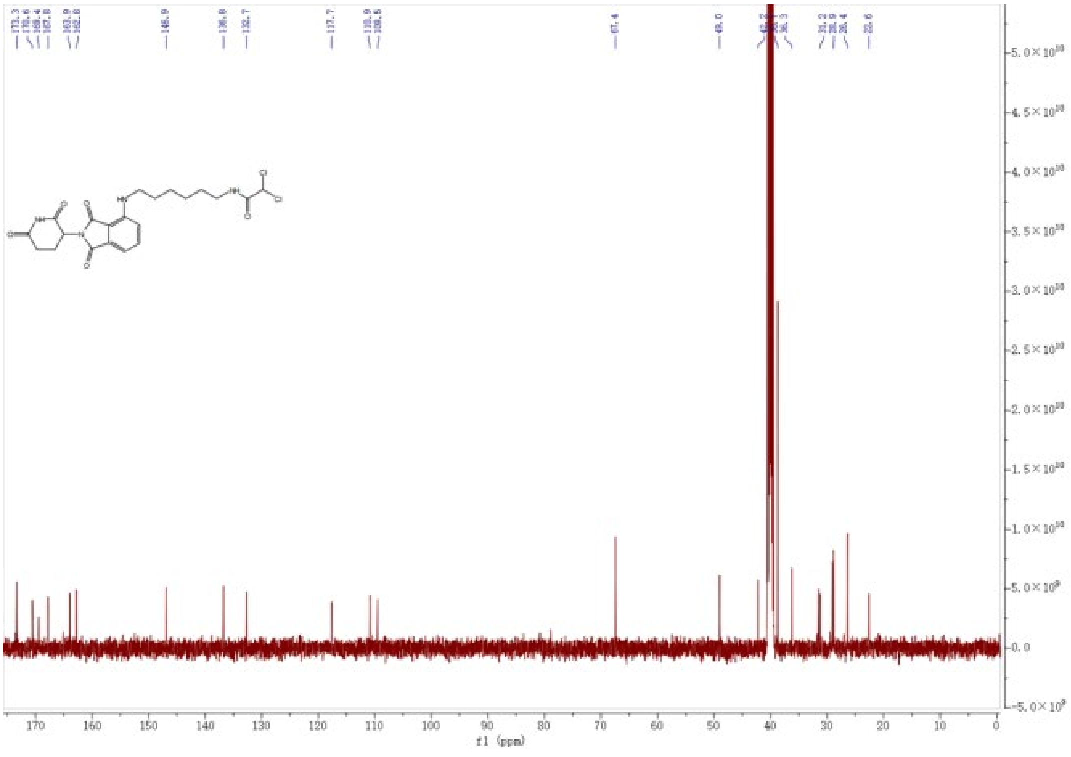

Figure S37 ^{13}C-NMR spectrum of A03

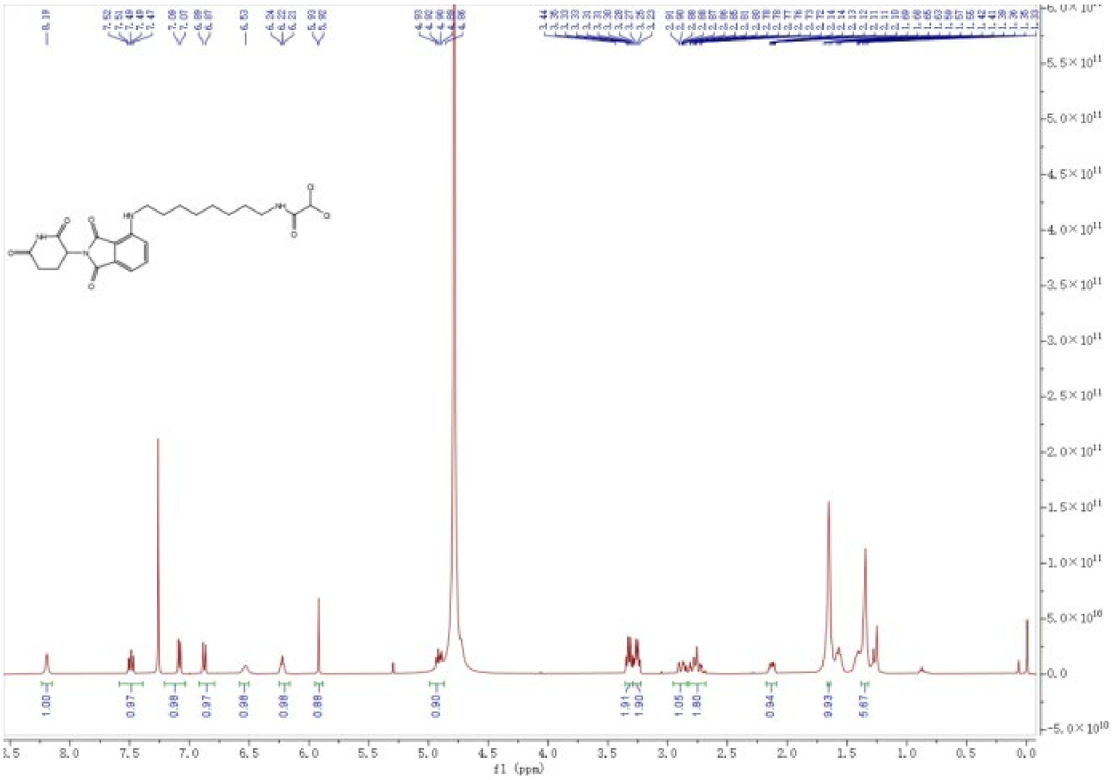

Figure S38 ^{1}H-NMR spectrum of **A04**

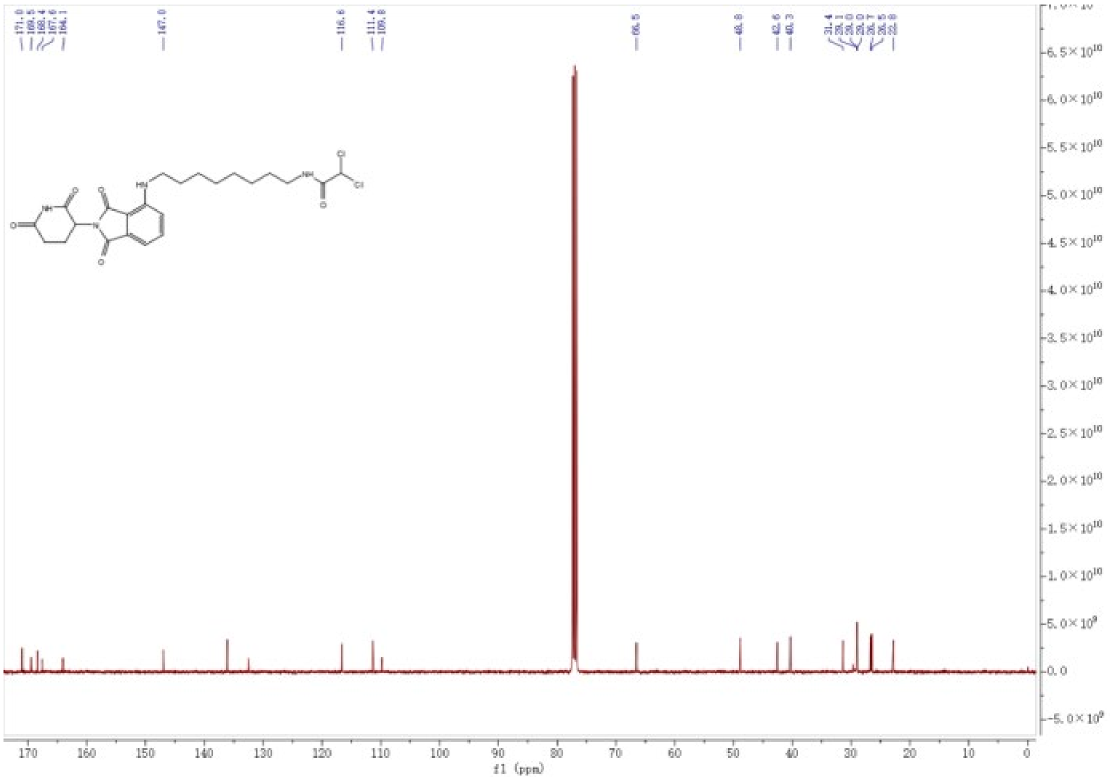

Figure S39 ^{13}C-NMR spectrum of **A04**

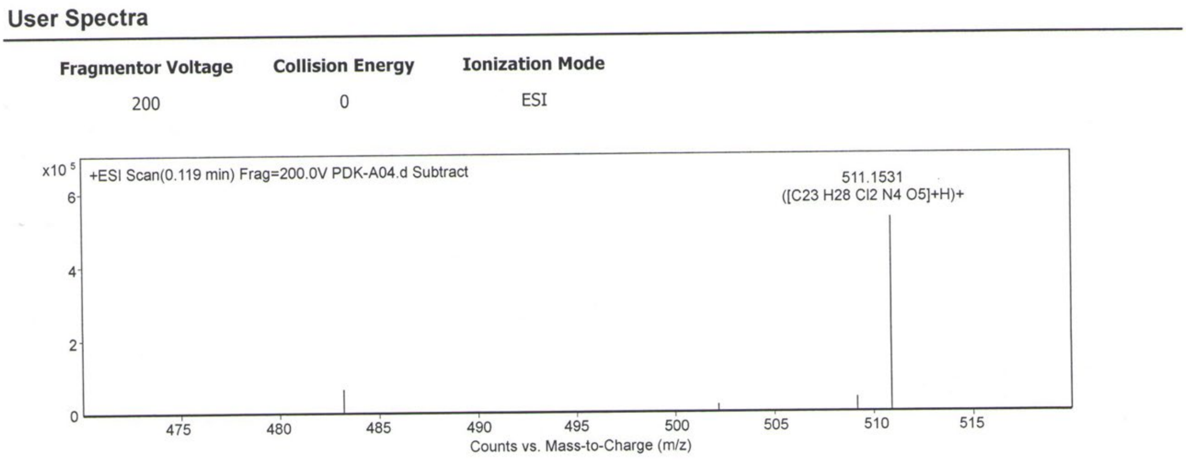

Figure S40 HR-MS spectrum of **A04**

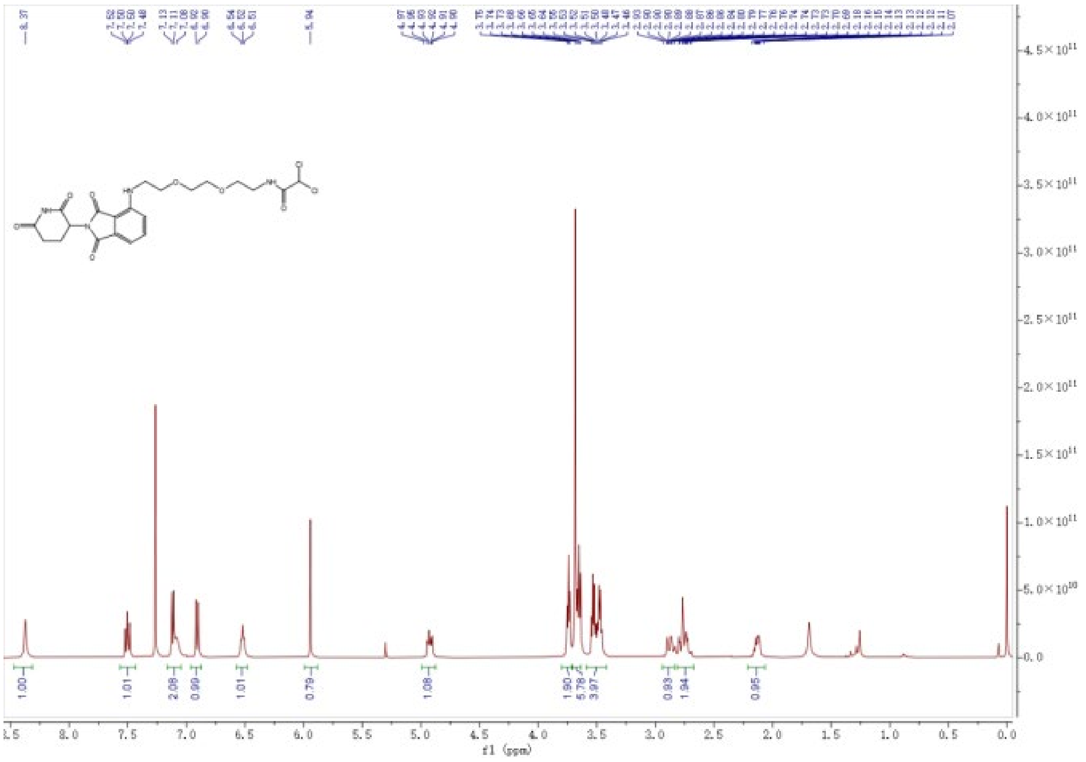

Figure S41 ^{1}H-NMR spectrum of A05

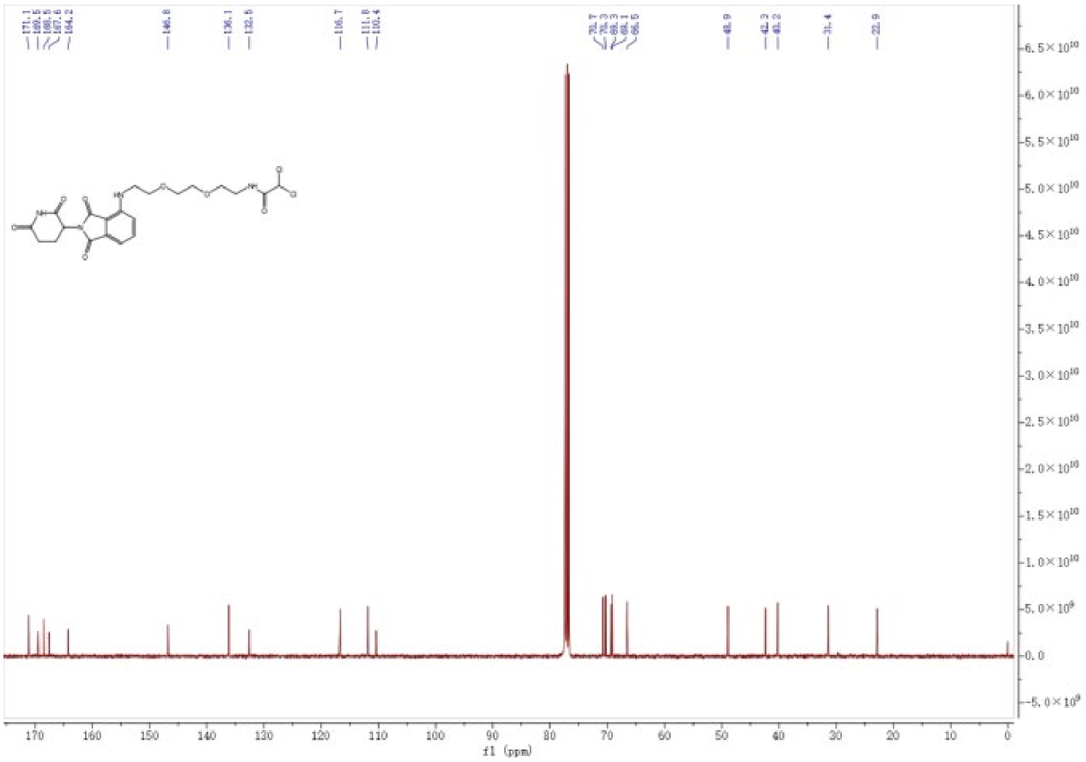

Figure S42 ^{13}C-NMR spectrum of A05

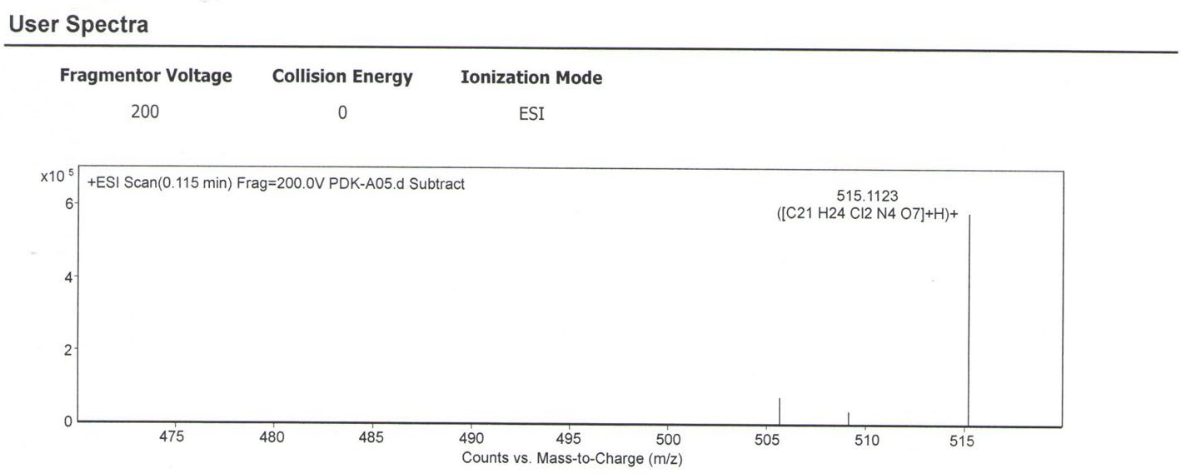

Figure S43 HR-MS spectrum of A05

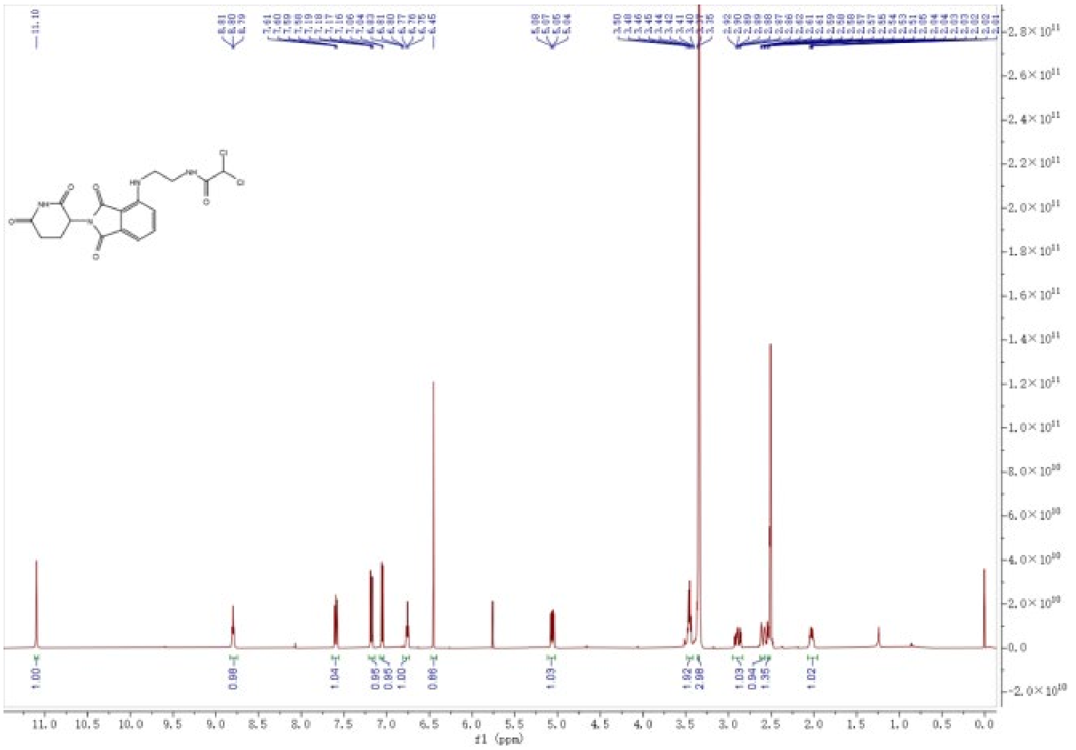

Figure S44 ^{1}H-NMR spectrum of A06

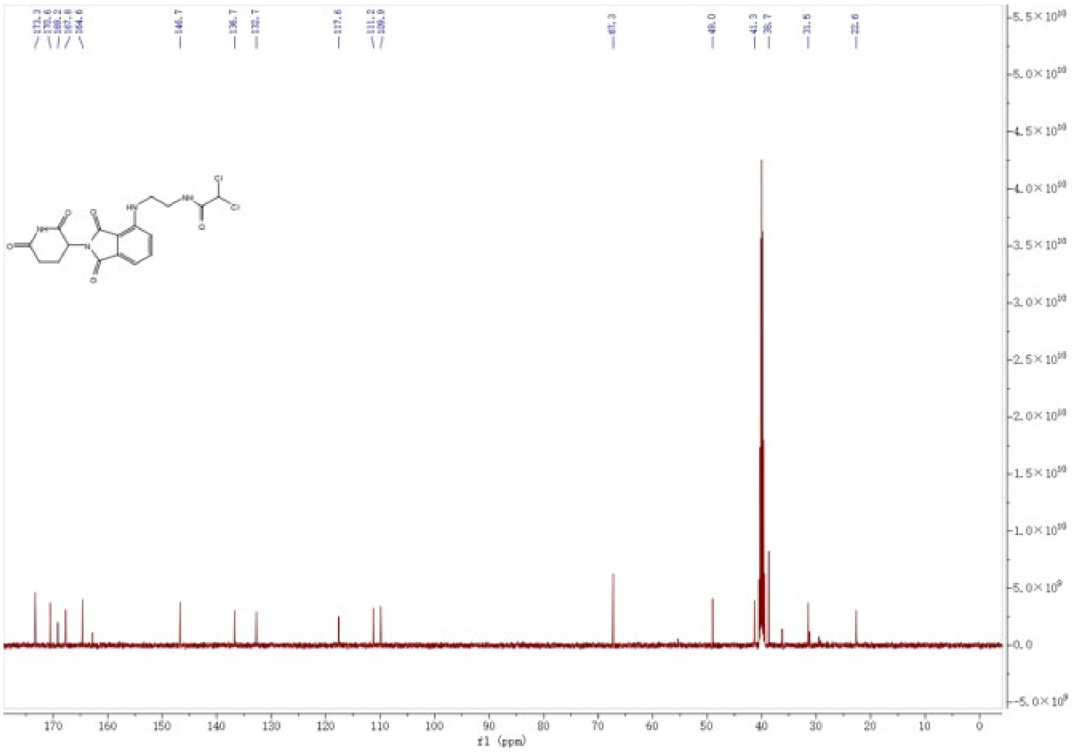

Figure S45 ^{13}C-NMR spectrum of A06

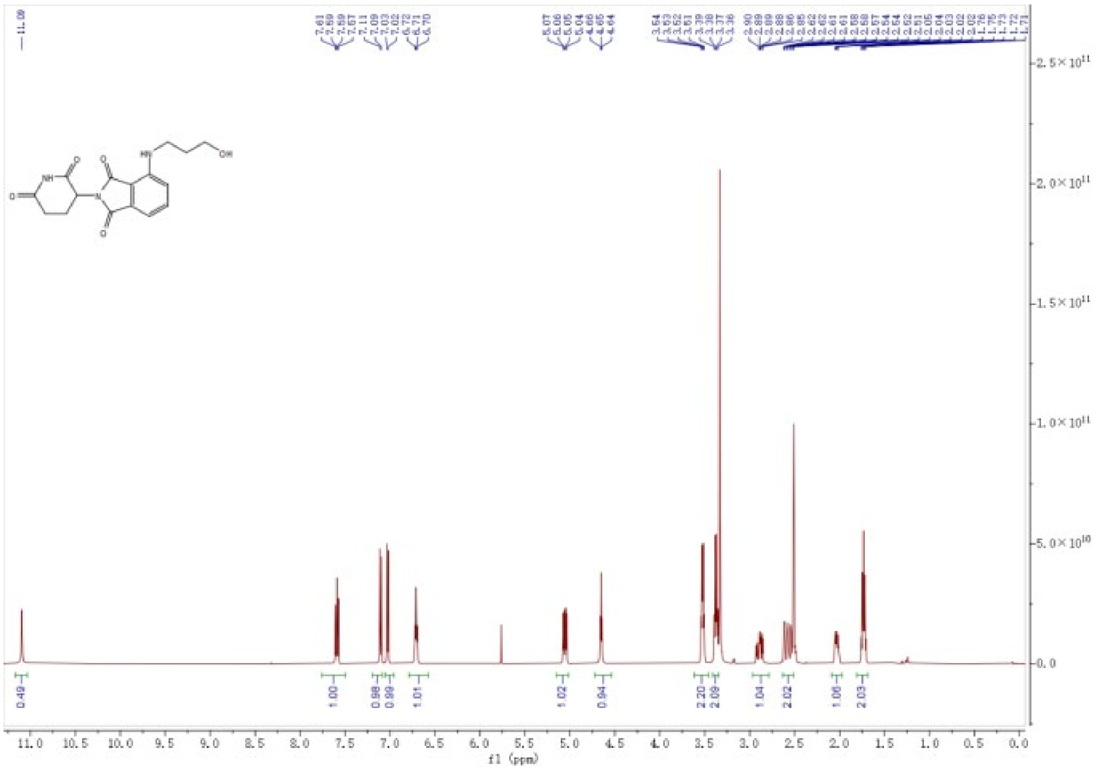

Figure S46 ^{1}H-NMR spectrum of 4a

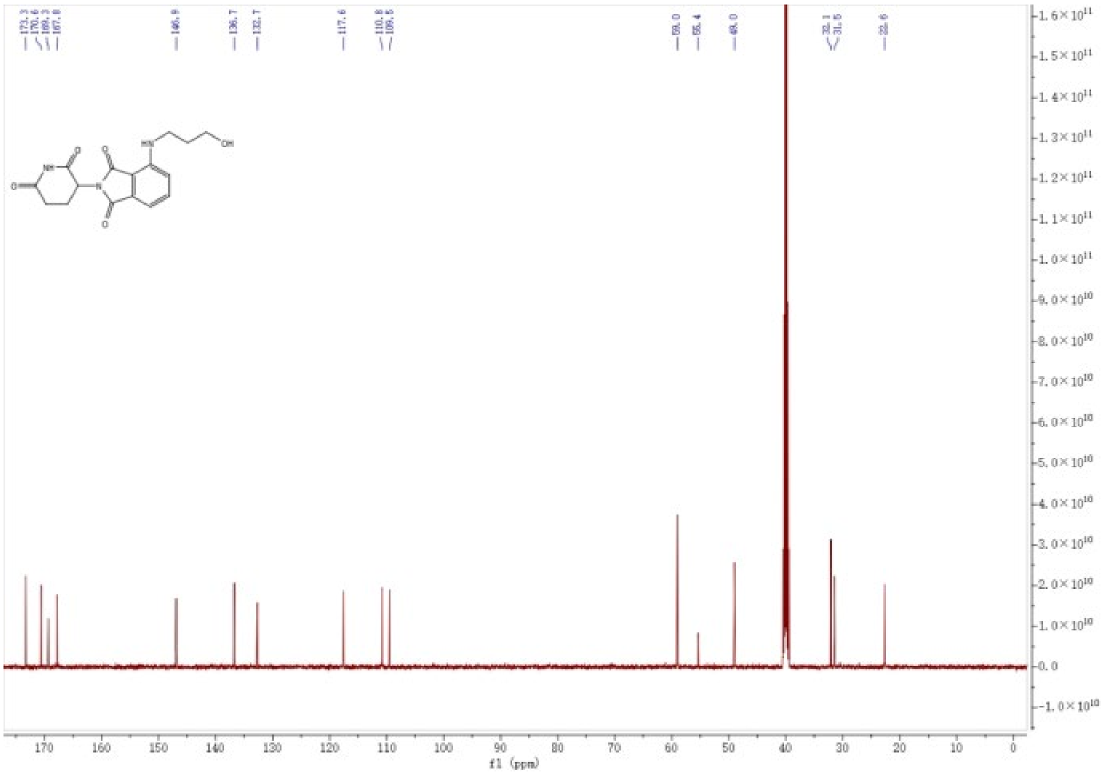

Figure S47 ^{13}C-NMR spectrum of 4a

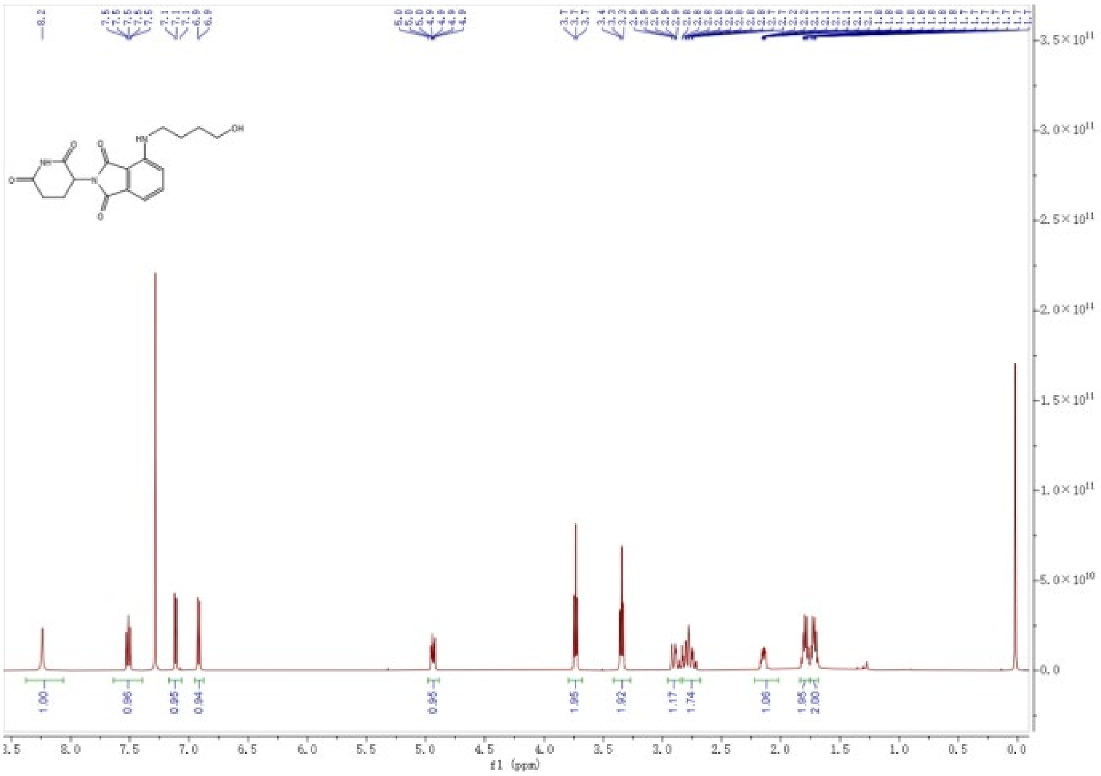

Figure S48 ^{1}H-NMR spectrum of 4b

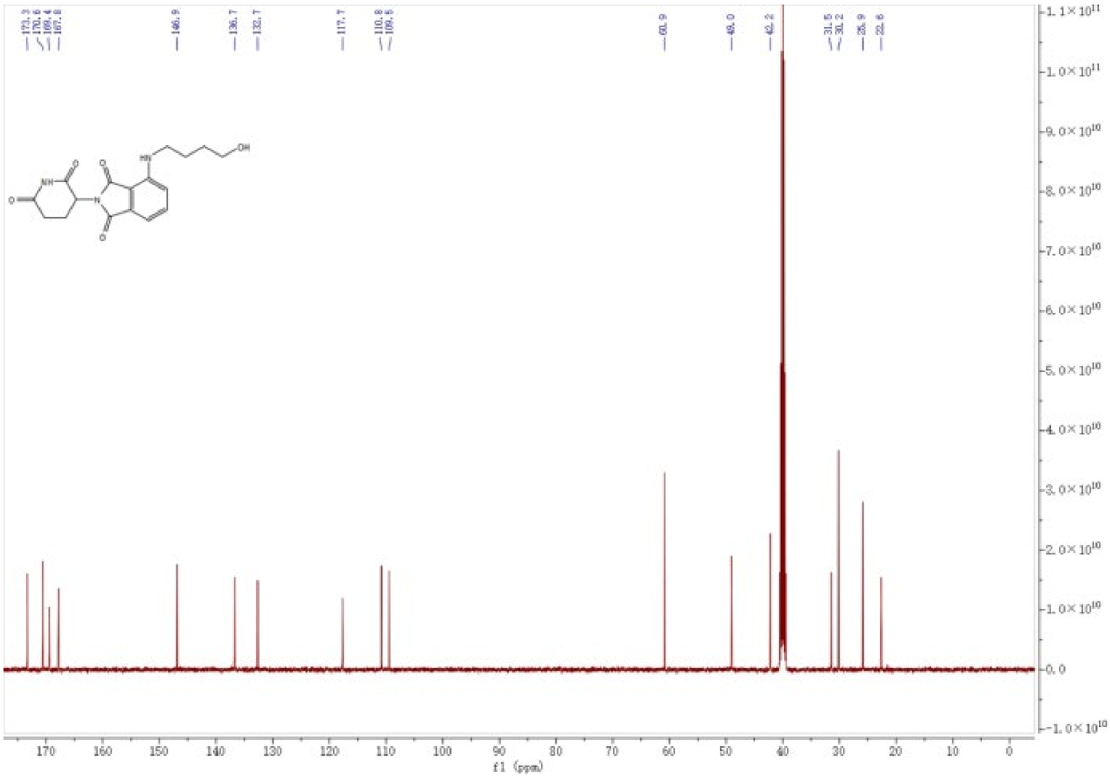

Figure S49 ^{13}C-NMR spectrum of 4b

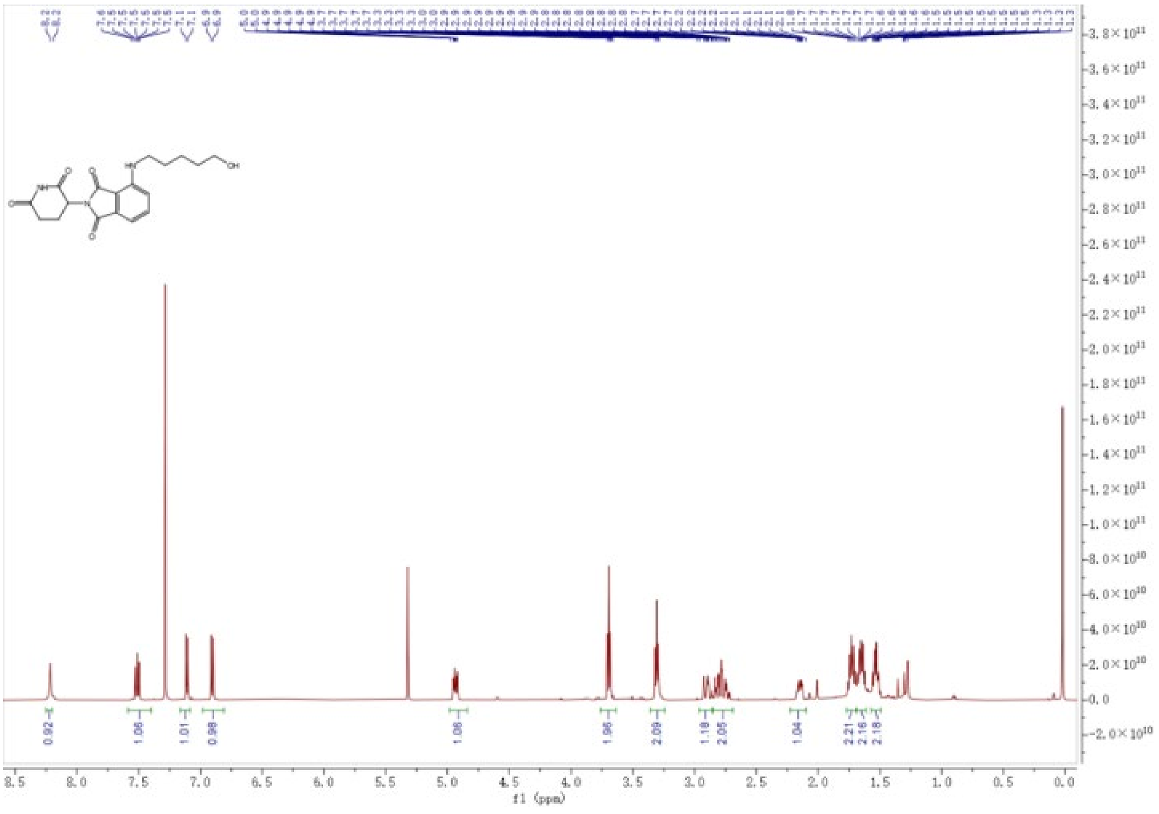

Figure S50 ^{1}H-NMR spectrum of 4c

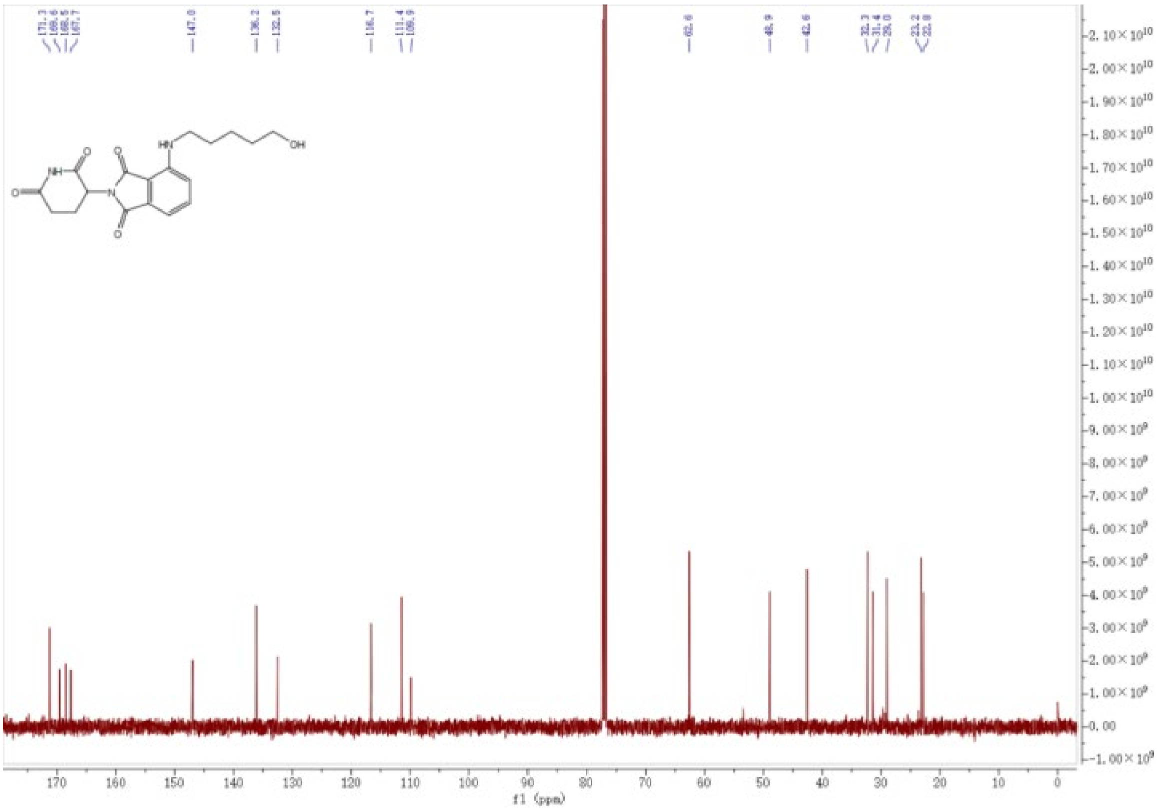

Figure S51 ^{13}C-NMR spectrum of 4c

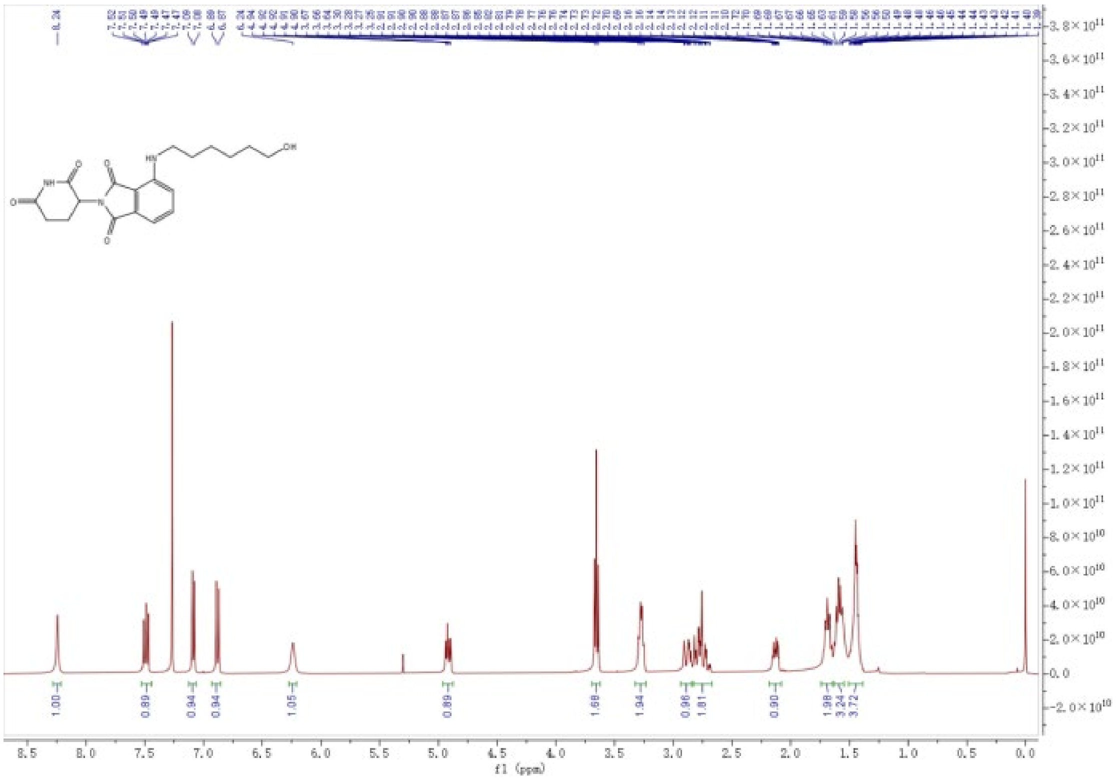

Figure S52 ^{1}H-NMR spectrum of 4d

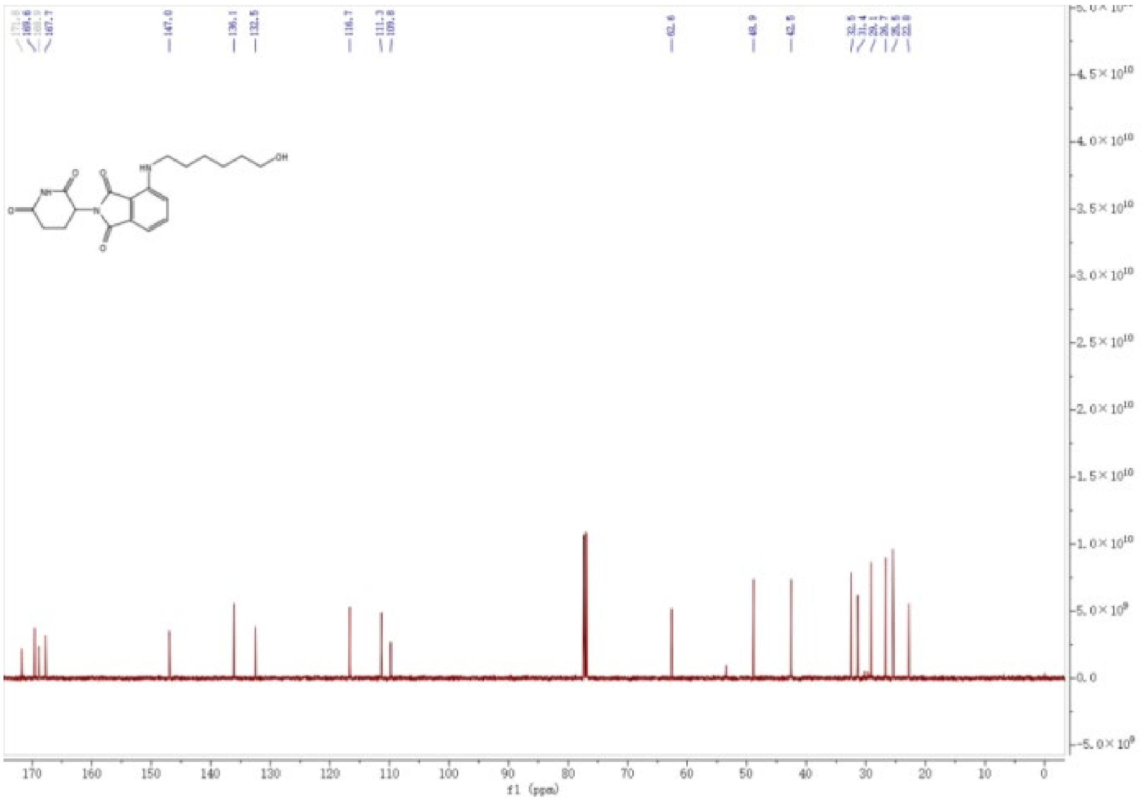

Figure S53 ^{13}C-NMR spectrum of 4d

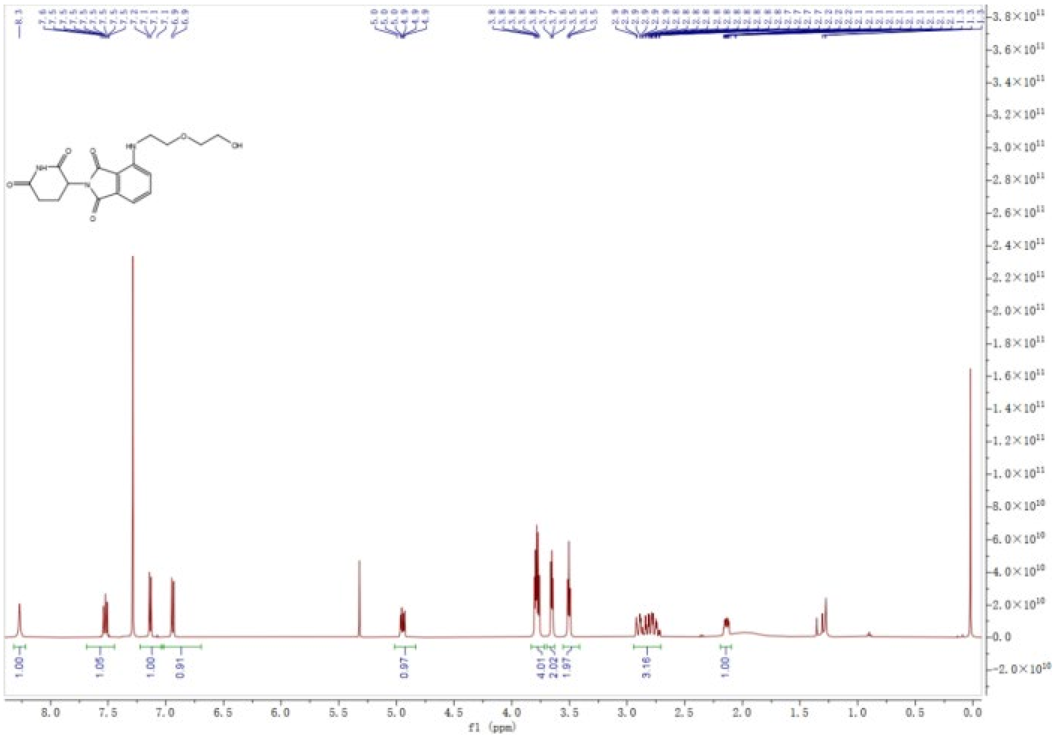

Figure S54 ^{1}H-NMR spectrum of 4e

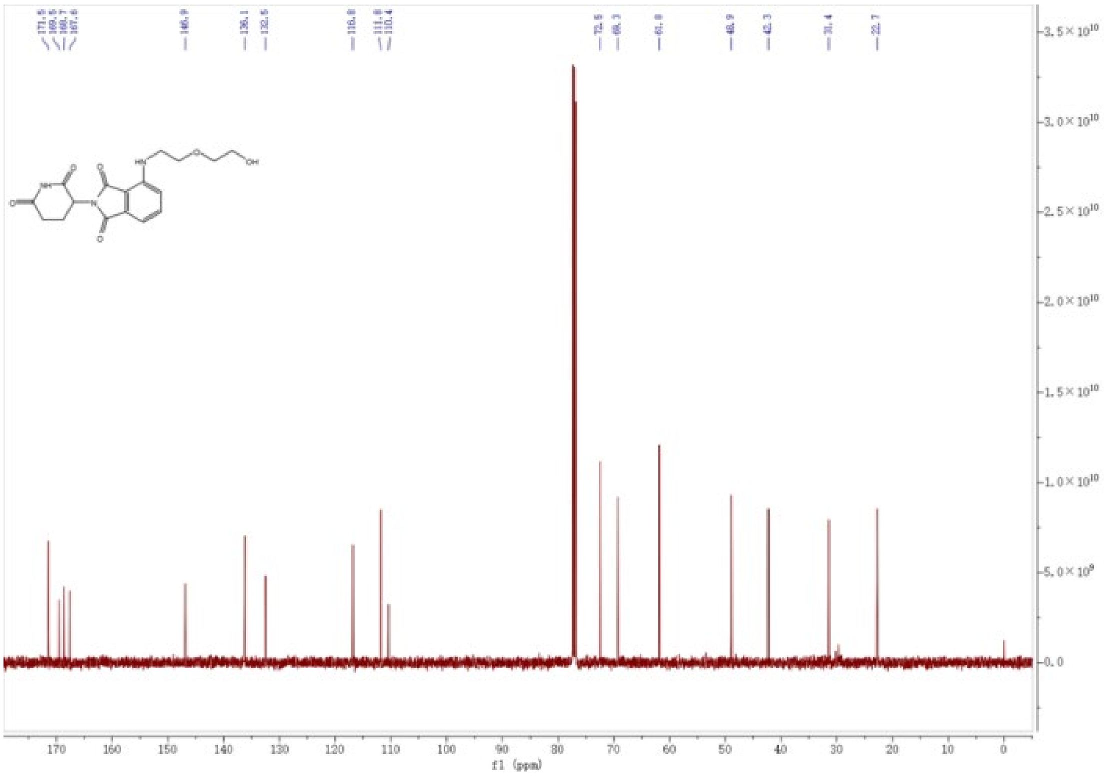

Figure S55 ^{13}C-NMR spectrum of 4e

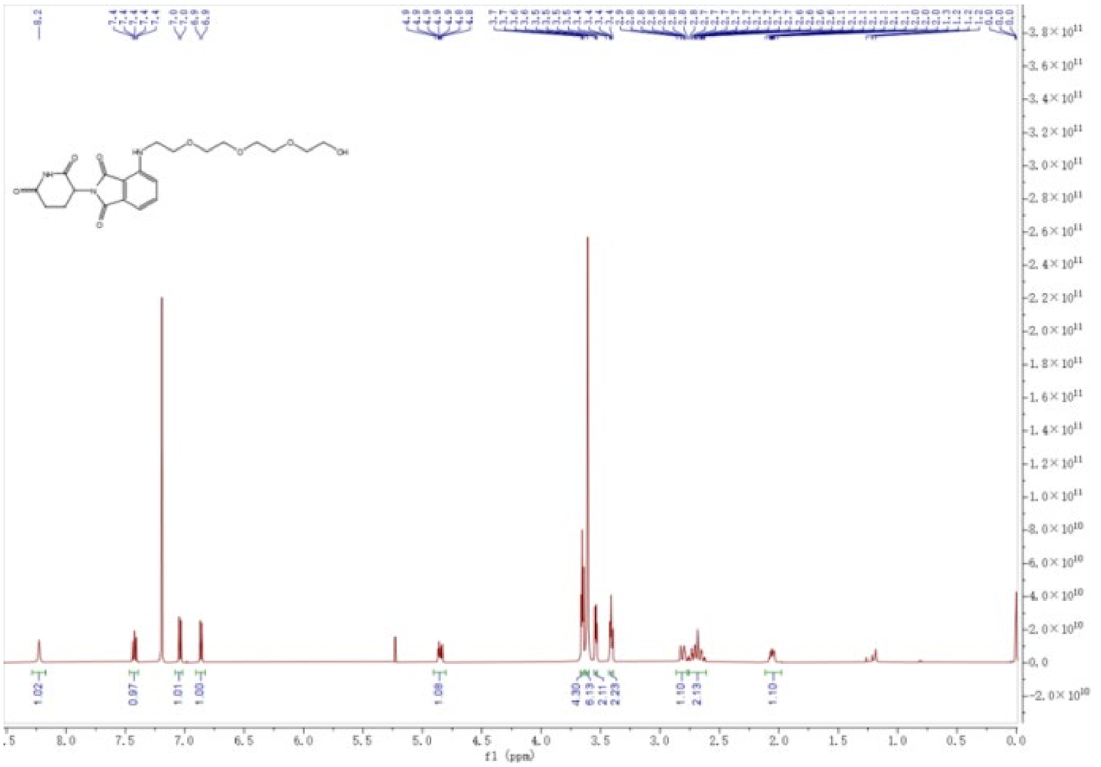

Figure S56 ^{1}H-NMR spectrum of 4f

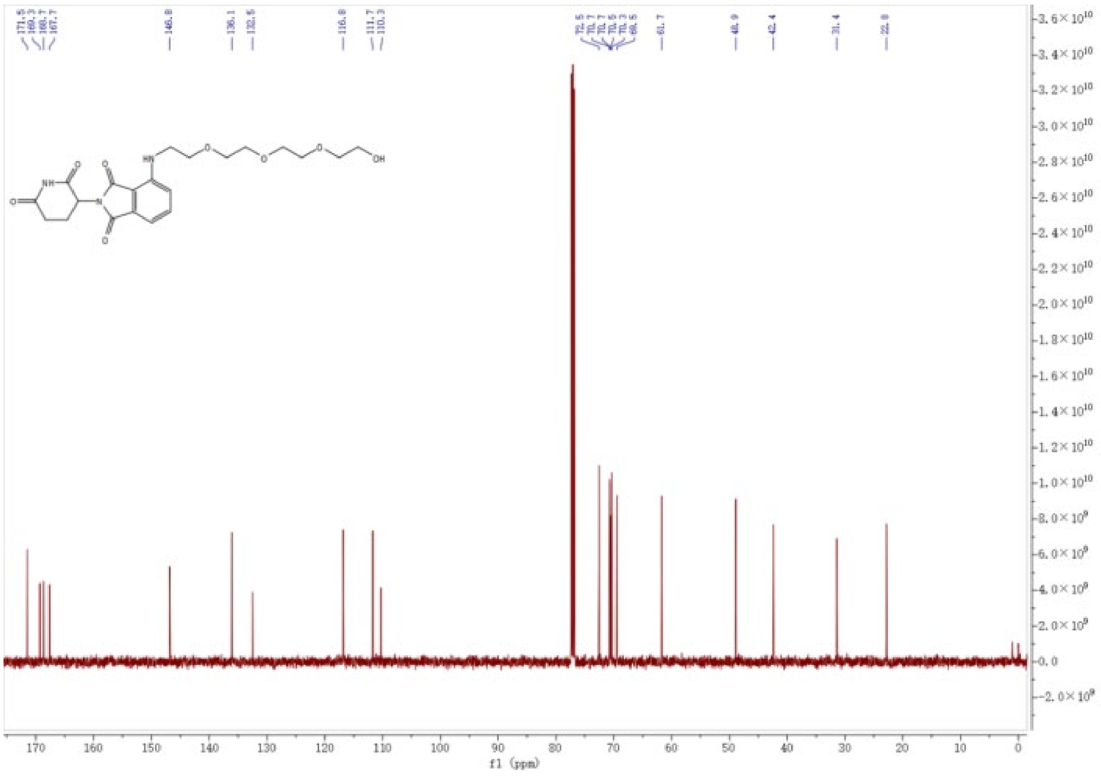

Figure S57 ^{13}C-NMR spectrum of 4f

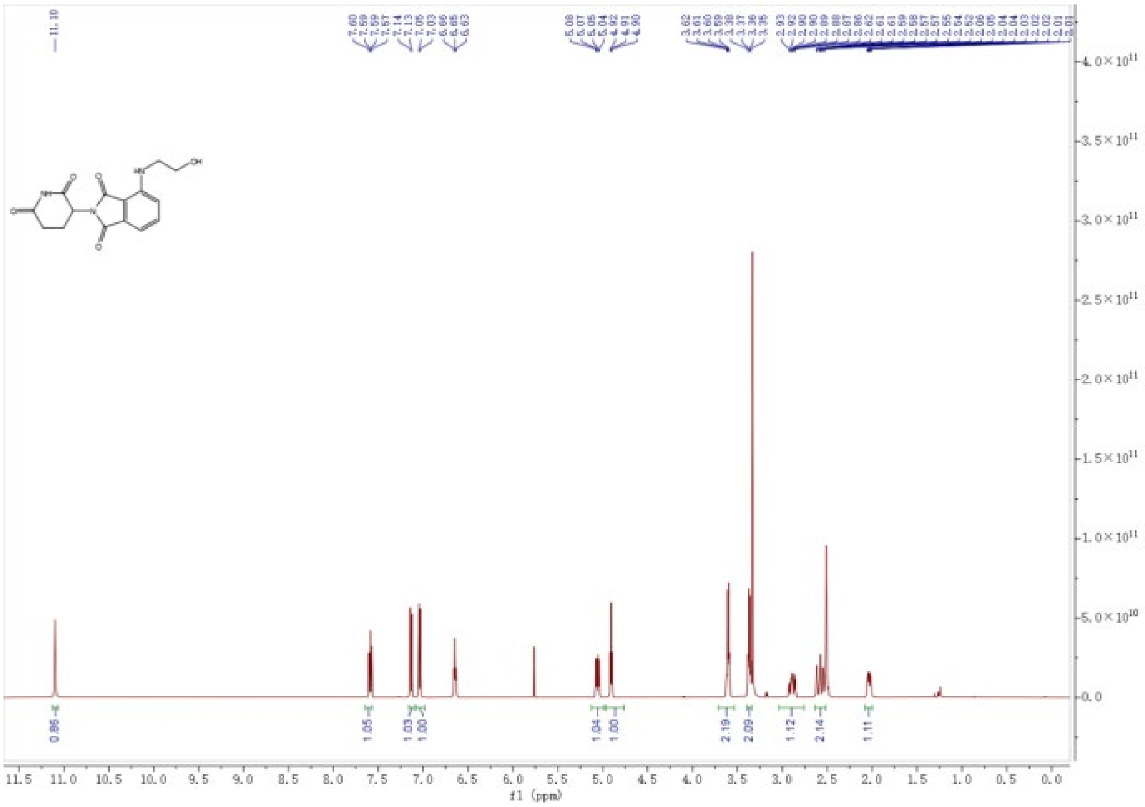

Figure S58 ^{1}H-NMR spectrum of 4g

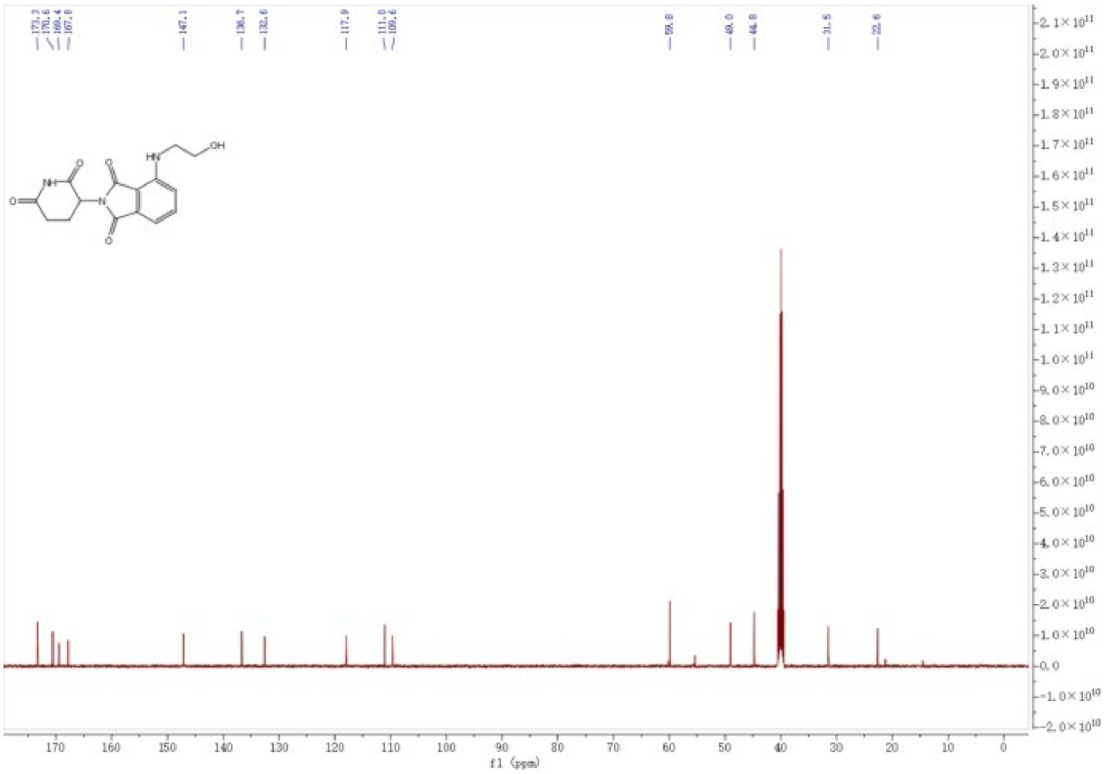

Figure S59 ^{13}C-NMR spectrum of 4g

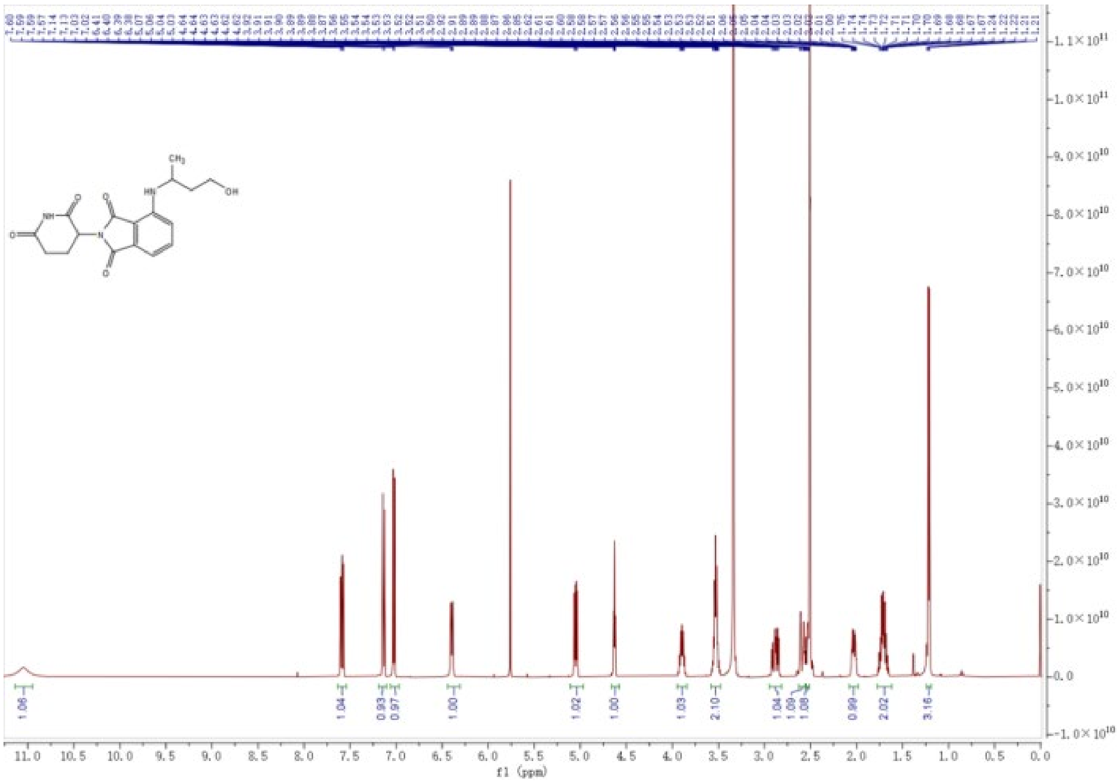

Figure S60 ^{1}H-NMR spectrum of 4h

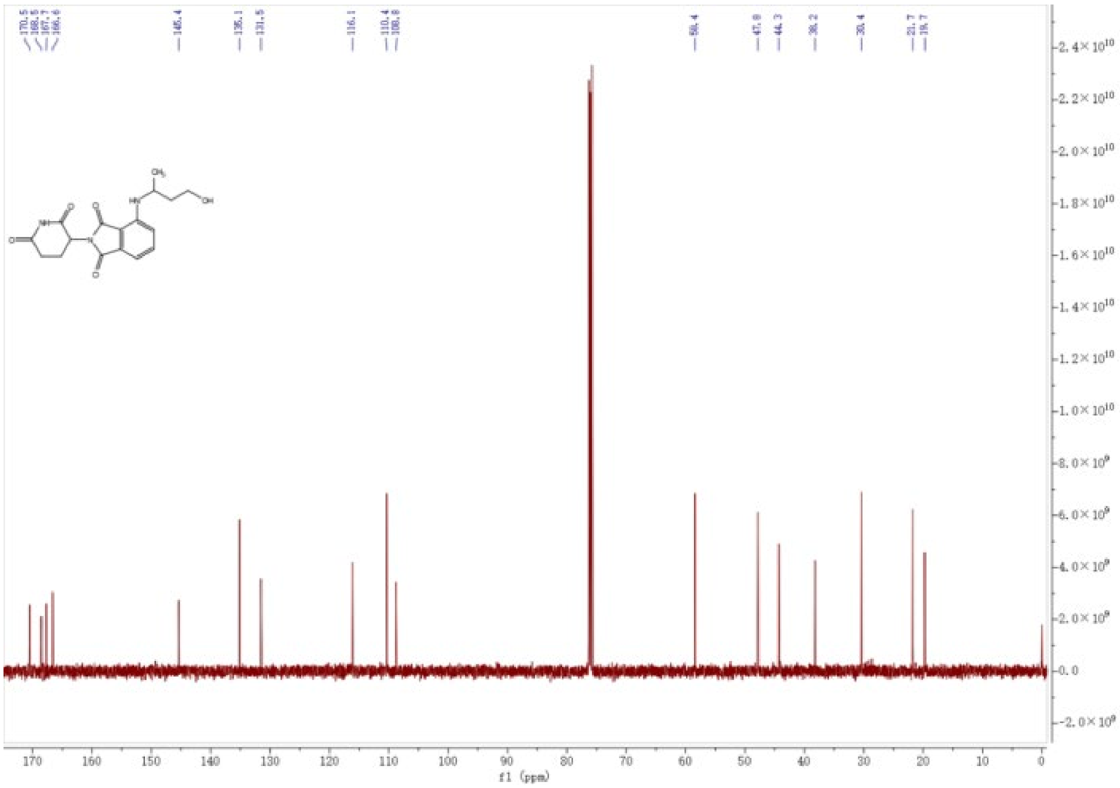

Figure S61 ^{13}C-NMR spectrum of 4h

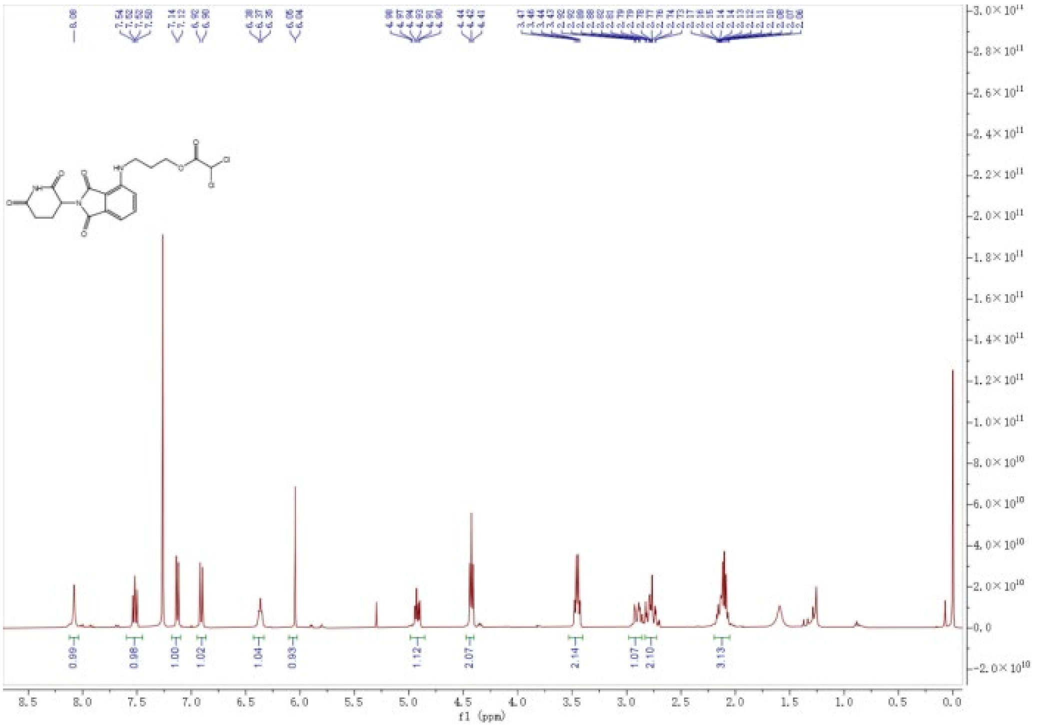

Figure S62 ^{1}H-NMR spectrum of B01

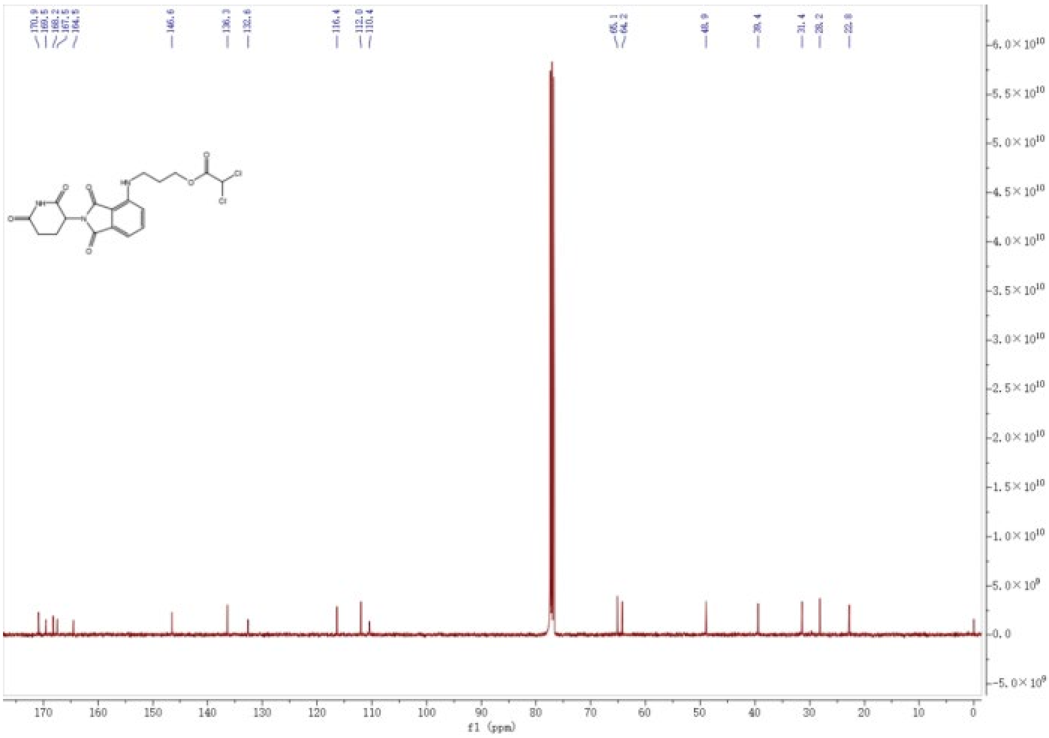

Figure S63 ^{13}C-NMR spectrum of B01

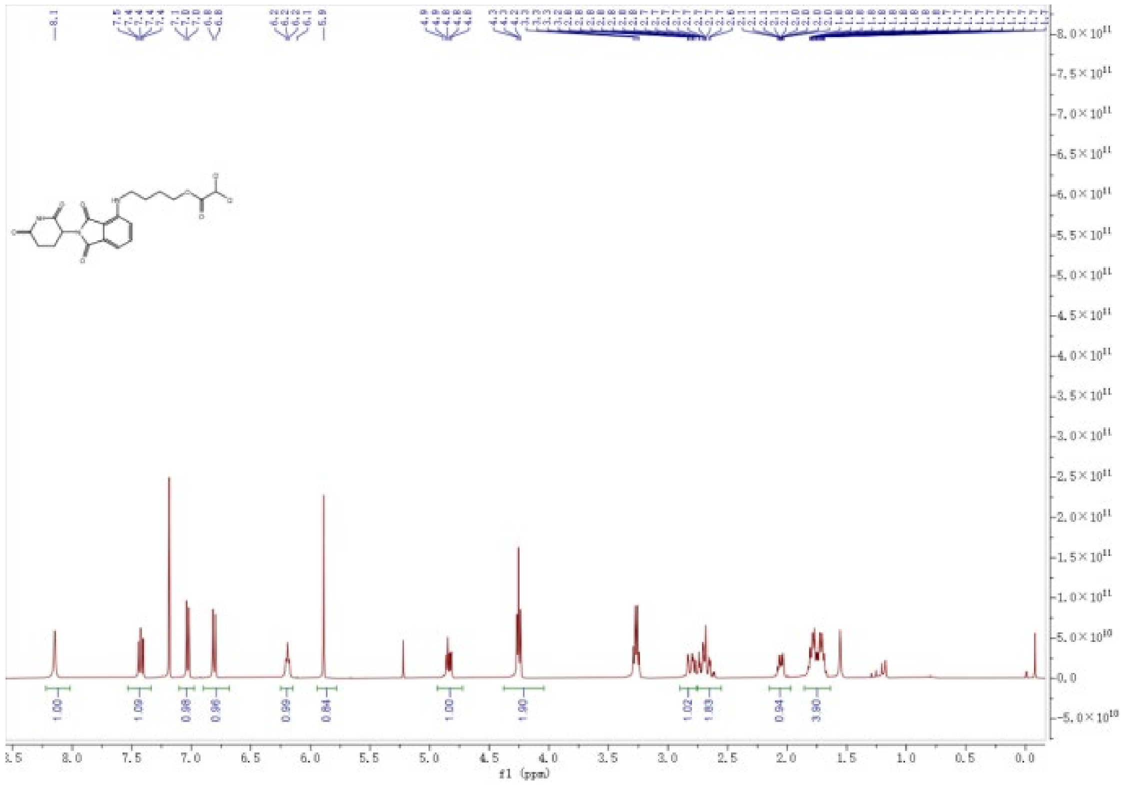

Figure S64 ^{1}H-NMR spectrum of B02

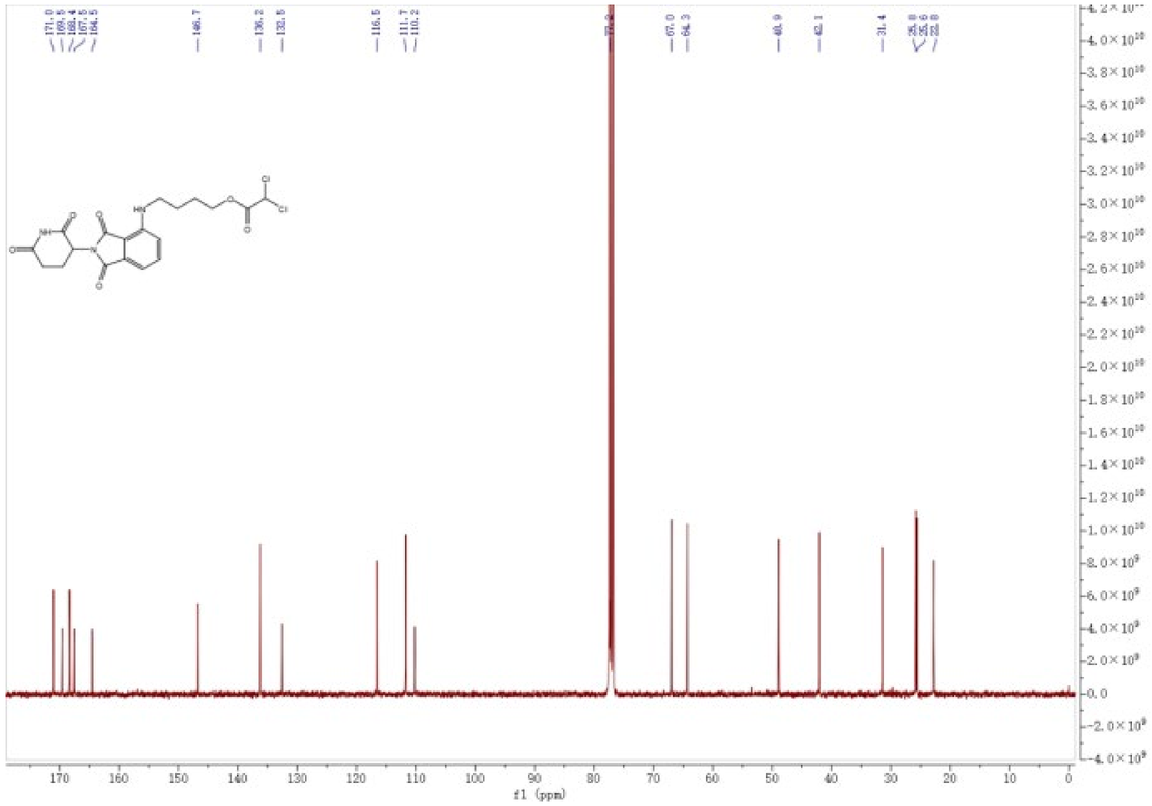

Figure S65 ^{13}C-NMR spectrum of B02

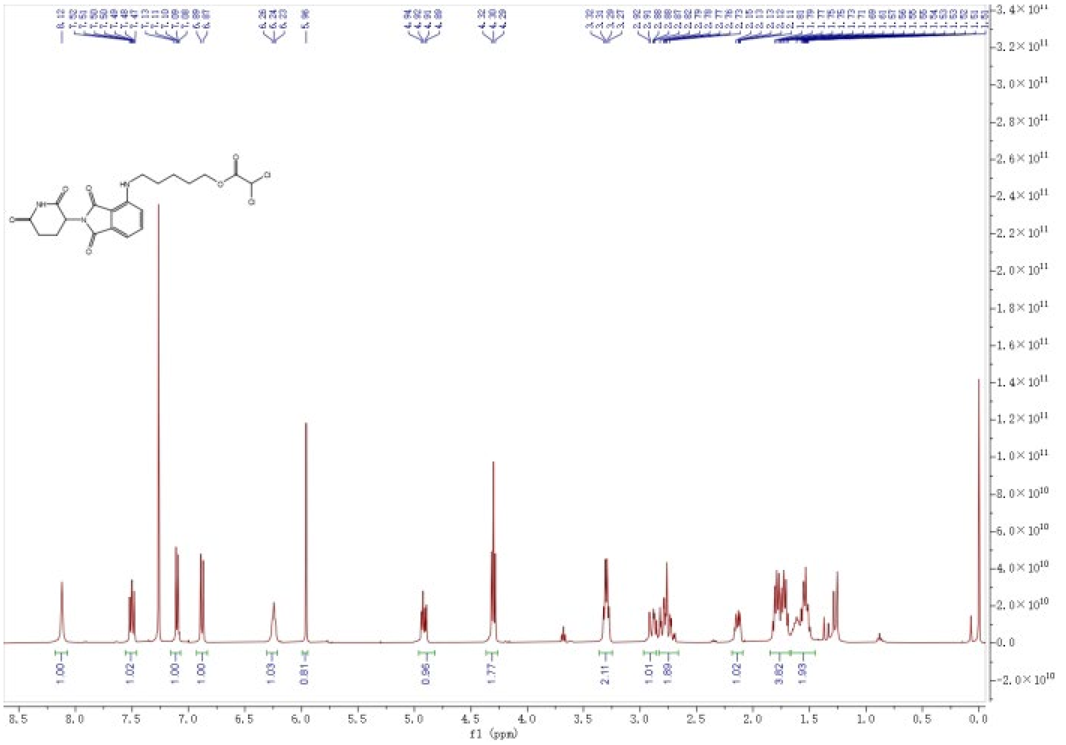

Figure S66 ^{1}H-NMR spectrum of B03

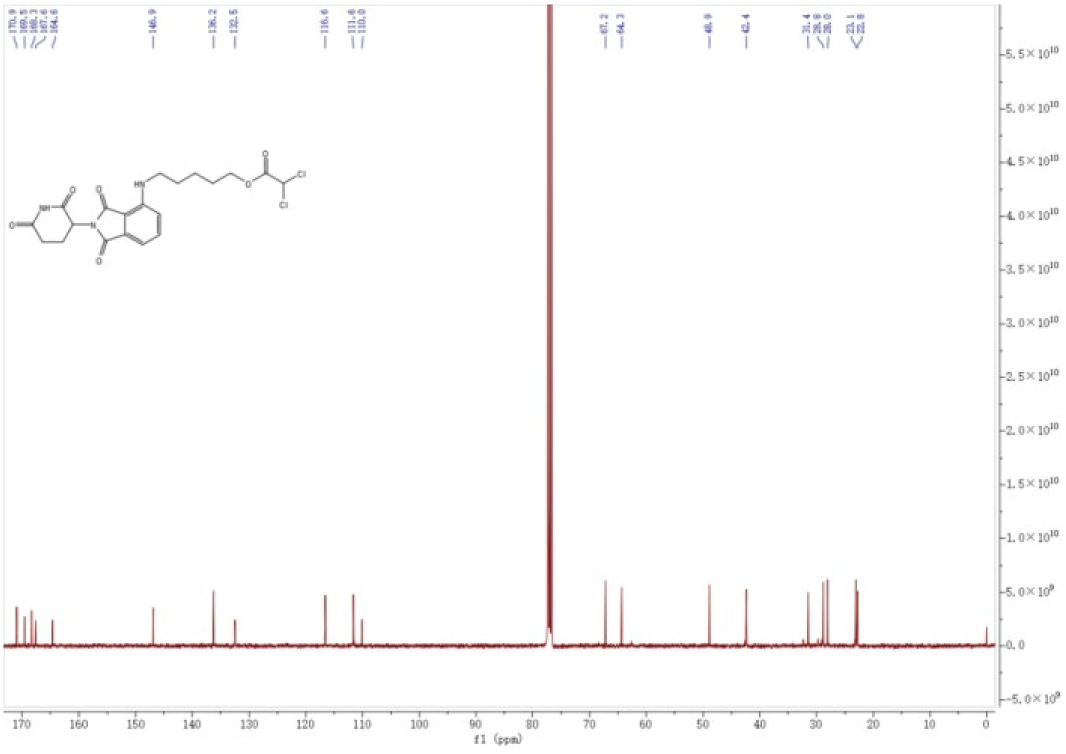

Figure S67 ^{13}C-NMR spectrum of B03

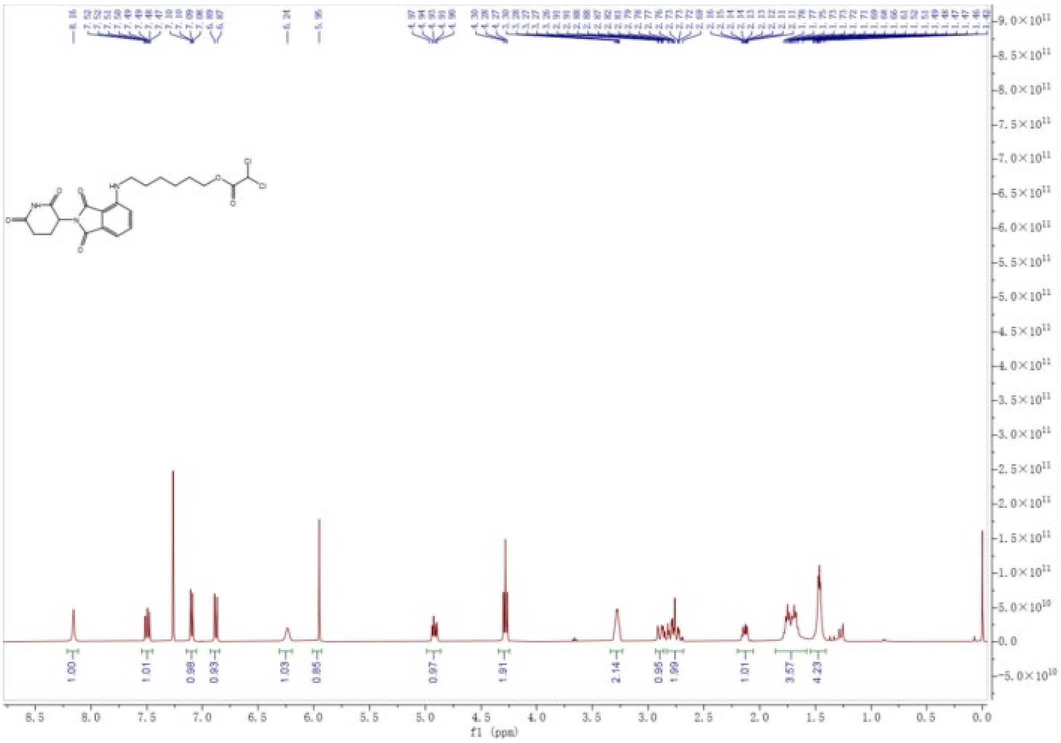

Figure S68 ^{1}H-NMR spectrum of B04

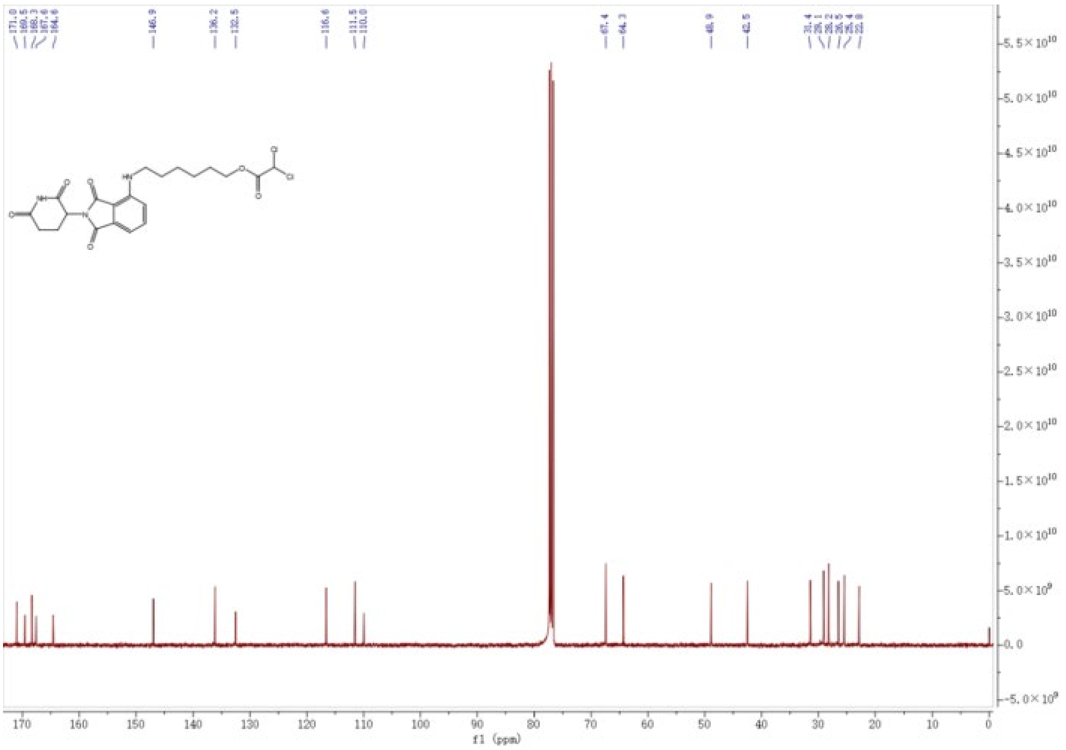

Figure S69 ^{13}C-NMR spectrum of B04

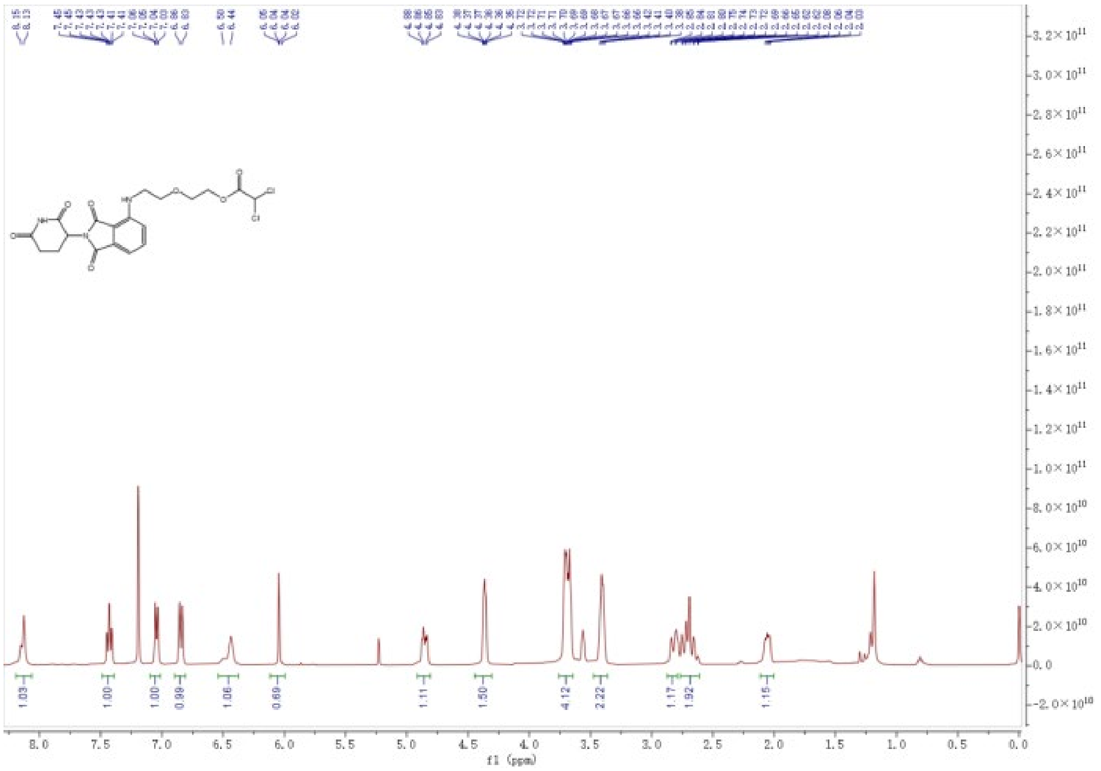

Figure S70 ^{1}H-NMR spectrum of B05

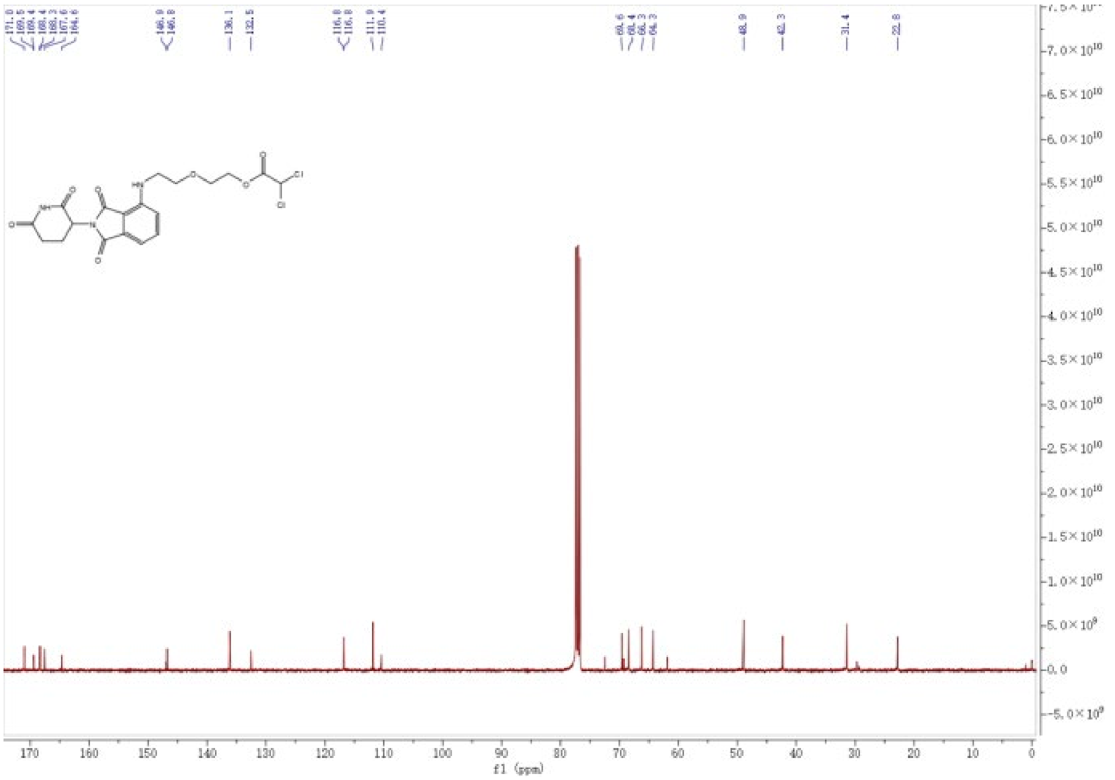

Figure S71 ^{13}C-NMR spectrum of B05

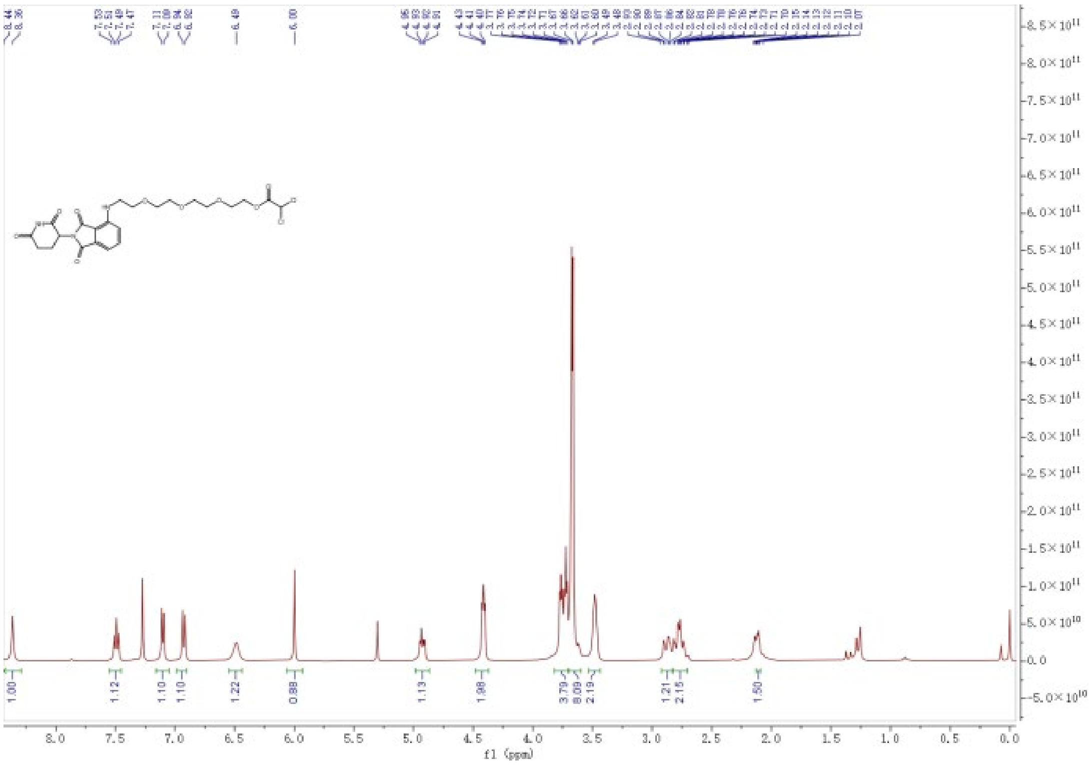

Figure S72 ^{1}H-NMR spectrum of B06

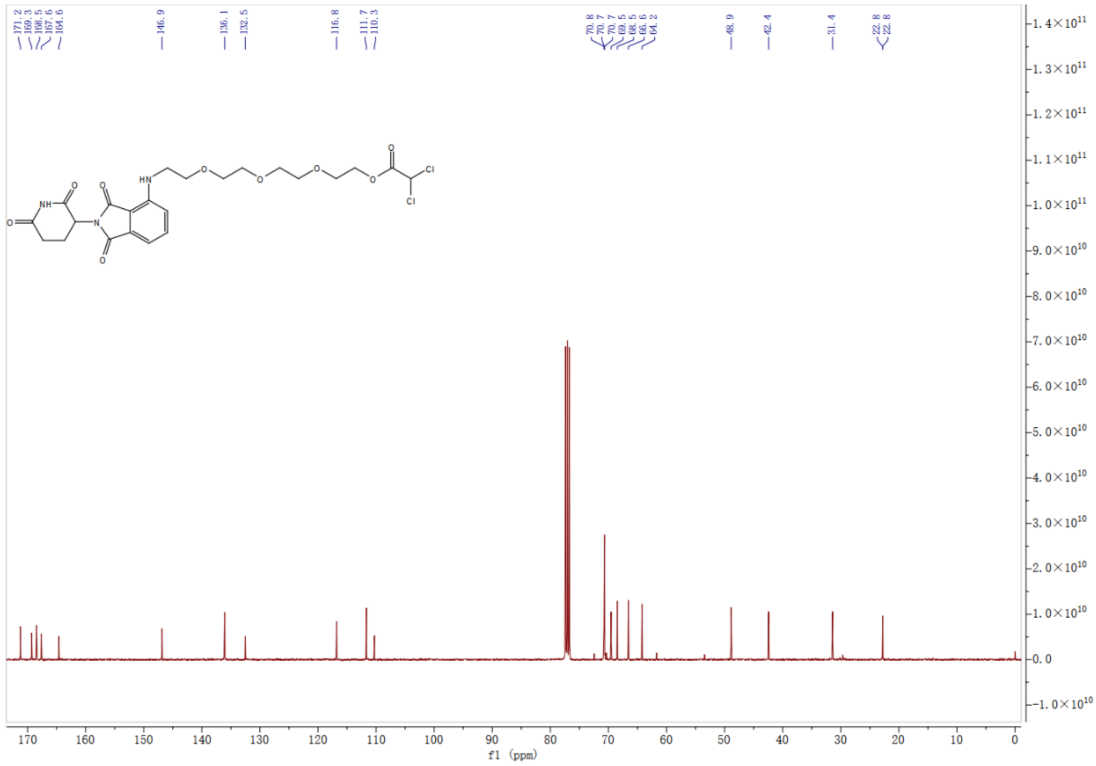

Figure S73 ^{13}C-NMR spectrum of B06

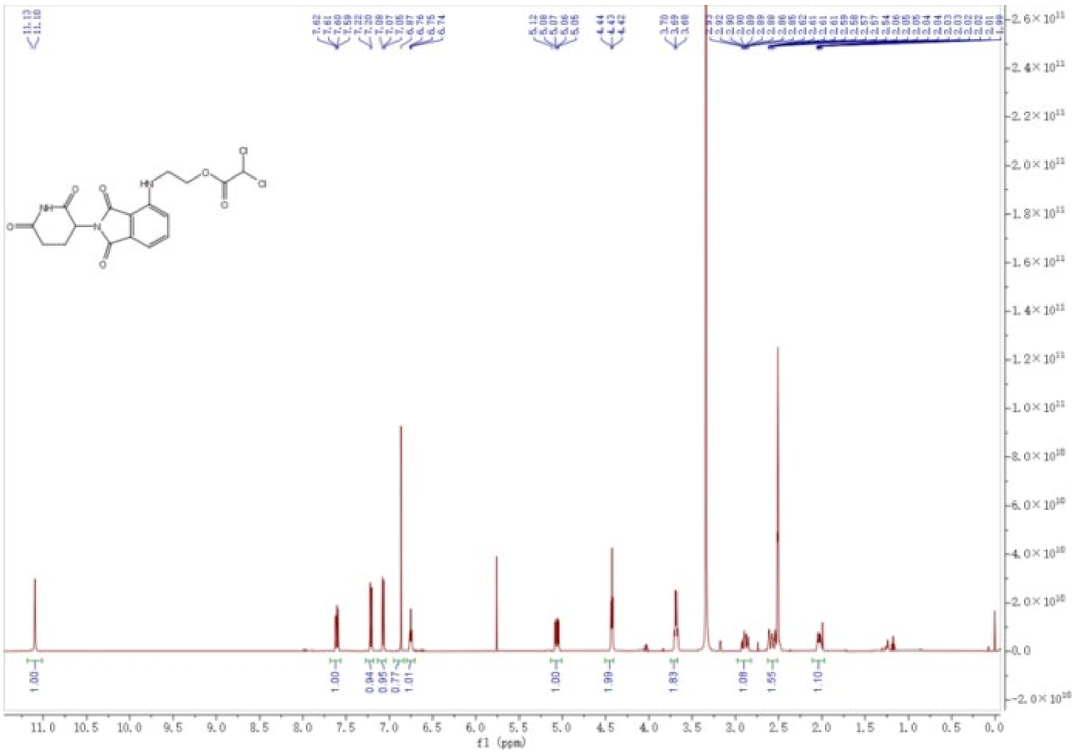

Figure S74 ^{1}H-NMR spectrum of B07

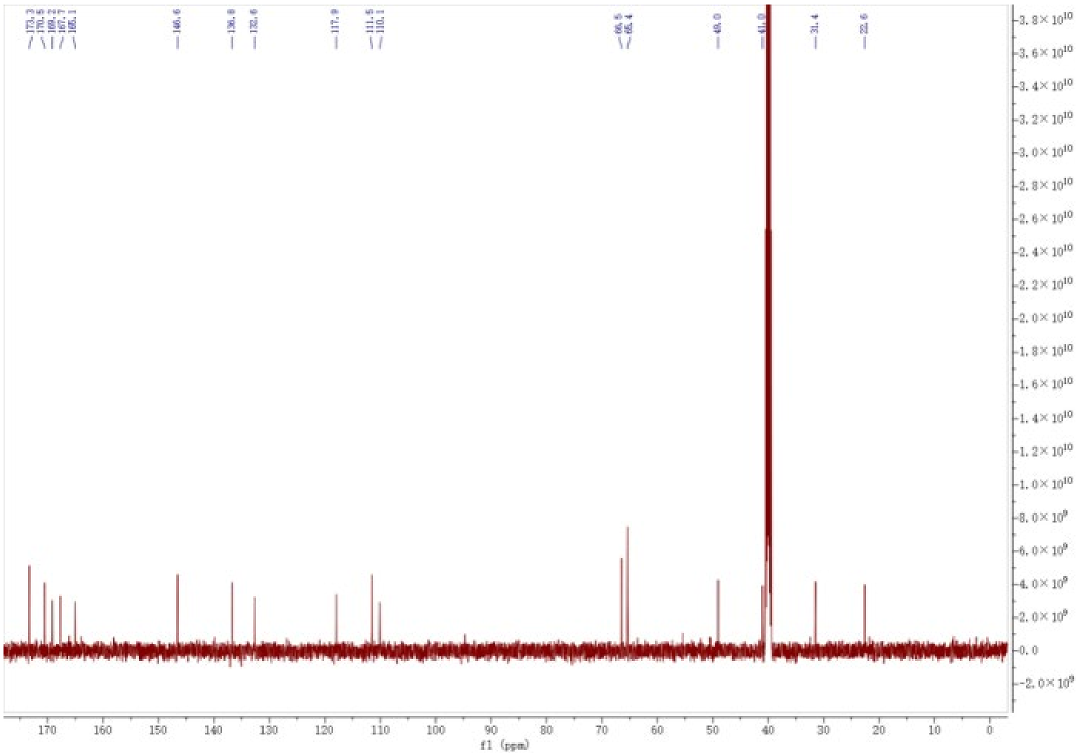

Figure S75 ^{13}C-NMR spectrum of B07

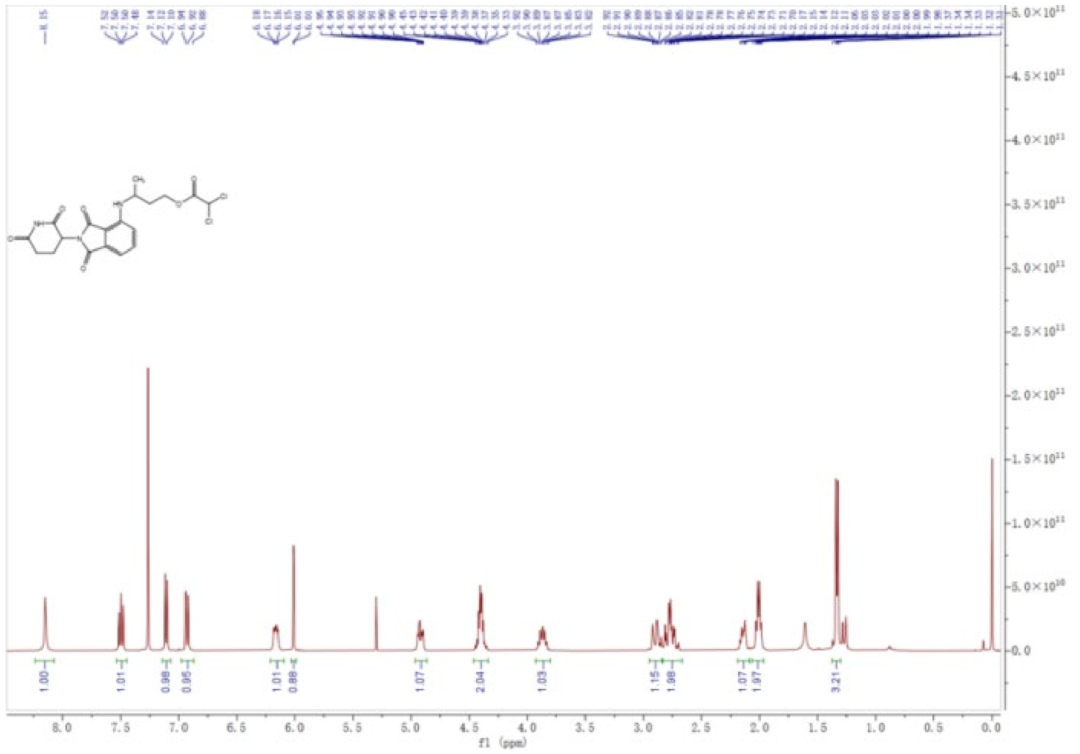

Figure S76 ^{1}H-NMR spectrum of B08

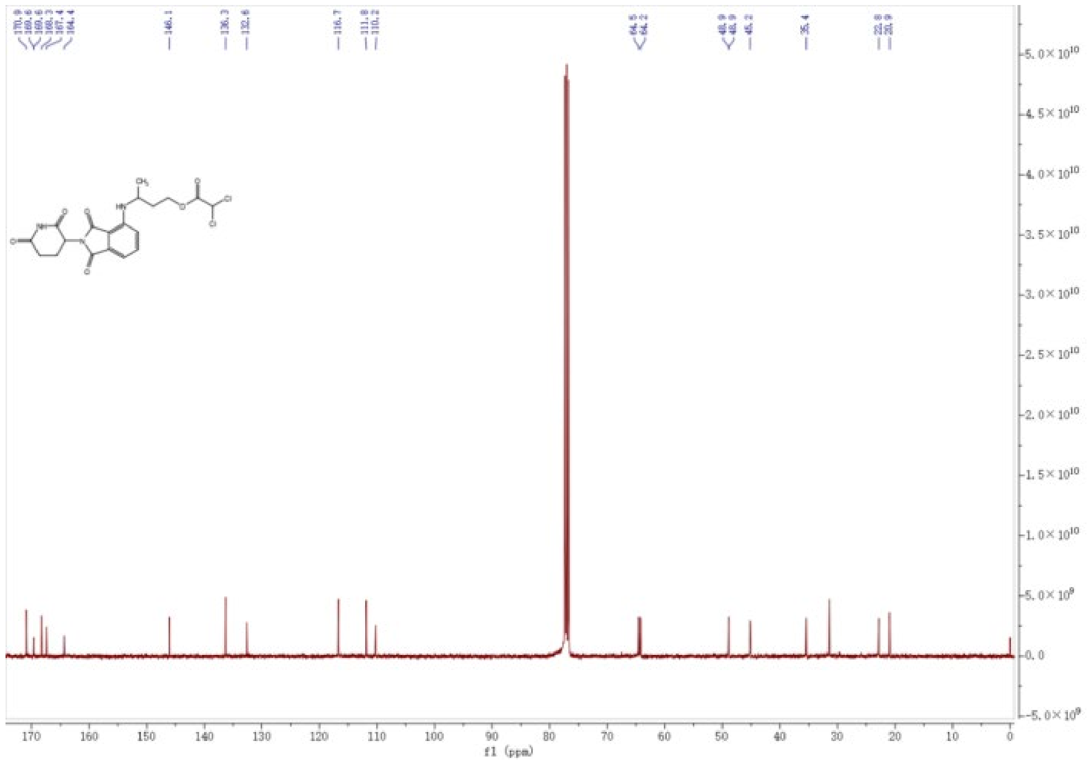

Figure S77 ^{13}C-NMR spectrum of B08

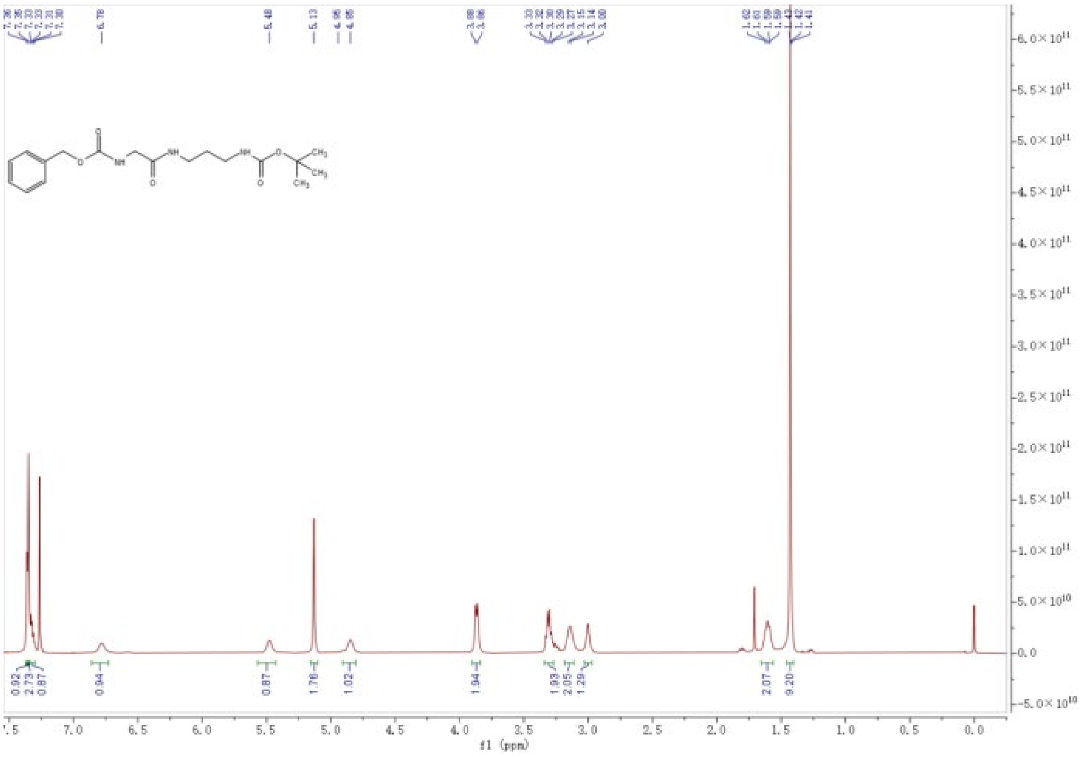

Figure S78 ^{1}H-NMR spectrum of 5a

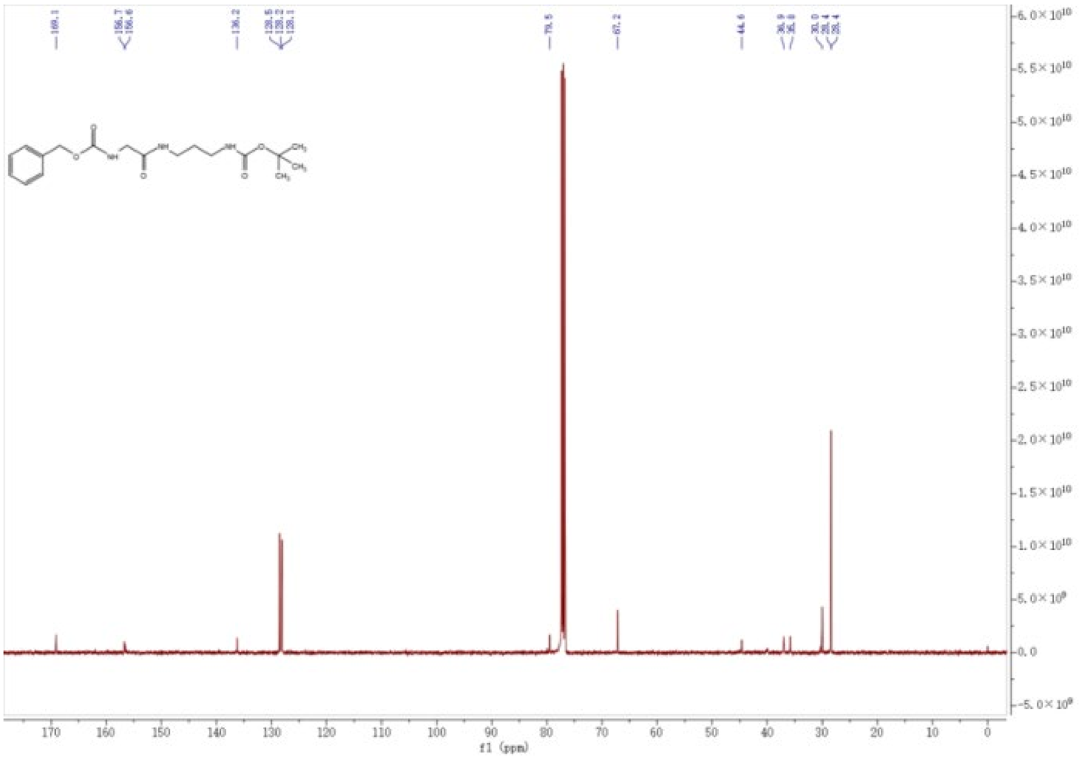

Figure S79 ^{13}C-NMR spectrum of 5a

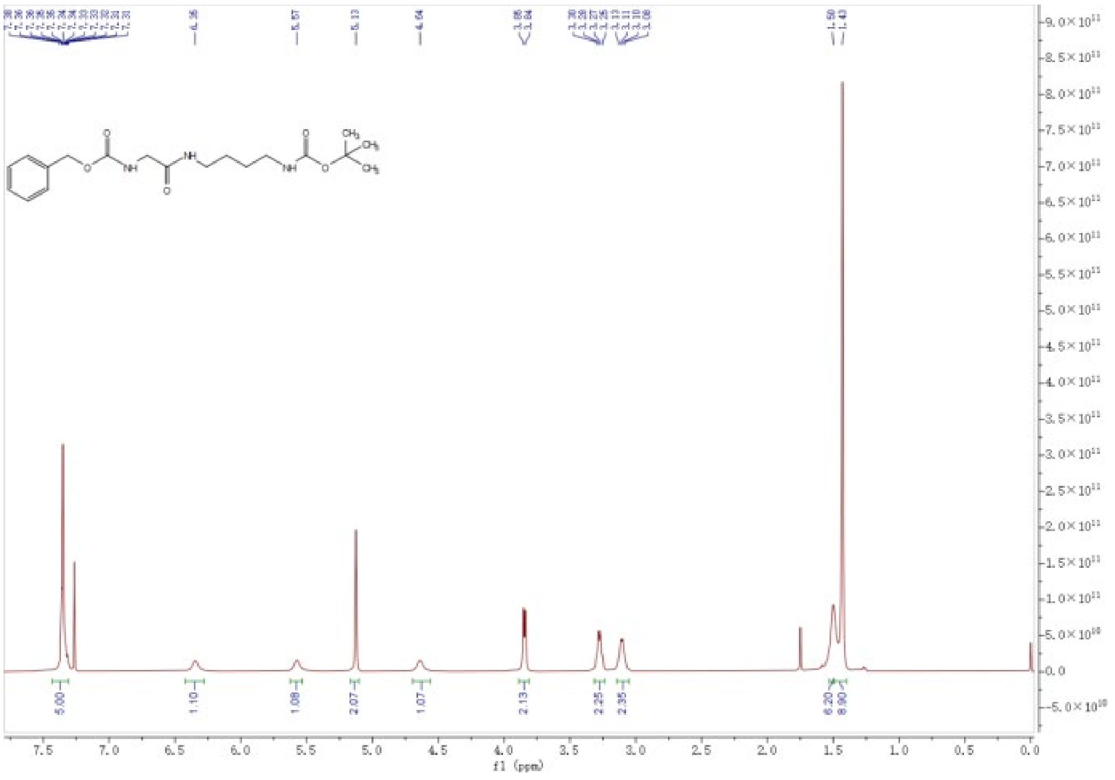

Figure S80 ^{1}H-NMR spectrum of 5b

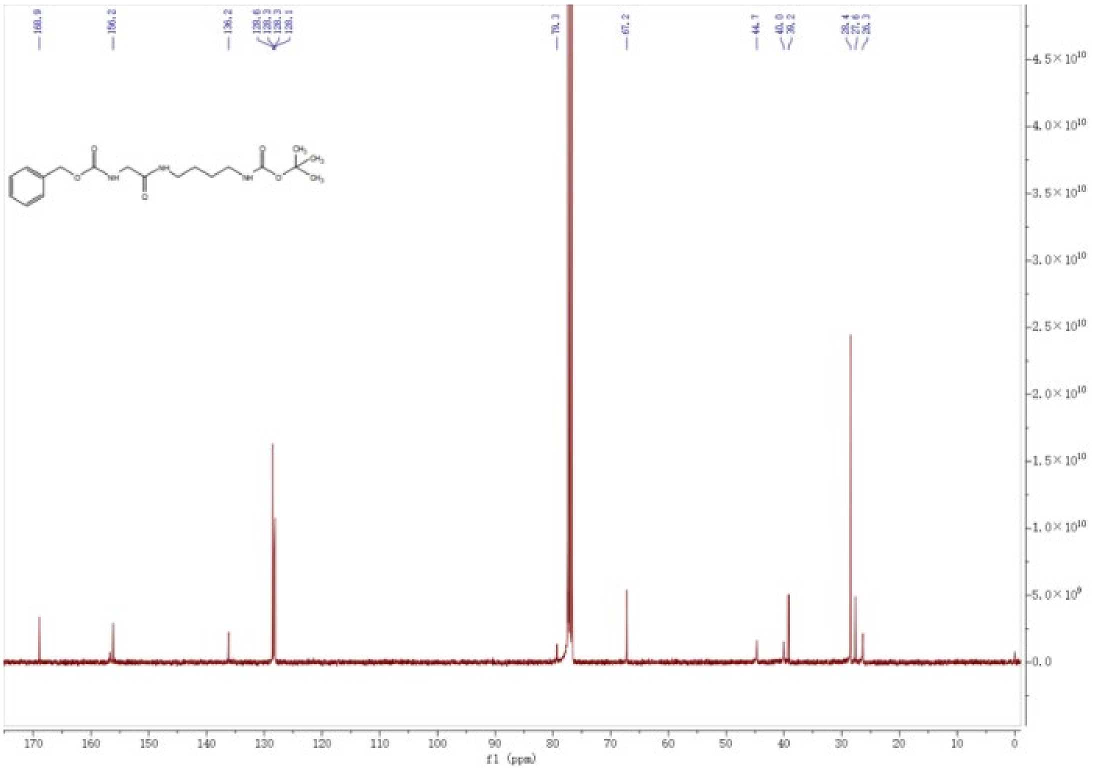

Figure S81 ^{13}C-NMR spectrum of 5b

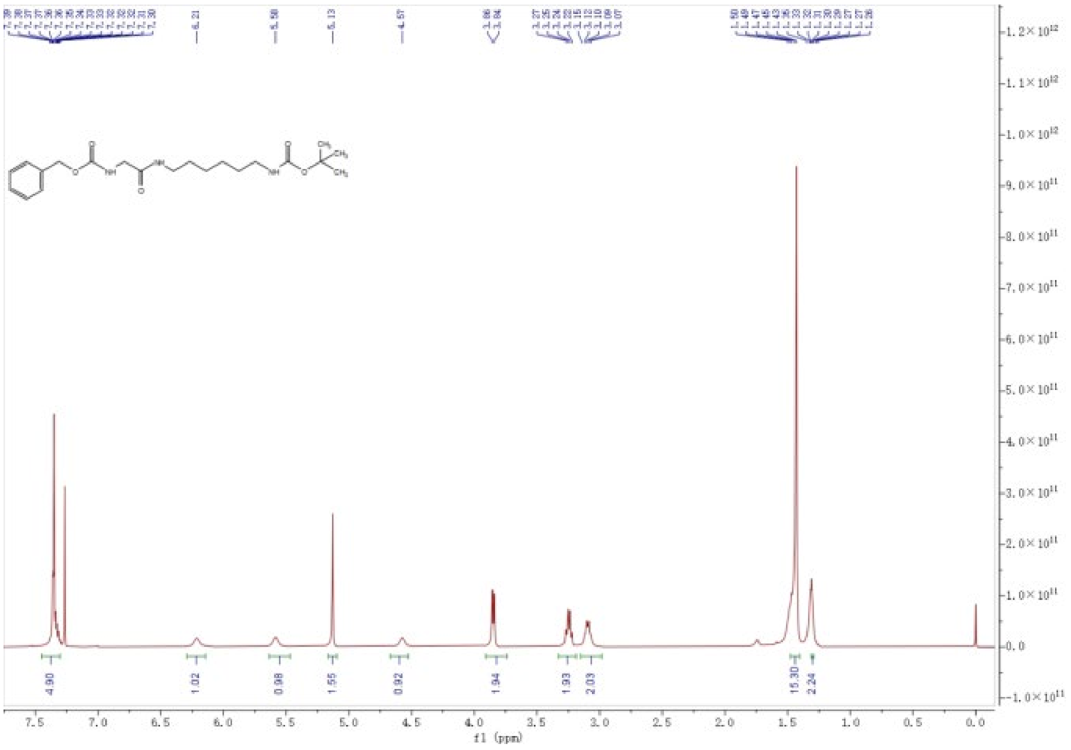

Figure S82 ^{1}H-NMR spectrum of 5c

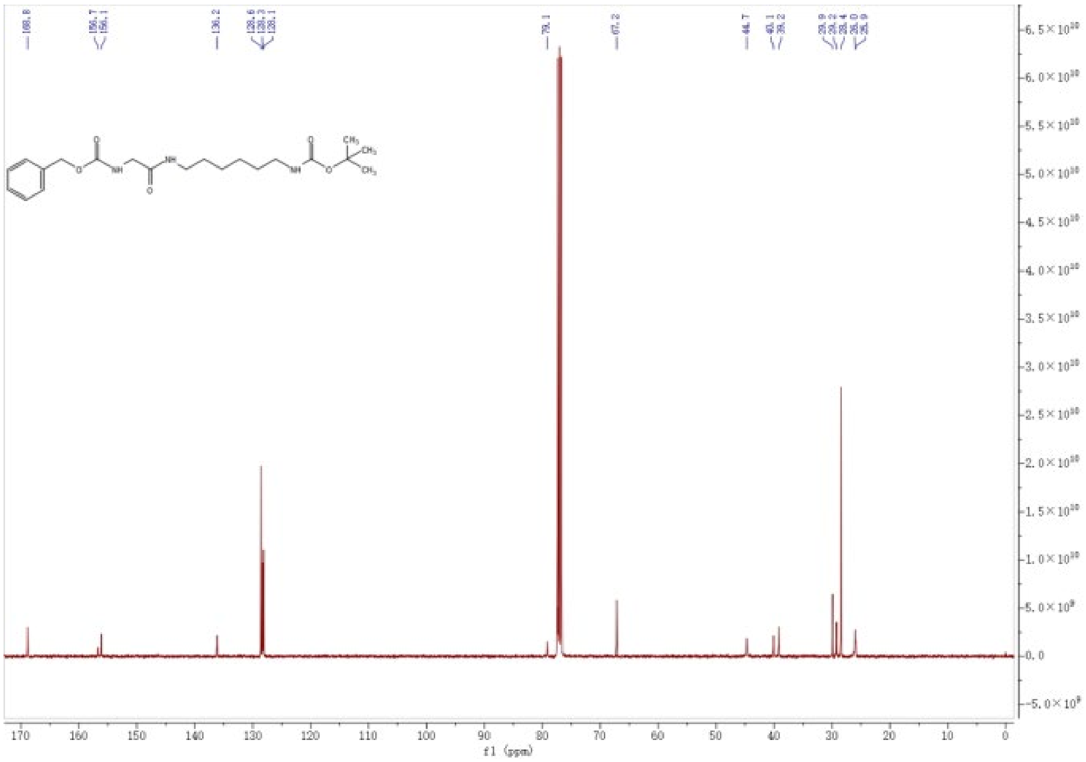

Figure S13 ^{13}C-NMR spectrum of 5c

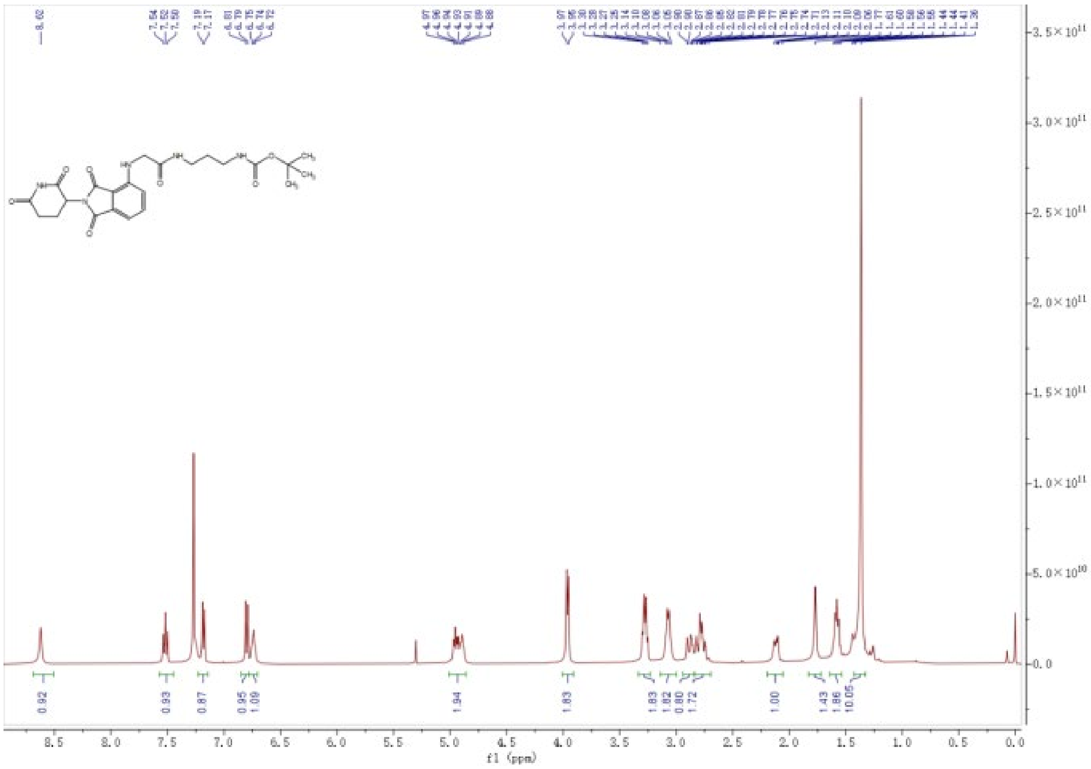

Figure S24 ^{1}H-NMR spectrum of 7a

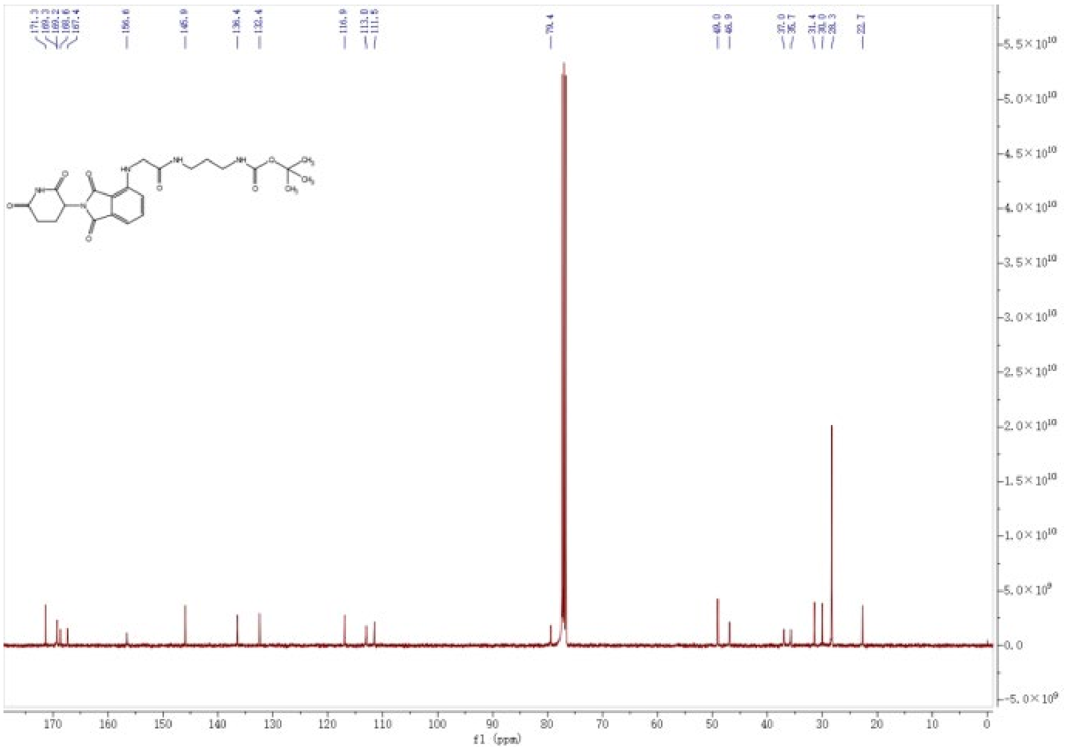

Figure S35 ^{13}C-NMR spectrum of 7a

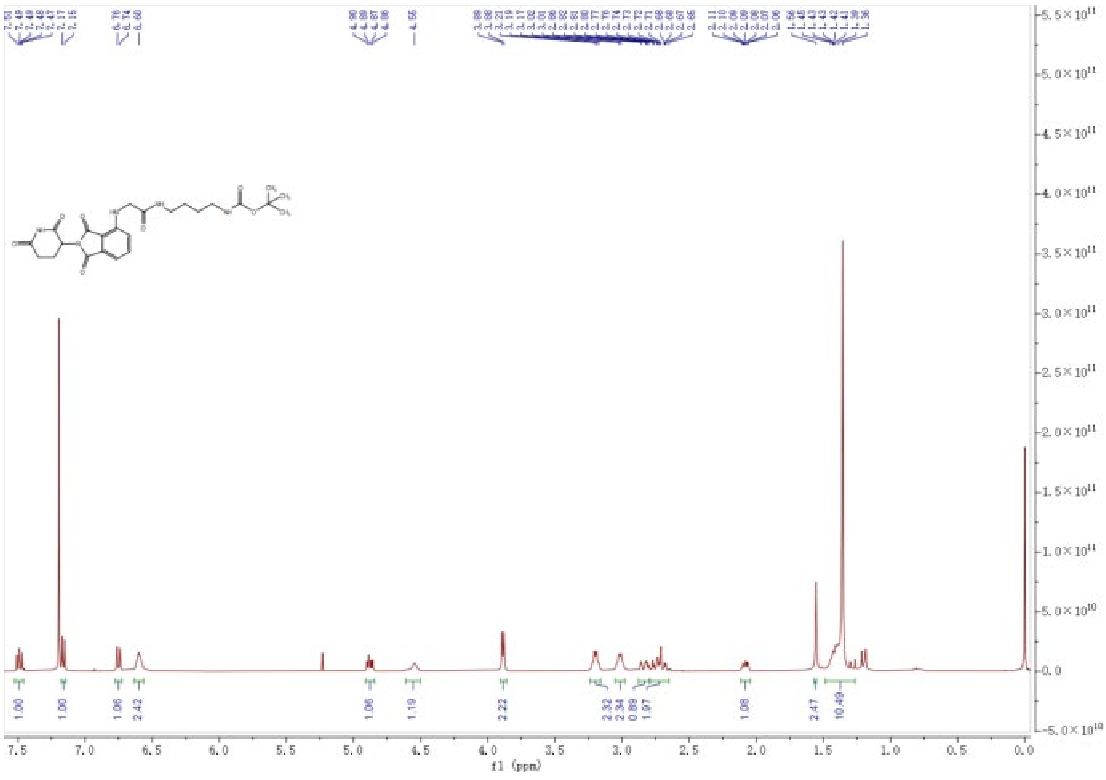

Figure S46 ¹H-NMR spectrum of 7b

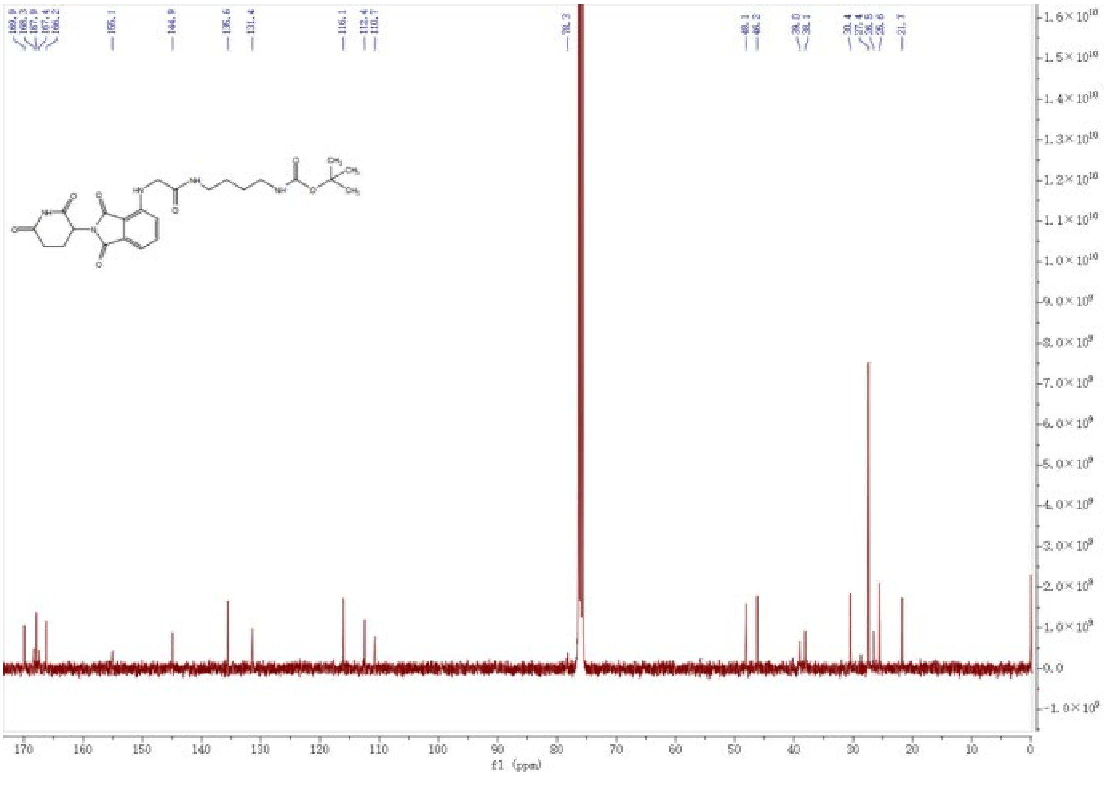

Figure S87 ¹³C-NMR spectrum of 7b

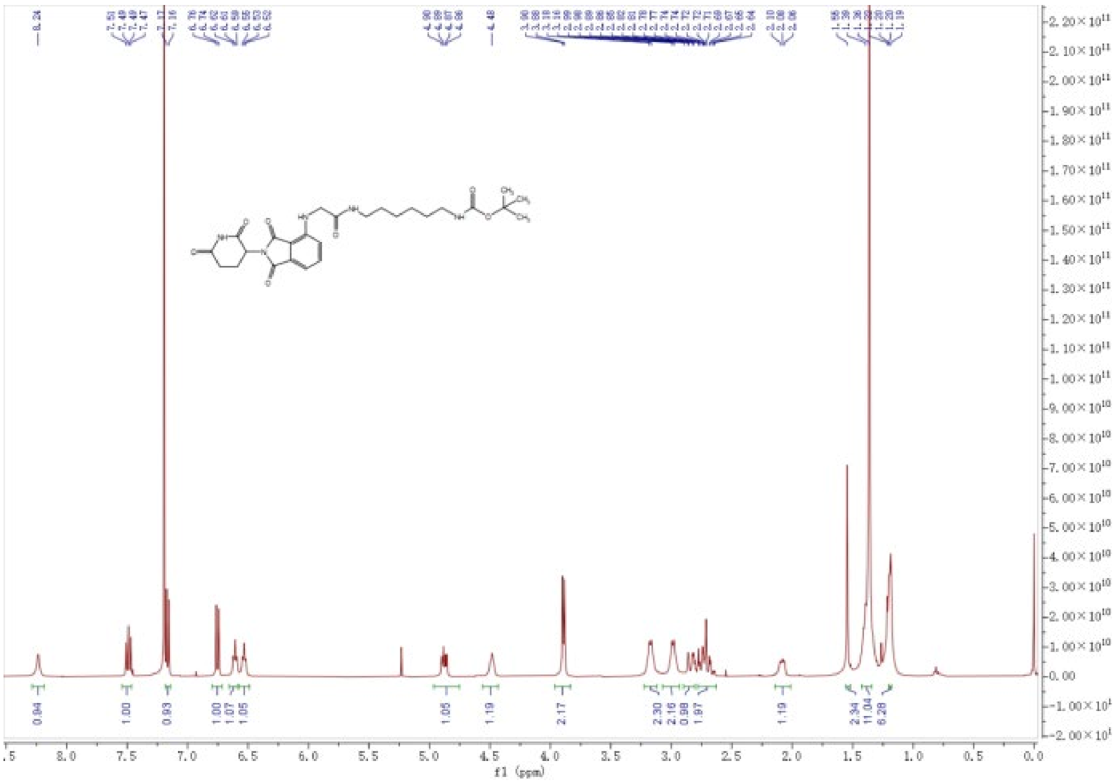

Figure S88 ^{1}H-NMR spectrum of 7c

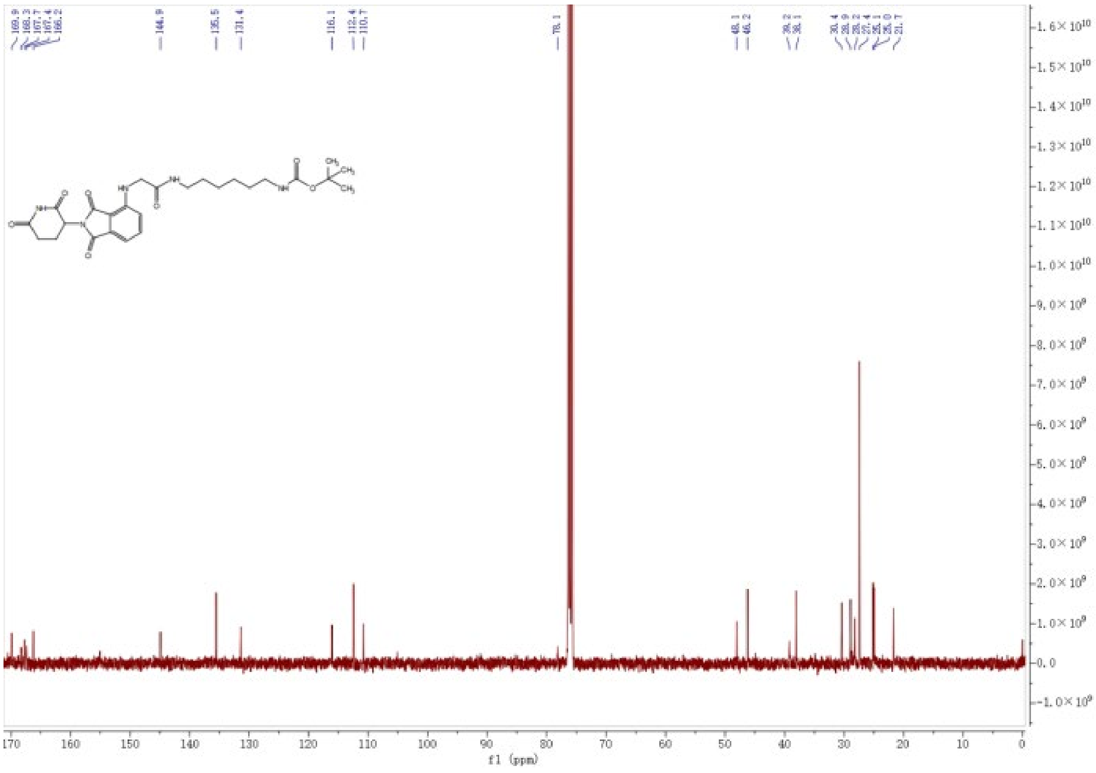

Figure S89 ^{13}C-NMR spectrum of 7c

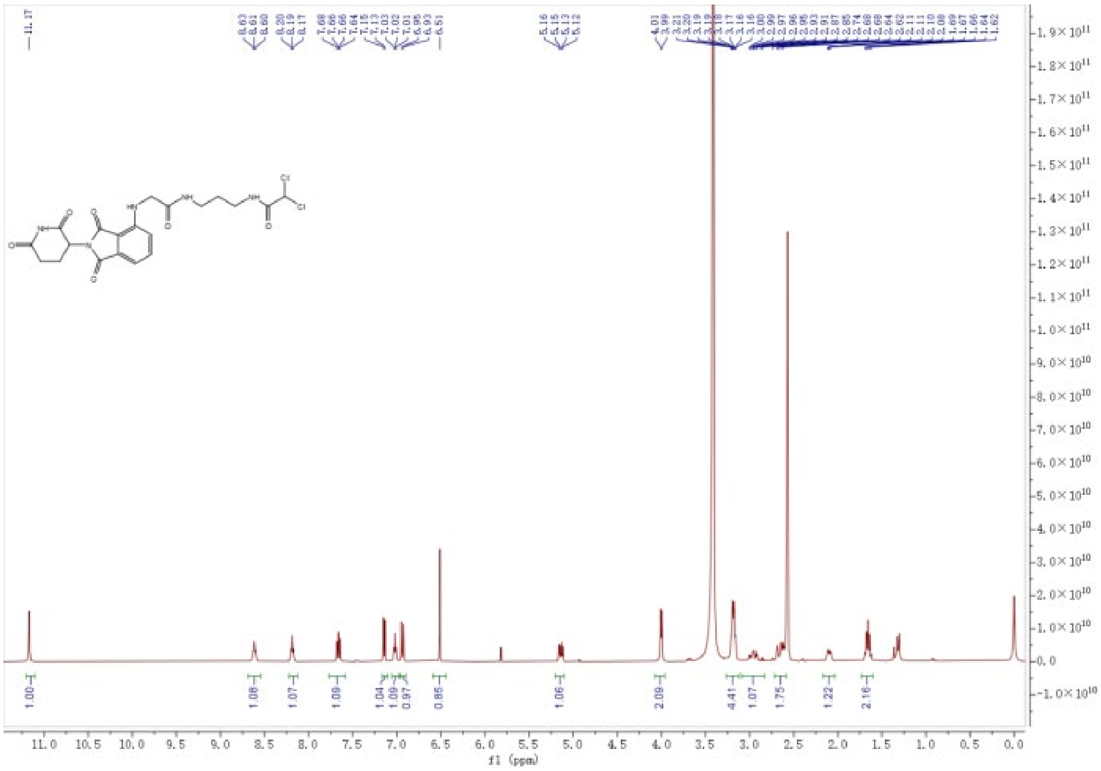

Figure S90 ^{1}H-NMR spectrum of C01

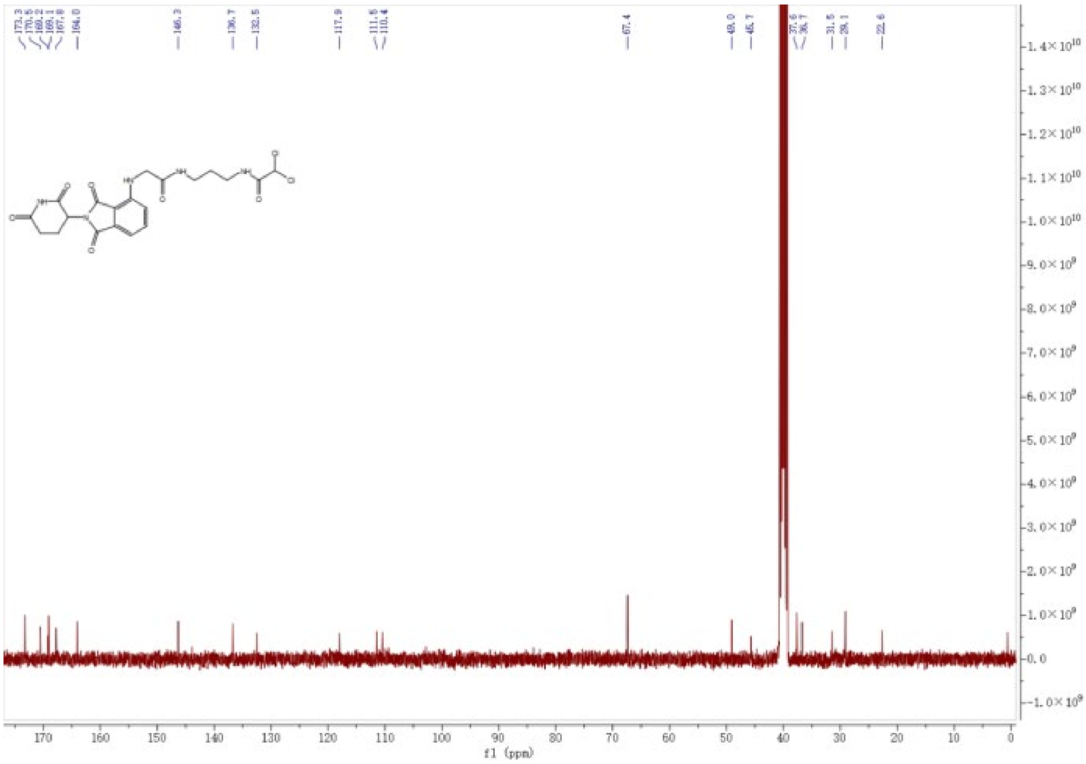

Figure S91 ^{13}C-NMR spectrum of C01

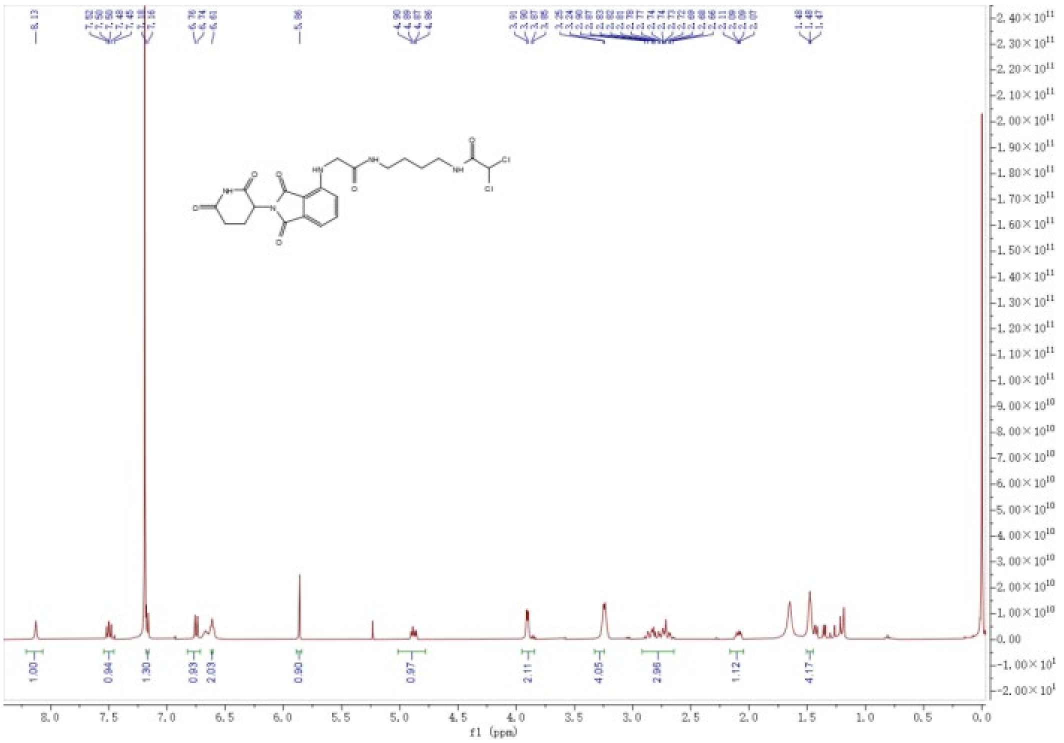

Figure S92 ^{1}H-NMR spectrum of C02

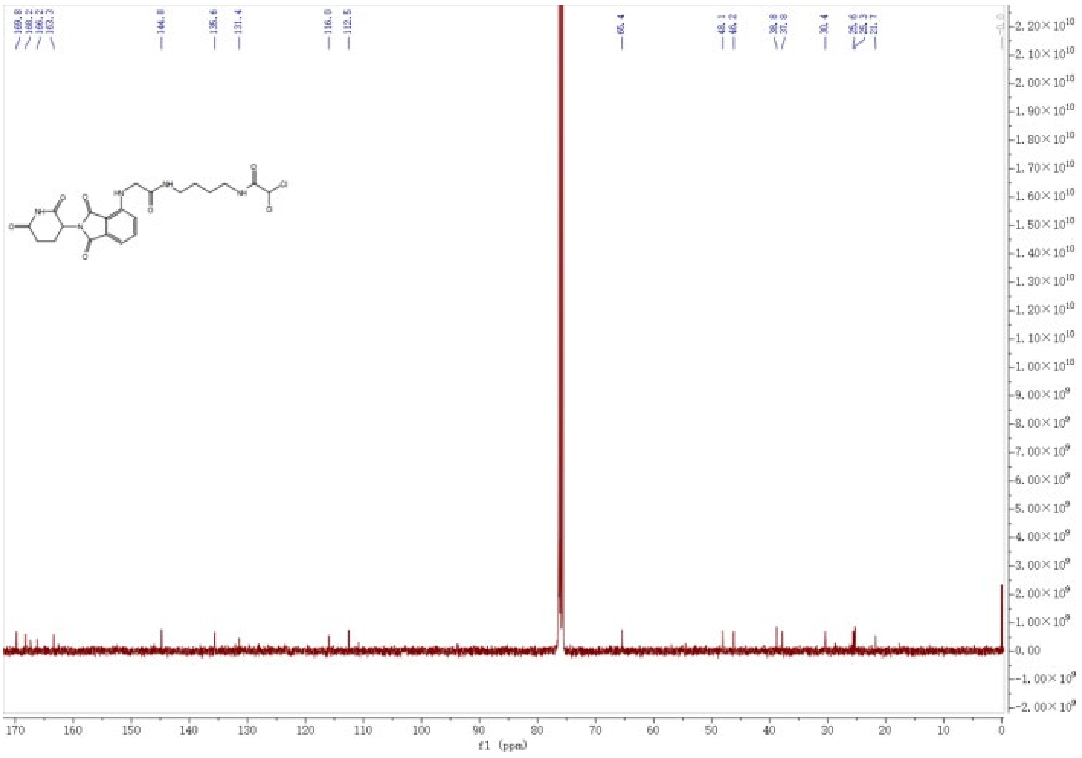

Figure S93 ^{13}C-NMR spectrum of C02

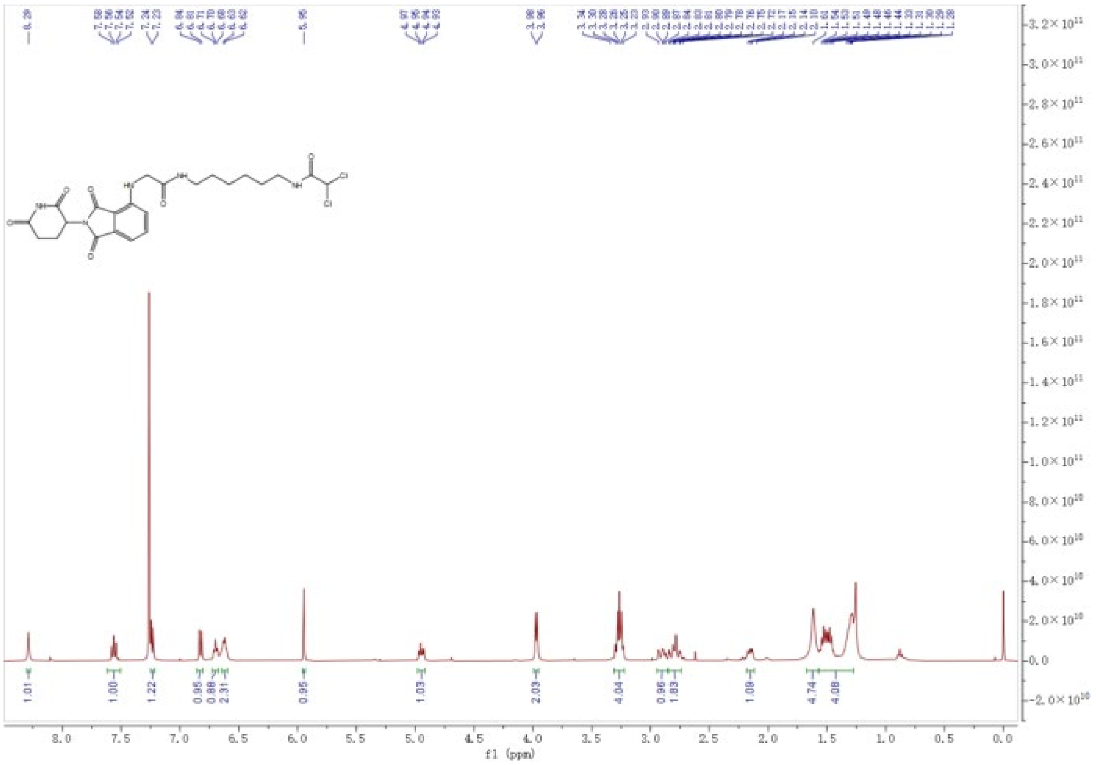

Figure S94 ^{1}H-NMR spectrum of C03

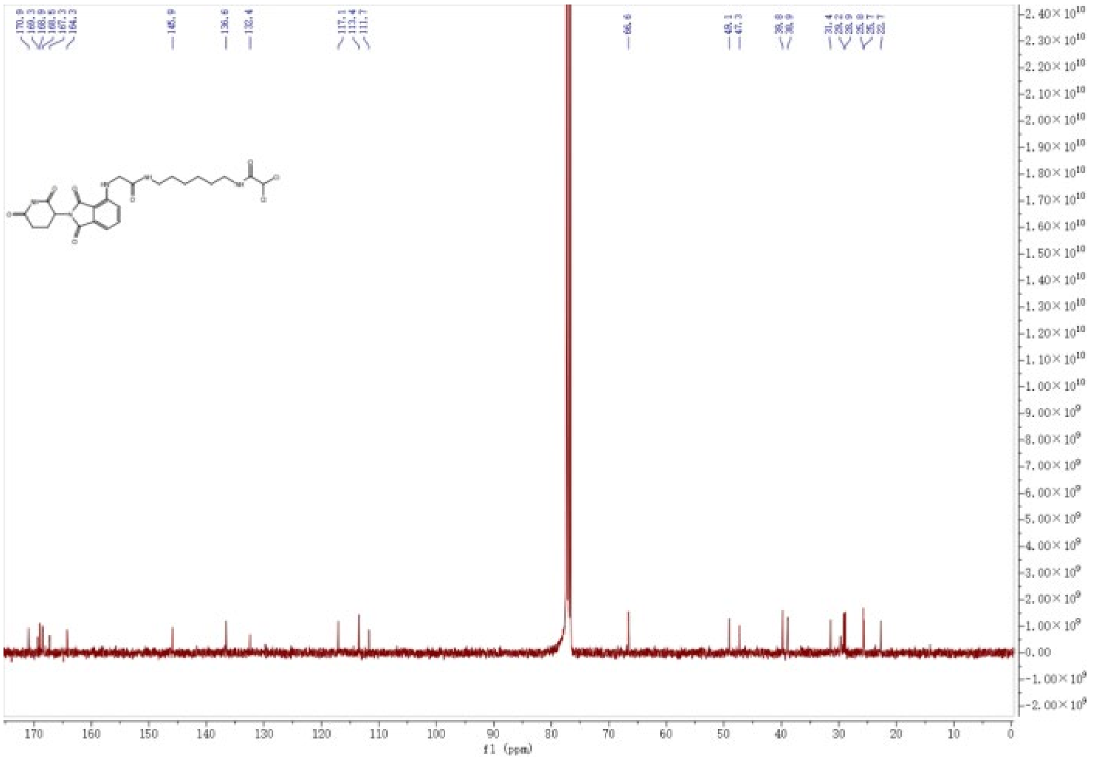

Figure S95 ^{13}C-NMR spectrum of C03

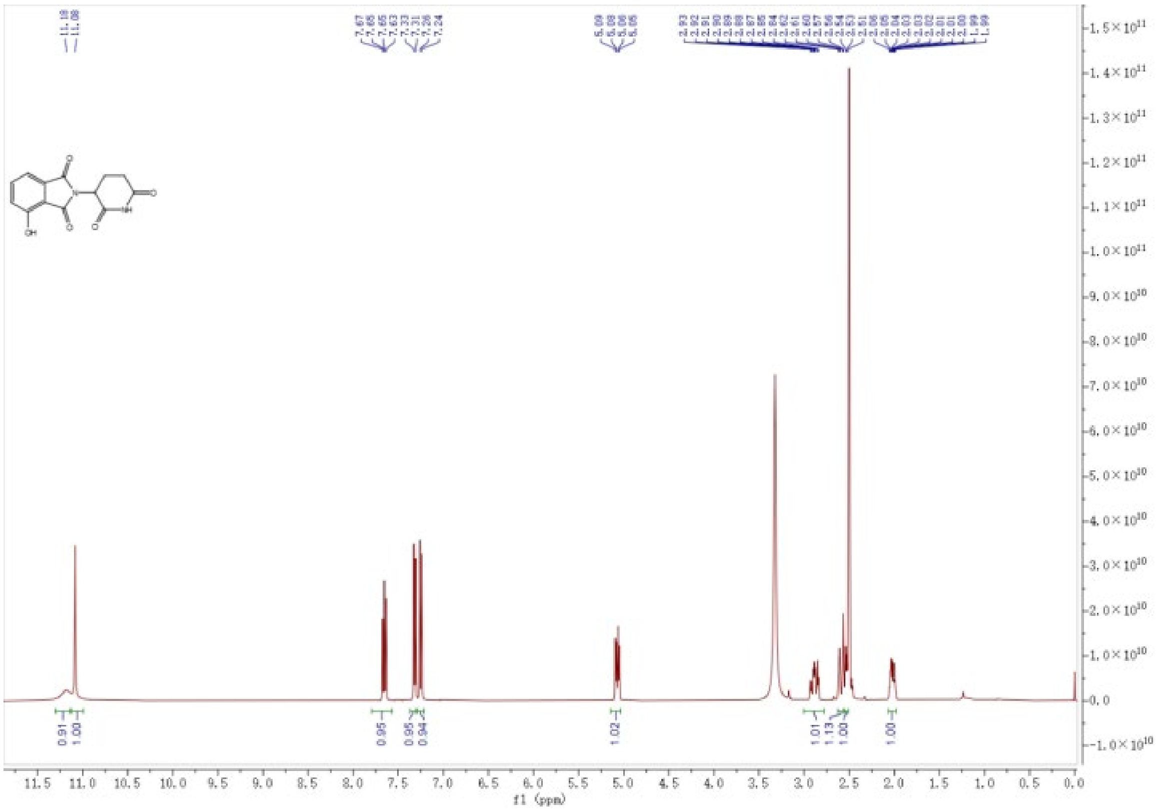

Figure S96 ^{1}H-NMR spectrum of 9

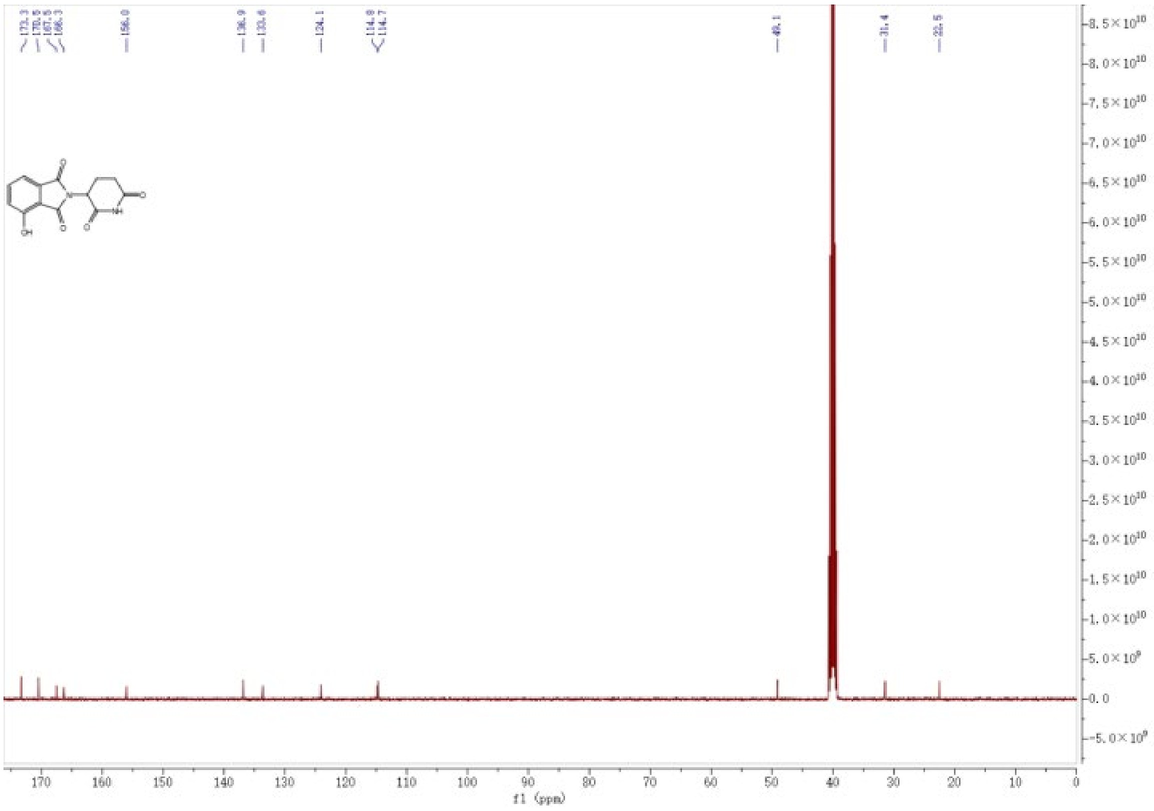

Figure S97 ^{13}C-NMR spectrum of 9

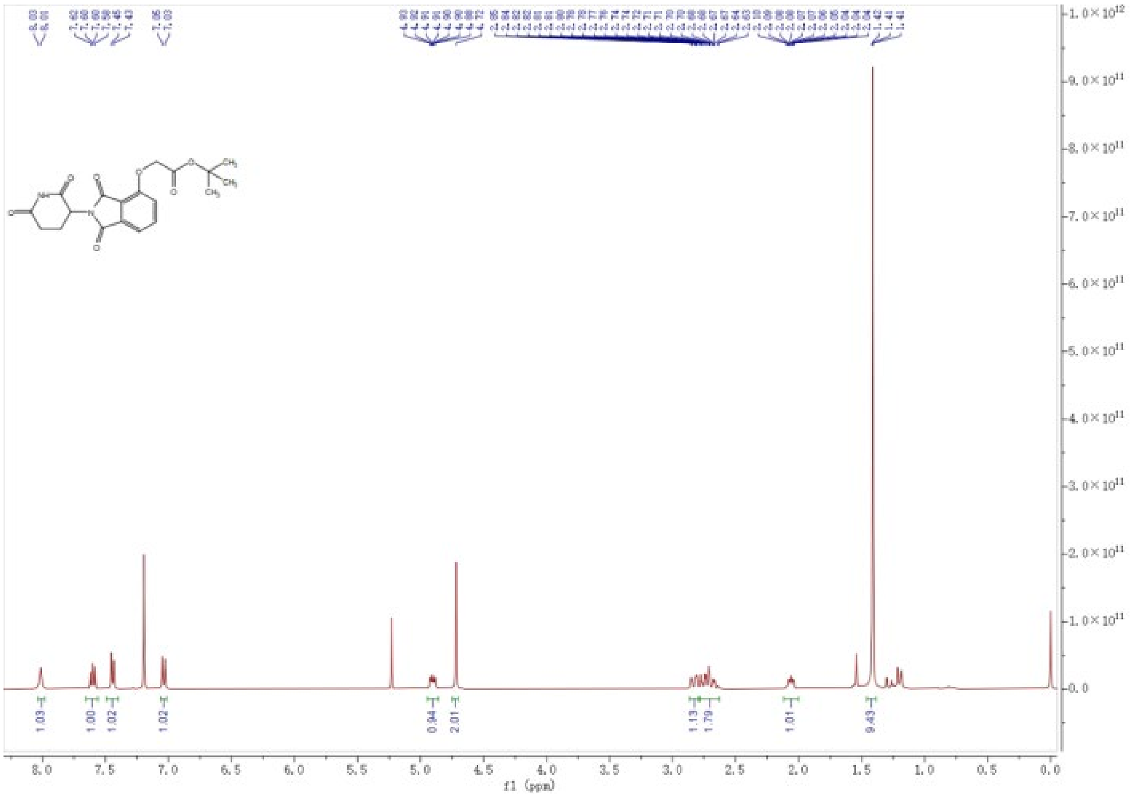

Figure S98 ^{1}H-NMR spectrum of 10

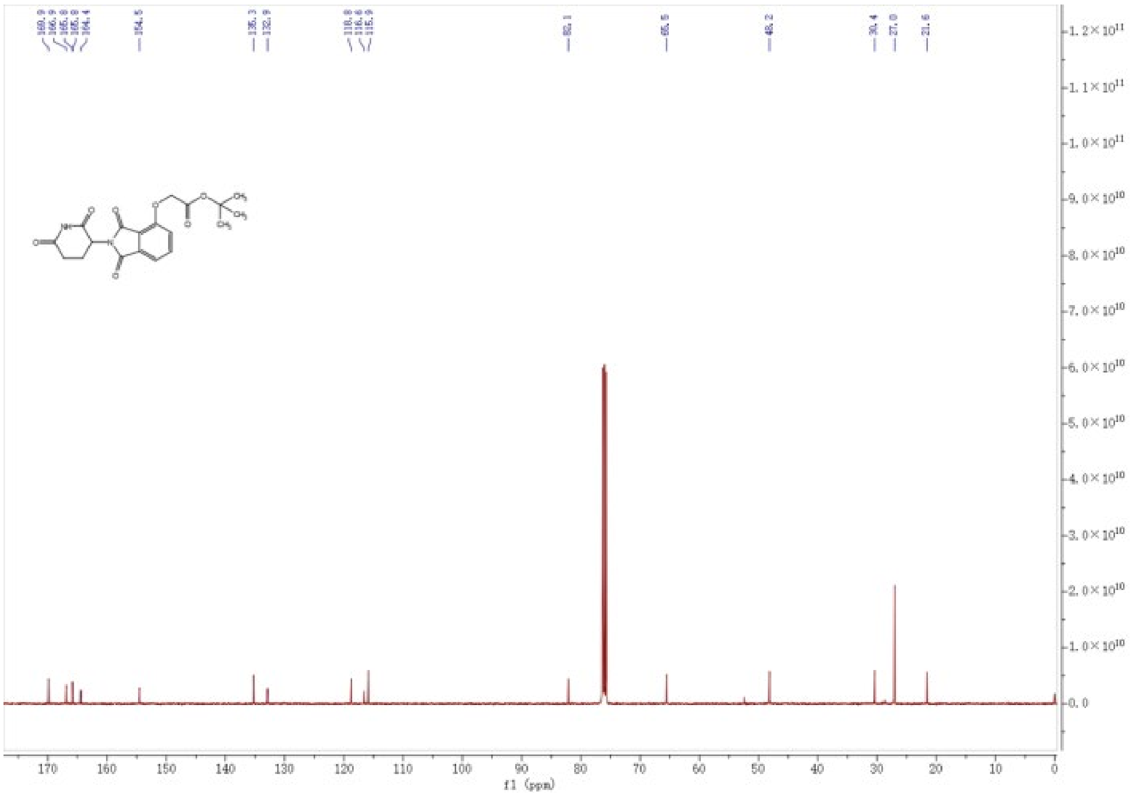

Figure S99 ^{13}C-NMR spectrum of 10

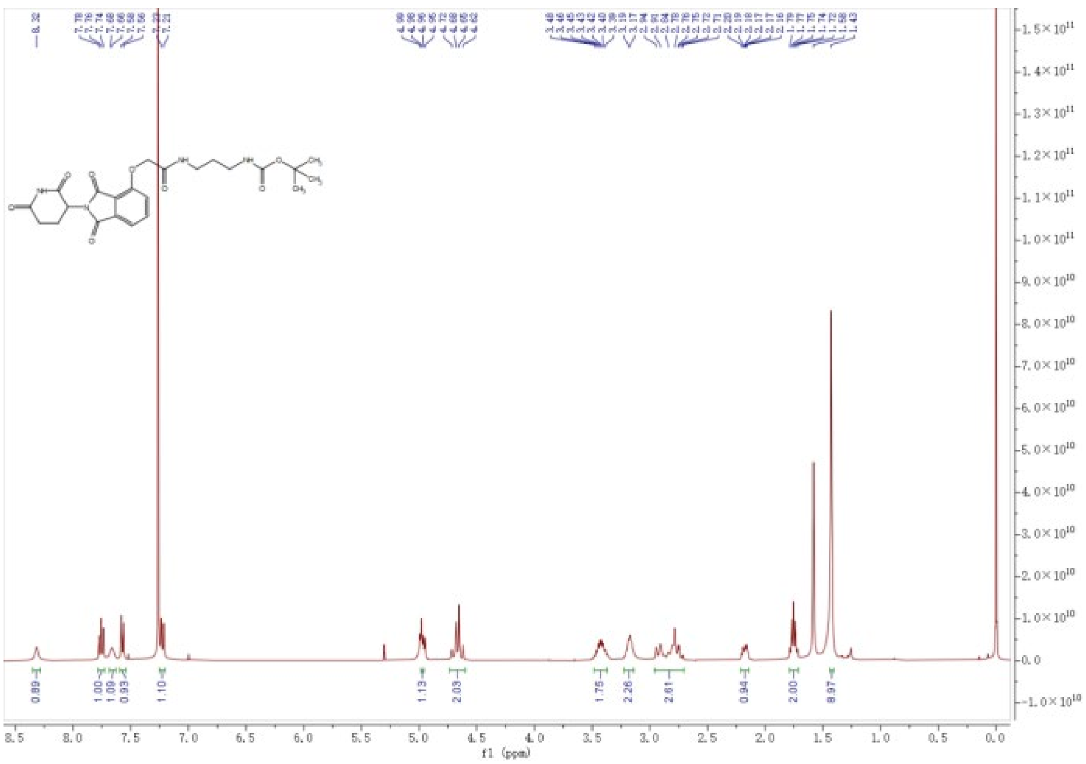

Figure S50 ^{1}H-NMR spectrum of 12a

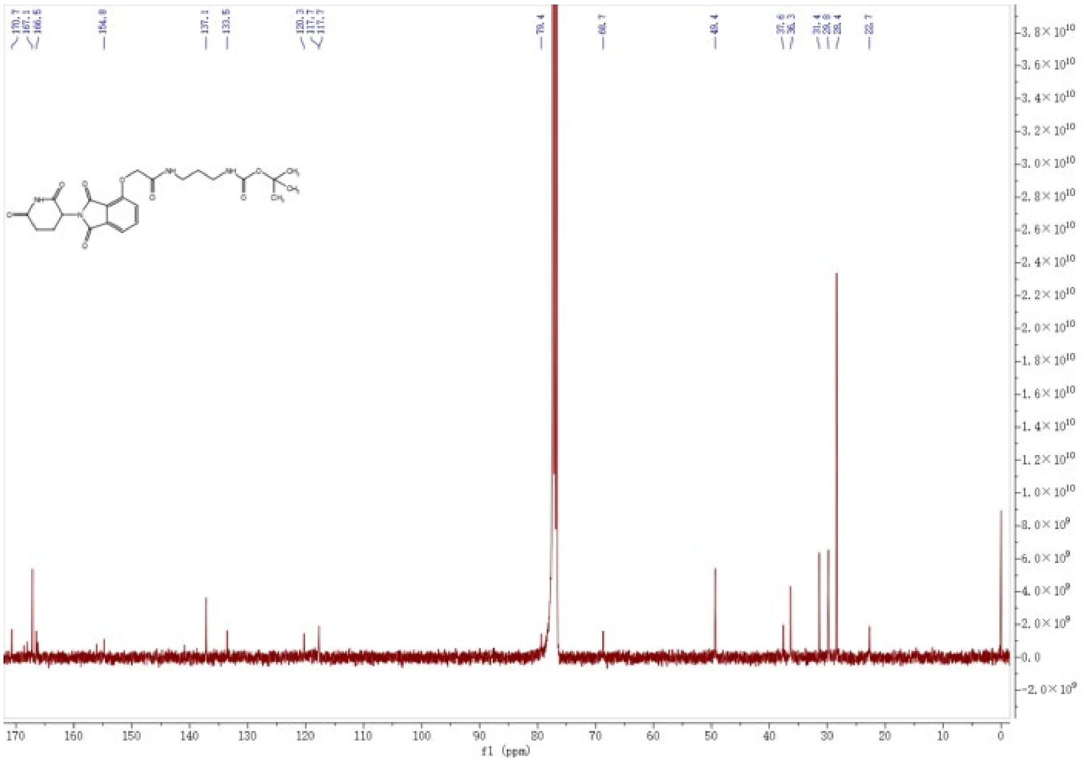

Figure S101 ^{13}C-NMR spectrum of 12a

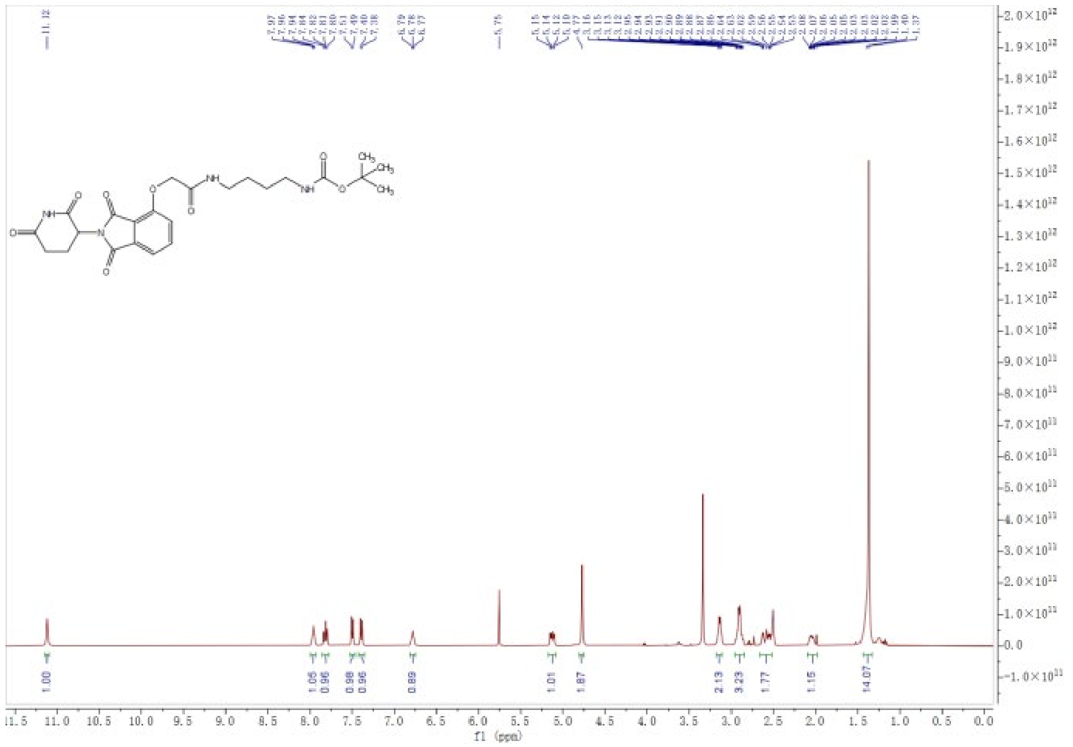

Figure S102 ^{1}H-NMR spectrum of 12b

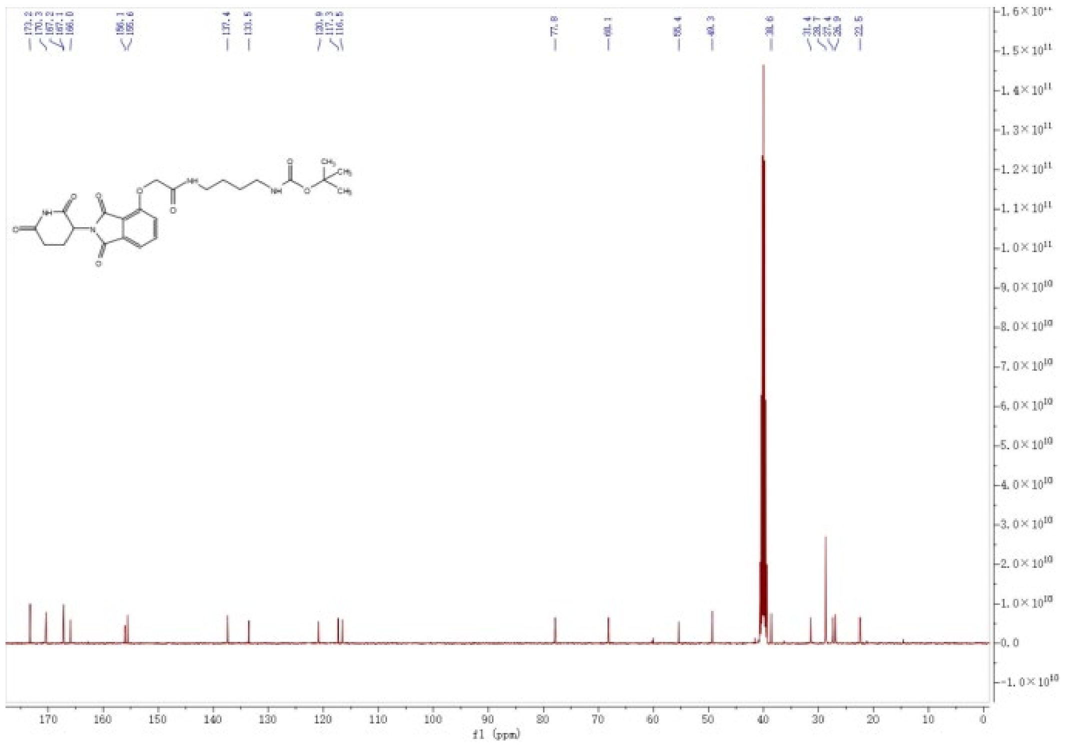

Figure S103 ^{13}C-NMR spectrum of 12b

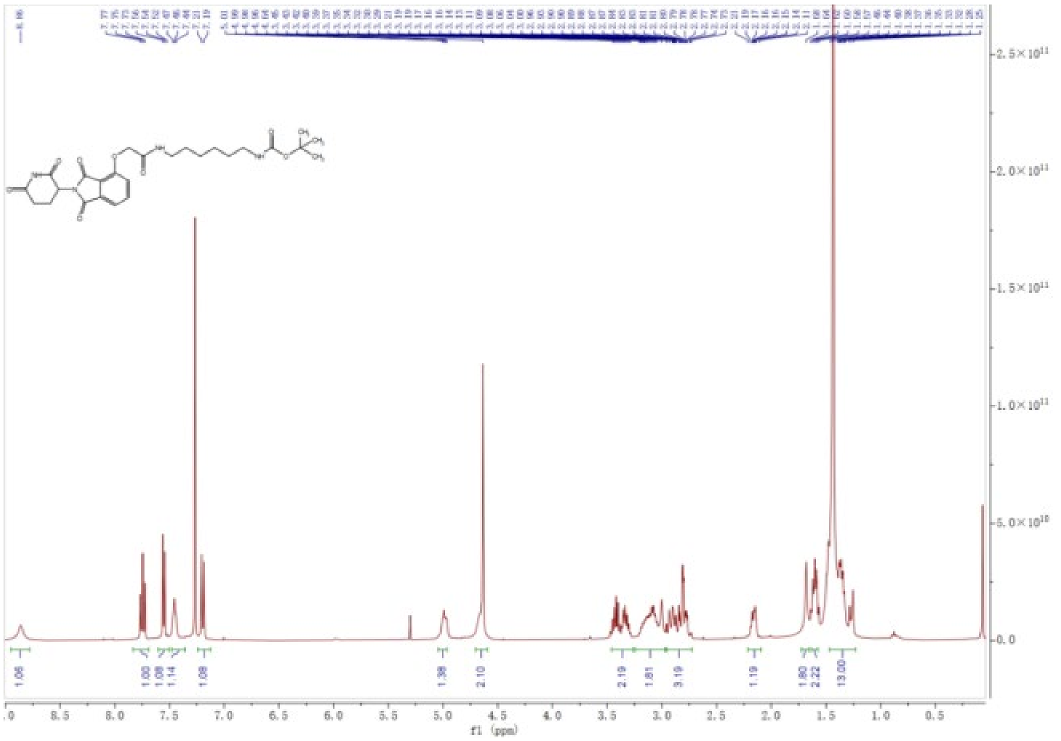

Figure S104 ^{1}H-NMR spectrum of 12c

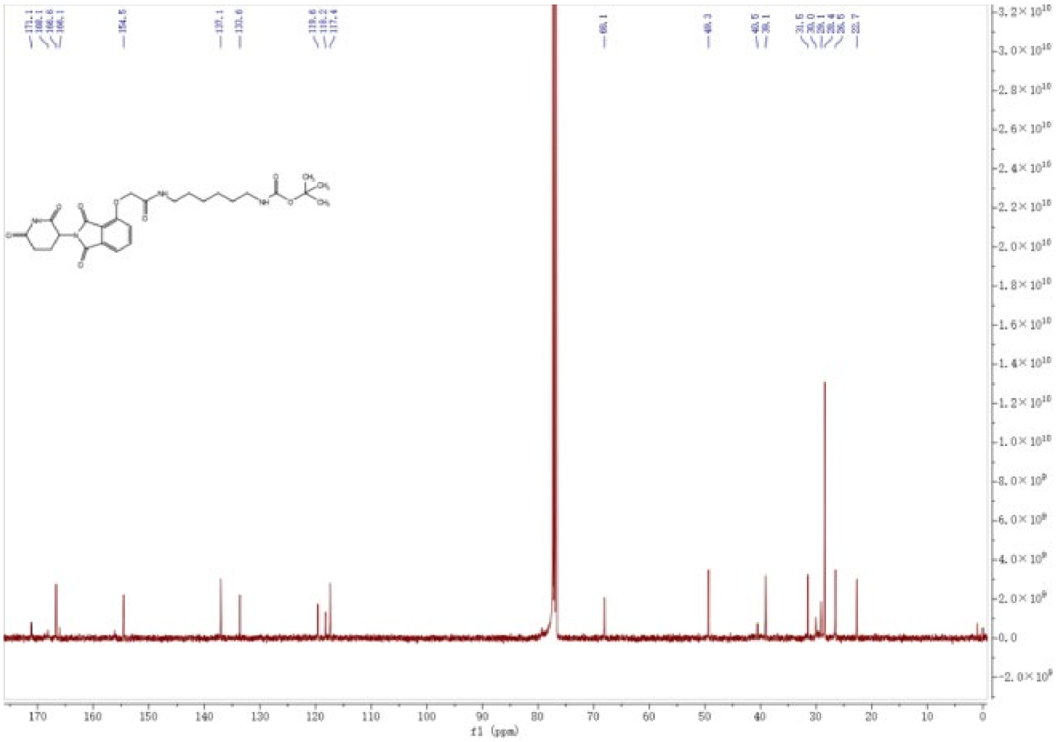

Figure S105 ^{13}C-NMR spectrum of 12c

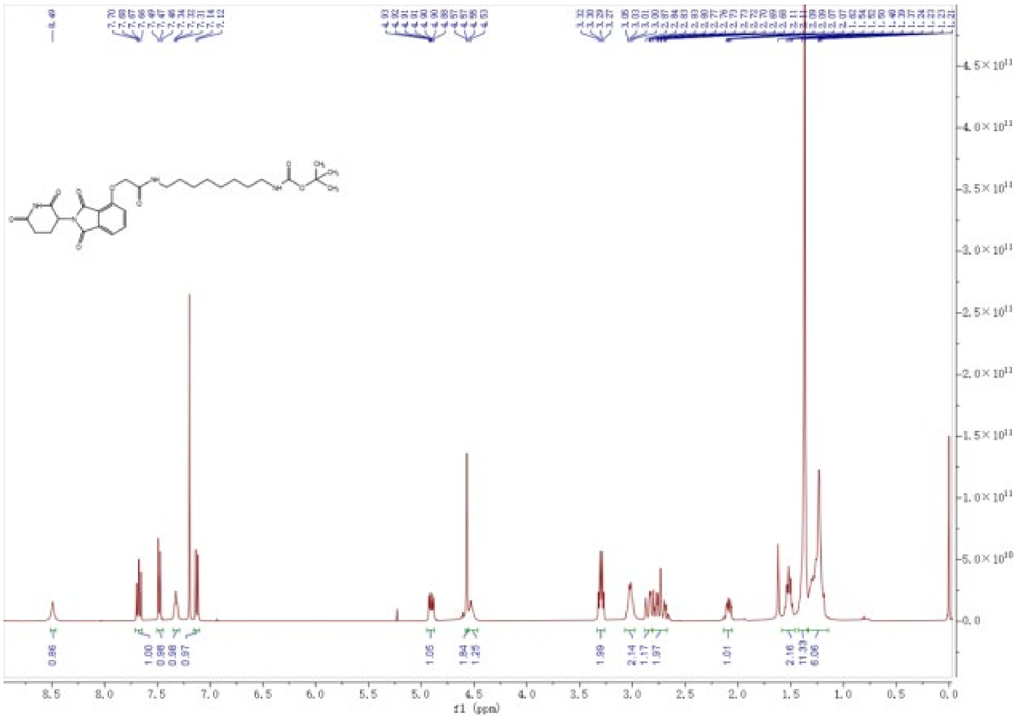

Figure S106 ^{1}H-NMR spectrum of 12d

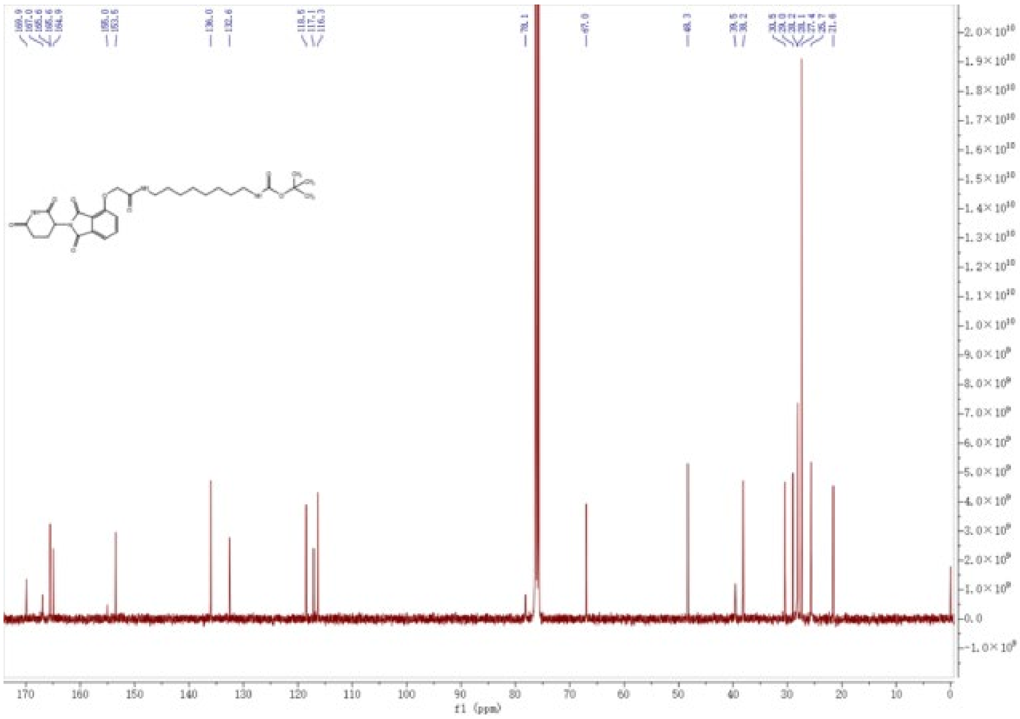

Figure S107 ^{13}C-NMR spectrum of 12d

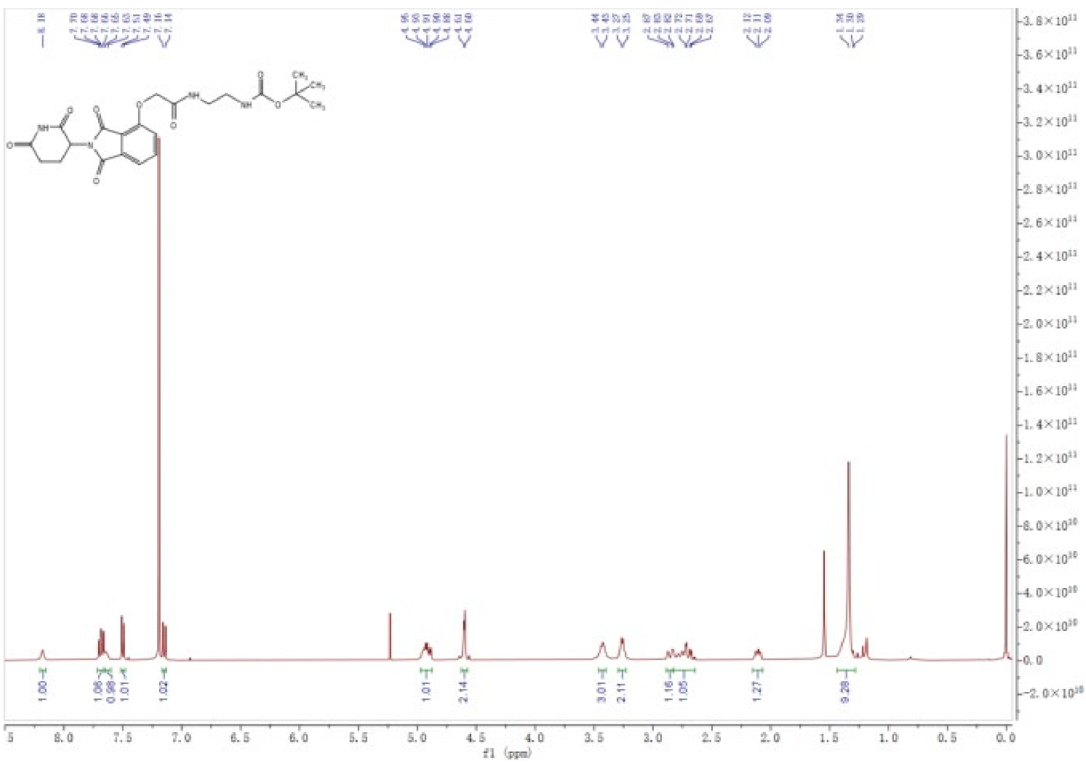

Figure S108 ^{1}H-NMR spectrum of 12e

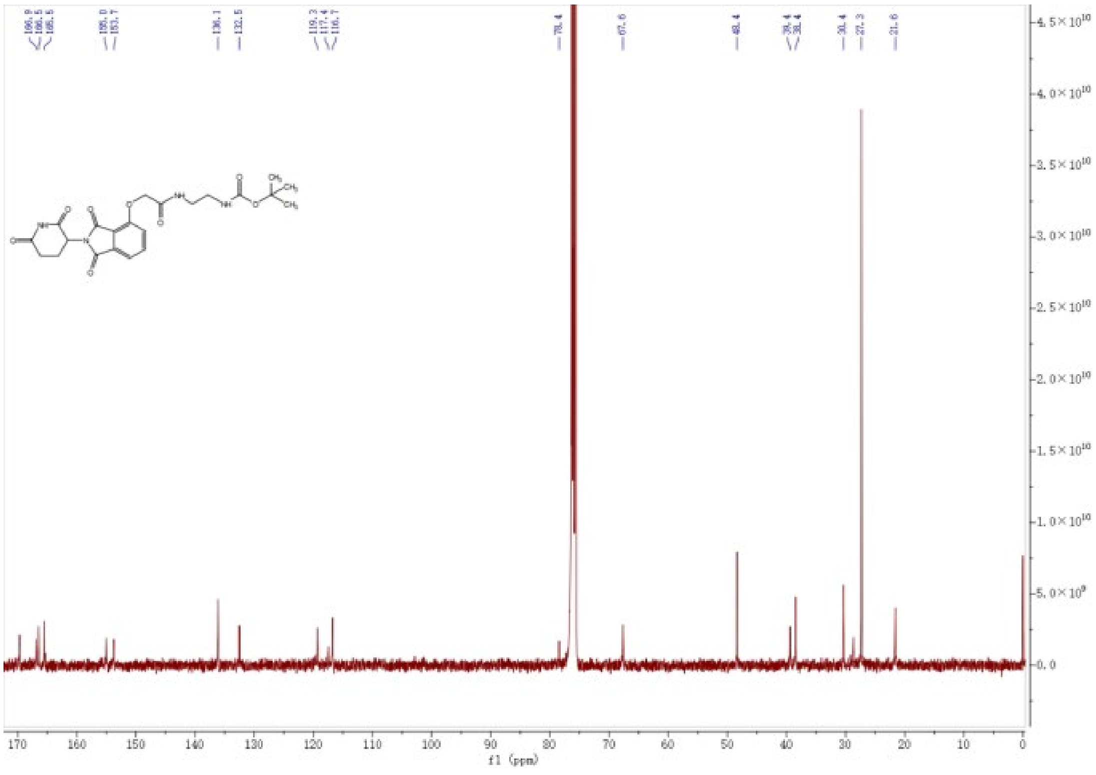

Figure S109 ^{13}C-NMR spectrum of 12e

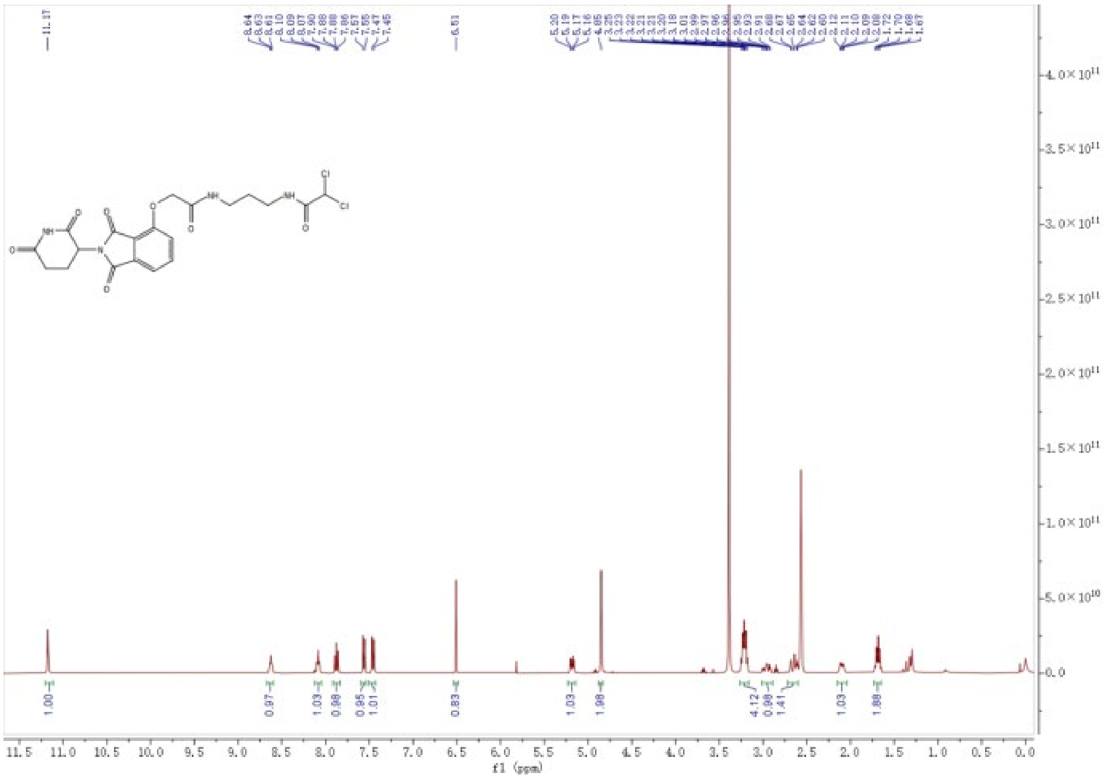

Figure S110 ^{1}H-NMR spectrum of D01

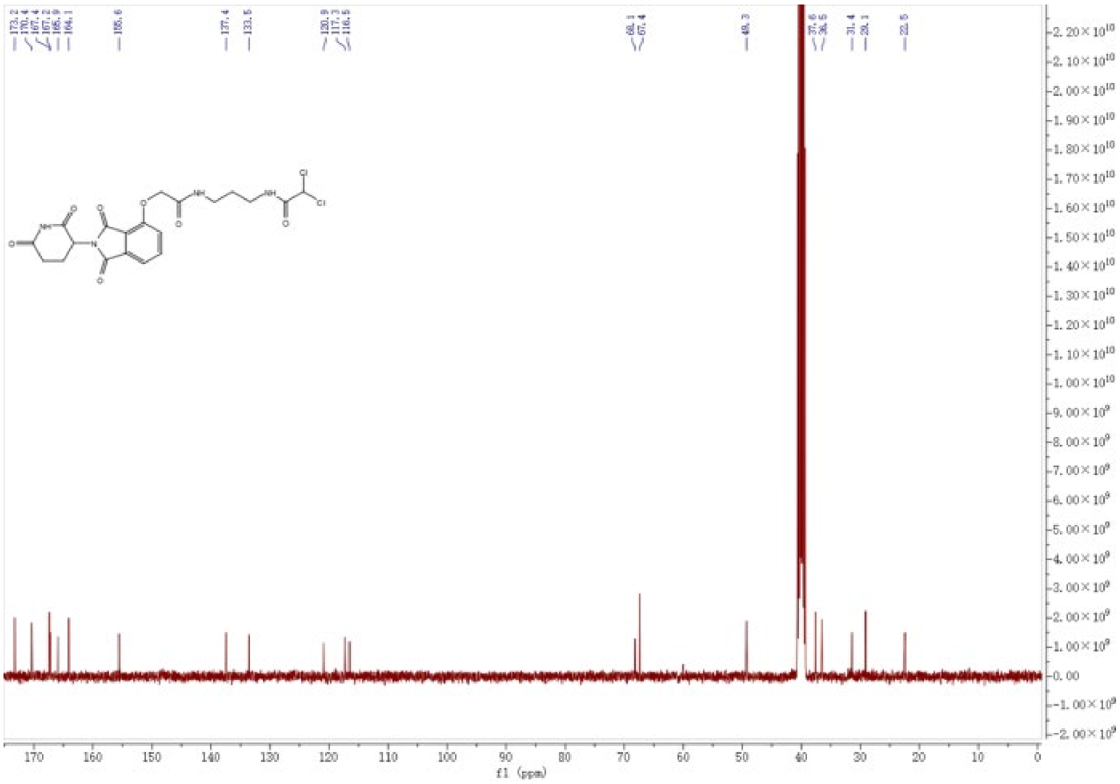

Figure S111 ^{13}C-NMR spectrum of D01

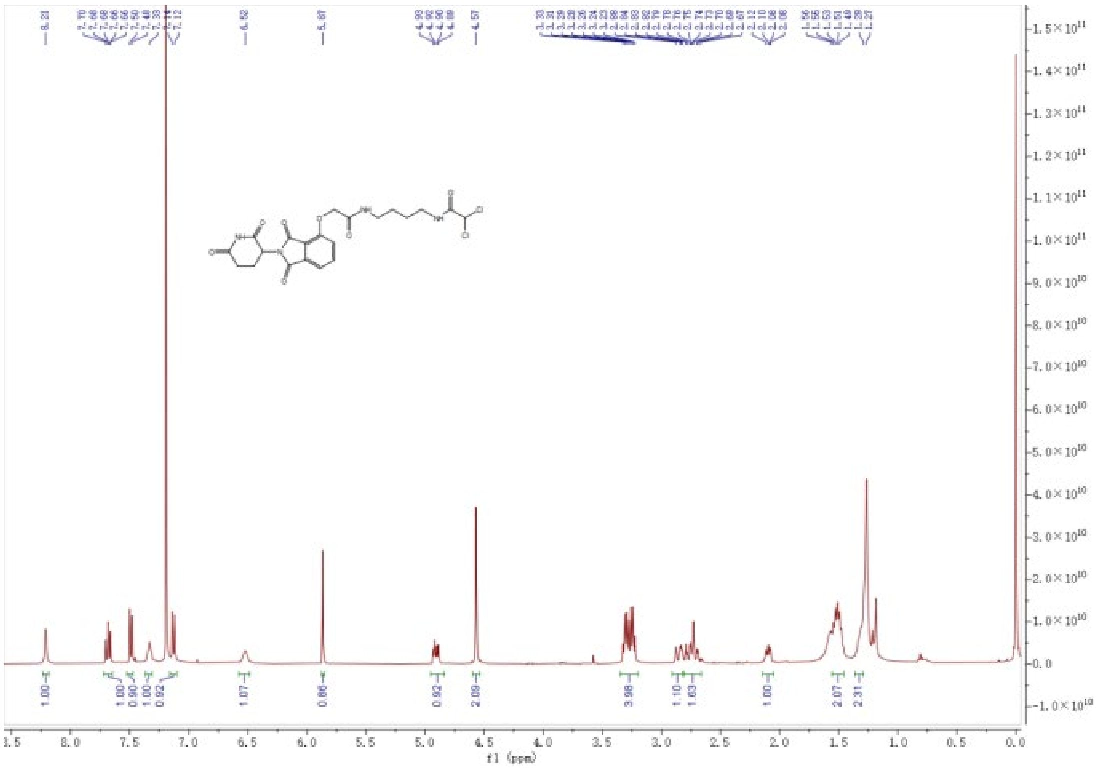

Figure S112 ^{1}H-NMR spectrum of D02

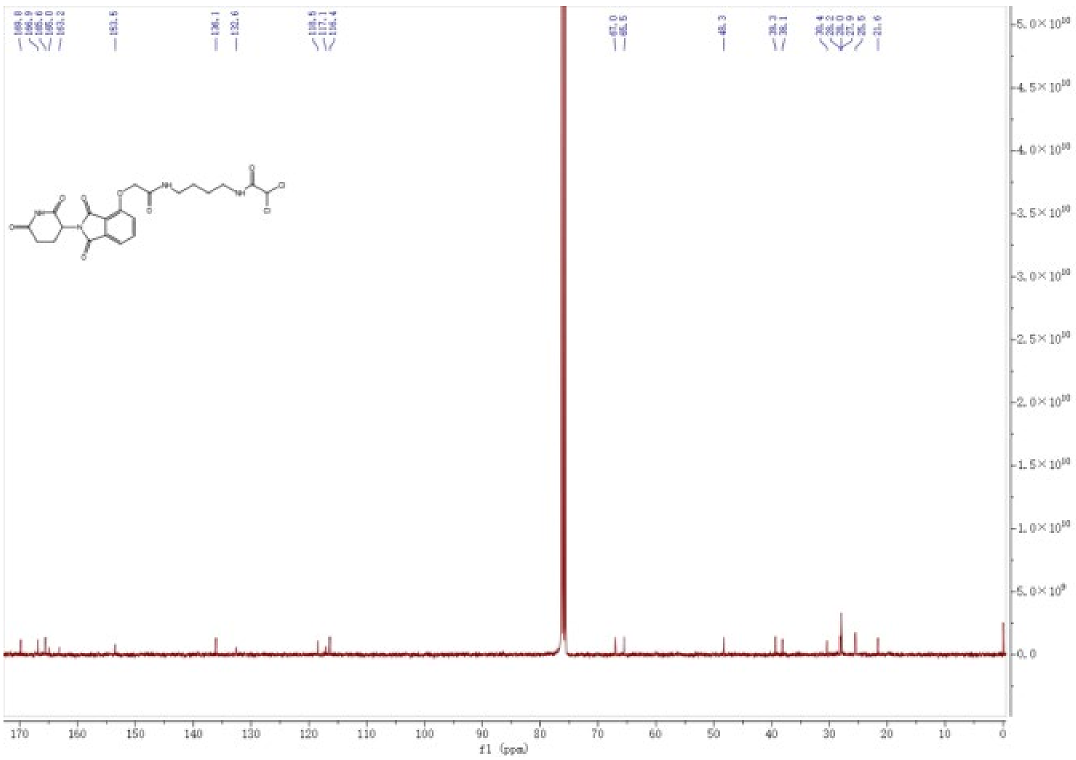

Figure S113 ^{13}C-NMR spectrum of D02

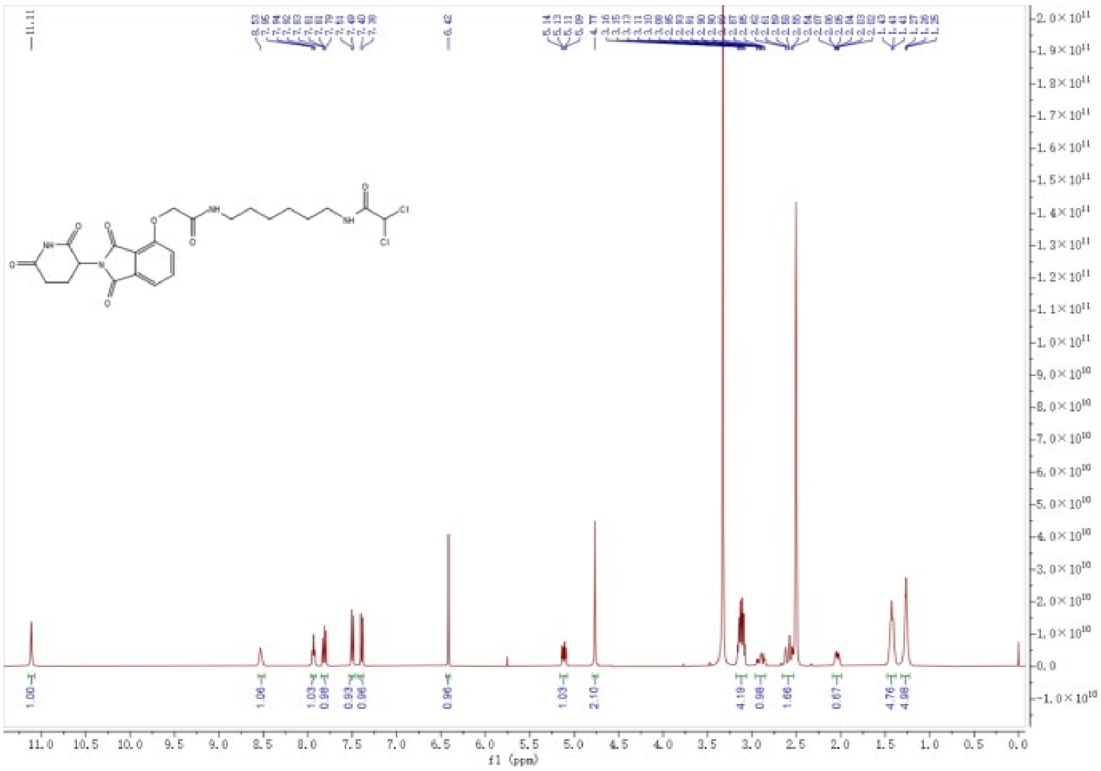

Figure S114 ^{1}H-NMR spectrum of D03

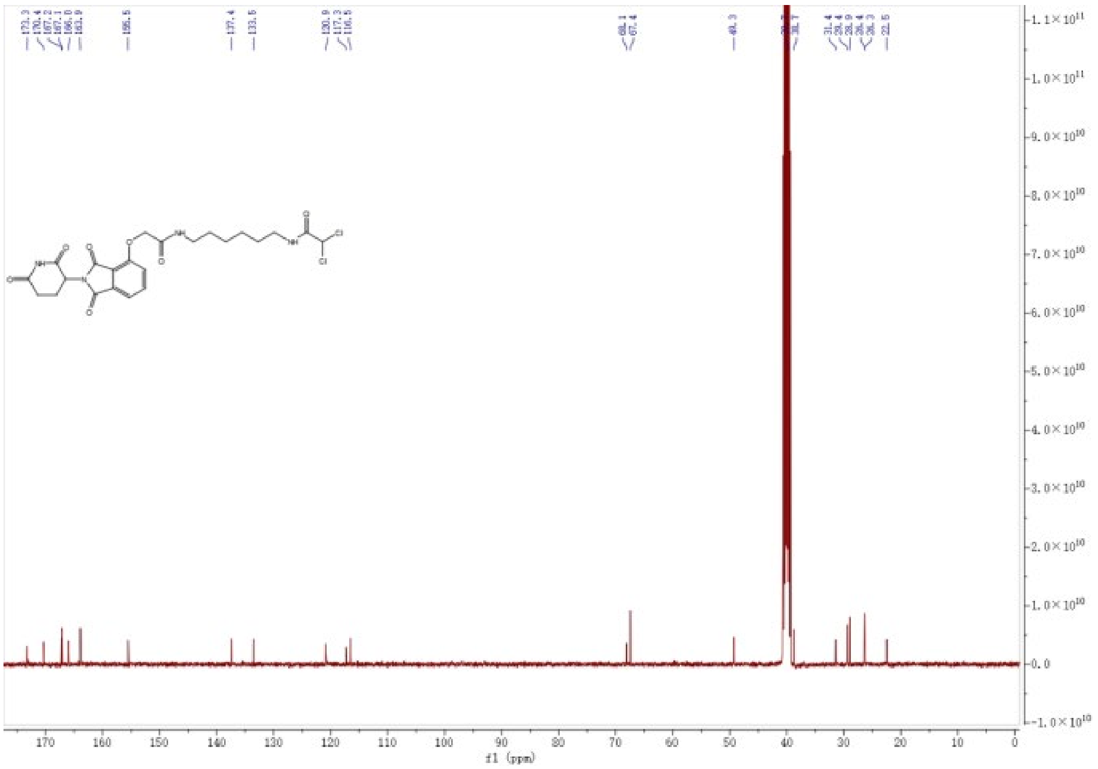

Figure S115 ^{13}C-NMR spectrum of D03

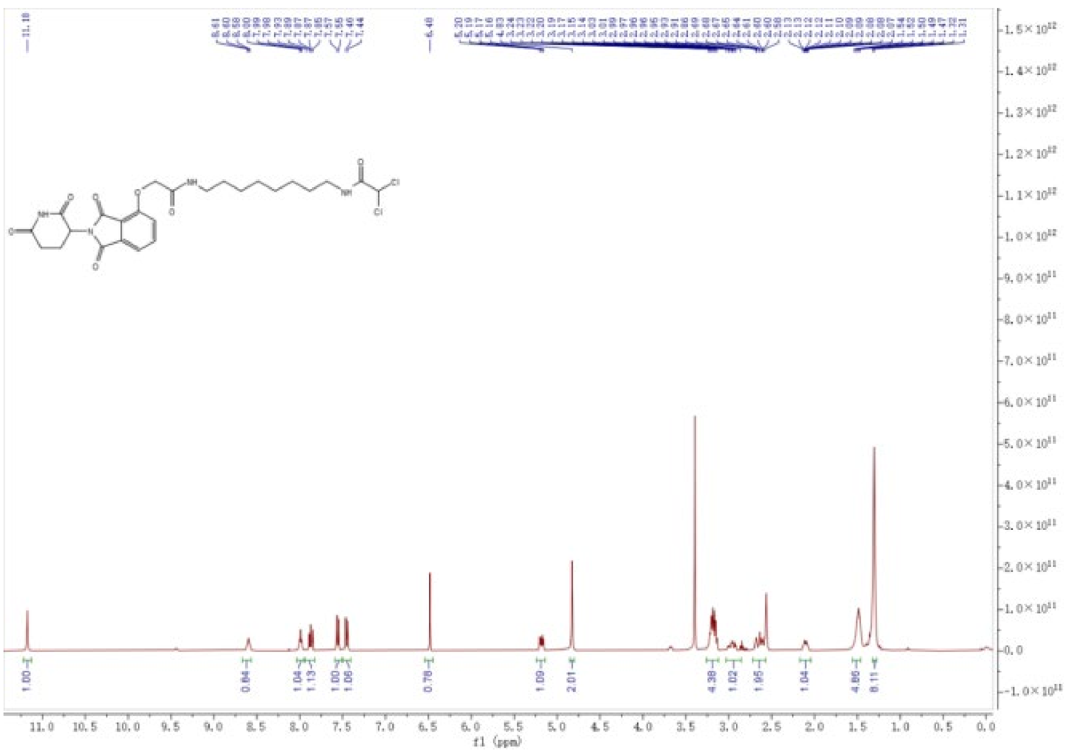

Figure S116 ^{1}H-NMR spectrum of D04

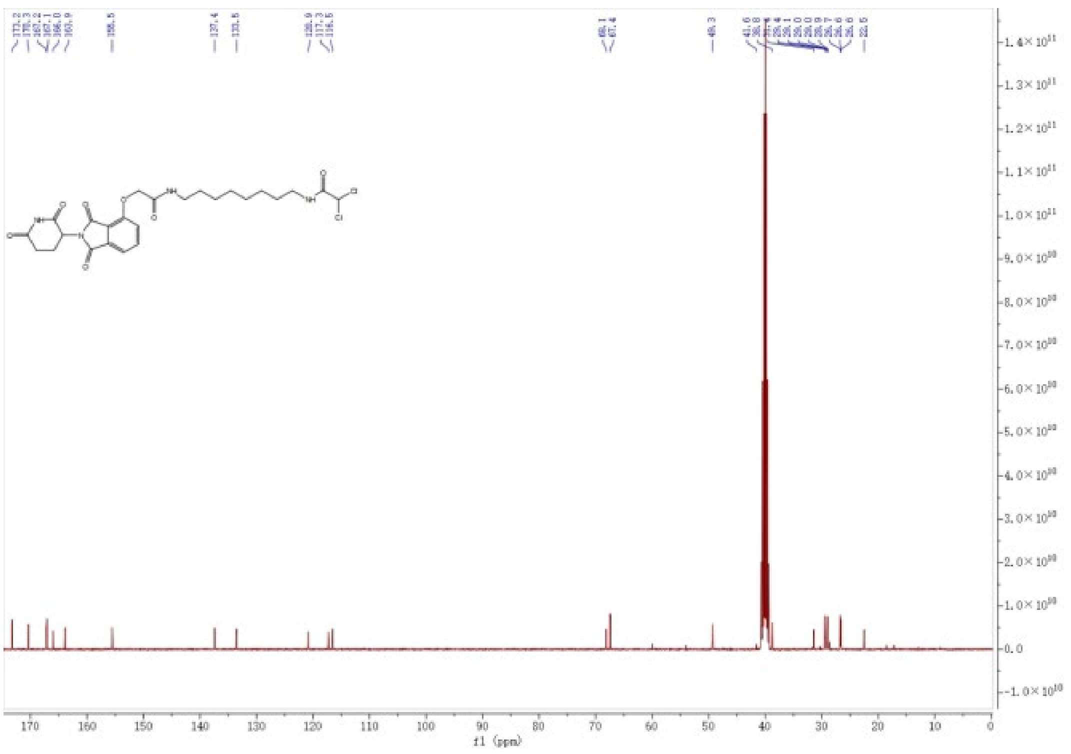

Figure S117 ^{13}C-NMR spectrum of D04

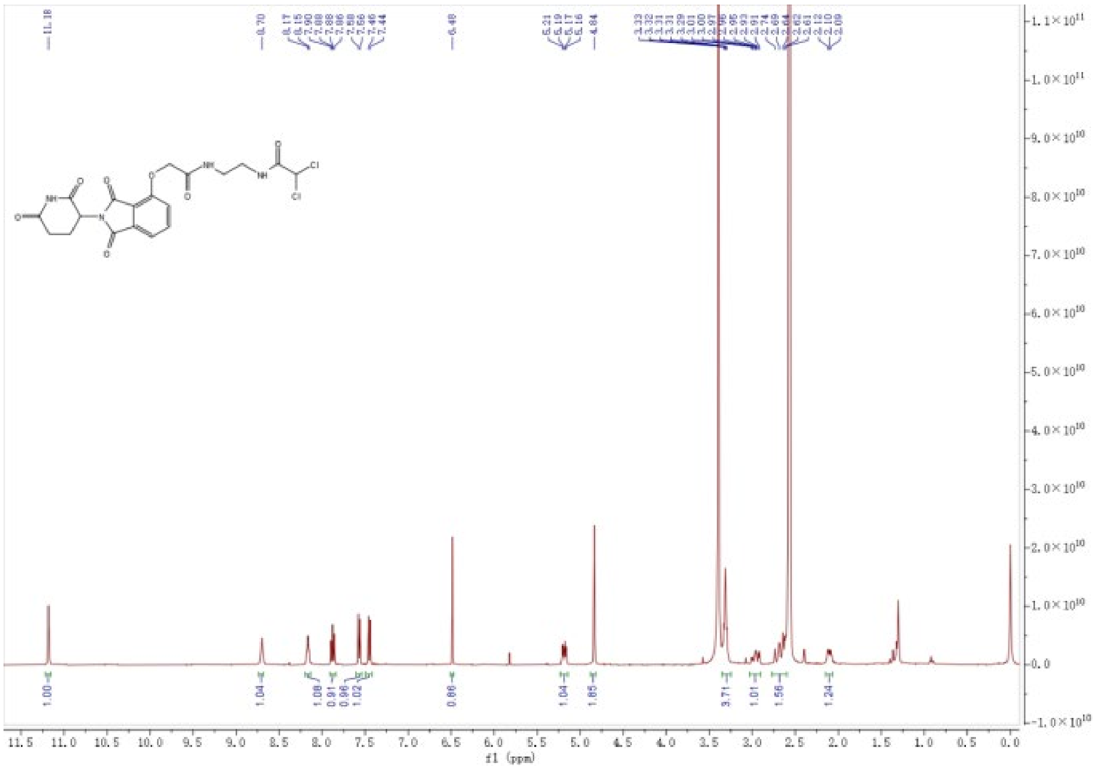

Figure S118 ^{1}H-NMR spectrum of D05

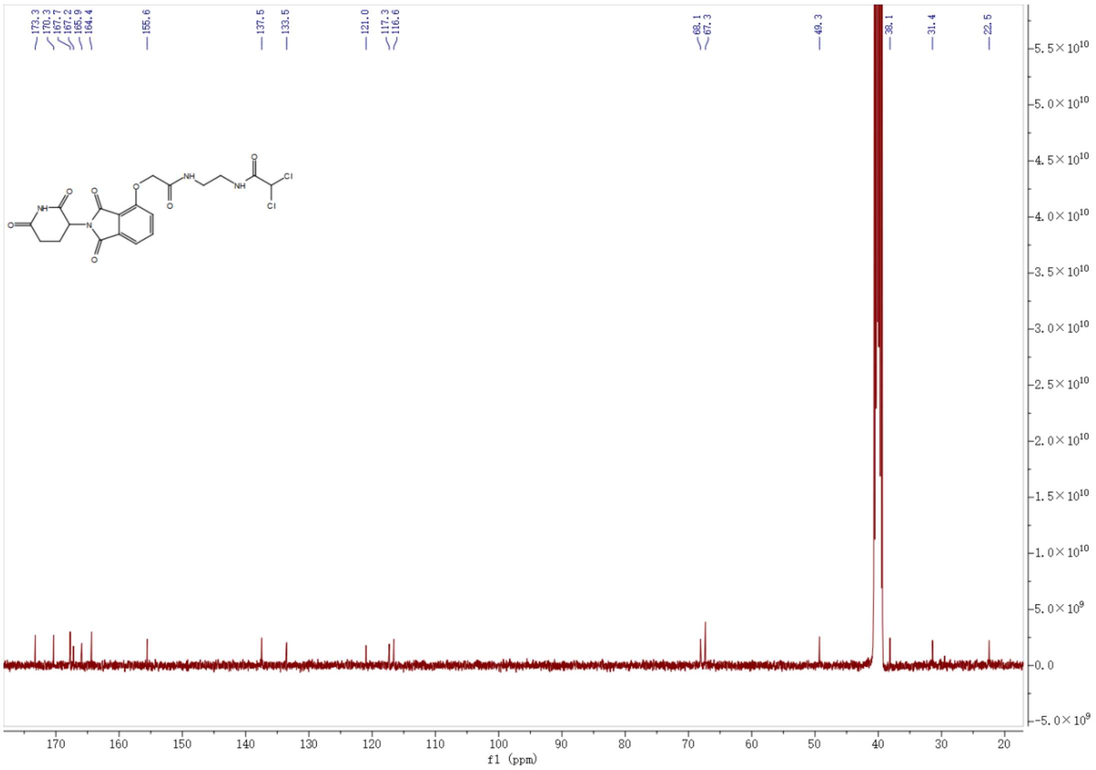

Figure S119 ^{13}C-NMR spectrum of D05
